# Design, Synthesis and Preclinical Evaluation of a Brain-Permeable PET Tracer for P2Y12 Receptor Imaging in the Brain

**DOI:** 10.1021/acs.jmedchem.5c00457

**Published:** 2025-07-25

**Authors:** Emanuel Joseph, Lea H. Kunze, Rebecca Schaefer, Giovanna Palumbo, Benjamin Kugelmann, Stephan Wagner, Sven Lammich, Regina Feederle, Michael Willem, Rudolf A. Werner, Matthias Brendel, Simon Lindner

**Affiliations:** † Department of Nuclear Medicine, LMU University Hospital, LMU Munich, 81377 Munich, Germany; ‡ German Center for Neurodegenerative Diseases (DZNE), 81377 Munich, Germany; § Biomedical Center Munich (BMC), LMU Munich, 81377 Munich, Germany; ∥ Core Facility Monoclonal Antibodies, Helmholtz Munich, German Research Center for Environmental Health, 85764 Neuherberg, Germany; ⊥ Munich Cluster for Systems Neurology (SyNergy), 81377 Munich, Germany; # Russell H. Morgan Department of Radiology and Radiological Sciences, Johns Hopkins School of Medicine, Baltimore 21287 Maryland, United States

## Abstract

Microglia, the innate immune cells of the central nervous system (CNS), act as first responders to brain injury. Their ability to switch between different neuroprotective and neurotoxic phenotypes, plays a central role in maintaining brain homeostasis. Recently, the P2Y12 receptor (P2Y12R) has been identified as a promising molecular biomarker for microglia activity, as its expression level is dependent on microglia phenotype and function. P2Y12R positron emission tomography (PET) might be a valuable diagnostic tool, however, tracers with sufficient brain retention have not been reported so far. Herein, we report a brain-permeable P2Y12R PET tracer for *in vivo* imaging of P2Y12R-positive microglia. Nicotinate [^18^F]**12** exhibited nanomolar affinity and specificity for the target receptor and showed a reduced uptake in microglia-depleted (PLX) mice, in comparison to WT and Trem2 knockout (Trem2^–/–^) mice. *Ex vivo* immunohistochemistry (IHC) and PET data revealed a strong correlation between microglia abundance, P2Y12R expression levels and tracer uptake.

## Graphical abstract

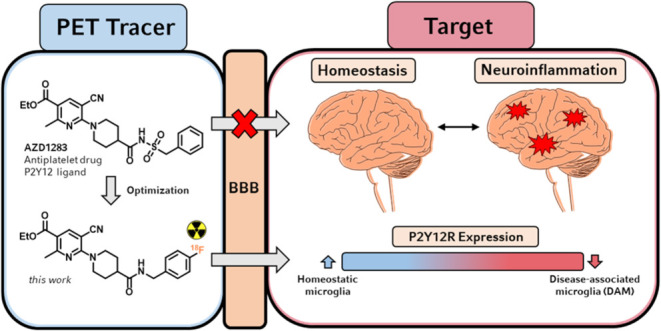

## Introduction

Microglia are the resident immune cells of the central nervous system (CNS) and play an important role in the protection, homeostasis and surveillance of the brain parenchyma.^1–3^ Thus, a broad spectrum of functions and abilities can be attributed to them, like the constant scanning of the surrounding brain tissue, the secretion of inflammatory cytokines or reactive oxygen species, the removal of cellular debris and de- and remyelination of neurons.^4–6^ This versatility is a result of the ability to switch between different phenotypes, each with its own cell morphology, gene expression and function.^7,8^ Thus, microglia can respond rapidly to injury or pathogens through a dynamic and reversible shift from homeostatic to activated microglia, which initiate an acute inflammatory reaction and subsequent neuroprotective care. Transcriptome analysis indicates that the aging process affects the active interplay of damage and repair, resulting in a reduction of homeostatic microglia throughout the lifespan.^9–11^ In the onset and progression of neurodegenerative diseases, such as Alzheimer's disease (AD), this natural effect is drastically amplified, leading to a microglia polarization toward chronic activation and neuroinflammation.^12,13^ So far, it has proven challenging to obtain sufficient and satisfactory *in vivo* data that reflect the heterogeneous spectrum of microglia phenotypes and activation states. However, this real-time information would be necessary to develop new therapeutic approaches for neurodegenerative diseases, based on the manipulation of microglia polarization in order to prevent or reverse maladaptive activation.^14,15^ Positron emission tomography (PET), a noninvasive and highly specific imaging method, represents a particularly suitable tool for imaging dynamic and complex processes *in vivo*, such as neuroinflammation. At present, PET is employed in clinical research to assess microglia activation. The most prevalent target to image activated microglia is the 18 kDa translocator protein (TSPO), although the biomarker is subject to several limitations. PET data interpretation is demanding since TSPO is expressed not only on microglia but also on endothelial cells and astrocytes. Furthermore, at elevated expression levels, it is not possible to distinguish between neuroprotective and neurotoxic phenotypes.^16,17^ Genetic polymorphism in humans can also result in low and high binding of PET tracers, creating a demand for more specific and universal imaging targets. While this exemplary biomarker and others are commonly used in research related to the activated phenotype, there are currently no PET tracers available for clinical use in imaging homeostatic microglia.^18^ In this context, the G-protein coupled purinergic 2Y type 12 receptor P2Y12R has emerged recently as a promising molecular target. In case of brain injury, P2Y12R detects extracellular nucleotides such as ADP and ATP, released from damaged neurons and glia cells and microglia activation is subsequently initiated.^19,20^ The morphological and functional changes of microglia from anti-inflammatory to pro-inflammatory states are associated with a distinct and rapid P2Y12 receptor down-regulation.^9,21^ This contrast emphasizes P2Y12R as a promising surrogate for the dynamic and complex neuroinflammation process. The receptor has a clear functional involvement in microglia activation and is found specifically on microglia within the CNS.^22^ In the periphery, it is found on platelets, where it plays an essential role as ADP receptor in the signaling pathway to platelet aggregation.^23,24^ To date, several radiotracers targeting P2Y12R have been developed (Figure 1). However, the reported compounds have only poor brain uptake and are therefore unsuitable for *in vivo* brain imaging. Van der Wildt and co-workers published two potent thienopyrimidine-based ligands, [^11^C]**1** and [^18^F]**2**, which turned out to be substrates for efflux transporters, hindering their brain accumulation.^25^ Most recently, the same group published another highly affine P2Y12R PET tracer **3**, based on a pyrazolidine-3,5-dione derivative. Unfortunately, this tracer demonstrated low *in vivo* stability and brain uptake.^26^ Other attempts focused on the development of small molecules based on the structure of the P2Y12R antagonist and platelet aggregation inhibitor AZD1283 **5**, but the compounds [^11^C]**4** and [^11^C]**5** were unable to cross the blood-brain barrier (BBB).^27–29^

**Figure 1:**
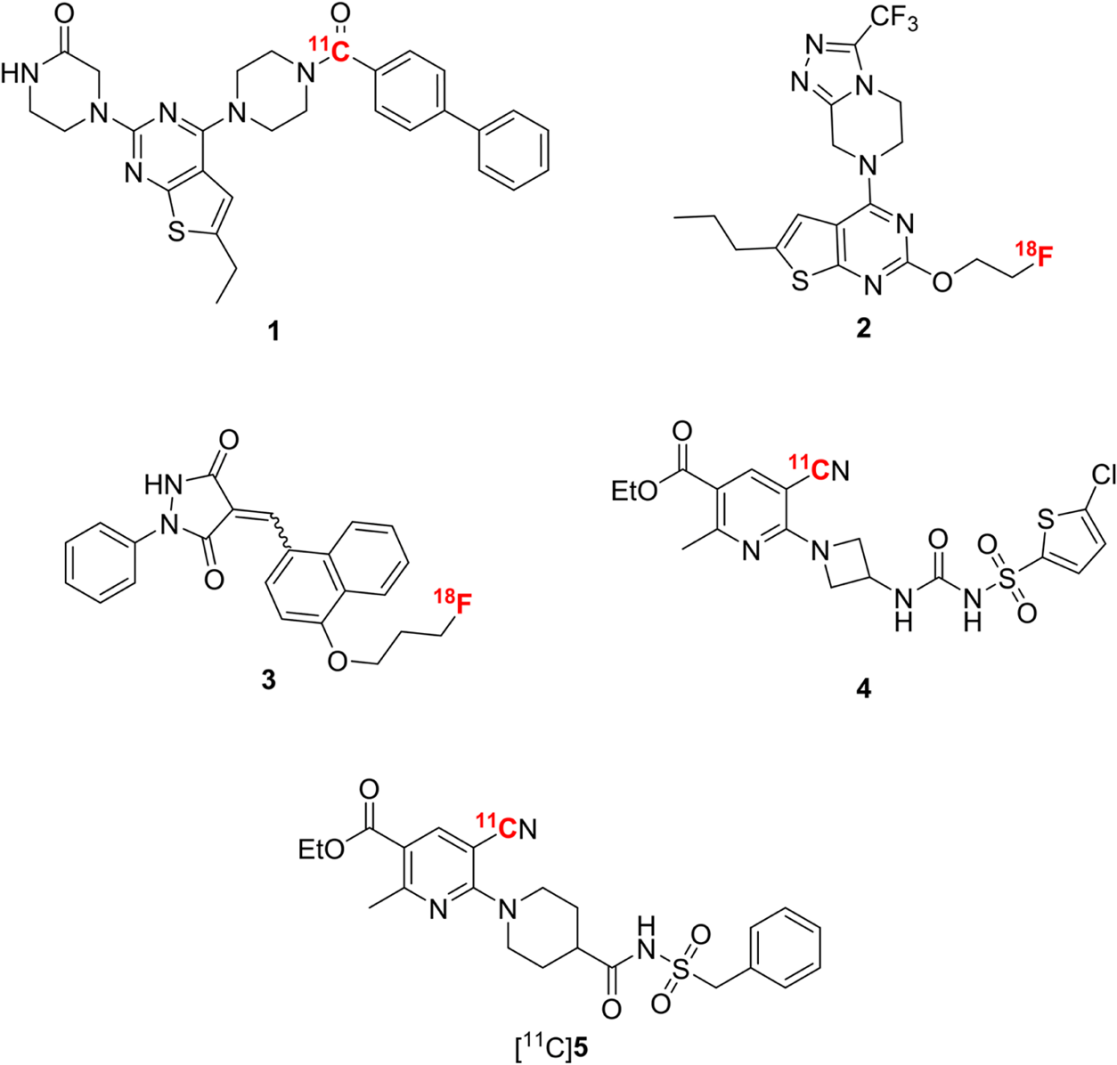
Published P2Y12R radioligands.^25–29^

Nevertheless, the development process of AZD1283 **5** has yielded interesting progenitor compounds, like compound **6**, that promise better brain accessibility while retaining a rather low level of affinity for the target receptor (Figure 2).^30,31^ On the basis of these structures, a set of 16 new compounds (**7**–**22**) was designed, synthesized and validated, aiming to provide a novel PET tracer candidate. The intent was to discover high affinity compounds while maintaining a sufficient brain permeability by varying substituents or linkers. Compound **12** was subsequently selected for further examination as a specific and brain-compatible PET tracer, due to its high target affinity and promising physicochemical properties. The compound was radiolabeled with fluorine-18 and tested in a set of *in vitro* and *in vivo* studies to confirm brain uptake and specificity. This work presents a further step toward P2Y12R imaging in the CNS by means of brain-permeable radiotracers, which could facilitate the characterization of P2Y12R expression and activity in the context of neuroinflammation.

**Figure 2:**
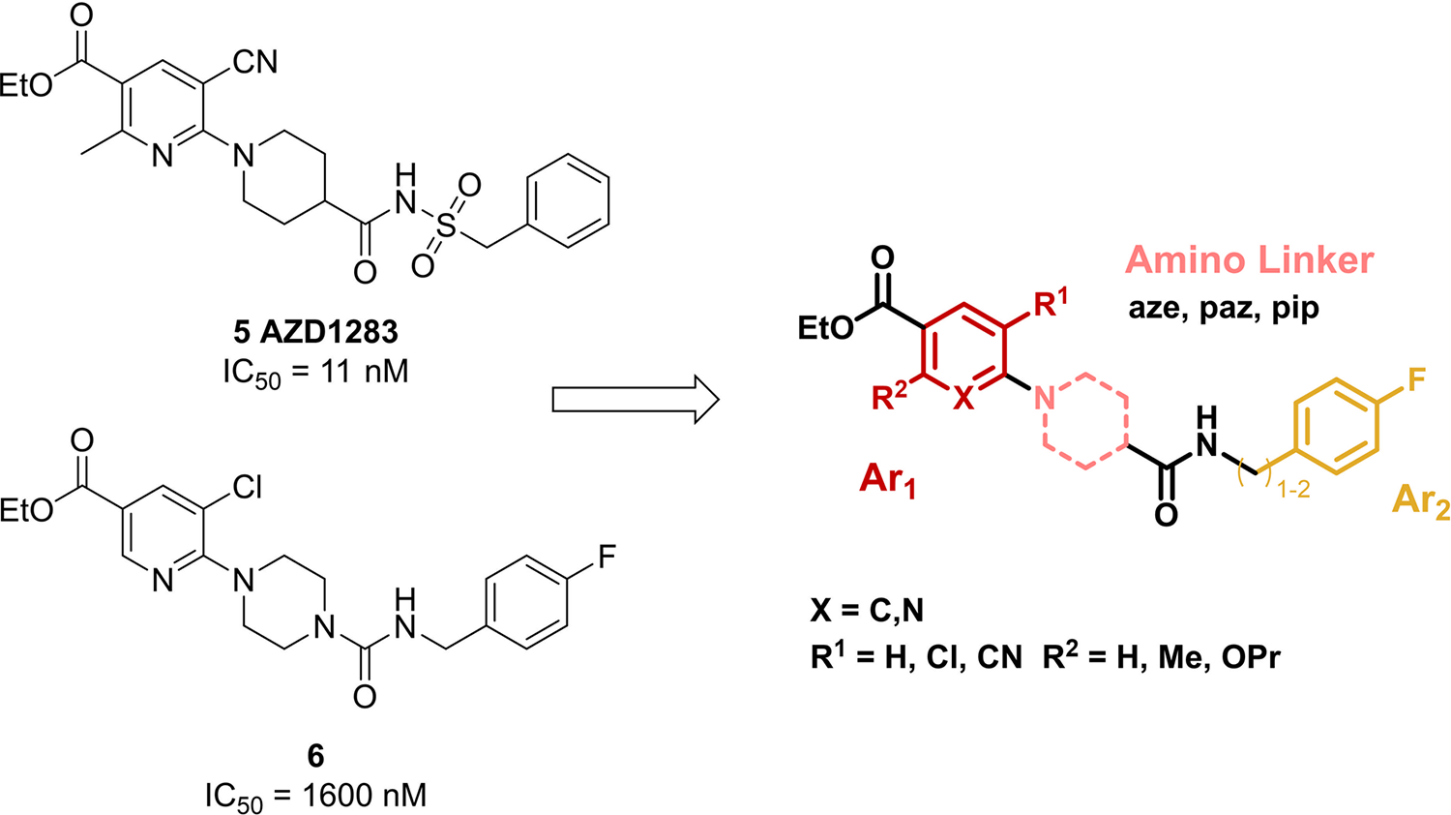
Lead compounds **5** (AZD1283) and **6** and derived variable fragments for structure modification. *In vitro* binding affinity (IC_50_) for the P2Y12 receptor in Chinese hamster ovarian cell membranes;^30^ Ar_1_, (Hetero)­Aryl 1; aze, 3-azetidinyl; paz, 4-piperazinyl; pip, 4-piperidinyl; Ar_2_, Aryl 2.

## Results

### Molecular Design and Docking

Among the three crystal structures for P2Y12R provided by the protein data bank (PDB), the4NJTstructure was chosen for docking experiments, since it is cocrystallized with AZD1283 **5**.^32^ The quaternary structure of the receptor is characterized by a group of 7 transmembrane helices (TM), with the AZD1283 binding pocket located between TMIV and TMVII (Figure 3A). To analyze the structure–activity relationship between ligand and receptor, AZD1283 was formally divided into three fragments: the (hetero) aryl fragment Ar_1_, the amino linker and the aryl fragment Ar_2_ (Figure 2). Using these substructures, we identified central interactions such as hydrogen bonding or π–π stacking using the drug discovery platform molecular operating environment (MOE) (Figure S1). In order to preselect the most promising structures for use as PET tracers, the proposed structures were limited to those that could be radiolabeled with fluorine-18. For docking experiments a set of potential P2Y12R ligands was generated, based on reported examples from literature as well as new motifs and substructures generated from scratch using MOE’s Scaffold Replacement tool. The tracer candidates need to be able to cross the BBB in sufficient amounts. Thus, we used two different algorithms, the CNS MPO Score^33^ and the BBB Score,^34^ to assess the likelihood of brain permeability of the respective compound. In total, a set of 55 structures was tested *in silico* in docking studies (Table S1). From these structures, compounds **5**-**22** were selected for structure–activity relationship (SAR) analysis, based on the docking score S and SAR results from literature (Table 1). Also, they all exhibit promising BBB or CNS MPO scores for successful application as CNS drug. The 2D interaction diagram of the binding pocket shows tight hydrogen bonds for the ethyl 6-aminonicotinate backbone with the residues Tyr105, Tyr109, Asn159 and Cys194 (Figure 3A,B). The docking scores for the compounds **9**/**11**/**12** (−9.23 *vs* −9.79 *vs* −10.03), and **15**/**17**/**18** (−9.66 *vs* −10.15 *vs* −10.44) revealed a superior affinity for the nitrile over chloride and hydrogen in position 5 of the pyridine ring.^30,31^ However, nitrile substituents exhibit a higher topological polar surface area (TPSA), which is ultimately reflected in lower BBB scores. Thus, chloride or hydrogen substituents at position 5 were still added to the set of test compounds to increase the likelihood of brain permeability. The series **10**/**11** and **16**/**17** indicated that a methyl group at position 2 may foster higher docking scores. The highest S values were obtained with compounds **13** and **19**, which have an aryl scaffold instead of the pyridine ring. A pyridine-fused lactone was introduced in **8** and **14**, to account for an increased microsomal stability and affinity.^35^ The lactones also displayed higher CNS scores than comparable nicotinates, presumably due to their reduced molecular weight and decreased flexibility. However, the lactones displayed the lowest docking scores, although a beneficial effect on brain permeability can be expected. Variations on the amino linker were made including piperazinyl (**6–8**), piperidinyl (**9–19**), or azetidinyl (**20–22**) residues, corresponding to the selection made by Bach and co-workers from a wide range of different nitrogen-heterocycles.^36^ A comparison of the S scores for the duplets **6**-**7**, **10** and **12** and **21**-**22** show that the amino linker exerts a minor influence on the predicted affinity value of the ligand. Whereas the usage of the azetidine fragment results in higher CNS scores, the S score is similar to the piperazine and lower than the piperidine residue. The sulfonyl amide of AZD1283 **5** displays a particularly strong interaction with the residues Lys280 and Arg256 (Figure 3A,B). A comparison with the predicted interaction profile of **6** in the binding pocket reveals that the omission of the sulfonyl amide group leads to weaker hydrogen bonds for these residues (−8.8 and −5.3 *vs* −3.6 and −4.2 kcal/mol) (Figure 3C,D) reflecting the difference in IC_50_ values of both compounds (Figure 2).^30,31^ Nevertheless, a substitution of the sulfonyl amide with a less polar group is essential, because brain accessibility is severely limited by the strong hydrophilic nature of the sulphonyl group. Replacing the sulphonyl group in **5** with a methylene group and prolonging the carbon chain toward the aryl residue led to a significant decrease in hydrophilicity in compound **18** compared to **5**, while maintaining a high docking score (−10.63 *vs* −10.44). In general, compound **15–19** (−9.66 to −11.03) exhibited higher docking scores than the corresponding homologues **9–13** (−9.23 to −10.58) supporting the hypothesis that the methylene group replacement is beneficial for receptor affinity. The aryl moiety offers a feasible position for radiofluorination by means of nucleophilic aromatic substitution. Experiments with different substituents on the aromatic ring of AZD1283 demonstrated, that the addition of a fluorine substituent in position 4 had only a minor effect on the affinity.^31^

**Figure 3:**
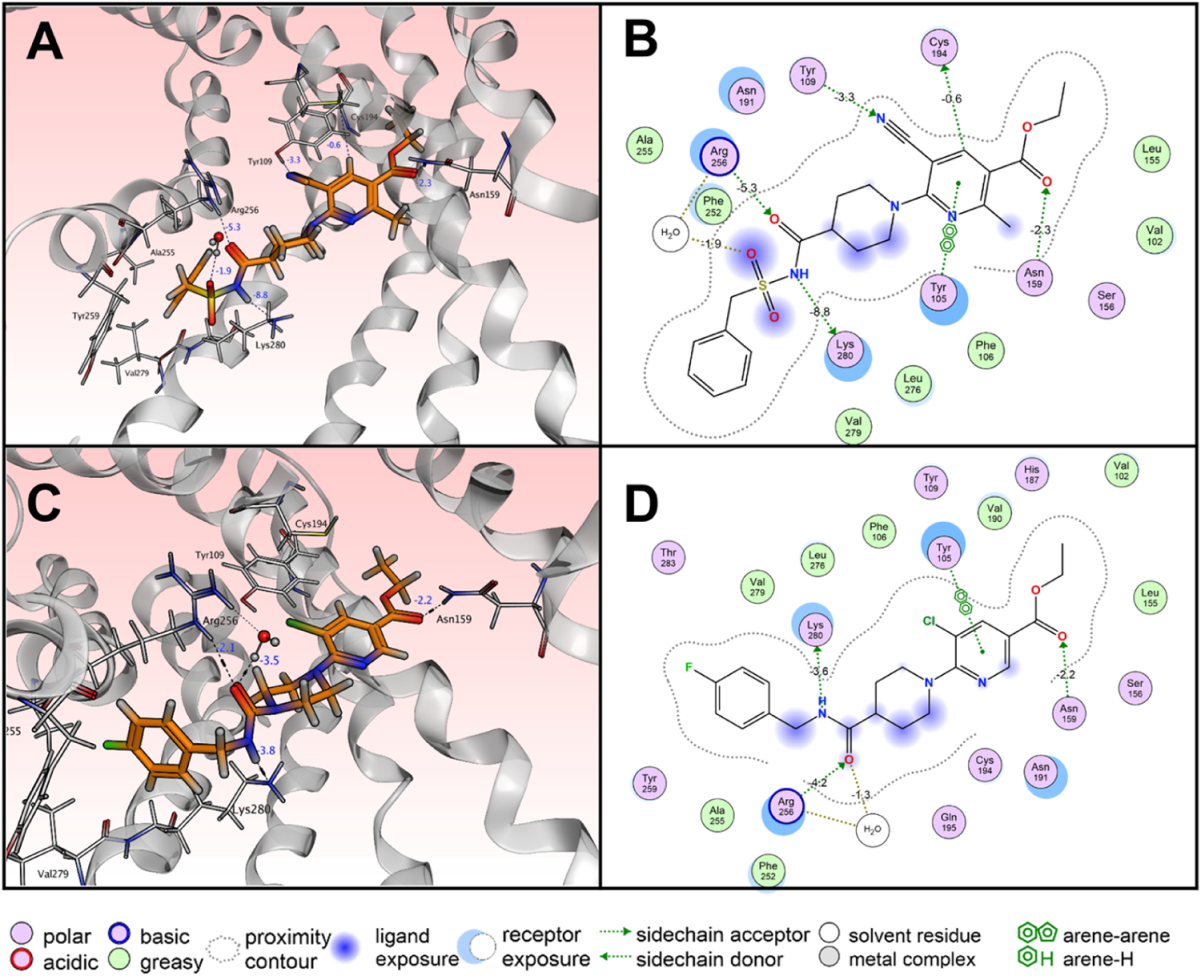
Molecular docking of AZD1283 **5** and **6**. (A) Predicted binding mode of AZD1283 **5** to the P2Y12R (PDB: 4NTJ). (B) 2D interaction diagram for AZD1283 **5**. (C) Predicted binding mode of compound **6** to the P2Y12R (PDB: 4NTJ). (D) 2D interaction diagram for **6**.

**Table 1:**
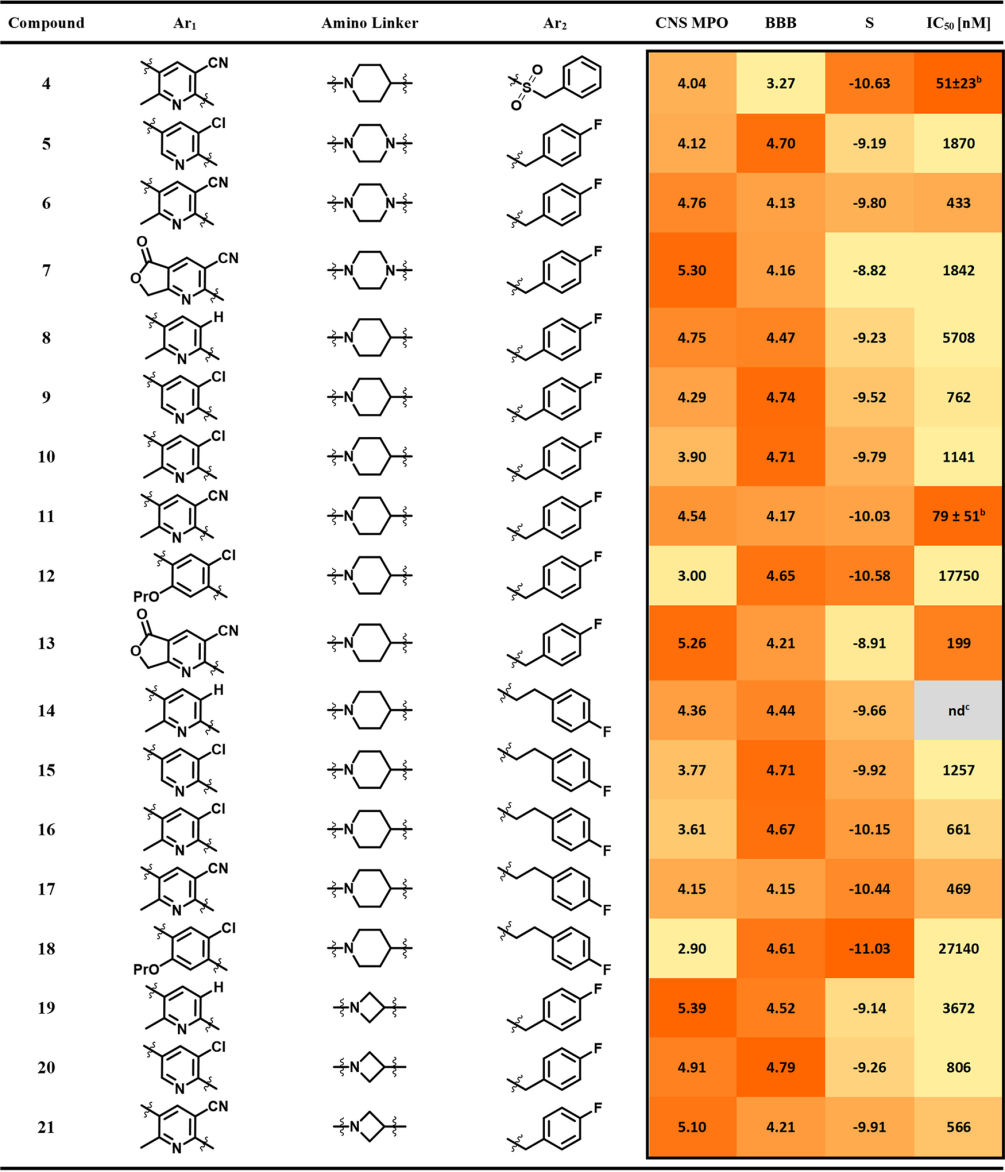
*In Silico* Data and IC_50_ Values for Compounds **5–22** ^a^

a BBB and CNS MPO score calculated with Marvin JS v23.11; S score calculated with MOE; IC_50_ values calculated with GraphPad Prism v8.4.3 based on experimentally measured inhibition values (triplicates, mean); red color indicates high values for CNS MPO, BBB and S scores and low IC_50_ values, yellow color vice versa.

b Experimentally measured inhibition values from three independent experiments (each in triplicate) expressed as mean ± SD.

c nd, not detected.

### Chemistry

The synthesis of compound **6**-**22** is based on the “reverse method” described by Bach et al. reacting 6-chloronicotinates with the corresponding amine building block.^30^ The Boc-protected *N*-heterocycles piperazine **23**, isonipecotic acid **26** and 3-azetidinecarboxylic acid **27** were used as starting materials to synthesize amine building blocks for the amino linker (Scheme 1). Boc-protected piperazine **23** reacted with 4-fluorobenzyl isocyanate to amide **24** in 53% yield, followed by Boc-deprotection with HCl in dioxane to give the free amine **25** in 68% yield. **26** and **27** were activated with HBTU and DCC and treated with 4-fluorobenzylamine or 2-(4-fluorophenyl)­ethan-1-amine to give the corresponding amides **28**-**30**. Subsequent Boc-deprotection with HCl in dioxane yielded amine **31**-**33** (86, 35 and 72% yield over two steps). The AZD1283 analogues **6**-**7**, **9**-**12**, **15**-**18** and **20**-**22** were prepared by treating 6-chloronicotinates **34–37** with the respective amine building blocks **25** or **31–33** (15–95% yield) (Scheme 2). Compounds **8** and **14** with a fused lactone moiety, were synthesized according to Scheme 3. After the preparation of **38** according to a literature procedure, the amine building blocks **25** and **31** were used to obtain **8** and **14** in 23 and 22% yield.^37^ Compounds **13** and **19** contain a benzene ring instead of the pyridine motif and were synthesized based on the commercially available 4-fluorsalicylic acid **39** (Scheme 4). Fisher esterification of **39** with conc. H_2_SO_4_ in EtOH gave **40** in 65% yield, which was then alkylated with propyl bromide to obtain **41** in 96% yield. Chlorination in position 4 with NCS yielded compound **42** (33% yield). Reaction with amine **31** and **32** gave the AZD1283 analogues **13** and **19** in 34 and 17% yield. Precursor **45** was prepared for radiolabeling experiments (Scheme 5). Boc-protected isonipecotic acid **26** was transferred to amine **44** through an amide coupling with 4-borpinacol ester benzyl amine and subsequent deprotection (27% yield over two steps). 6-Chloronicotinate **37** was treated with **44** to obtain the precursor **45** (85% yield).

**Scheme 1:**
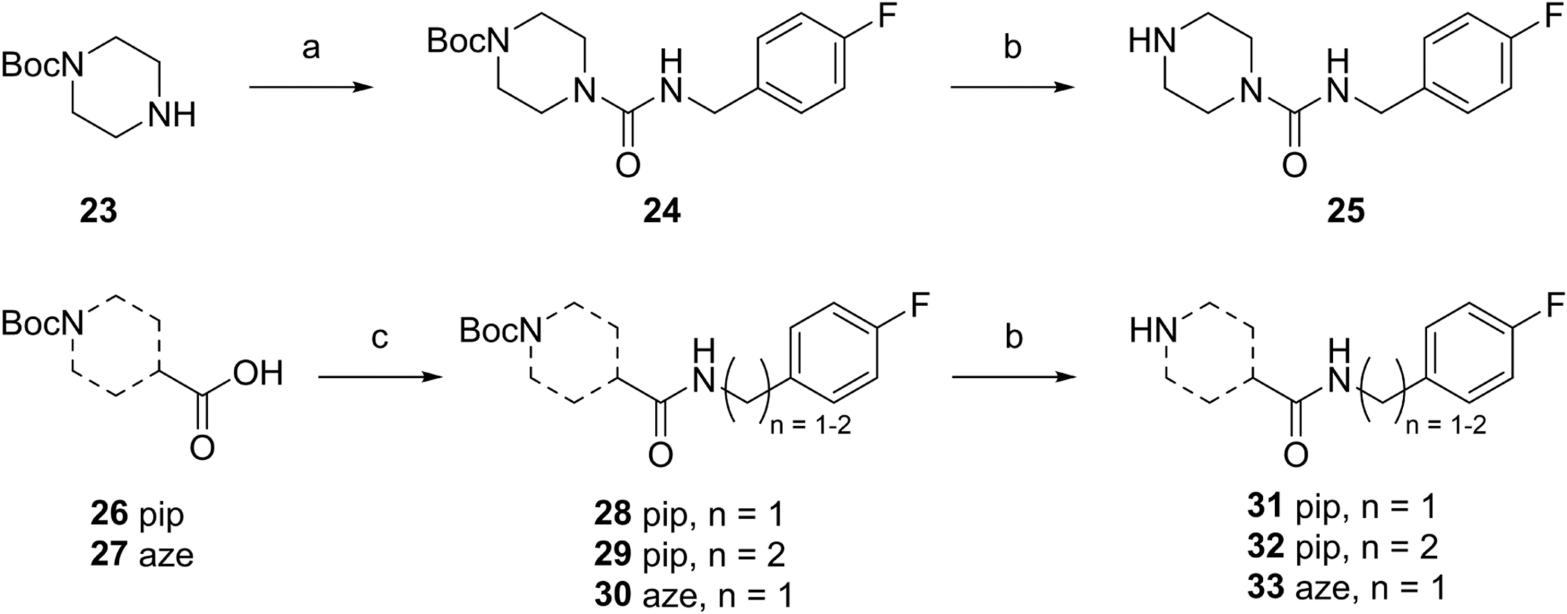
Synthesis of Amine Building Blocks **25** and **31**–**33** ^a^

a Reagents and reaction conditions: (a) 4-FBnNCO, THF, rt, 16 h; (b) 4 M HCl, dioxane, rt, 4 h; (c) 4-fluorobenzylamine or 2-(4-fluorophenyl)­ethan-1-amine, HBTU, DCC, DIPEA, DCM, rt, 16 h; pip, piperidinyl; aze, azetidinyl.

**Scheme 2:**
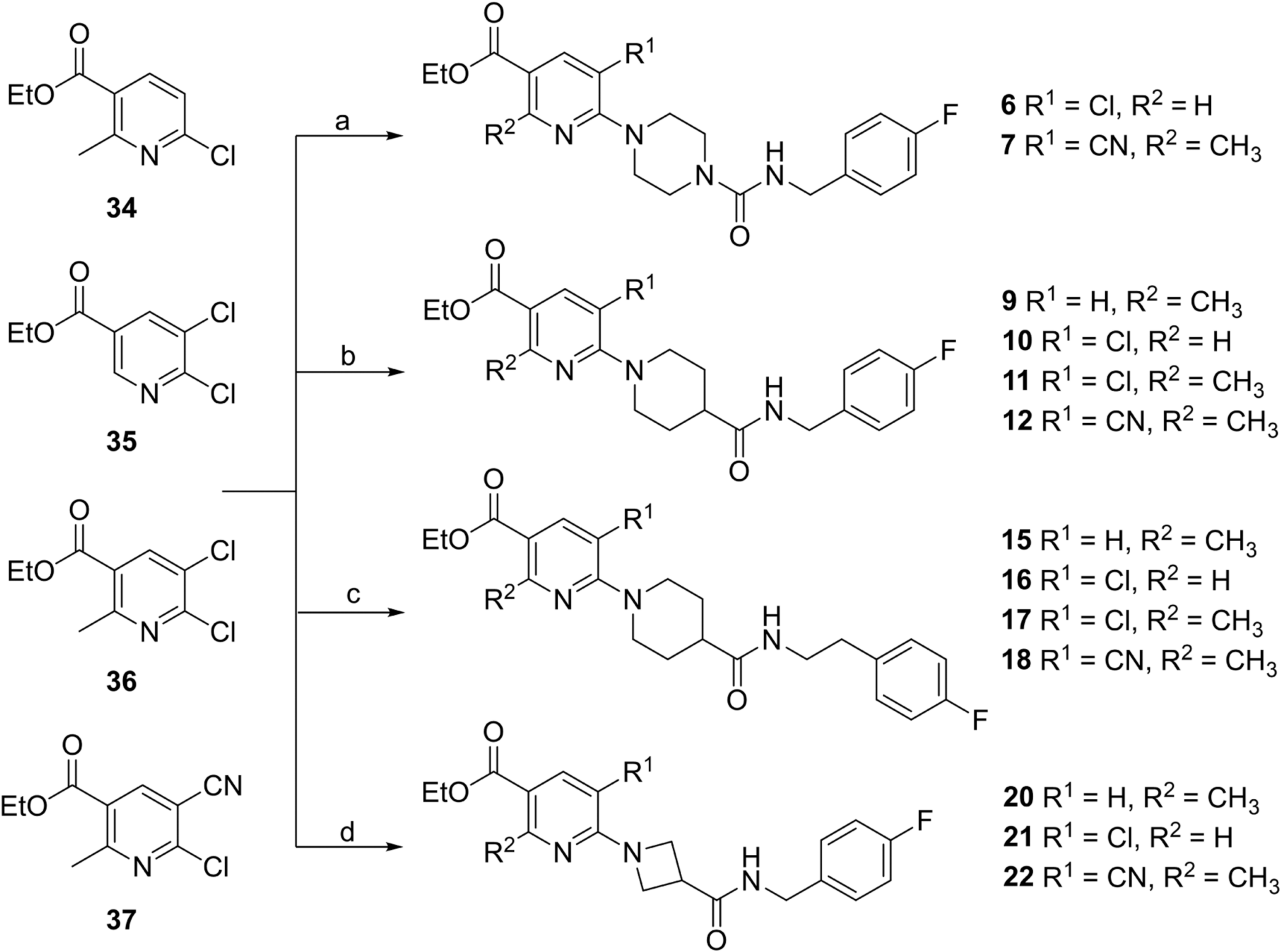
Synthesis of AZD1283 Analogues **6-7**, **9**–**12, 15–18, 20–22** ^a^

a Reagents and reaction conditions: (a) **25**, TEA, EtOH, 72 °C, 16 h; (b) **31**, TEA, EtOH, 72 °C, 16 h; (c) **32**, TEA, EtOH, 72 °C, 16 h; (d) **33**, TEA, EtOH, 72 °C, 16 h.

**Scheme 3:**
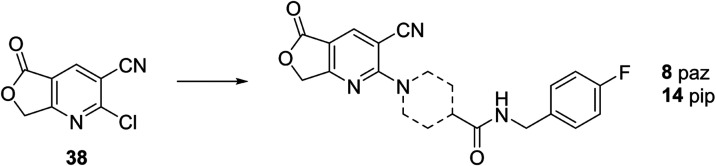
Synthesis of AZD1283 Analogues **8** and **14** ^a^

a Reagents and reaction conditions: **25** or **31**, TEA, EtOH, 72 °C, 16 h; paz, piperazinyl; pip, piperidinyl.

**Scheme 4:**
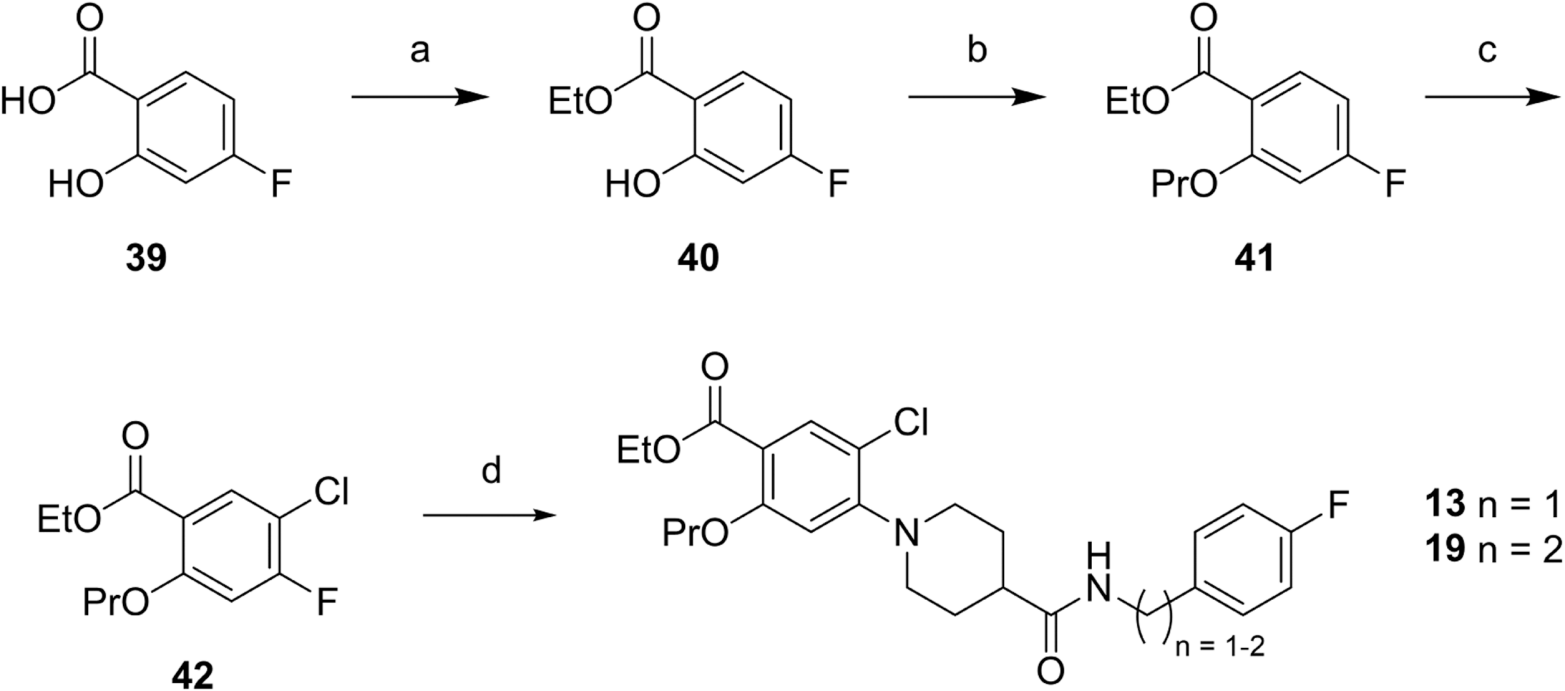
Synthesis of AZD1283 Analogues **13** and **19** ^a^

a Reagents and reaction conditions: (a) H_2_SO_4_, EtOH, 72 °C, 8 h; (b) Propyl bromide, K_2_CO_3_, MEK, 80 °C, 48 h; (c) NCS, rt, 16 h; (d) **31** or **32**, K_2_CO_3_, DMSO, 120 °C, 24 h.

**Scheme 5:**
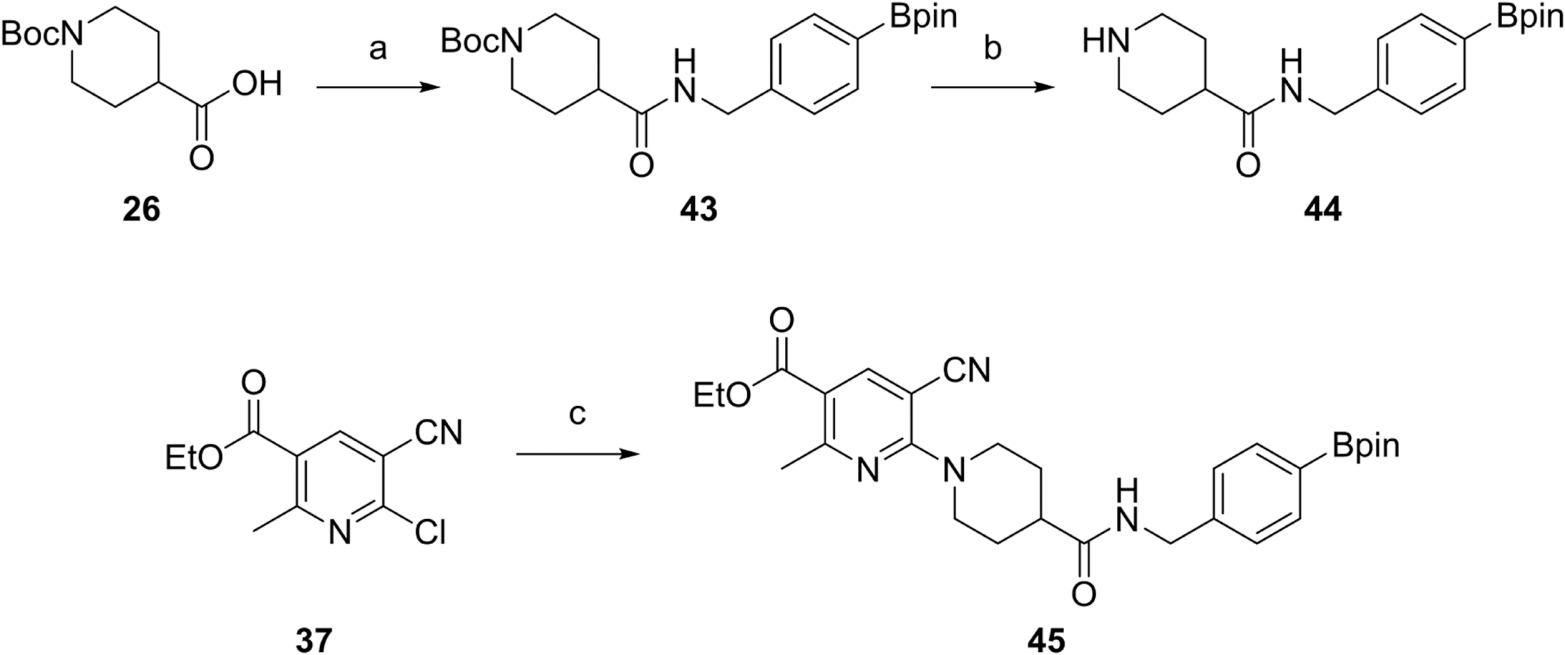
Synthesis of Precursor **45** ^a^

a Reagents and reaction conditions: (a) 4-BpinBn-NH_2_, HBTU, DCC, DIPEA, DCM, rt, 16 h; (b) 4 M HCl, dioxane, rt, 4 h; (c) **44**, TEA, EtOH, 72 °C, 16 h.

### *In Vitro* Competitive Binding Assay

The inhibitory effect of compounds **5**-**22** on the P2Y12R was determined utilizing a competitive radioligand binding assay following a modified protocol by Dupuis et al. with [^33^P]­2-MeSADP, a commercially available P2Y12R radioligand, as competitor (Table 1 and Figure S2).^38^ Compounds **5** and **6** were included to compare IC_50_ values with reported values from literature (Figure 2). Many compounds show a good to moderate affinity for the receptor, with the most potent ligand being AZD1283 **5** (IC_50_ = 51 nM) directly followed by **12** (IC_50_ = 79 nM) and **14** (IC_50_ = 199 nM). Compounds with a nitrile substituent were more affine than the compounds with chloride or hydrogen substituents, which confirms the prediction from previous docking experiments. Compounds **13** and **19** with the highest predicted affinity for the receptor *in silico*, showed the weakest interaction in the binding assay (17,750 and 27,140 nM). Thus, replacing the pyridyl with an aryl was not suitable given the observed reduction in affinity, although a potential interfering contribution of the propoxy group at position 4 has to be taken into account. High docking scores could be achieved by elongating the carbon chain of the 4-fluorbenzyl group in compounds **15**-**19** despite the absence of an additional hydrogen bond acceptor moiety. Compared to the respective homologues **9**–**13**, *in vitro* results could not confirm the *in silico* results. Only **17** displayed a better affinity than its respective counterpart **11** (661 *vs* 1141 nM). While the docking scores for the lactones **8** and **14** were the lowest of the test set, their IC_50_ values (1842 and 199 nM) turned out better than predicted. The piperidinyl ring was found to be superior over the respective piperazine or azetidine alternative. Compounds **9**,**10** and **12** show higher affinities than their respective homologues **6**-**7** and **20**–**22**. Eventually, compound **12** was selected for radiofluorination and further evaluation, because it showed the highest affinity toward P2Y12R and brain accumulation seemed possible based on good CNS MPO and BBB scores (>4).

### Radiochemistry

The radiofluorination followed an adapted protocol developed by Hoffmann and co-workers.^39^ Radioligand [^18^F]**12** was synthesized in an average total synthesis time of 65 min *via* nucleophilic aromatic fluorination of borpinacol ester precursor **45** (Scheme 6). Starting from a [^18^F]­F^–^ activity of 1.1–5.5 GBq, the radiotracer [^18^F]**12** was obtained with an activity yield (AY) of 0.16–1.05 GBq. Based on the amount of [^18^F]­F^–^ trapped on the QMA cartridge, the radiochemical yield (RCY) was 21 ± 9% (*n* = 15) with a radiochemical purity (RCP) of >99% and a molar activity (A_m_) of 36 ± 24 GBq/μmol (*n* = 12).^40^ Representing chromatograms for the semipreparative HPLC purification are shown in the Supporting Information (Figures S3–S5). Tracer identity was confirmed by coinjection of [^18^F]**12** with cold reference compound **12** (Figure S6).

**Scheme 6:**
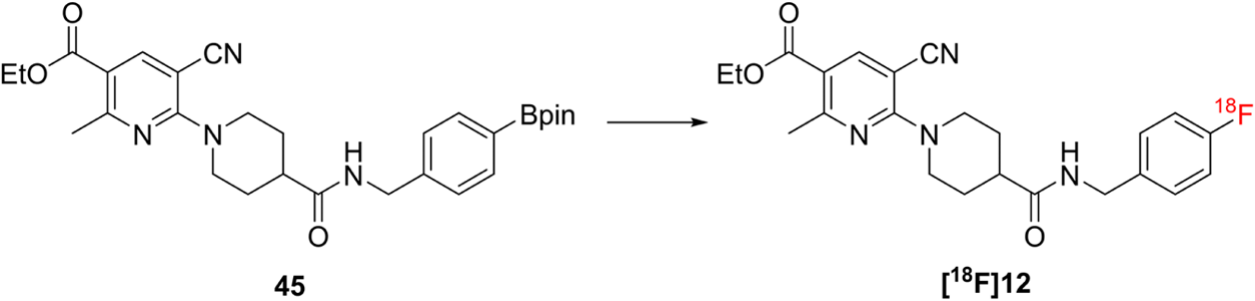
Synthesis of radioligand [^18^F]**12** ^a^

a Reagents and reaction conditions: [^18^F]­F^–^, Cu­(OTf)_2_Py_4_, NBu_4_OTf, DMI, *n*-BuOH, air, 110 °C, 10 min.

### *In Vitro* Stability Tests and Log *D* Determination of [^18^F]­12

To exclude kinetic instability or degradation by proteolysis, the tracer was incubated in PBS buffer, murine blood or human blood at 37 °C for a period of 2 h (Figure S7). Samples were taken at 7 time points (0–120 min) and analyzed by radio-HPLC. PBS and human plasma samples displayed one single peak representing the parent fraction, which remained stable over time. The absence of additional peaks and changes of peak shape indicates that the radioligand remains stable with no decomposition for at least 2 h in PBS formulation and human plasma. However, tracer decomposition was observed in murine plasma with one major polar metabolite accounting for around 60% of total radioactivity after 2 h. The lipophilicity of [^18^F]**12** was determined using the shake flask method between *n*-octanol and PBS buffer (pH 7.4).^29^ The resulting distribution coefficient (log *D*_7.4_ value) was 2.67 ± 0.45 (*n* = 16), indicating a moderate lipophilicity. This value is within the desired range for brain radiotracers (log *D*_7.4_ = 2–3.5).^41^

### PET Imaging and Biodistribution

Dynamic *in vivo* PET imaging was carried out in WT mice (*n* = 8) in order to assess the tracer distribution and brain uptake for [^18^F]**12** (Figure 4A). An initial peak of activity and subsequent rapid washout in blood and lungs was observed after tracer injection. The activity then predominantly accumulated in the liver, kidneys, bladder and intestines (Figure 4B). Elevated bone uptake indicating radio-defluorination *in vivo*, was not detected. The brain time-activity-curve (TAC) demonstrated that [^18^F]**12** successfully crossed the BBB with a maximum brain uptake *C*_max_ of 2.5 ± 0.7 SUV within the first 2 min p.i., followed by a rapid tracer clearance (Figure 4C). 25 min after tracer administration, the activity signal reached a plateau phase with a slow decrease in signal intensity (*C*_25min_/*C*_60min_ ∼ 1.5). Dynamic *in vivo* PET imaging was repeated with WT mice (*n* = 2) under self-block conditions. A 200-fold excess of nonradioactive compound **12** was injected 10 min prior to radiotracer administration (Figure 4C). Brain TAC analysis revealed increased brain uptake during both the initial uptake phase and the subsequent plateau phase under self-block conditions. Mouse organs were collected 60 min p.i. after intracardial perfusion to quantify the tracer uptake *ex vivo*. [^18^F]**12** still exhibits an activity level of 0.6 ± 0.2%ID/g in the brain, demonstrating that a substantial amount of tracer remained in the CNS after the PET scan (Figure 4D and Table S2). In blood samples tracer quantification resulted in an activity level of 1.2 ± 0.6%ID/g. Similar to [^11^C]­AZD1283, [^18^F]**12** displays only a low retention in blood, which is noteworthy given that P2Y12R is expressed on blood platelets.^27^ The highest signals of tracer uptake were measured in metabolic and excretory organs such as the liver with 19.6 ± 4.8%ID/g and kidney 5.5 ± 1.1%ID/g.

**Figure 4:**
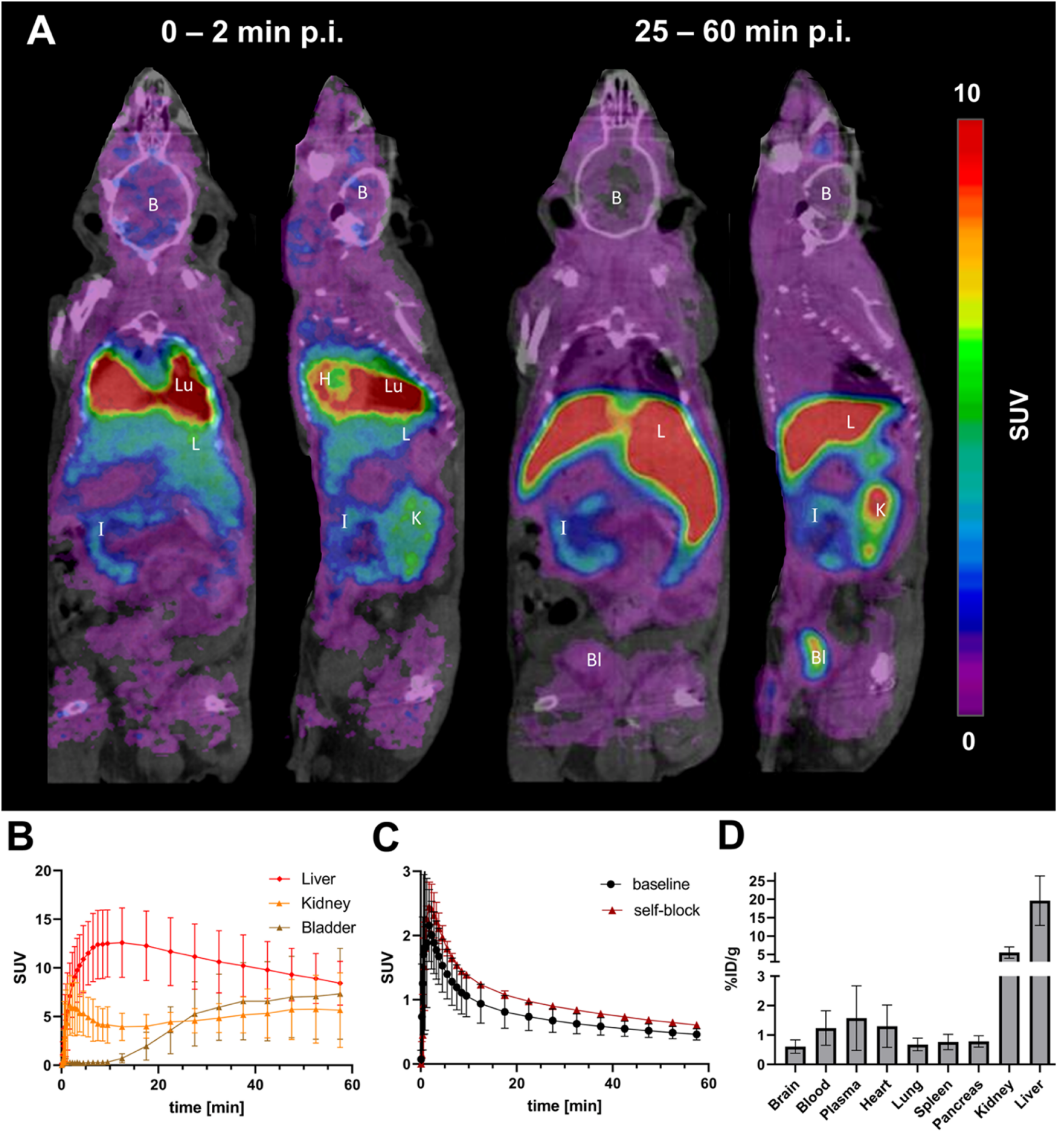
(A) Representative average PET/CT images of [^18^F]**12** in a WT mouse 0–2 min and 25–60 min p.i.; B, brain; Bl, bladder; H, heart; I, intestine; K, kidney; L, liver; Lu, lung. (B) Average liver, kidney and bladder TACs and (C) Average whole brain TAC of [^18^F]**12** in WT mice under baseline and blocking conditions. (D) *Ex vivo* biodistribution including brain and blood (*n* = 12), plasma, heart, lung, spleen, pancreas, kidney and liver (*n* = 2).

As *in vivo* blocking experiments except self-block were not possible due to the unavailability of brain permeable P2Y12R inhibitors, microglia-depleted mice were used as negative controls. The feeding of the colony-stimulating factor-1 receptor (CSF1R) antagonist PLX-5622 to wild-type mice results in a near-total depletion of microglia with reduced P2Y12R expression after a three-week period of a chow diet.^42–44^ Dynamic PET imaging was carried out in microglia-depleted (PLX) mice (*n* = 8) and whole brain TACs were generated (Figure 5A,B). The initial peak tracer uptake was lower (*C*_max_ = 2.0 ± 0.5 SUV) compared to WT mice (*C*_max_ = 2.5 ± 0.7 SUV), and the later plateau phase indicated reduced tracer retention in the CNS. This effect was most significant for the time period 25–60 min p.i. (42% higher accumulation in WT, *p* = 0.0029, Figure 5C) and is further illustrated in the differential SUV scaled image comparing WT and PLX treated mice.

**Figure 5:**
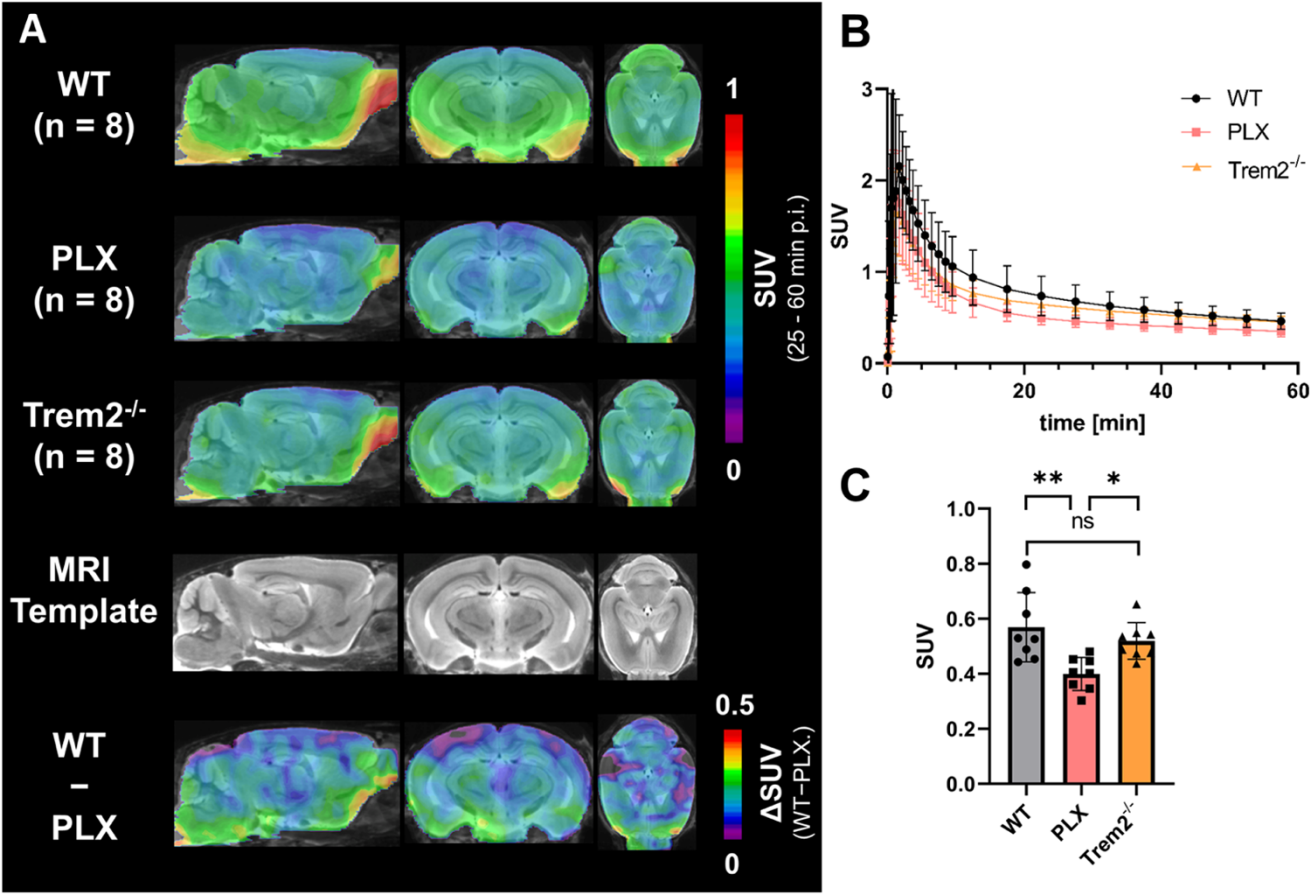
(A) Average PET/CT brain image of [^18^F]**12** in WT, PLX and Trem2^–/–^ mice (*n* = 8), 25–60 min p.i. with MRI template overlay, and differential image WT–PLX; whole body PET/CT images are shown in Figure S8. (B) Grouped whole-brain TACs for WT, PLX treated and Trem2^–/–^ mice. (C) Tracer quantification in whole brain, 25–60 min p.i. in WT, PLX treated and Trem2^–/–^ mice. One-way ANOVA/Tukey’s multiple comparisons test (*F* = 7.630, *p* = 0.0032), *p* > 0.05 (ns), *p* ≤ 0.05 (*), *p* ≤ 0.01 (**), mean ± SD.

Based on reports demonstrating higher levels of P2Y12R positive microglia in Trem2^–/–^ mice, we anticipated a higher tracer uptake due to increased target expression in these mice.^45,46^ TACs from dynamic PET imaging in Trem2^–/–^ mice (*n* = 8) revealed the lowest tracer uptake in comparison to WT and microglia-depleted mice during the initial phase (*C*_max_ = 1.7 ± 0.5 SUV), whereas the later plateau phase was characterized by elevated tracer retention in the Trem2^–/–^ mouse model compared to PLX mice. This effect is significant 25–60 min p.i. (30% higher accumulation in Trem2^–/–^, *p* = 0.0362). However, no significant difference was observed comparing WT and Trem2^–/–^ mice.

To exclude a potential bias in brain PET interpretation caused by varying blood levels, volume-of-distribution (*V*_T_) images were computed using an image-derived cardiac input function. Brain *V*_T_ values confirmed group differences of tracer uptake, which was increased in WT (16%; *p* = 0.002) and Trem2^–/–^ mice (10%; *p* = 0.0454) compared to PLX mice (Figure S9). Cardiac ventricle TACs used for *V*_T_ calculation exhibited similar shapes for all groups (Figure S10). SUV results were also controlled for a potential influence by the mass-dose effect, but no correlation between injected tracer amounts and PET SUV values was detected (Figure S11).

### Correlation of PET Signal with Immunohistochemistry Results

*Ex vivo* immunohistochemical evaluation of Iba1 and P2Y12R expression was performed for a subset of mice (4 WT, 4 PLX, 8 Trem2^–/–^) to correlate P2Y12 PET signal with target expression. While WT mice exhibited a high level of P2Y12R-positive microglia in cortical sections, Iba1 and P2Y12R were almost completely absent in PLX mice (Figures 6A, and S12B,C). High levels of Iba1 and P2Y12R were also detectable in Trem2^–/–^ mice, but reflecting the results of the PET studies, P2Y12R levels were not elevated compared to WT mice. Cortical SUV levels, derived from PET imaging in the most significant time frame (25–60 min p.i.) (Figure S12D), were correlated with Iba1 or P2Y12R expression density. A strong association between Iba1 signal and tracer uptake (*R* = 0.6589, *p* = 0.0055, Figure 6B) and between the P2Y12R signal and tracer uptake (*R* = 0.754, *p* = 0.0007, Figure 6C) was observed. The P2Y12 signal was well associated with Iba1 (*R* = 0.6, *p* = 0.014, Figure 6D) indicating predominant microglial receptor visualization.

**Figure 6:**
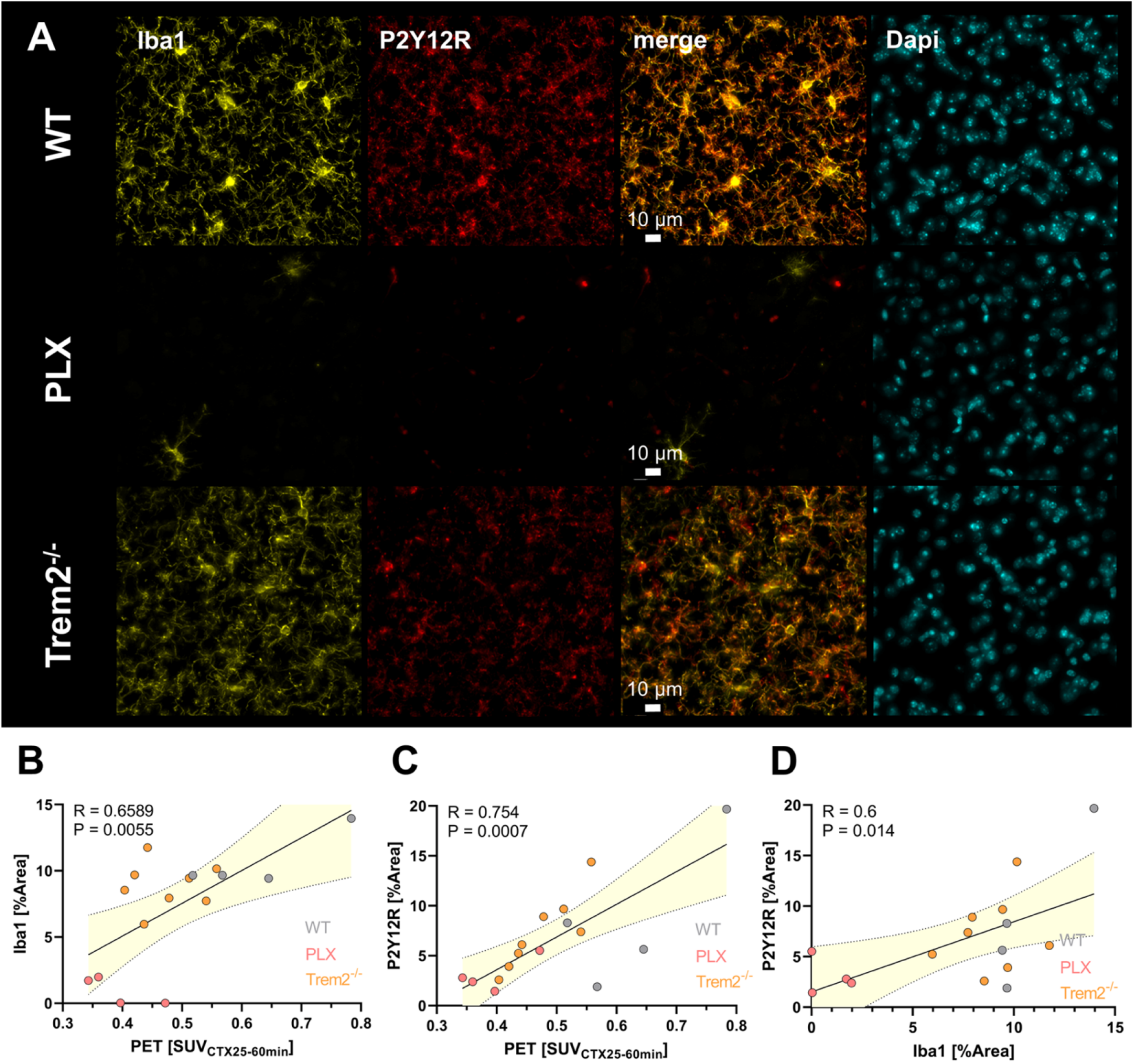
(A) Iba1, P2Y12R and Dapi immunofluorescence staining in WT, PLX and Trem2^–/–^ mouse cerebral cortex, scale bar = 10 μm. Correlation of the (B) cortical Iba1 and (C) cortical P2Y12R immunofluorescence signal (%Area) with cortical PET SUV_CTX25–60min_ for [^18^F]**12**. (D) Correlation of the cortical Iba1 with P2Y12R immunofluorescence signal (%Area). Linear regression, α = 0.05, 95% CI.

### *In Vitro* ARG

*In vitro* ARG experiments on brain sections from all animal models were performed under baseline and blocking conditions. Brain sections were coincubated with [^18^F]**12** and a 1000-fold excess of ticagrelor, a potent and structurally nonrelated P2Y12R antagonist or nonradioactive compound **12** (Figure 7A). Tracer signal of unblocked [^18^F]**12** displayed a clear and uniform pattern. This aligns with the anticipated homogeneous distribution of homeostatic microglia in healthy WT mice observed in immunofluorescence staining with an antimouse P2Y12R antibody in adjacent brain sections (Figure S12A) and previous ARG studies with P2Y12R ligand **2**.^25,28^ Quantification of cortical brain signal revealed similar levels in WT, Trem2^–/–^ and PLX sections (Figure 7B). However, under blocking conditions with Ticagrelor, the signal was significantly reduced in WT (*p* < 0.001) and Trem2^–/–^ (*p* < 0.05) sections, whereas no reduction was observed in PLX sections (*p* > 0.05), which can be explained by the absence of target receptor in PLX mice. However, as already concluded from our PET data (Figure 5), the high signal in PLX sections may reflect a substantial amount of nonspecific (NSB) or off-target binding. Blocking with nonradioactive compound **12** led to an almost complete blocking of the signal in all three groups. Comparing the cortex (CTX) signal normalized to the signal in the hypothalamus (HYP) revealed a significant decreased uptake in PLX sections compared to the other groups (WT: *p* < 0.005; Trem2^–/–^: *p* < 0.001; Figure 7C,D).

**Figure 7:**
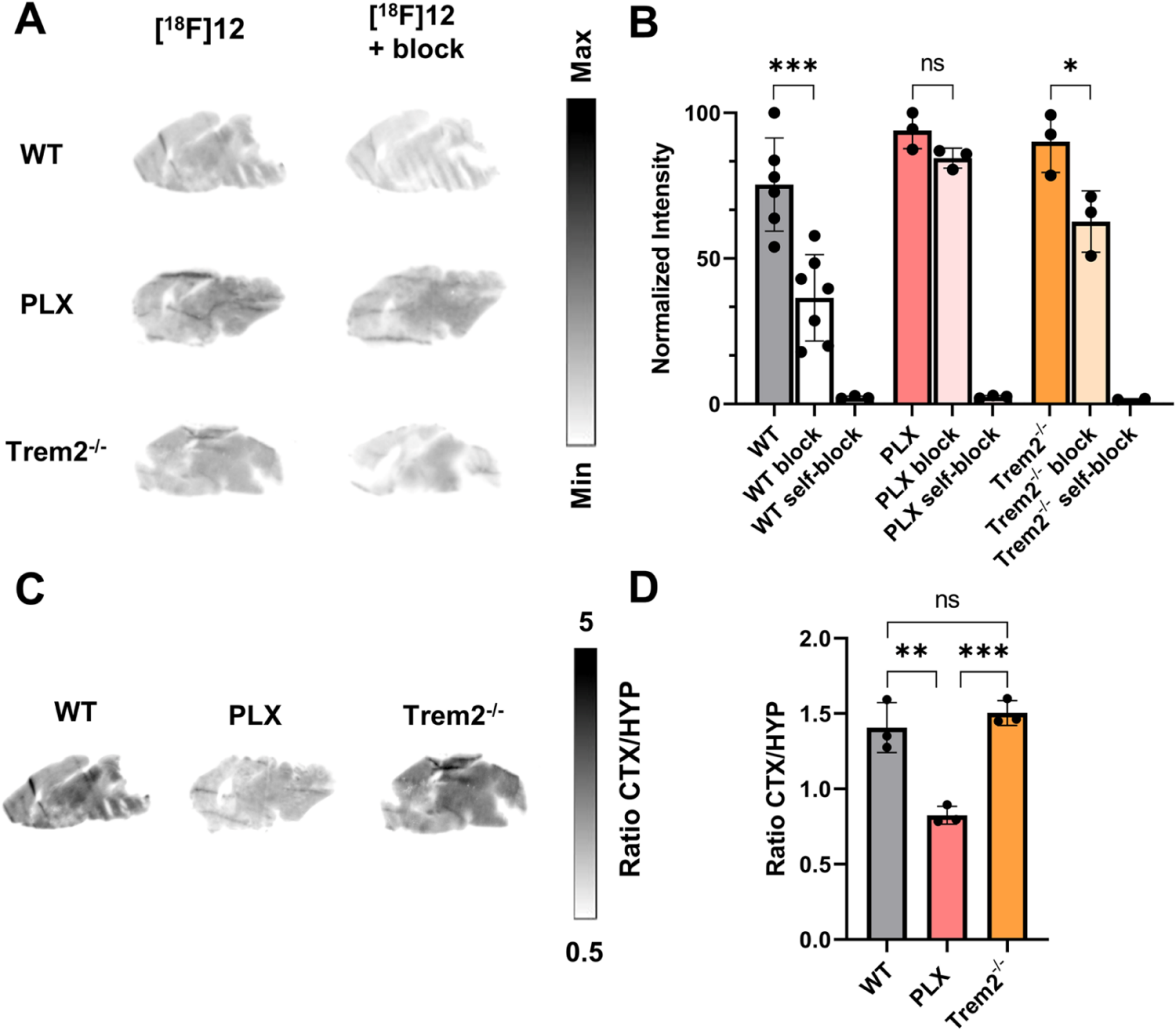
(A) *In vitro* autoradiography of WT, PLX and Trem2^–/–^ sections with [^18^F]**12** at baseline and blocking conditions (1 μM Ticagrelor); (B) quantification of cortical brain signal of [^18^F]**12** in WT (baseline *n* = 6, blocking *n* = 7 and self-block conditions *n* = 3), PLX (*n* = 3 each) and Trem2^–/–^ (*n* = 3 each) sections; one-way ANOVA/Tukey’s multiple comparisons test (*F* = 28.72, *p* < 0.0001); (C) *in vitro* autoradiography of normalized WT, PLX and Trem2^–/–^ sections representing CTX/HYP ratios; (D) quantification of CTX/HYP ratios (*n* = 3 each); one-way ANOVA/Tukey’s multiple comparisons test (*F* = 32.13, *p* = 0.0006), *p* > 0.05 (ns), *p* ≤ 0.05 (*), *p* ≤ 0.01 (**), *p* ≤ 0.001 (***), mean ± SD.

### *Ex Vivo* Metabolite Analysis in Murine Plasma

The metabolic profile of compound [^18^F]**12** was assessed in plasma, brain and liver samples 60 min p.i. The radio-HPLC analysis of WT plasma samples revealed that the parent fraction was reduced to about 11% intact tracer 60 min p.i. (Figure 8). Two new metabolites, [^18^F]**M1** (3%) and [^18^F]**M2** (86%), were formed. Liver samples contained equal ratios of intact tracer [^18^F]**12** (47%) and [^18^F]**M2** (43%) and no [^18^F]**M1**. The radio-HPLC analysis of plasma samples displayed comparable metabolic degradation (7 ± 4% intact tracer after 60 min p.i.) in WT, PLX and Trem2^–/–^ mice (Figure S13A) and radio-HPLC analysis of brain samples confirmed the exclusive presence of intact [^18^F]**12** (>99%) in the CNS 60 min p.i. in all groups. (Figures 8 and S13B).

**Figure 8:**
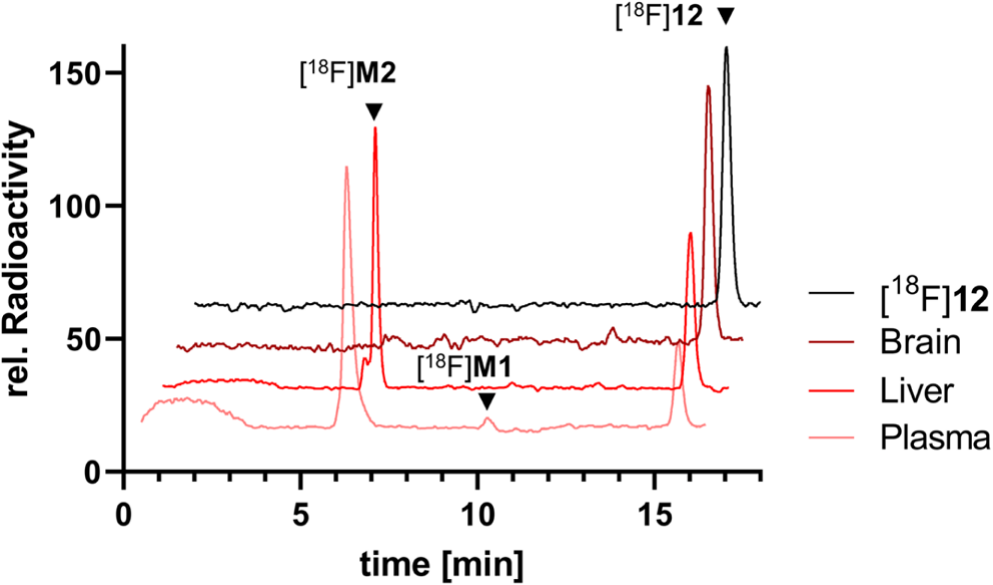
Representative radio-HPLC chromatograms of plasma, liver and brain samples from WT mice 60 min p.i. and [^18^F]**12** as a reference standard.

## Discussion

In recent years, P2Y12R has emerged as a promising new imaging target in the context of neurodegenerative diseases. *In vivo* imaging of the receptor by PET offers the opportunity to determine microglia activation based on the level of P2Y12R-positive homeostatic microglia. The observed shift toward activated P2Y12-negative microglia in neurodegenerative diseases such as AD in their early stages has significant implications for diagnosis, monitoring and therapeutic intervention assessment. However, the availability of a highly selective and specific PET tracer with optimal physicochemical properties for brain uptake and clearance is a prerequisite, given the gradual decrease of target throughout disease progression. Although previous attempts with compounds **1**-**5** have yielded promising *in vitro* results as potent P2Y12R inhibitors, they only exhibited limited brain uptake, thus rendering PET imaging P2Y12R in the context of neuroinflammation in the CNS impossible.^25–29^ The objective of this study was to identify a AZD1283 derived compound in order to facilitate the BBB passage while maintaining a sufficient degree of affinity for the target receptor.

It was previously hypothesized that the sulphonamide moiety was the primary factor preventing the penetration of compounds **3** and **4** through the BBB due to the high MW and TPSA of the negatively charged species *in vivo*.^27,29^ Accordingly, we sought to replace this group with a less polar carbon-based scaffold despite its positive impact on receptor affinity. The crystal structure and interaction diagram also revealed a relatively large space without significant ligand–receptor interactions around the amino linker, which could be used to further adapt the physicochemical properties by incorporating unipolar structures without affecting the affinity too much. In the binding assay, compound **12** exhibited the highest affinity from all structures tested, with a nanomolar IC_50_ value that was comparable to that of AZD1283. Predictions from the CNS MPO and BBB scores also indicated brain permeability. Thus, candidate **12** was selected, radiolabeled and used for further evaluation. TACs from PET analysis and brain radioactivity measured by *ex vivo* biodistribution indicated the anticipated brain uptake in WT mice which could be unambiguously confirmed by identification of intact tracer in the brain by *ex vivo* radio-HPLC. Hence, [^18^F]**12** is able to cross the BBB and has a favorable kinetic profile for neuroimaging. Further, *ex vivo* radio-HPLC revealed no radio-metabolites in the brain 60 min p.i., whereas in plasma, the fraction of intact tracer was strongly reduced indicative of a substantial tracer metabolization. This metabolization was already observed *in vitro*, in contrast to the high stability observed in human blood and PBS. The results suggest that the tracer is a substrate of carboxylesterase, which is about 30-fold less active in human blood.^47^ The strong increase of polarity of the metabolite also hints toward esterase-catalyzed hydrolysis of the ester group to the corresponding carboxylic acid. AZD1283 was stopped in clinical studies, due to concerns about the metabolic stability of the ester group.^48^ In general, rapid tracer metabolism is undesirable, since it reduces the bioavailability of the intact tracer and generates radio-metabolites with an unknown pharmacokinetic profile. However, polar metabolites also have a decreased probability of brain uptake. Interestingly, Jackson et al. did not detect any metabolites derived from [^11^C]­AZD1283 in blood and liver 30 min p.i.^27^ This indicates that the replacement of the sulphonamide in [^18^F]**12** has an impact on the metabolic stability of the radiotracer *in vivo*. An interesting aspect is the low binding of the tracer to peripheral P2Y12R, which is predominantly expressed on blood platelets. The high metabolic rate of the tracer may explain low peripheral P2Y12R binding assuming ester cleavage as primary mechanism, because the resulting carboxylic acid showed a dramatically reduced target affinity for the P2Y12R.^48,49^ The fact that the tracer appears to be substantially metabolized *in vitro* presents a nontrivial problem for assays that rely on murine blood samples to determine P2Y12R specificity. Moreover, radio-HPLC only represents the metabolite distribution in platelet-poor plasma, and the binding profile of the metabolites is currently unknown. Tracer uptake in the liver and the kidneys suggested renal excretion as the primary mechanism of tracer elimination. PET data indicated also uptake in the intestines, which suggests that hepatobiliary elimination may also be a contributing factor. In order to assess specificity of the PET tracer *in vivo*, blocking studies are typically carried out. This currently remains unfeasible for the P2Y12R due to the unavailability of alternative brain-permeable P2Y12R inhibitors, and intracisterna magna administration of P2Y12R inhibitors represents a rather invasive intervention. Self-block with nonradioactive compound **12** resulted in an increased tracer uptake in the brain, rather than the expected blocking effect. This circumstance has been previously reported, *e.g.*, Gundam et al. described increased brain uptake of a radioligand when coadministered with the nonradioactive compound, which they attributed to peripheral effects such as reduced hepatic metabolism and increased systemic availability of the tracer.^50^ Similarly, Stéen et al. observed a higher brain uptake under blocking conditions in PET imaging compared to baseline, which they explained by increased plasma availability and possible changes in the tracer’s metabolic fate or plasma protein binding.^51^ In line with these reports, our own tracer stability assays in murine blood confirmed that the compound is subject to metabolic degradation, suggesting that peripheral metabolism plays a significant role for brain tracer availability, especially since the main metabolite does not cross the blood brain barrier. PLX treated mice were used as negative control, exhibiting an almost complete reduction in P2Y12R expression as a consequence of microglia depletion, which was confirmed by IHC. In addition to WT mice, Trem2^–/–^ mice were used based on reports of elevated expression of homeostatic genes and increased P2Y12R density.^45^ A significant difference in tracer uptake was observed in PET between microglia-depleted PLX mice and WT mice, as well as Trem2^–/–^ controls, which is in accordance with IHC results, showing higher microglia and P2Y12R levels in cortical sections of the WT and Trem2^–/–^ control group. But, PLX treated mice also exhibited substantial tracer retention in the brain, which may be indicative for microglia- and P2Y12R-independent nonspecific binding in the brain parenchyma. Also, no significant difference in brain signal was detected between WT and Trem2^–/–^ mice. The *ex vivo* Iba1 or P2Y12R IHC signal was correlated with the *in vivo* PET signal in the cortex and revealed a statistically significant dependency. This correlation analysis supports the conclusion that the PET signal reflects microglial P2Y12R expression levels and that the PET signal can be attributed to tracer binding to P2Y12R on microglia. PET image interpretation was critically reviewed to exclude confounding factors such as an altered metabolism, different mass doses or blood tracer levels in the cohorts. We did not find any indication that the difference in brain uptake is driven by one of these factors. It is likely that differences in peak tracer uptake also reflect P2Y12R availability in WT and PLX mice. Similar levels of tracer uptake in Trem2^–/–^ compared to WT mice contrasts reports by Götzl et al.^45^ showing increased P2Y12 expression in Trem2^–/–^ mice. This discrepancy could potentially be attributed to the different age of the animals used in the work by Götzl et al. (9 months) and in our study (17 months) considering an overall decline of microglia, and thus P2Y12R, during the lifespan of WT and Trem2^–/–^ mice.^52^ But, existing literature also shows contradictory findings regarding P2Y12R levels in younger mice.^53,54^ Previous studies have also demonstrated that Trem2 loss-of-function may lead to a reduced cerebral blood flow as a result of decline in brain function and metabolism, which might also impact time-activity characteristics.^55^ *In vitro* ARG blocking studies with ticagrelor demonstrated specificity for P2Y12R in WT and Trem2^–/–^ sections, but nonspecific binding cannot be excluded. Recently, glycogen synthase kinase 3 α (GSK3α) was identified as a potential off-target of a structurally related P2Y12R radioligand.^26^ Quantitative ARG results align with the PET observations and indicate that the brain uptake reflects microglial P2Y12R expression and is not biased from perfusion, metabolism or clearance.

Most recently, Yao et al. reported a new series of brain-permeable P2Y12 tracers closely related to [^18^F]**12**.^56^ Both studies independently demonstrate the high potential of nicotinates as P2Y12R ligands for PET imaging. However, further modification of [^18^F]**12** will be necessary to reduce NSB and increase stability. Higher molar activities could improve sensitivity, especially in cases of low target levels.

## Conclusions

[^18^F]**12** represents a novel brain penetrant P2Y12R targeting radiotracer, which is a significant advancement in the field of PET imaging of P2Y12R in the CNS, with great potential for further *in vivo* applications in the field of neuroinflammation and neurodegenerative diseases.

## Experimental Section

### General Information

Starting materials and solvents were purchased from commercial sources and were used without further purification. AZD1283 was purchased from Sigma-Aldrich (SML2080). If required, reactions were carried out under positive N_2_ atmosphere in flame-dried glassware. Syringes were purged with nitrogen prior to use for moisture sensitive and anhydrous reagents. All reactions were monitored by thin-layer chromatography (TLC) using Merck silica gel 60 F_254_ aluminum plates. The spots were visualized under UV (254 nm) or by staining the TLC plate with KMnO_4_ solution (K_2_CO_3_, 10 g KMnO_4_, 1.5 g H_2_O, 150 mL 10% NaOH in H_2_O, 1.25 mL). The TLC plate was heated with a heat gun if necessary. Chromatography purifications were performed on silica gel (SiO_2_, 0.040–0.063 mm, 230–400 mesh ASTM) from Merck. Celite 545 (0.02–0.1 mm particle size) was purchased from Merck. ^13^C and ^1^H NMR spectra were recorded on BRUKER AMX 600 instruments. Chemical shifts are reported as δ values in parts per million (ppm) relative to the respective residual solvent peak (δ ^1^H NMR: CDCl_3_ 7.26, DMSO-*d*_6_ 2.50; ^13^C NMR: CDCl_3_ 77.16, DMSO-*d*_6_ 39.52). Coupling constants J are reported in hertz (Hz). Following abbreviations or combinations of them are used for signal coupling: s (singlet), d (doublet), t (triplet), q (quartet), quint (quintet), m (multiplet). Spectra were processed using MestreNova software (12.0.2). High resolution mass spectra (HRMS) were recorded on a Finnigan MAT 95Q or Finnigan MAT 90 or JEOL JMS-700 instrument. Yields refer to isolated compounds with satisfying purity, which was determined by ^1^H NMR. Quality control of selected organic compounds was performed by HPLC (Agilent Technologies, 1200 series). All compounds used for *in vitro* and *in vivo* testing are >95% pure by HPLC analysis. Test compounds were controlled for potential assay interference activity with SwissADME.^57^ Animal handling and experiments in this study were performed under the supervision of a veterinarian in accordance with the German Animal Welfare Law and were approved by the Government of Upper Bavaria, Germany (Vet_02–21–156, Vet_03–22–25). Blood samples used in our stability assays were obtained from a healthy voluntary donor (one of the experimenters). The study was approved by the local ethics committee of the medical faculty of the LMU Munich (Application Number: 21–0721) All participants gave full informed consent.

### Molecular Design and Docking

Crystal structure analysis and modeling experiments were conducted using MOE (2022.02). The P2Y12R protein structure file (PDB code 4NTJ) was prepared using the Quickprep function to add hydrogen atoms, missing residues and partial charges to the protein structure. A pharmacophore with 6 features for the active binding site was defined based on structural analysis of key interactions reported in the literature (Figure S1).^18,58^ Ligands were required to match at least one of these features. New ligand structures were generated by altering the resident AZD1283 ligand in the 4NTJ crystal structure through the scaffold replacement tool (Filter: MW < 500, TPSA [40–120]). Taking into account synthetic accessibility, the most promising compounds were selected for further docking studies together with literature known scaffolds.^30,31,36,49^ 30 conformations were placed for every compound by a pharmacophore docking algorithm (Scoring by London dG function) followed by a refinement step using the induced fit selection for 5 poses (Scoring by GBVI/WSA dG function). The best-docked pose for every ligand was ranked based on their final S score. Prediction scores BBB and CNS MPO for brain uptake for each entry were calculated using MarvinSketch (21.16) and Marvin JS (23.11.0) by ChemAxon.

### Chemical Synthesis

#### General Procedure 1 (GP1): Amide Bond Formation

Carboxylic acid (1.0 equiv), DIPEA (1.2 equiv), DCC (1.05 equiv) and HBTU (1.03 equiv), were solved in dry DCM (10 mL). The solution was cooled down to 0 °C and the corresponding amine (1.0 equiv) was added dropwise. The solution was stirred at rt for 16 h. Upon completion of the reaction, the reaction mixture was filtered through Celite and the filter cake was rinsed with DCM (2 × 10 mL). The combined organic layer was washed with saturated NaHCO_3_ (2 × 10 mL) solution and H_2_O (1 × 10 mL), dried over MgSO_4_ and evaporated *in vacuo*. The crude product was purified by column chromatography on silica gel to yield the corresponding amide.

#### General Procedure 2 (GP2): Boc Deprotection

Boc-protected amine (1.0 equiv) was solved in dry dioxane (5 mL) and the solution was cooled down to 0 °C. 4 M HCl in dioxane (4 equiv) was added dropwise and the reaction was stirred at rt for 6 h. The resulting suspension was filtered and washed with cold dioxane (3 × 10 mL) to afford the unprotected amine as white solid without further purification.

#### General Procedure 3 (GP3): Synthesis of 6-Aminonicotinate **6**–**12**, **14**–**18**, **20**–**22**

6-Chloronicotinate (1.0 equiv), TEA (3.0 equiv) and the corresponding amine **25** or **31–33** (1.1 equiv) were dissolved in dry EtOH (2 mL). The reaction mixture was stirred at 80 °C for 24 h. Upon completion of the reaction, the solvent was removed *in vacuo.* The residue was resolved in water (10 mL) and the aqueous phase was extracted with ethyl acetate (3 × 10 mL). The combined organic layer was washed with brine (20 mL), dried over MgSO_4_ and evaporated *in vacuo*. The crude product was purified by column chromatography on silica gel to give the corresponding 6-aminonicotinate.

#### General Procedure 4 (GP4): Synthesis of 6-Aminobenzoate **13** and **19**

Propoxybenzoate **42** (1.0 equiv), K_2_CO_3_ (1.5 equiv) and the corresponding amine **31** or **32** (1.2 equiv) were dissolved in dry DMF (2 mL). The reaction mixture was stirred at 120 °C for 24 h. Upon completion of the reaction, water (10 mL) was added and the aqueous phase was extracted with ethyl acetate (3 × 10 mL). The combined organic layer was washed with brine (20 mL), dried over MgSO_4_ and evaporated *in vacuo*. The crude product was purified by column chromatography on silica gel to give the corresponding 6-aminoaryl.

##### *tert*-Butyl Piperazine-1-carboxylate (**23**)

To a solution of piperazine (861 mg, 10.0 mmol, 1.0 equiv) in dry MeOH (10 mL) was added dropwise Boc_2_O (1.71 mL, 8 mmol, 0.8 equiv) at 0 °C and the solution was stirred for 5 h at rt. The reaction mixture was concentrated *in vacuo* and the residue was diluted with water (10 mL). The water phase was extracted with EtOAc (3 × 10 mL) and the combined organic layer was washed with saturated NaHCO_3_ solution (10 mL), dried over MgSO_4_ and evaporated *in vacuo*. The crude product was purified by column chromatography on silica gel to give **23** (910 mg, 4.9 mmol, 61%) as colorless crystals. ^1^H NMR (400 MHz, CDCl_3_): δ = 3.39–3.31 (m, 4H), 2.82–2.71 (m, 4H), 2.19 (s, 1H), 1.42 (s, 9H). ^13^C NMR (100 MHz, CDCl_3_) δ 154.9, 79.6, 45.9, 44.8, 28.5. HRMS (ESI): [M + H^+^, C_9_H_19_N_2_O_2_^+^], calculated: 187.1441, found: 187.1441.

##### *tert*-Butyl 4-((4-Fluorobenzyl)­carbamoyl)­piperazine-1-carboxylate (**24**)

To a solution of Boc-protected piperazine **23** (275 mg, 1.5 mmol, 1 equiv) in dry DCM (3 mL) was added dropwise 1-fluoro-4 (isocyanatomethyl)­benzene (227 mg, 1.5 mmol, 1 equiv) in DCM (3 mL) and the solution was stirred for 16 h at rt. After the completion of the reaction, the organic phase was washed with brine (10 mL), dried over MgSO_4_ and evaporated *in vacuo*. The crude product was purified by column chromatography on silica gel to give **24** (153 mg, 0.5 mmol, 30%) as colorless crystals. ^1^H NMR (400 MHz, CDCl_3_): δ = 7.28–7.21 (m, 3H), 7.02–6.95 (m, 2H), 4.94 (t, *J* = 5.5 Hz, 1H), 4.36 (d, *J* = 5.5 Hz, 2H), 3.44–3.30 (m, 8H), 1.45 (s, 9H). ^13^C NMR (100 MHz, CDCl_3_): δ = 157.5, 154.7, 135.2 (d, *J* = 3.2 Hz), 129.5 (d, *J* = 8.0 Hz), 115.5 (d, *J* = 21.4 Hz), 80.3, 44.3, 43.7, 28.5. HRMS (ESI): [M + Na^+^, C_17_H_24_N_3_O_3_FNa^+^], calculated: 360.1694, found: 360.1693.

##### *N*-(4-Fluorobenzyl) Piperazine-1-carboxamide (**25**)

Boc-protected amine **24 (**120 mg, 0.36 mmol) was used in GP2 to afford amine **25** as colorless crystals (62 mg, 0.26 mmol, 74%). ^**1**^H NMR (400 MHz, D_2_O): δ = 7.36–7.28 (m, 2H), 7.16–7.07 (m, 2H), 4.33 (s, 2H), 3.72–3.66 (m, 4H), 3.31–3.25 (m, 4H). ^13^C NMR (100 MHz, D_2_O): δ = 161.7 (d, *J* = 242.3 Hz), 158.7, 135.2 (d, *J* = 3.0 Hz), 128.7 (d, *J* = 8.3 Hz), 115.2 (d, *J* = 21.5 Hz), 43.3, 42.9, 40.6. HRMS (ESI): [M + H^+^, C_12_H_17_NO_3_F^+^], calculated: 238.1350, found: 238.1347.

##### *tert*-Butyl 4-((4-Fluorobenzyl)­carbamoyl)­piperidine-1-carboxylate (**28**)

Carboxylic acid **26 (**1.15 g, 5 mmol) was used in GP1 with 4-fluorbenzylamine (625 mg, 5 mmol) to afford **28** as colorless crystals (1.45 g, 4.3 mmol, 86%). ^**1**^H NMR (400 MHz, CDCl_3_): δ = 7.15–7.07 (m, 2H), 7.01–6.92 (m, 2H), 5.78 (s, 1H), 4.09 (dt, *J* = 13.5, 3.2 Hz, 2H), 3.46 (q, *J* = 6.2 Hz, 2H), 2.77 (t, *J* = 6.9 Hz, 2H), 2.68 (td, *J* = 13.1, 2.6 Hz, 2H), 2.23–2.11 (m, 1H), 1.76–1.67 (m, 2H), 1.63–1.49 (m, 2H), 1.43 (s, 9H). ^13^C NMR (100 MHz, CDCl_3_): δ = 174.60, 161.7 (d, *J* = 244.6 Hz), 154.7, 134.5 (d, *J* = 3.3 Hz), 130.2 (d, *J* = 7.9 Hz), 115.5 (d, *J* = 21.3 Hz), 79.7, 43.3, 40.7, 34.9, 28.7, 28.5. HRMS (ESI): [M + Na^+^, C_18_H_25_N_2_O_3_FNa^+^], calculated: 359.1741, found: 359.1740.

##### *tert*-Butyl 4-((4-Fluorophenethyl)­carbamoyl)­piperidine-1-carboxylate (**29**)

Carboxylic acid **26 (**688 mg, 3 mmol) was used in GP1 with 4-fluorophenethylamine (575 mg, 3 mmol) to afford **29** as colorless crystals (501 mg, 1.4 mmol, 48%). ^1^H NMR (400 MHz, CDCl_3_): δ = 7.24–7.16 (m, 2H), 6.98 (t, *J* = 8.7 Hz, 2H), 6.01 (t, *J* = 5.7 Hz, 1H), 4.37 (d, *J* = 5.8 Hz, 2H), 4.11 (s, 2H), 2.83–2.59 (m, 2H), 2.24 (tt, *J* = 11.6, 3.8 Hz, 1H), 1.86–1.73 (m, 2H), 1.63 (qd, *J* = 12.3, 4.6 Hz, 2H), 1.43 (s, 9H). ^13^C NMR (100 MHz, CDCl_3_): δ = 174.4, 162.3 (d, *J* = 245.8 Hz), 154.8, 134.2 (d, *J* = 3.2 Hz), 129.5 (d, *J* = 8.0 Hz), 115.7 (d, *J* = 21.5 Hz), 79.8, 43.4–43.3 (m), 43.1, 42.8, 28.7, 28.5. HRMS (ESI): [M + Na^+^, C_19_H_27_N_2_O_3_FNa^+^], calculated: 373.1898, found: 373.1909.

##### *tert*-Butyl 3-((4-Fluorobenzyl)­carbamoyl)­azetidine-1-carboxylate (**30**)

Carboxylic acid **27 (**1.01 g, 5 mmol) was used in GP1 with 4-fluorbenzylamine (625 mg, 5 mmol) to afford **30** as colorless crystals (1.24 g, 0.9 mmol, 80%). ^1^H NMR (400 MHz, CDCl_3_): δ = 7.24–7.16 (m, 2H), 7.02–6.94 (m, 2H), 6.39 (d, *J* = 6.0 Hz, 1H), 4.37 (d, *J* = 5.8 Hz, 2H), 4.06 (d, *J* = 7.4 Hz, 2H), 3.98 (t, *J* = 8.5 Hz, 2H), 3.18 (tt, *J* = 8.6, 6.0 Hz, 1H), 1.40 (s, 9H). ^13^C NMR (100 MHz, CDCl_3_): δ = 171.7, 162.3 (d, *J* = 245.9 Hz), 156.3, 133.9 (d, *J* = 3.2 Hz), 129.6 (d, *J* = 8.1 Hz), 115.7 (d, *J* = 21.5 Hz), 79.9, 51.8, 43.1, 33.3, 28.4. HRMS (ESI): [M + Na^+^, C_16_H_21_N_2_O_3_FNa^+^], calculated: 331.1428, found: 331.1429.

##### *N*-(4-Fluorobenzyl)­piperidine-4-carboxamide (**31**)

Boc-protected amine **28 (**336 mg, 1 mmol) was used in GP2 to afford amine **31** as colorless crystals (216 mg, 0.9 mmol, 91%). ^1^H NMR (400 MHz, D_2_O): δ = 7.30 (dd, *J* = 8.5, 5.4 Hz, 2H), 7.11 (t, *J* = 8.9 Hz, 2H), 4.34 (s, 2H), 3.50 (dt, *J* = 13.2, 3.6 Hz, 2H), 3.07 (td, *J* = 12.9, 3.2 Hz, 2H), 2.67 (tt, *J* = 11.7, 3.8 Hz, 1H), 2.07 (dd, *J* = 14.9, 3.8 Hz, 2H), 1.97–1.79 (m, 2H). ^13^C NMR (100 MHz, D_2_O): δ = 176.0, 161.8 (d, *J* = 242.7 Hz), 133.8 (d, *J* = 3.1 Hz), 129.0 (d, *J* = 8.2 Hz), 115.3 (d, *J* = 21.6 Hz), 43.1, 42.3, 39.7, 25.0. HRMS (ESI): [M + H^+^, C_13_H_18_NO_2_F^+^], calculated: 237.1398, found: 237.1395.

##### *N*-(4-Fluorophenethyl)­piperidine-4-carboxamide (**32**)

Boc-protected amine **29** (439 mg, 1.25 mmol) was used in GP2 to afford amine **32** as colorless crystals (253 mg, 1.0 mmol, 72%). ^1^H NMR (400 MHz, MeOD): δ = 7.25–7.17 (m, 2H), 7.04–6.96 (m, 2H), 3.42–3.33 (m, 2H), 3.04 (dt, *J* = 12.7, 3.4 Hz, 2H), 2.77 (t, *J* = 7.2 Hz, 2H), 2.57 (td, *J* = 12.5, 3.0 Hz, 2H), 2.27 (tt, *J* = 11.6, 3.9 Hz, 1H), 1.73–1.50 (m, 4H). ^13^C NMR (100 MHz, MeOD): δ = 180.4, 177.8, 163.0 (d, *J* = 242.6 Hz), 136.4 (d, *J* = 3.2 Hz), 131.5 (d, *J* = 7.9 Hz), 116.0 (d, *J* = 21.4 Hz), 46.3, 44.3, 41.7, 35.6, 30.2. HRMS (ESI): [M + H^+^, C_14_H_20_NO_2_F^+^], calculated: 251.1554, found: 251.1553.

##### *N*-(4-Fluorobenzyl)­azetidine-3-carboxamide (**33**)

Boc-protected amine **30 (**617 mg, 2.0 mmol) was used in GP2 to afford amine **33** as colorless crystals (374 mg, 1.8 mmol, 90%). ^1^H NMR (400 MHz, D_2_O): δ = 7.33 (dd, *J* = 8.4, 5.5 Hz, 2H), 7.20–7.09 (m, 2H), 4.39 (s, 2H), 4.27 (dd, *J* = 8.3, 3.9 Hz, 4H), 3.83–3.72 (m, 1H). ^13^C NMR (100 MHz, D_2_O): δ = 172.0, 161.9 (d, *J* = 243.1 Hz), 133.4, 129.2 (d, *J* = 8.8 Hz), 115.4 (d, *J* = 21.8 Hz), 48.1, 42.6, 35.6. HRMS (ESI): [M + H^+^, C_11_H_14_N_2_OF^+^], calculated: 209.1805, found: 209.1801.

##### Ethyl 6-Chloro-2-methylnicotinate (**34**)

6-Chloronicotinate **34** was prepared according to a literature procedure.^59^ ^1^H NMR (400 MHz, CDCl_3_): δ = 8.13 (d, *J* = 8.3 Hz, 1H), 7.23–7.19 (m, 1H), 4.36 (qd, *J* = 7.1, 0.8 Hz, 2H), 2.79 (s, 3H), 1.38 (td, *J* = 7.2, 0.8 Hz, 3H). ^13^C NMR (100 MHz, CDCl_3_): δ = 165.8, 161.4, 153.3, 141.1, 124.5, 121.5, 61.6, 24.7, 14.3.

##### Ethyl 5,6-Dichloronicotinate (**35**)

5,6-Dichloronicotinic acid (2.4 g, 12.5 mmol, 1.0 equiv) was solved in EtOH (10 mL). The solution was cooled down to 0 °C and thionyl chloride (2 mL, 27.5 mmol, 2.2 equiv) was added dropwise. The solution was refluxed for 2 h. Upon completion of the reaction, the reaction mixture was concentrated *in vacuo*. NaHCO_3_ (50 mL) was added to the residue and the aqueous phase was extracted with ethyl acetate (3 × 20 mL).The combined organic layer was dried over MgSO_4_ and evaporated *in vacuo*. The crude product was purified by column chromatography on silica gel to yield 6-chloronicotinate **35** as colorless crystals (0.87 g, 4.0 mmol, 32%). ^1^H NMR (400 MHz, CDCl_3_): δ = 8.87 (d, *J* = 2.1 Hz, 1H), 8.34 (d, *J* = 2.0 Hz, 1H), 4.42 (q, *J* = 7.1 Hz, 2H), 1.41 (t, *J* = 7.1 Hz, 3H). ^13^C NMR (100 MHz, CDCl_3_): δ = 163.44, 153.18, 148.24, 139.35, 130.72, 126.47, 62.13, 14.22. HRMS (EI): [M, C_8_H_7_NO_2_Cl_2_], calculated: 218.9854, found: 218.9849.

##### Ethyl 5,6-Dichloro-2-Methylnicotinate (**36**)

6-Chloronicotinate **36** was prepared according to a literature procedure.^59^ ^1^H NMR (400 MHz, CDCl_3_): δ = 8.26 (s, 1H), 4.39 (qd, *J* = 7.1, 0.8 Hz, 2H), 2.79 (d, *J* = 0.9 Hz, 3H), 1.41 (td, *J* = 7.1, 0.8 Hz, 3H). ^13^C NMR (100 MHz, CDCl_3_): δ = 164.7, 158.9, 150.8, 140.8, 127.7, 125.6, 62.0, 24.2, 14.4.

##### Ethyl 6-Chloro-5-cyano-2-methylnicotinate (**37**)

6-Chloronicotinate **37** was prepared according to a literature procedure.^60^ ^1^H NMR (400 MHz, CDCl_3_): δ = 8.47 (s, 1H), 4.40 (q, *J* = 7.1 Hz, 2H), 2.88 (s, 3H), 1.40 (t, *J* = 7.1 Hz, 4H). ^13^C NMR (100 MHz, CDCl_3_): δ = 165.4, 163.8, 153.8, 144.7, 124.8, 114.2, 108.2, 62.4, 25.3, 14.3. HRMS (ESI): [M – H^+^, C_10_H_8_N2O_2_Cl^–^], calculated: 223.0280, found: 223.0277.

##### Ethyl 5-Chloro-6-(4-((4-fluorobenzyl)­carbamoyl)­piperazin-1-yl)­nicotinate (**6**)

6-Chloronicotinate **35 (**22 mg, 0.1 mmol) was used in GP3 to afford piperazinyl-pyridine **6** as colorless crystals (22 mg, 0.05 mmol, 52%) ^1^H NMR (400 MHz, CDCl_3_): δ = 8.74 (d, *J* = 2.0 Hz, 1H), 8.14 (d, *J* = 2.0 Hz, 1H), 7.29 (dd, *J* = 8.4, 5.5 Hz, 3H), 7.05–6.97 (m, 2H), 4.84 (t, *J* = 5.5 Hz, 1H), 4.41 (d, *J* = 5.5 Hz, 2H), 4.36 (q, *J* = 7.1 Hz, 2H), 3.55 (s, 8H), 1.37 (t, *J* = 7.1 Hz, 3H).^13^C NMR (100 MHz, CDCl_3_): δ = 164.7, 159.9, 157.7, 143.9 (d, *J* = 782.1 Hz), 135.2, 129.6 (d, *J* = 7.9 Hz), 120.4 (d, *J* = 13.7 Hz), 115.6 (d, *J* = 21.5 Hz), 61.3, 48.3, 44.5, 43.7, 14.4. HRMS (ESI): [M–H^+^, C_20_H_21_N_4_O_3_F^–^], calculated: 419.1292, found: 419.1286.

##### Ethyl 5-Cyano-6-(4-((4-fluorobenzyl)­carbamoyl)­piperazin-1-yl)-2-methylnicotinate (**7**)

6-Chloronicotinate **37** (22 mg, 0.1 mmol) was used in GP3 to afford piperazinyl-pyridine **7** as colorless solid (23 mg, 0.05 mmol, 54%). ^1^H NMR (400 MHz, CDCl_3_): δ = 8.35 (s, 1H), 7.28 (dt, *J* = 6.3, 2.8 Hz, 2H), 7.04–6.97 (m, 2H), 4.87 (t, *J* = 5.8 Hz, 1H), 4.40 (d, *J* = 5.5 Hz, 2H), 4.32 (q, *J* = 7.2 Hz, 2H), 3.99–3.91 (m, 4H), 3.60–3.53 (m, 4H), 2.72 (s, 3H), 1.37 (t, *J* = 7.1 Hz, 3H).^13^C NMR (100 MHz, CDCl_3_): δ = 164.6, 164.5, 162.2 (d, *J* = 245.5 Hz), 158.4, 157.4, 147.7, 135.0 (d, *J* = 3.1 Hz), 129.5 (d, *J* = 8.0 Hz), 117.9, 115.5 (d, *J* = 21.5 Hz), 89.3, 61.1, 46.6, 44.3, 43.2, 25.7, 14.3.HRMS (ESI): [M + Na^+^, C_22_H_24_N_5_O_3_FNa^+^], calculated: 448.1755, found: 448.1748.

##### Ethyl 6-(4-((4-Fluorobenzyl)­carbamoyl)­piperidin-1-yl)-2-methylnicotinate (**9**)

6-Chloronicotinate **34 (**80 mg, 0.4 mmol) was used in GP3 to afford piperidinyl-pyridine **9** as colorless crystals (67 mg, 0.19 mmol, 81%). ^1^H NMR (400 MHz, CDCl_3_): δ = 8.02 (d, *J* = 9.0 Hz, 1H), 7.25–7.20 (m, 2H), 7.05–6.97 (m, 2H), 6.45 (d, *J* = 9.0 Hz, 1H), 5.79 (d, *J* = 7.2 Hz, 1H), 4.51 (dd, *J* = 10.3, 6.8 Hz, 2H), 4.41 (d, *J* = 5.1 Hz, 2H), 4.29 (q, *J* = 7.1 Hz, 2H), 2.94 (t, *J* = 12.6 Hz, 2H), 2.70 (s, 3H), 2.40 (tt, *J* = 11.5, 3.8 Hz, 1H), 1.95 (d, *J* = 13.2 Hz, 2H), 1.84–1.68 (m, 2H), 1.36 (t, *J* = 7.1 Hz, 3H).^13^C NMR (100 MHz, CDCl_3_): δ = 174.1, 166.7, 163.5, 161.0, 160.6, 140.5, 134.1 (d, *J* = 3.1 Hz), 129.5 (d, *J* = 8.1 Hz), 115.6 (d, *J* = 21.5 Hz), 113.4, 102.8, 60.2, 44.3, 43.5, 42.8, 28.4, 25.5, 14.4. HRMS (ESI): [M + H^+^, C_22_H_27_N_3_O_3_F^+^], calculated: 400.2031, found: 400.2028.

##### Ethyl 5-Chloro-6-(4-((4-fluorobenzyl)­carbamoyl)­piperidin-1-yl)­nicotinate (**10**)

6-Chloronicotinate **35 (**44 mg, 0.2 mmol) was used in GP3 to afford piperidinyl-pyridine **10** as colorless crystals (80 mg, 0.19 mmol, 95%). ^1^H NMR (400 MHz, CDCl_3_): δ = 8.72 (d, *J* = 2.0 Hz, 1H), 8.10 (d, *J* = 2.0 Hz, 1H), 7.23 (ddd, *J* = 8.3, 5.3, 2.5 Hz, 3H), 7.05–6.97 (m, 2H), 5.86 (d, *J* = 6.2 Hz, 1H), 4.42 (d, *J* = 5.7 Hz, 2H), 4.35 (q, *J* = 7.1 Hz, 2H), 4.21–4.12 (m, 2H), 3.00–2.87 (m, 2H), 2.37 (tt, *J* = 10.1, 4.9 Hz, 1H), 2.03–1.85 (m, 4H), 1.37 (t, *J* = 7.1 Hz, 3H). ^13^C NMR (100 MHz, CDCl_3_): δ = 174.4, 164.8, 162.4 (d, *J* = 246.0 Hz), 160.4, 147.8, 139.9, 134.2 (d, *J* = 3.3 Hz), 129.6 (d, *J* = 8.2 Hz), 120.4, 119.9, 115.7 (d, *J* = 21.5 Hz), 61.2, 48.6, 43.5, 43.0, 28.9, 14.4. HRMS (ESI): [M + H^+^, C_21_H_24_N_3_O_3_FCl^+^], calculated: 420.1485, found: 420.1481.

##### Ethyl 5-Chloro-6-(4-((4-fluorobenzyl)­carbamoyl)­piperidin-1-yl)-2-methylnicotinate (**11**)

6-Chloronicotinate **36 (**68 mg, 0.17 mmol) was used in GP3 to afford piperidinyl-pyridine **11** as colorless crystals (44 mg, 0.10 mmol, 60%). ^1^H NMR (400 MHz, CDCl_3_): δ = 7.25–7.21 (m, 2H), 7.07–6.97 (m, 2H), 5.79 (s, 1H), 4.43 (d, *J* = 5.7 Hz, 2H), 4.32 (q, *J* = 7.1 Hz, 2H), 4.20 (d, *J* = 12.9 Hz, 2H), 2.91 (td, *J* = 13.6, 12.2, 3.5 Hz, 2H), 2.69 (s, 3H), 2.36 (tt, *J* = 10.2, 4.7 Hz, 1H), 2.01–1.84 (m, 5H), 1.37 (t, *J* = 7.2 Hz, 3H). ^13^C NMR (100 MHz, CDCl_3_): δ = 174.5, 165.7, 162.4 (d, *J* = 245.8 Hz), 158.3, 157.8, 141.5, 134.3, 129.6 (d, *J* = 8.2 Hz), 118.2, 117.1, 115.8 (d, *J* = 21.5 Hz), 61.0, 48.4, 43.7, 43.0, 28.9, 24.7, 14.5. HRMS (ESI): [M + H^+^, C_22_H_26_N_3_O_3_F^+^], calculated: 434.1641, found: 434.1636.

##### Ethyl 5-Cyano-6-(4-((4-fluorobenzyl)­carbamoyl)­piperidin-1-yl)-2-methylnicotinate (**12**)

6-Chloronicotinate **37 (**56 mg, 0.25 mmol) was used in GP3 to afford piperidinyl-pyridine **12** as colorless crystals (86 mg, 0.19 mmol, 81%). ^1^H NMR (400 MHz, CDCl_3_): δ = 8.34 (s, 1H), 7.23 (dd, *J* = 6.2, 3.0 Hz, 2H), 7.02 (dd, *J* = 9.5, 7.8 Hz, 2H), 5.77 (s, 1H), 4.75 (d, *J* = 13.5 Hz, 2H), 4.42 (d, *J* = 5.6 Hz, 2H), 4.31 (q, *J* = 7.1 Hz, 2H), 3.15 (ddd, *J* = 13.9, 11.6, 2.8 Hz, 2H), 2.71 (s, 3H), 2.45 (tt, *J* = 11.3, 4.1 Hz, 1H), 1.99 (dd, *J* = 13.3, 3.6 Hz, 3H), 1.86 (qd, *J* = 11.8, 4.0 Hz, 2H), 1.37 (td, *J* = 7.1, 0.9 Hz, 3H). ^13^C NMR (100 MHz, CDCl_3_): δ = 173.7, 164.7, 164.4, 161.0 (d, *J* = 505.2 Hz), 158.4, 147.8, 129.5 (d, *J* = 8.2 Hz), 118.2, 115.7 (d, *J* = 21.5 Hz), 114.8, 89.0, 60.9, 46.9, 43.2, 42.9, 28.7, 25.7, 14.3. HRMS (ESI): [M + H^+^, C_23_H_26_N_4_O_3_F^+^], calculated: 425.1983, found: 425.1978.

##### Ethyl 6-(4-((4-Fluorophenethyl)­carbamoyl)­piperidin-1-yl)-2-methylnicotinate (**15**)

6-Chloronicotinate **34 (**40 mg, 0.2 mmol) was used in GP3 to afford piperidinyl-pyridine **15** as colorless crystals (35 mg, 0.09 mmol, 42%). ^1^H NMR (400 MHz, CDCl_3_): δ = 8.02 (d, *J* = 8.9 Hz, 1H), 7.18–7.09 (m, 2H), 7.04–6.95 (m, 2H), 6.44 (d, *J* = 9.0 Hz, 1H), 5.46 (s, 1H), 4.54–4.43 (m, 2H), 4.29 (q, *J* = 7.1 Hz, 2H), 3.50 (q, *J* = 6.7 Hz, 2H), 2.91 (td, *J* = 13.6, 12.7, 2.8 Hz, 2H), 2.79 (t, *J* = 6.9 Hz, 2H), 2.70 (s, 3H), 2.30 (tt, *J* = 11.5, 3.9 Hz, 1H), 1.86 (dd, *J* = 13.4, 3.5 Hz, 2H), 1.75–1.62 (m, 2H), 1.36 (t, *J* = 7.1 Hz, 3H). ^13^C NMR (100 MHz, CDCl_3_): δ = 174.4, 166.9, 161.8 (d, *J* = 244.7 Hz), 160.8, 159.0, 140.7, 134.5 (d, *J* = 3.4 Hz), 130.3 (d, *J* = 7.9 Hz), 115.6 (d, *J* = 21.2 Hz), 113.5, 103.0, 60.3, 44.4, 43.6, 40.7, 35.0, 28.5, 25.6, 14.6. HRMS (ESI): [M + H^+^, C_23_H_29_N_3_O_3_F^+^], calculated: 414.2187, found: 414.2180.

##### Ethyl 5-Chloro-6-(4-((4-fluorophenethyl)­carbamoyl)­piperidin-1-yl)­nicotinate (**16**)

6-Chloronicotinate **35 (**44 mg, 0.2 mmol) was used in GP3 to afford piperidinyl-pyridine **16** as colorless solid (83 mg, 0.19 mmol, 96%). ^1^H NMR (400 MHz, CDCl_3_): δ = 8.72 (dd, *J* = 2.0, 0.8 Hz, 1H), 8.10 (dd, *J* = 2.0, 0.8 Hz, 1H), 7.18–7.11 (m, 2H), 7.05–6.94 (m, 2H), 5.53 (t, *J* = 6.0 Hz, 1H), 4.40–4.30 (m, 2H), 4.20–4.07 (m, 2H), 3.51 (td, *J* = 7.0, 5.9 Hz, 2H), 2.90 (ddd, *J* = 13.4, 10.2, 4.3 Hz, 2H), 2.80 (t, *J* = 7.0 Hz, 2H), 2.27 (tt, *J* = 10.4, 5.5 Hz, 1H), 1.92–1.76 (m, 4H), 1.37 (dd, *J* = 7.6, 6.8 Hz, 3H). ^13^C NMR (100 MHz, CDCl_3_): δ = 174.5, 164.8, 161.8 (d, *J* = 244.6 Hz), 160.4, 147.8, 139.9, 134.6 (d, *J* = 3.4 Hz), 130.3 (d, *J* = 7.8 Hz), 120.3, 119.8, 115.6 (d, *J* = 21.2 Hz), 61.2, 48.5, 43.5, 40.7, 35.0, 28.9, 14.4. HRMS (ESI): [M + H^+^, C_22_H_26_N_3_O_3_FCl^+^], calculated: 434.1641, found: 434.1636.

##### Ethyl 5-Chloro-6-(4-((4-fluorophenethyl)­carbamoyl)­piperidin-1-yl)-2-methylnicotinate (**17**)

6-Chloronicotinate **36** (35 mg, 0.15 mmol) was used in GP3 to afford piperidinyl-pyridine **17** as colorless solid (13 mg, 0.03 mmol, 15%). ^1^H NMR (400 MHz, CDCl_3_): δ = 8.08 (s, 1H), 7.15 (dd, *J* = 8.4, 5.5 Hz, 2H), 7.00 (t, *J* = 8.7 Hz, 2H), 5.51 (s, 1H), 4.32 (q, *J* = 7.1 Hz, 2H), 4.17 (d, *J* = 13.1 Hz, 2H), 3.51 (q, *J* = 6.7 Hz, 2H), 2.88 (td, *J* = 13.2, 4.0 Hz, 2H), 2.80 (t, *J* = 6.9 Hz, 2H), 2.68 (s, 3H), 2.26 (tt, *J* = 10.6, 5.4 Hz, 1H), 1.86 (q, *J* = 4.1 Hz, 4H), 1.37 (t, *J* = 7.1 Hz, 3H). ^13^C NMR (100 MHz, CDCl_3_): δ = 174.6, 165.7, 164.4–156.3 (m), 141.5, 134.6 (d, *J* = 3.3 Hz), 130.3 (d, *J* = 7.8 Hz), 118.2, 117.0, 115.6 (d, *J* = 21.2 Hz), 61.0, 48.3, 43.6, 40.7, 35.0, 28.9, 24.7, 14.5. HRMS (ESI): [M + Na^+^, C_23_H_27_N_3_O_3_ClFNa^+^], calculated: 470.1617, found: 470.1612.

##### Ethyl 5-Cyano-6-(4-((4-fluorophenethyl)­carbamoyl)­piperidin-1-yl)-2-methylnicotinate (**18**)

6-Chloronicotinate **37 (**45 mg, 0.2 mmol) was used in GP3 to afford piperidinyl-pyridine **18** as colorless solid (55 mg, 0.13 mmol, 65%). ^1^H NMR (400 MHz, CDCl_3_): δ = 8.31 (s, 1H), 7.17–7.07 (m, 2H), 7.02–6.93 (m, 2H), 5.64 (t, *J* = 5.9 Hz, 1H), 4.76–4.65 (m, 2H), 4.29 (q, *J* = 7.1 Hz, 2H), 3.48 (td, *J* = 7.0, 5.9 Hz, 2H), 3.16–3.06 (m, 2H), 2.78 (t, *J* = 7.0 Hz, 2H), 2.69 (s, 3H), 2.35 (tt, *J* = 11.3, 4.2 Hz, 1H), 1.95–1.70 (m, 5H), 1.36 (t, *J* = 7.1 Hz, 3H). ^13^C NMR (100 MHz, CDCl_3_): δ = 174.0, 164.8, 164.5, 161.7 (d, *J* = 244.6 Hz), 158.4, 147.8, 134.5 (d, *J* = 3.3 Hz), 130.2 (d, *J* = 7.8 Hz), 118.2, 115.5 (d, *J* = 21.2 Hz), 114.8, 89.0, 61.0, 47.0, 43.1, 40.7, 34.9, 28.8, 25.8, 14.4. HRMS (ESI): [M + H^+^, C_24_H_28_N_4_O_3_F^+^], calculated: 439.2140, found: 439.2133.

##### Ethyl 6-(3-((4-Fluorobenzyl)­carbamoyl)­azetidin-1-yl)-2-methylnicotinate (**20**)

6-Chloronicotinate **34 (**44 mg, 0.2 mmol) was used in GP3 to afford azetidinyl-pyridine **20** as colorless solid (20 mg, 0.05 mmol, 27%). ^1^H NMR (400 MHz, CDCl_3_): δ = 8.01 (d, *J* = 8.6 Hz, 1H), 7.30–7.19 (m, 3H), 7.07–6.95 (m, 2H), 6.10 (d, *J* = 8.7 Hz, 1H), 6.02 (s, 1H), 4.44 (d, *J* = 5.8 Hz, 2H), 4.36–4.19 (m, 6H), 3.41 (tt, *J* = 8.2, 6.2 Hz, 1H), 2.69 (s, 3H), 1.35 (t, *J* = 7.1 Hz, 3H). ^13^C NMR (100 MHz, CDCl_3_): δ = 171.7, 167.0, 163.6, 161.2, 160.8 (d, *J* = 64.8 Hz), 140.1, 133.8 (d, *J* = 3.3 Hz), 129.7 (d, *J* = 8.1 Hz), 115.8 (d, *J* = 21.5 Hz), 114.4, 102.3, 60.4, 53.1, 43.3, 34.8, 25.4, 14.5. HRMS (ESI): [M + H^+^, C_20_H_23_N_3_O_3_F^+^], calculated: 372.1718, found: 372.1714.

##### Ethyl 5-Chloro-6-(3-((4-fluorobenzyl)­carbamoyl)­azetidin-1-yl)­nicotinate (**21**)

6-Chloronicotinate **35 (**44 mg, 0.2 mmol) was used in GP3 to afford azetidinyl-pyridine **21** as colorless solid (40 mg, 0.1 mmol, 51%). ^1^H NMR (400 MHz, DMSO-*d*_6_): δ = 8.58 (s, 1H), 8.56 (d, *J* = 2.0 Hz, 1H), 7.92 (d, *J* = 1.9 Hz, 1H), 7.30 (dd, *J* = 8.4, 5.7 Hz, 2H), 7.15 (t, *J* = 8.7 Hz, 2H), 4.45 (t, *J* = 8.9 Hz, 2H), 4.29 (ddt, *J* = 20.0, 14.1, 7.0 Hz, 6H), 3.57–3.44 (m, 1H), 1.29 (t, *J* = 7.1 Hz, 3H). ^13^C NMR (100 MHz, DMSO-*d*_6_): δ = 171.4, 164.0, 161.2 (d, *J* = 242.4 Hz), 156.8, 148.2, 137.8, 135.4 (d, *J* = 3.0 Hz), 129.3 (d, *J* = 8.0 Hz), 116.0, 115.1 (d, *J* = 21.4 Hz), 113.5, 60.5, 55.0, 41.6, 33.2, 14.2. HRMS (ESI): [M + H^+^, C_19_H_20_N_3_O_3_FCl^+^], calculated: 392.1172, found: 392.1168.

##### Ethyl 5-Cyano-6-(3-((4-fluorobenzyl)­carbamoyl)­azetidin-1-yl)-2-methylnicotinate (**22**)

6-Chloronicotinate **37 (**45 mg, 0.2 mmol) was used in GP3 to afford azetidinyl-pyridine **22** as colorless solid (23 mg, 0.06 mmol, 29%). ^1^H NMR (400 MHz, CDCl_3_): δ = 8.25 (s, 1H), 7.28–7.23 (m, 2H), 7.07–6.99 (m, 2H), 5.91 (s, 1H), 4.62–4.49 (m, 4H), 4.44 (d, *J* = 5.7 Hz, 2H), 4.30 (q, *J* = 7.1 Hz, 2H), 3.40 (tt, *J* = 8.6, 6.2 Hz, 1H), 2.70 (s, 3H), 1.36 (t, *J* = 7.1 Hz, 3H). ^13^C NMR (100 MHz, CDCl_3_): δ = 170.9, 165.1 (d, *J* = 48.3 Hz), 163.6, 161.1, 158.1, 146.1, 133.5 (d, *J* = 3.2 Hz), 129.7 (d, *J* = 8.1 Hz), 117.2, 115.8 (d, *J* = 21.5 Hz), 114.3, 86.6, 60.9, 54.3, 43.3, 34.5, 25.7, 14.3. HRMS (ESI): [M + H^+^, C_21_H_22_N_4_O_3_F^+^], calculated: 397.1670, found: 397.1667.

##### 2-Chloro-5-oxo-5,7-dihydrofuro­[3,4-*b*]­pyridine-3-carbonitrile (**38**)

Fused-lactone **38** was prepared according to a literature procedure.^37^ ^1^H NMR (400 MHz, DMSO-*d*_6_): δ = 9.12 (d, *J* = 5.2 Hz, 1H), 5.74–5.39 (m, 2H). ^13^C NMR (100 MHz, DMSO-*d*_6_): δ = 170.8, 166.6, 156.8, 141.8, 119.0, 114.6, 110.3, 70.5.

##### 4-(3-Cyano-5-oxo-5,7-dihydrofuro­[3,4-*b*]­pyridin-2-yl)-*N*-(4-fluorobenzyl)­piperazine-1-carboxamide (**8**)

Fused-lactone **38 (**19 mg, 0.1 mmol) was used in GP3 to afford compound **8** as colorless solid (9 mg, 0.09 mmol, 23%). ^1^H NMR (400 MHz, CDCl_3_): δ = 8.24 (s, 1H), 7.32–7.26 (m, 2H), 7.06–6.99 (m, 2H), 5.30 (s, 4H), 5.13 (d, *J* = 0.5 Hz, 2H), 4.76 (t, *J* = 5.6 Hz, 1H), 4.42 (d, *J* = 5.5 Hz, 2H), 4.05–3.98 (m, 4H), 3.69–3.59 (m, 4H), 1.57 (s, 3H). ^13^C NMR (100 MHz, CDCl_3_): δ = 170.4, 167.6, 162.4 (d, *J* = 245.8 Hz), 162.0, 157.3, 143.2, 135.0, 129.7 (d, *J* = 8.1 Hz), 117.2, 115.7 (d, *J* = 21.4 Hz), 109.7, 94.1, 70.1, 47.5, 44.5, 43.2. HRMS (ESI): [M + Cl^–^, C_20_H_18_N_5_O_3_FCl^–^], calculated: 430.1088, found: 430.1085.

##### 1-(3-Cyano-5-oxo-5,7-dihydrofuro­[3,4-*b*]­pyridin-2-yl)-*N*-(4-fluorobenzyl)­piperidine-4-carboxamide (**14**)

Fused-lactone **38 (**39 mg, 0.2 mmol) was used in GP3 to afford compound **14** as colorless solid (17 mg, 0.04 mmol, 22%). ^1^H NMR (400 MHz, DMSO-*d*_6_): δ = 8.55 (s, 1H), 8.42 (t, *J* = 6.0 Hz, 1H), 7.31–7.22 (m, 2H), 7.19–7.08 (m, 2H), 4.54 (d, *J* = 13.3 Hz, 2H), 4.25 (d, *J* = 5.4 Hz, 2H), 3.32–3.20 (m, 2H), 2.58 (ddt, *J* = 11.1, 8.2, 4.0 Hz, 1H), 1.97–1.83 (m, 2H), 1.76–1.63 (m, 2H). ^13^C NMR (100 MHz, DMSO-*d*_6_): δ = 173.6, 170.8, 167.7, 165.1 (d, *J* = 559.5 Hz), 161.4, 143.3, 136.0–135.6 (m), 129.0 (d, *J* = 8.1 Hz), 117.5, 115.0 (d, *J* = 21.2 Hz), 108.2, 92.9, 69.9, 54.9, 47.4, 41.2 (d, *J* = 8.5 Hz), 28.3. HRMS (ESI): [M + Cl^–^, C_21_H_19_N_4_O_3_FCl^–^], calculated: 429.1135, found: 429.1133.

##### Ethyl 4-Fluoro-2-hydroxybenzoate (**40**)

4-fluoro-2-hydroxybenzoic acid **39** (780 mg, 5 mmol, 1 equiv) was solved in ethanol (10 mL). Concentrated H_2_SO_4_ (0.5 mL) was added dropwise and the solution was refluxed for 16 h at 80 °C. The reaction mixture was cooled down to rt and the solvent was evaporated *in vacuo*. Ethyl acetate (10 mL) and water (10 mL) were added to the residual and the water phase was extracted with ethyl acetate (2 × 10 mL). The combined organic layer was washed with brine (10 mL), dried over MgSO_4_ and evaporated *in vacuo*. The crude product was purified by column chromatography on silica gel to yield **40** as colorless solid (596 mg, 3.2 mmol, 65%). ^1^H NMR (400 MHz, CDCl_3_): δ = 11.09 (s, 1H), 7.85 (dd, *J* = 8.9, 6.6 Hz, 1H), 6.66 (dd, *J* = 10.3, 2.5 Hz, 1H), 6.59 (ddd, *J* = 9.0, 8.2, 2.5 Hz, 1H), 4.41 (q, *J* = 7.1 Hz, 2H), 1.41 (t, *J* = 7.1 Hz, 3H). ^13^C NMR (100 MHz, CDCl_3_): δ = 169.7, 167.3 (d, *J* = 254.5 Hz), 163.9 (d, *J* = 14.2 Hz), 132.2 (d, *J* = 11.4 Hz), 109.5 (d, *J* = 2.6 Hz), 107.4 (d, *J* = 22.7 Hz), 104.5 (d, *J* = 24.3 Hz), 61.7, 14.3. HRMS (EI): [M, C_9_H_9_O_3_F], calculated: 184.0536, found: 184.0530.

##### Ethyl 4-Fluoro-2-propoxybenzoate (**41**)

2-Hydroxybenzoate **40** (575 mg, 3.125 mmol, 1.0 equiv) and K_2_CO_3_ (520 mg, 3.75 mmol, 1.2 equiv) were solved in dry MEK (15 mL). Propyl bromide (400 μL, 4.4 mmol, 1.4 equiv) was added dropwise and the reaction mixture was refluxed for 24 h at 90 °C. The reaction mixture was cooled down to rt and filtered through Celite. The solvent was evaporated *in vacuo* and ethyl acetate (10 mL) and water (10 mL) were added to the residual. The water phase was extracted with ethyl acetate (2 × 10 mL) and the combined organic layer was washed with brine (10 mL), dried over MgSO_4_ and evaporated *in vacuo*. The crude product was purified by column chromatography on silica gel to yield **41** as colorless crystals (679 mg, 3.0 mmol, 96%). ^1^H NMR (400 MHz, CDCl_3_): δ = 7.83 (dd, *J* = 9.1, 6.8 Hz, 1H), 6.71–6.60 (m, 2H), 4.35 (q, *J* = 7.0 Hz, 2H), 3.97 (t, *J* = 6.2 Hz, 2H), 1.87 (q, *J* = 6.8 Hz, 2H), 1.37 (t, *J* = 6.7 Hz, 3H), 1.08 (t, *J* = 7.2 Hz, 3H). ^13^C NMR (100 MHz, CDCl_3_): δ = 167.3, 164.8, 160.8 (d, *J* = 10.6 Hz), 133.9 (d, *J* = 10.9 Hz), 116.9, 107.0 (d, *J* = 21.8 Hz), 101.0 (d, *J* = 25.5 Hz), 70.8, 61.1, 22.6, 14.5, 10.7. HRMS (EI): [M, C_12_H_15_O_3_F], calculated: 226.1005, found: 226.0999.

##### Ethyl 5-Chloro-4-fluoro-2-propoxybenzoate (**42**)

2-Propoxybenzoate **41** (320 mg, 1.4 mmol, 1 equiv) was solved in MeCN (3 mL). Concentrated H_2_SO_4_ (80 μL, 1.5 mmol, 1.05 equiv), was added dropwise and the solution was stirred for 5 min at rt. NCS was added (198 mg, 1.5 mmol, 1.05 equiv), and the reaction mixture was refluxed for 24 h at 95 °C. The reaction was cooled down to rt and the solvent was evaporated *in vacuo*. Ethyl acetate (10 mL) and water (10 mL) were added to the residual and the water phase was extracted with ethyl acetate (2 × 10 mL). The combined organic layer was washed with brine (10 mL), dried over MgSO_4_ and evaporated *in vacuo*. The crude product was purified by column chromatography on silica gel to yield **42** as colorless crystals (121 mg, 0.38 mmol, 33%). ^1^H NMR (400 MHz, CDCl_3_): δ = 7.88 (d, *J* = 8.6 Hz, 1H), 6.74 (d, *J* = 11.0 Hz, 1H), 4.34 (q, *J* = 7.1 Hz, 2H), 3.95 (t, *J* = 6.4 Hz, 2H), 1.86 (dtd, *J* = 13.8, 7.4, 6.4 Hz, 2H), 1.37 (t, *J* = 7.1 Hz, 3H), 1.07 (t, *J* = 7.4 Hz, 3H). ^13^C NMR (100 MHz, CDCl_3_): δ = 164.6, 160.9 (d, *J* = 254.4 Hz), 159.1 (d, *J* = 9.4 Hz), 133.7 (d, *J* = 2.2 Hz), 117.6 (d, *J* = 3.4 Hz), 111.8 (d, *J* = 18.2 Hz), 102.2 (d, *J* = 24.8 Hz), 71.2, 61.3, 22.5, 14.4, 10.6. HRMS (EI): [M, C_12_H_14_O_3_ClF], calculated: 260.0616, found: 260.0610.

##### Ethyl 5-Chloro-4-(4-((4-fluorobenzyl)­carbamoyl)­piperidin-1-yl)-2-propoxybenzoate (**13**)

Propoxybenzoate **42 (**52 mg, 0.2 mmol) and amine **31** (57 mg, 0.24 mmol) were used in GP4 to afford 6-aminobenzyl amide **13** as colorless solid (32 mg, 0.07 mmol, 34%). ^1^H NMR (400 MHz, CDCl_3_): δ = 7.82 (s, 1H), 7.25 (dd, *J* = 8.6, 5.3 Hz, 2H), 7.08–6.95 (m, 2H), 6.51 (s, 1H), 5.88 (d, *J* = 6.1 Hz, 1H), 4.43 (d, *J* = 5.6 Hz, 2H), 4.30 (q, *J* = 7.1 Hz, 2H), 3.95 (t, *J* = 6.4 Hz, 2H), 3.54 (dd, *J* = 9.4, 5.7 Hz, 2H), 2.76–2.65 (m, 2H), 2.31 (p, *J* = 7.4 Hz, 1H), 1.98 (td, *J* = 10.1, 8.8, 3.7 Hz, 4H), 1.90–1.81 (m, 2H), 1.35 (t, *J* = 7.1 Hz, 3H), 1.06 (t, *J* = 7.4 Hz, 3H). ^13^C NMR (100 MHz, CDCl_3_): δ = 174.5, 165.2, 162.4 (d, *J* = 245.9 Hz), 159.0, 154.0, 134.2 (d, *J* = 3.3 Hz), 133.8, 129.6 (d, *J* = 8.1 Hz), 119.1, 115.7 (d, *J* = 21.5 Hz), 114.9, 105.5, 71.0, 60.8, 50.9, 43.0, 43.0, 29.1, 22.7, 14.4, 10.7, 1.2. HRMS (ESI): [M + Cl^–^, C_25_H_30_N_2_O_4_Cl_2_F^–^], calculated: 511.1572, found: 511.1565.

##### Ethyl 5-Chloro-4-(4-((4-fluorophenethyl)­carbamoyl)­piperidin-1-yl)-2-propoxybenzoate (**19**)

Propoxybenzoate **42 (**52 mg, 0.2 mmol) and amine **32** (57 mg, 0.24 mmol) were used in GP4 to afford 6-aminobenzyl amide **19** as colorless solid (16 mg, 0.03 mmol, 17%). ^1^H NMR (400 MHz, CDCl_3_): δ = 7.83 (s, 1H), 7.19–7.11 (m, 2H), 7.05–6.95 (m, 2H), 6.50 (s, 1H), 5.52 (t, *J* = 5.8 Hz, 1H), 4.31 (q, *J* = 7.1 Hz, 2H), 3.95 (t, *J* = 6.4 Hz, 2H), 3.52 (q, *J* = 6.6 Hz, 4H), 2.81 (t, *J* = 6.9 Hz, 2H), 2.69 (dt, *J* = 11.8, 6.9 Hz, 2H), 2.21 (p, *J* = 7.8 Hz, 1H), 1.96–1.78 (m, 6H), 1.36 (t, *J* = 7.1 Hz, 3H), 1.07 (t, *J* = 7.4 Hz, 3H). ^13^C NMR (100 MHz, CDCl_3_): δ = 174.6, 165.2, 159.8 (d, *J* = 160.8 Hz), 154.1, 134.6 (d, *J* = 3.3 Hz), 130.3 (d, *J* = 7.9 Hz), 119.1, 115.6 (d, *J* = 21.3 Hz), 114.9, 105.5, 71.0, 60.8, 50.9, 43.0, 40.7, 35.0, 29.1, 22.7, 14.5, 10.7. HRMS (ESI): [M + Na^+^, C_26_H_32_N_2_O_4_ClNa^+^], calculated: 513.1927, found: 513.1932.

##### *tert*-Butyl 4-((4-(4,4,5,5-Tetramethyl-1,3,2-dioxaborolan-2-yl)­benzyl)­carbamoyl)­piperidine-1-carboxylate (**43**)

Carboxylic acid **26 (**404 mg, 1.5 mmol) was used in GP1 with (4-(4,4,5,5-tetramethyl-1,3,2-dioxaborolan-2-yl)­phenyl)­methanamine (344 mg, 0.6 mmol) to afford **43** as colorless crystals (283 mg, 4.3 mmol, 42%).^1^H NMR (400 MHz, CDCl_3_): δ = 7.77 (d, *J* = 7.6 Hz, 2H), 7.25 (d, *J* = 7.5 Hz, 2H), 5.81 (d, *J* = 6.9 Hz, 1H), 4.44 (d, *J* = 5.7 Hz, 2H), 4.21–4.06 (m, 2H), 2.72 (t, *J* = 12.5 Hz, 2H), 2.25 (tt, *J* = 11.5, 3.7 Hz, 1H), 1.87–1.76 (m, 2H), 1.73–1.57 (m, 2H), 1.45 (s, 9H), 1.33 (s, 12H). ^13^C NMR (100 MHz, CDCl_3_): δ = 174.3, 154.8, 141.4, 135.4, 127.2, 84.0, 79.8, 43.7, 43.5, 28.8, 28.6, 25.0. HRMS (ESI): [M + Na^+^, C_24_H_37_BN_2_O_5_Na^+^], calculated: 467.2688, found: 467.2682.

##### *N*-(4-(4,4,5,5-Tetramethyl-1,3,2-dioxaborolan-2-yl)­benzyl)­piperidine-4-carboxamide (**44**)

Boc-protected amine **43** (222 mg, 0.5 mmol) was used in GP2 to afford amine **44** as colorless crystals (111 mg, 0.3 mmol, 65%).^1^H NMR (400 MHz, CDCl_3_): δ = 7.40–7.33 (m, 2H), 6.95 (d, *J* = 7.7 Hz, 2H), 4.05 (s, 2H), 3.09 (dt, *J* = 12.9, 3.6 Hz, 2H), 2.72 (td, *J* = 12.6, 3.3 Hz, 2H), 2.31 (ddt, *J* = 10.9, 7.9, 3.5 Hz, 1H), 1.76–1.51 (m, 4H), 0.99 (s, 12H). ^13^C NMR (100 MHz, CDCl_3_): δ = 175.7, 143.2, 135.9, 127.8, 85.0, 68.1, 44.3, 44.0, 40.8, 26.6, 25.2. HRMS (ESI): [M + H^+^, C_19_H_30_BN_2_O_5_^+^], calculated: 345.2344.2688, found: 345.2342.

##### Ethyl 5-Cyano-2-methyl-6-(4-((4-(4,4,5,5-tetramethyl-1,3,2-dioxaborolan-2-yl)­benzyl)­carbamoyl)­piperidin-1-yl)­nicotinate (**45**)

6-Chloronicotinate **37 (**45 mg, 0.2 mmol) was used in GP3 to afford piperidinyl-pyridine **45** as colorless crystals (67 mg, 0.17 mmol, 85%). ^1^H NMR (400 MHz, CDCl_3_): δ = 8.32 (s, 1H), 7.81–7.72 (m, 2H), 7.25 (dd, *J* = 7.0, 1.2 Hz, 2H), 5.90 (t, *J* = 5.7 Hz, 1H), 4.73 (dt, *J* = 13.7, 3.6 Hz, 2H), 4.44 (d, *J* = 5.7 Hz, 2H), 4.30 (q, *J* = 7.1 Hz, 2H), 3.14 (ddd, *J* = 14.0, 11.6, 2.8 Hz, 2H), 2.70 (s, 3H), 2.44 (tt, *J* = 11.2, 4.1 Hz, 1H), 1.97 (dd, *J* = 13.4, 3.7 Hz, 2H), 1.91–1.78 (m, 2H), 1.36 (t, *J* = 7.1 Hz, 3H), 1.33 (s, 11H). ^13^C NMR (100 MHz, CDCl_3_): δ = 173.8, 164.8, 164.5, 158.5, 147.9, 141.4, 135.4, 127.2, 118.3, 114.8, 89.0, 84.0, 61.0, 47.0, 43.7, 43.2, 28.8, 25.9, 25.0, 14.4. HRMS (ESI): [M + H^+^, C_29_H_38_N_4_O_5_B^+^], calculated: 533.2930, found: 533.2921.

### Cell Culture

P2Y12-expressing human astrocytoma cells (1321-N1-HA-P2Y12, Kerafast, Shirley, MA) were cultured in Dulbecco′s modified eagle medium (DMEM) containing 1% penicillin-streptomycin solution (Thermo Fisher Scientific, Waltham, MA), and 10% fetal bovine serum (Biochrome, Berlin, Germany). The cell cultures were maintained in an incubator at 37 °C under a humidified and 5% CO_2_-conditioned atmosphere. Cells were passaged at 80% confluency. For binding assays, cells were harvested with Trypsin-EDTA (5 mL, Gibco) and resuspended in medium. The suspension was centrifuged at 1000g for 5 min and the supernatant was discarded. The cells were adjusted to assay cell concentration (2 × 10^6^ cells/mL) by the addition of D-PBS and stored at 37 °C up to 1 h prior to the binding assay.

### Competitive Binding Assay

The IC_50_ value of compound **5**–**22** was determined utilizing a modified competitive radioligand binding protocol by Dupuis et al.^38^ Ligand concentrations (25 μL, 1–10 μM in DMSO) were prepared as triplicates together with [^33^P]­2-MeSADP (25 μL, 6.25 nM; Hartmann Analytic, Braunschweig, Germany) in PBS in 96-well plates. The assay was initiated by the addition of the cell suspension (200 μL, 10^7^ cells/mL) and incubation at 37 °C with gentle shaking at 200 rpm for 10 min. The mixture was transferred to a 96-well filter plate (1.0 μm glass fiber filter, MultiScreenHTS FB Filter Plate, Merck Millipore, Billercia, MA) and filtered with a vacuum manifold (MultiScreenHTS Vacuum Manifold, Merck Millipore, Billercia, MA). The filters were washed by the addition of cold PBS buffer (4 × 200 μL) and dried at rt for 1 h. Afterward, a punch-tip plate was mounted and the filters were separated into scintillation vials (10 mL) containing scintillation cocktail (4 mL, Rotiszint eco plus LSC-Universalcocktail). The vials were shortly vortexed and left to settle for 1 h, before the remaining radioactivity was quantified by liquid-scintillation counting (LSC, Tri-Carb 2810TR, PerkinElmer, Waltham, MA). Bound activity was plotted as a function of ligand concentration in GraphPad Prism 8.0 (GraphPad Software, version 8.4.3., San Diego, CA).

### Radiochemistry

The radiofluorination followed an adapted protocol developed by Hoffmann and co-workers.^39^ No carrier-added [^18^F]­fluoride was produced *via* ^18^O­(p,n)^18^F nuclear reaction by proton irradiation of ^18^O-enriched water and sent to a [^18^F]­fluoride incoming reservoir. [^18^F]­Fluoride solution (1 mL, 500–5000 MBq) was passed through a carbonated quaternary methylammonium cartridge (QMA) through the male side to trap [^18^F]­fluoride (reverse loading-elution protocol).^61^ The [^18^F]­fluoride was flushed with *n*-BuOH (2 mL) and then eluted with a solution of NBu_4_OTf (1 mg, 3.6 μmol) in *n*-BuOH (400 μL) into a solution of the precursor **42** (1.05 mg, 2.5 μmol, 1 equiv) and Cu­(TfO)_2_Py_4_ (6.78 mg, 10 μmol, 4 equiv) in DMI (800 μL). The reaction mixture was heated for 10 min at 110 °C under atmospheric air and then left to cool down for 5 min to rt. RCC was determined based on radio-TLC (EtOAc/Hex 5:5). The crude reaction mixture was diluted with HEPES buffer (8 mL, 1.5 M, pH = 5.7) and loaded on a C8 plus cartridge. After flushing with water (5 mL) and elution with EtOH (0.8 mL) the crude product was purified by semipreparative HPLC (Luna 5 μm PFP(2) 100 Å, LC Column 250 × 10 mm). The product fraction (5 mL) was collected, diluted with water (20 mL) and trapped on a C18 cartridge. The cartridge was flushed again with water (5 mL) and the product was eluted with EtOH (0.3 mL) and formulated by the addition of PBS (3 mL). Product identity and molar activities of the ready-to-use product solution were determined by HPLC (Luna 5 μm PFP(2) 100 Å, LC Column 250 × 4.6 mm). Representative HPLC chromatograms and HPLC conditions are provided in the Supporting Information (Figures S3–S6).

### Stability

Human blood was collected in citrate tubes (S-Monovette Citrat 9NC, Sarstedt, Nümbrecht, Germany). Blood (1 mL) was mixed with 10 MBq tracer solution (100 μL) and incubated at 37 °C with gentle shaking at 200 rpm. Samples (100 μL) were drawn at each time point, mixed with an equal volume of MeCN and centrifuged for 3 min at 20,817*g*. Plasma samples were then injected on radio-HPLC. Samples for kinetic stability were prepared by mixing PBS (1 mL, pH = 7.4) with 10 MBq tracer solution (100 μL) and incubated at 37 °C with gentle shaking at 200 rpm. Samples (100 μL) were drawn at each time point, and injected on radio-HPLC. Murine blood samples were prepared by mixing freshly collected blood from WT mice with 50 μL of 0.1 M sodium citrate solution (1:9 v/v). Blood (0.5 mL) was mixed with 1 MBq tracer solution (10 μL) and incubated at 37 °C with gentle shaking at 200 rpm. Samples (50 μL) were drawn at each time point, mixed with an equal volume of MeCN and centrifuged for 3 min at 20817g. Plasma samples were then injected on radio-HPLC. All samples (PBS, human and murine) were diluted to 5 mL with HPLC-grade water and injected onto the column-switch HPLC (Agilent Technologies 1200 series, Santa Clara, CA) for metabolite analysis (Figure S7).

### Log *D*_7.4_ Value

The distribution of [^18^F]**12** between *n*-octanol and PBS buffer was determined by the shake-flask method at rt.^29^ 1 mL of [^18^F]**12** tracer formulation (10 MBq/mL) in PBS was mixed with 1 mL *n*-octanol for one min using a vortex mixer. After 30 min, three samples (100 μL) were taken from each phase. The recovery rate (>97%) was determined by taking three additional samples (100 μL) from the formulation solution. Radioactivity for all samples was quantified by a γ-counter (counting time 1 min, counting window 450–570 keV, HIDEX AMG γ Counter version 1.6.0.0, Turku, Finland). The Log *D*_7.4_ value was calculated based on the ratio of radioactivity between the *n*-octanol phase (*A*_oct_) and the PBS phase (*A*_Buffer_) (log *D*_7.4_ = log­(*A*_oct_/*A*_Buffer_)) and reported as mean ± SD.

### *In Vitro* Autoradiography

WT, PLX and Trem2^–/–^ mouse brain sections were thawed to rt for 5 min and then preincubated with PBS buffer (pH = 7.4) for 10 min. Sections were then incubated with a maximum of 0.5 MBq/mL [^18^F]**12** tracer formulation at rt for 45 min in PBS buffer. To determine specificity, adjacent brain sections were coincubated with a 1000-fold excess of ticagrelor (1 μM) or a 1000-fold excess of nonradioactive compound **12** (1 μM) in the presence of 0.5 MBq/mL [^18^F]**12** tracer formulation. Subsequently, sections were washed with 30% ethanol for 1 min, 70% ethanol for 2 min and PBS for 1 min, before leaving them to dry at rt for 60 min. Afterward, brain sections were exposed to a phosphor imaging plate at rt for 24 h and then scanned with a CR-Reader (CR35 BIO, DÜRR MEDICAL, Bietigheim-Bissingen, Germany). The images were analyzed using Aida Image Analyzer software (v.4.50.010, Elysia-raytest GmbH, Straubenhardt, Germany). Quantification was performed by placing a region of interest (ROI) in the CTX and the hypothalamus (HYP) as pseudo reference region. Results are either expressed as normalized intensity relative to maximal intensity after background subtraction or as CTX to HYP ratio.

### PET Imaging

Mice (*n* = 8, average age WT = 10 months, PLX = 9 months, Trem2^–/–^ = 19 months, 4 male and 4 female for each group) were injected with 13.0 ± 5.6 MBq of [^18^F]**12** in 200 μL of phosphate buffer *via* the lateral tail vein under isoflurane anesthesia. The mice were scanned first with CT (70 kVp/650 μA, exposure time 300 ms, Helical 1.0 pitch). The scan was followed by a 60 min dynamic PET scan (with coincidence mode 1–5 in 1 scan position) under constant anesthesia with isoflurane (1.5% at 1.5 L flow per minute) using a Mediso Nanoscan PET/CT (Budapest, Hungary). For self-block experiments with WT mice (*n* = 2), nonradioactive compound **12** (60 nmol) was injected prior to the CT scan, followed by the tracer injection. PET/CT images were reconstructed using the Tera Tomo 3D algorithm (4 iterations and 6 subsets) and analyzed using PMOD (version 3.5; PMOD Technologies Ltd., Zurich, Switzerland). PET images of each mouse were coregistered with CT images of the corresponding mouse. A standardized global mean volume of interest (VOI) was used to assess tracer enrichment in the brain and to generate time-activity curves. For liver, bladder and kidney, VOIs were manually drawn as spheres. Data are reported as %ID/g or SUV. For each dynamic PET scan, V_T_ was calculated using the method described by Logan et al., employing a cardiac input function obtained from a standardized VOI.^62^ The resulting brain *V*_T_ image was then analyzed using a standardized global mean VOI. No corrections were made for radiometabolites.

### Biodistribution and Metabolite Analysis

60 min after tracer administration, mice were transferred to the anesthesia box for isoflurane overdose and euthanized by cervical dislocation. Mouse organs and blood samples were collected, weighed and counted in a γ-counter. Blood samples were obtained by cardiac puncture and transferred to EDTA tubes (Microvette 500 K3E, Sarstedt, Nümbrecht, Germany) to prevent platelet aggregation. Blood samples were centrifuged at 3000*g* for 5 min to isolate blood plasma. An equal volume of TFA solution (10%) was added to plasma to precipitate remaining plasma proteins. The precipitate was separated by centrifugation at 20,817*g* for 5 min. The plasma sample was diluted to 5 mL with HPLC-grade water and injected onto the column-switch HPLC (Agilent Technologies 1200 series, Santa Clara, CA) for metabolite analysis. Brain and liver samples were homogenized (Ika disperser T8.10) in a 1:1 mixture of MeCN and H_2_O and centrifuged at 3000*g* for 5 min. The supernatant was diluted to 5 mL with HPLC-grade water and injected onto the column-switch HPLC for metabolite analysis.

### Antibody Generation

For generation of monoclonal antibodies against mouse P2Y12R, a Lou/c rat was immunized subcutaneously (s.c.) and intraperitoneally (i.p.) with a mixture of 40 μg ovalbumin-coupled peptide corresponding to P2Y12R aa 325–342 TSGTNKKKGQEGGEPS (Peps4LS, Heidelberg) dissolved in 400 μL PBS containing 5 nmol CpG2003 (TIB MOLBIOL, Berlin, Germany), and 400 μL Incomplete Freund’s adjuvant (Sigma-Aldrich). After 8 weeks, a boost without Freund’s adjuvant was given i.p. and s.c. three days before fusion. Fusion of mouse immune spleen cells with the myeloma cell line P3 × 63-Ag8.653 was performed using poly­(ethylene glycol) 1500 according to standard procedure.^63^ After fusion, the cells were plated in 96-well plates in RPMI 1640 medium supplemented with 20% fetal calf serum, 1 mM pyruvate, 1× nonessential amino acids and HAT media supplement (Hybri-Max, Sigma-Aldrich). Hybridoma supernatants were screened 10 days later in a flow cytometry assay (iQue, Intellicyt; Sartorius) using biotinylated peptides captured on streptavidin beads (PolyAN, Berlin) and incubated for 90 min with hybridoma supernatant and Atto-488-coupled isotype-specific monoclonal mouse-antirat IgG secondary antibodies. Antibody binding was analyzed using ForeCyt software (Sartorius). Positive supernatants were further validated in capture ELISA, Western blot and immunohistochemistry. Hybridoma cells from selected supernatants were subcloned by limiting dilution to obtain stable monoclonal cells lines. Experiments in this work were performed with clone P2YM 17A12 (ratIgG2c/k).

### Immunohistochemistry

Paraformaldehyde-fixed 50 μm thick sagittal brain sections were incubated overnight at 4 °C in PBS with 5% normal goat serum and 0.5% Triton X-100 containing guinea pig monoclonal anti-Iba1 primary antibody (1:500, Synaptic Systems GmbH, Göttingen, Germany, 234308) and rat monoclonal anti-P2Y12R primary antibody (1:200, P2YM 17A12, this work). Sections were washed with PBS (3×) supplemented with 0.5% Triton X-100 and incubated for 2 h at rt with a suitable secondary antibody (antiguinea pig and antirat antibody Alexa Fluor, ThermoFisher) and Dapi. Imaging was conducted on the THUNDER Imager Tissue (Leica Microsystems CMS GmbH, Wetzlar, Germany) with a ×20 objective for zoom-in and ×10 objective for overview picture in two sagittal sections. The images were processed using the LAS X Software (version 3.9.1.28433), and analysis was conducted with FiJi/ImageJ (version 1.54f)^64^ by quantifying Iba1 and P2Y12R signal over a predefined threshold.

### Statistical Analysis

Group differences in PET, autoradiography experiments and immunohistology were identified with a one-way ANOVA and Tukey’s multiple comparisons test using GraphPad Prism statistical software (GraphPad Software, version 8.4.3., San Diego, CA). For correlation studies of IHC results and PET data regression lines were calculated including 95% confidence intervals. Pearson′s correlation coefficients are provided in each graph. A threshold of *p* < 0.05 was considered significant for the rejection of the null hypothesis.

## Supplementary Material







## Notes

Molecular formula strings (CSV)
Docked complex **6** (PDB)
Docking results with BBB and CNS MPO scores, NMR spectra and HPLC analysis for test compounds, competitive binding curves, HPLC chromatograms of the radiosynthesis, stability test and metabolism assessment, biodistribution data, PET and IHC images and plots of IHC, PET and *V*_T_ quantification (PDF)

## Abbreviations Used

%ID/g: percent injected dose per gram
AD: Alzheimer’s disease
AM: molar activity
ARG: autoradiography
AY: activity yield
BBB: blood-brain barrier
Boc: *tert*-butyloxycarbonyl
CNS: central nervous system
CNS MPO: central nervous system multiparameter optimization
CTX: cortex
DCC: *N*,*N*′-dicyclohexylcarbodiimide
DIPEA: *N*,*N*-diisopropylethylamine
DMEM: Dulbeccos Modified Eagle Medium
DMI: 1,3-dimethyl-2-imidazolidinone
D-PBS: Dulbelcco′s phosphate-buffered saline
HBTU: hexafluorophosphate benzotriazole tetramethyl uronium
HEPES: 4-(2-hydroxyethyl)-1-piperazineethanesulfonic acid
HPLC: high-performance liquid chromatography
HRMS: high-resolution mass spectrometry
HYP: hypothalamus
i.p.: intraperitoneally
IC_50_: half maximal inhibitory concentration
IHC: immunohistochemistry
LSC: liquid scintillation counting
MOE: molecular operating environment
MW: molecular weight
NCS: *N*-chlorosuccinimide
NMR: nuclear magnetic resonance
NSB: nonspecific binding
p.i.: post injection
P2Y12R: purinergic 2Y type 12 receptor
PBS: phosphate-buffered saline
PDB: Protein Data Bank
PET: positron emission tomography
QMA: quaternary methylammonium cartridge
RCP: radiochemical purity
RCY: radiochemical yield
ROI: region of interest
RT: room temperature
s.c.: subcutaneously
SAR: structure–activity relationship
SD: standard deviation
SUV: standardized uptake value
TAC: time-activity curve
TEA: triethylamine
TLC: thin-layer chromatography
TM: transmembrane helices
TPSA: topological polar surface area
TSPO: eighteen kDa translocator protein
VOI: volume of interest
*V*_T_: volume-of-distribution
WT: wild-type
