# Supplementary material for: Design, Synthesis and Preclinical Evaluation of a Brain-Permeable PET Tracer for P2Y12 Receptor Imaging in the Brain
Source: J Med Chem. 2025 Jul 25;68(15):15543–62. doi: 10.1021/acs.jmedchem.5c00457 (PMC12362594; doi:10.1021/acs.jmedchem.5c00457)

Supporting Information

Design, Synthesis and Preclinical Evaluation of a brain-permeable PET Tracer for P2Y12 Receptor Imaging in the Brain

Emanuel Joseph [1], Lea H. Kunze [1,2], Rebecca Schaefer [1], Giovanna Palumbo [1], Benjamin Kugelmann [1], Stephan Wagner [1], Sven Lammich [3], Regina Feederle [2,4,5], Michael Willem [3], Rudolf A. Werner [1,6], Matthias Brendel [1,2,5] and Simon Lindner [1,5]*

[1] Department of Nuclear Medicine, LMU University Hospital, LMU Munich, 81377 Munich, Germany

[2] German Center for Neurodegenerative Diseases (DZNE), 81377 Munich, Germany

[3] Biomedical Center Munich (BMC), LMU Munich, 81377 Munich, Germany

[4] Core Facility Monoclonal Antibodies, Helmholtz Munich, German Research Center for Environmental Health, 85764 Neuherberg, Germany

[5] Munich Cluster for Systems Neurology (SyNergy), 81377 Munich, Germany

[6] Russell H. Morgan Department of Radiology and Radiological Sciences, Johns Hopkins School of Medicine, Baltimore, 21287 Maryland, United States

**Corresponding author email address:* simon.lindner@med.uni-muenchen.de

Content

Docking results and *in silico* brain permeability prediction 3

IC_{50} Value 8

HPLC Radiosynthesis 9

Stability test 10

Biodistribution data 11

PET Imaging 11

Immunohistochemistry 13

Metabolite studies 14

Representative HPLC Spectra for test compounds. 15

NMR spectra 19

Docking results and *in silico* brain permeability prediction

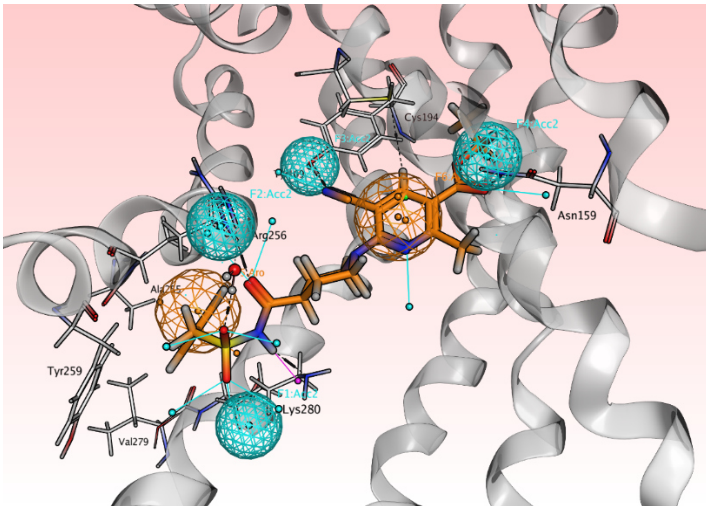

Figure S1. Pharmacophore for docking studies. 6 Features were defined based on central interactions: F1:Acc2 Lys280; F2:Acc2 Arg256; F3:Acc2 Tyr109; F4:Acc2 Asn159; F5:Aro Ar2; F6:Aro Ar1. (F = feature; Acc = hydrogen acceptor; Aro = aromatic interaction; Ar = aryl fragment).

Table S1. Docking Results. CNS MPO, BBB and S scores (RMSD = root mean square deviation).

Entry	Compound	Ar$_1$	Amino Linker	Ar$_2$	CNS MPO	BBB	S	RMSD
1	8	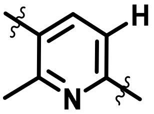	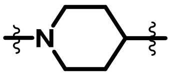	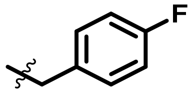	4.75	4.47	-9.23	1.22
2	14	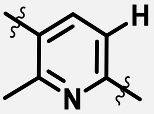	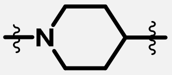	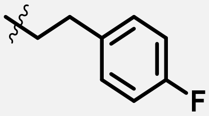	4.36	4.44	-9.66	1.39
3		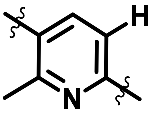	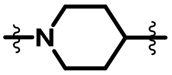	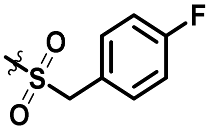	4.57	3.58	-10.23	1.78
4		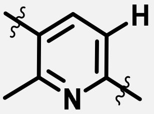	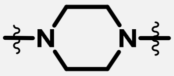	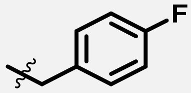	5.15	4.42	-9.21	1.34
5		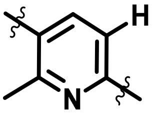	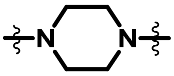	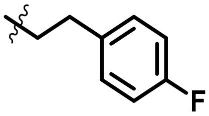	4.88	4.39	-9.54	1.09
6		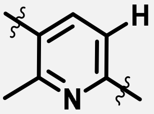	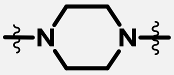	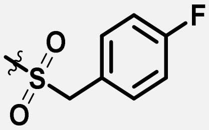	4.46	3.54	-9.30	1.46
7	11	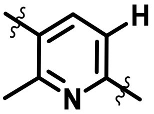	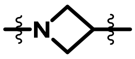	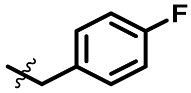	5.39	4.52	-9.14	1.04
8		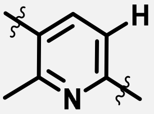	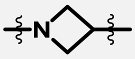	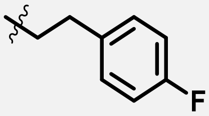	5.13	4.49	-9.55	0.96
9		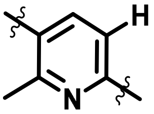	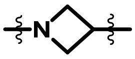	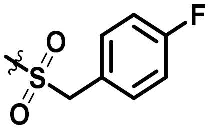	4.77	3.61	-9.83	1.24
10	9	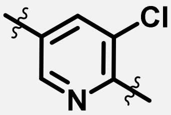	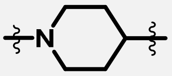	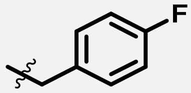	4.29	4.74	-9.52	1.27
11	15	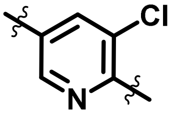	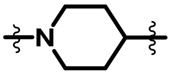	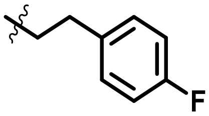	3.77	4.71	-9.92	1.34
12		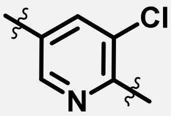	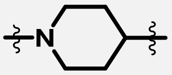	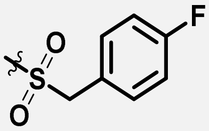	4.20	3.86	-9.82	1.97
13	6	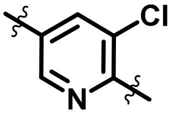	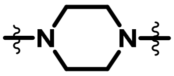	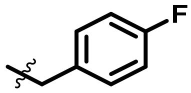	4.12	4.70	-9.19	0.80

14		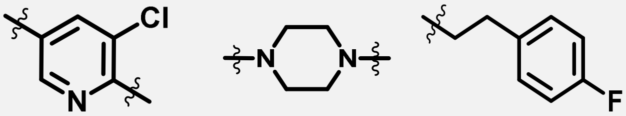	4.26	4.66	-9.36	1.10
15		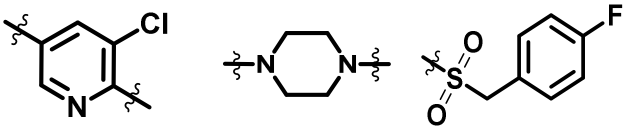	4.31	3.81	-9.81	1.42
16	12	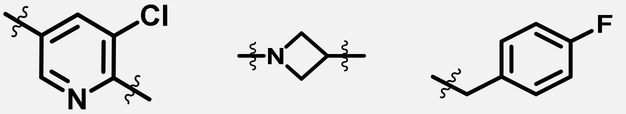	4.91	4.79	-9.26	1.20
17		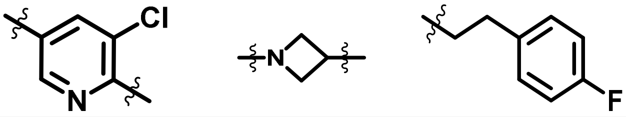	4.51	4.77	-9.55	0.94
18		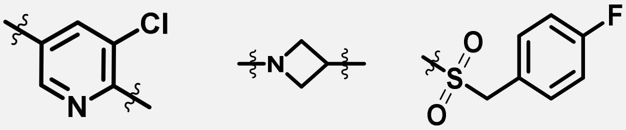	4.63	3.90	-9.88	1.23
19	18	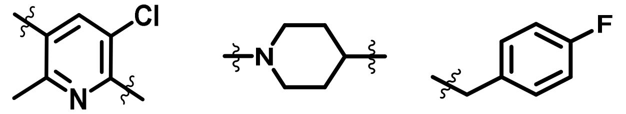	3.90	4.71	-9.79	1.24
20	17	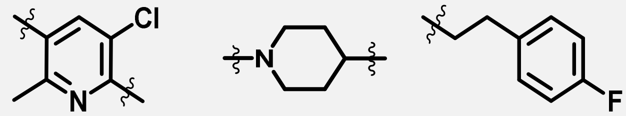	3.61	4.67	-10.15	1.59
21		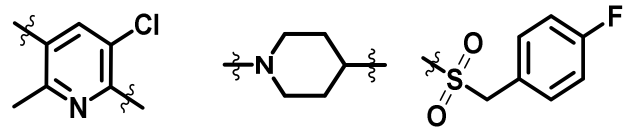	3.73	3.84	-10.56	1.70
22		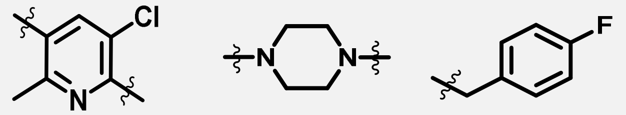	4.41	4.66	-9.57	1.13
23		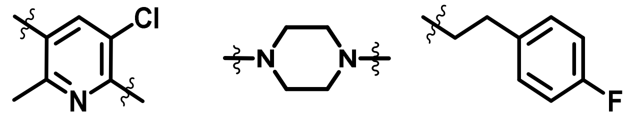	3.42	4.63	-9.80	1.47
24		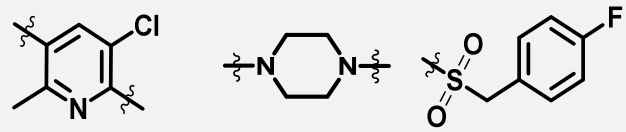	4.21	3.79	-9.79	1.37
25		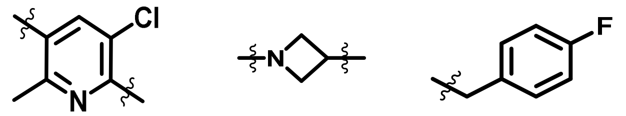	4.68	4.77	-9.69	0.88
26		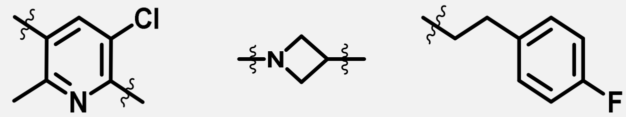	4.65	4.74	-9.94	1.23
27		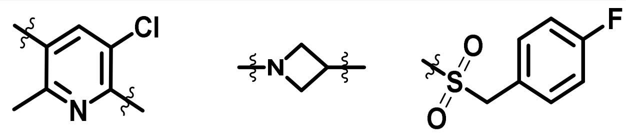	4.53	3.88	-9.91	1.47
28	10	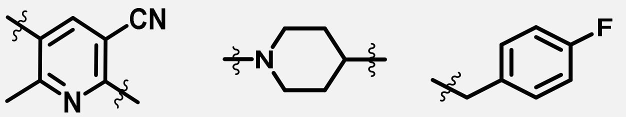	4.54	4.17	-10.03	1.67

29	16	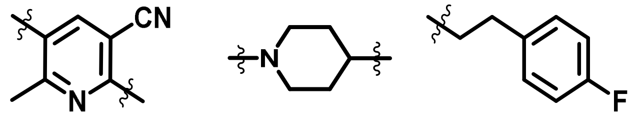	4.15	4.15	-10.44	1.02
30		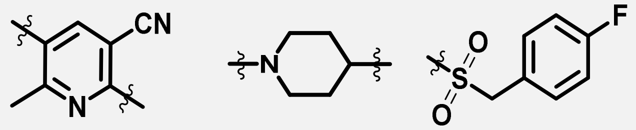	3.92	3.01	-10.86	1.64
31	5	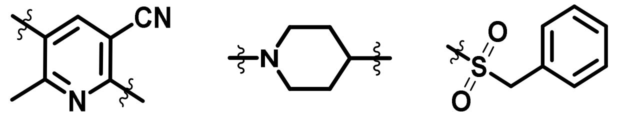	4.04	3.27	-10.63	1.12
32	7	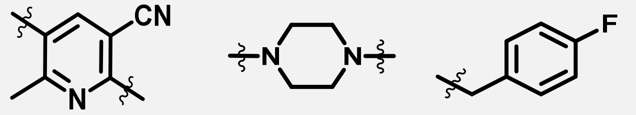	4.76	4.13	-9.80	1.04
33		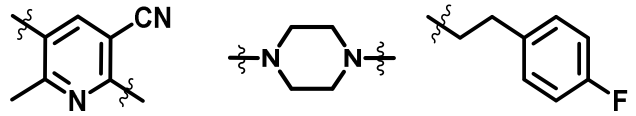	4.52	4.10	-9.64	0.89
34		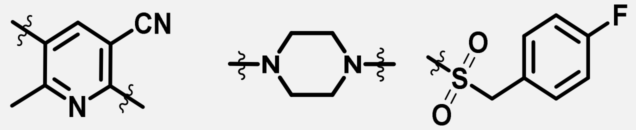	3.91	3.01	-10.34	1.05
35	13	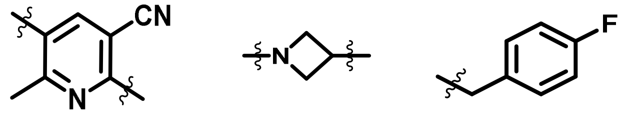	5.10	4.21	-9.91	1.58
36		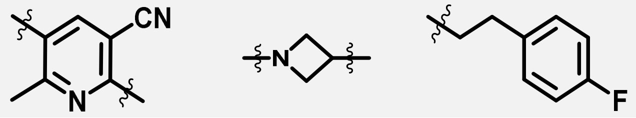	4.85	4.19	-9.95	1.06
37		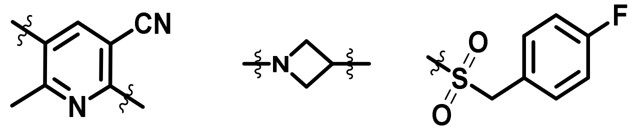	4.12	3.03	-10.59	1.03
38	19	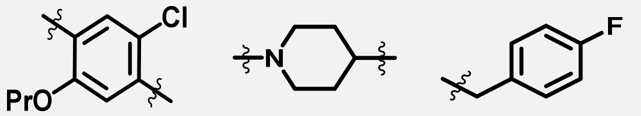	3.00	4.65	-10.58	0.99
39	20	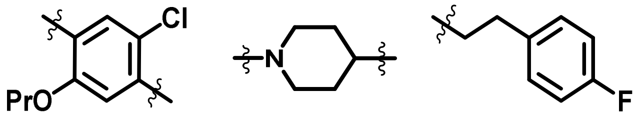	2.90	4.61	-11.03	1.22
40		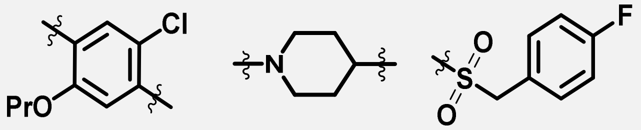	2.86	3.81	-10.26	3.20
41		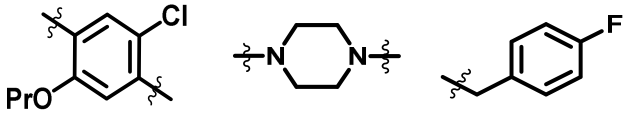	3.19	4.60	-10.58	1.07
42		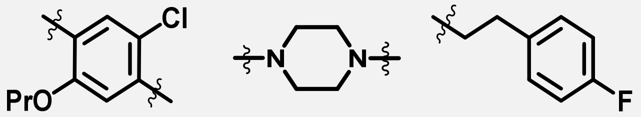	2.95	4.56	-10.51	1.00
43		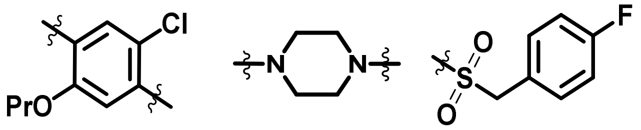	3.28	3.77	-10.78	1.85

44		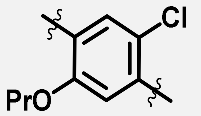	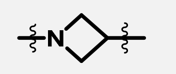	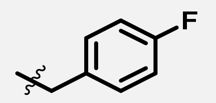	4.03	4.73	-10.49	1.23
45		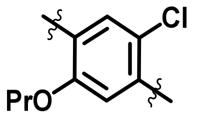	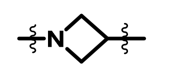	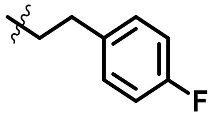	3.17	4.69	-10.80	1.26
46		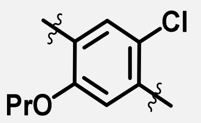	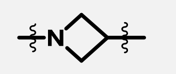	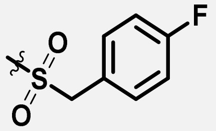	3.44	3.86	-10.75	1.16
47	22	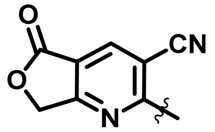	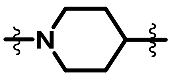	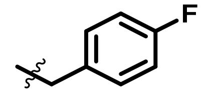	5.26	4.21	-8.91	1.35
48		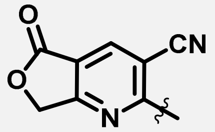	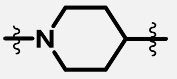	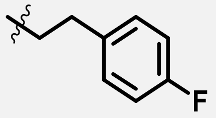	5.01	4.19	-8.04	0.79
49		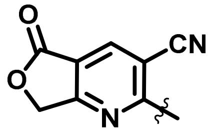	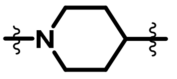	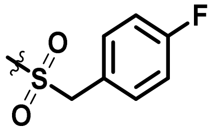	4.12	3.03	-7.84	1.22
50	21	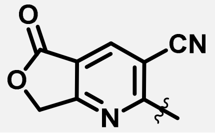	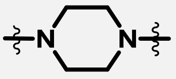	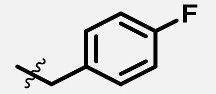	5.30	4.16	-8.82	1.48
51		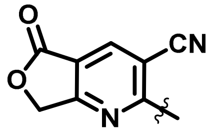	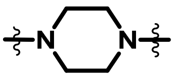	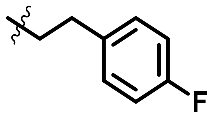	5.16	4.15	-9.18	1.07
52		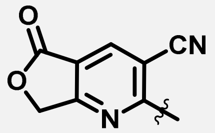	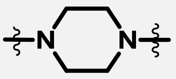	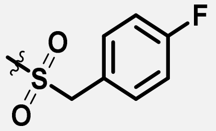	4.12	3.03	-9.25	2.03
53		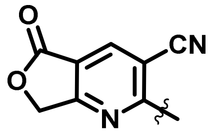	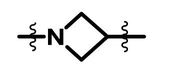	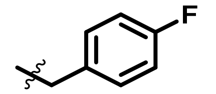	5.61	4.23	-8.76	1.71
54		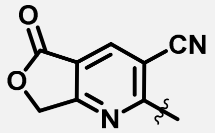	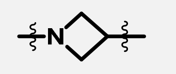	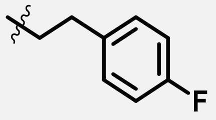	5.50	4.22	-8.86	1.44
55		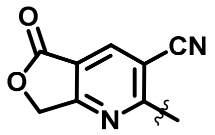	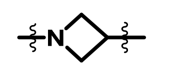	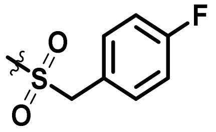	4.33	3.04	-8.91	1.22

IC$_{50}$ Value

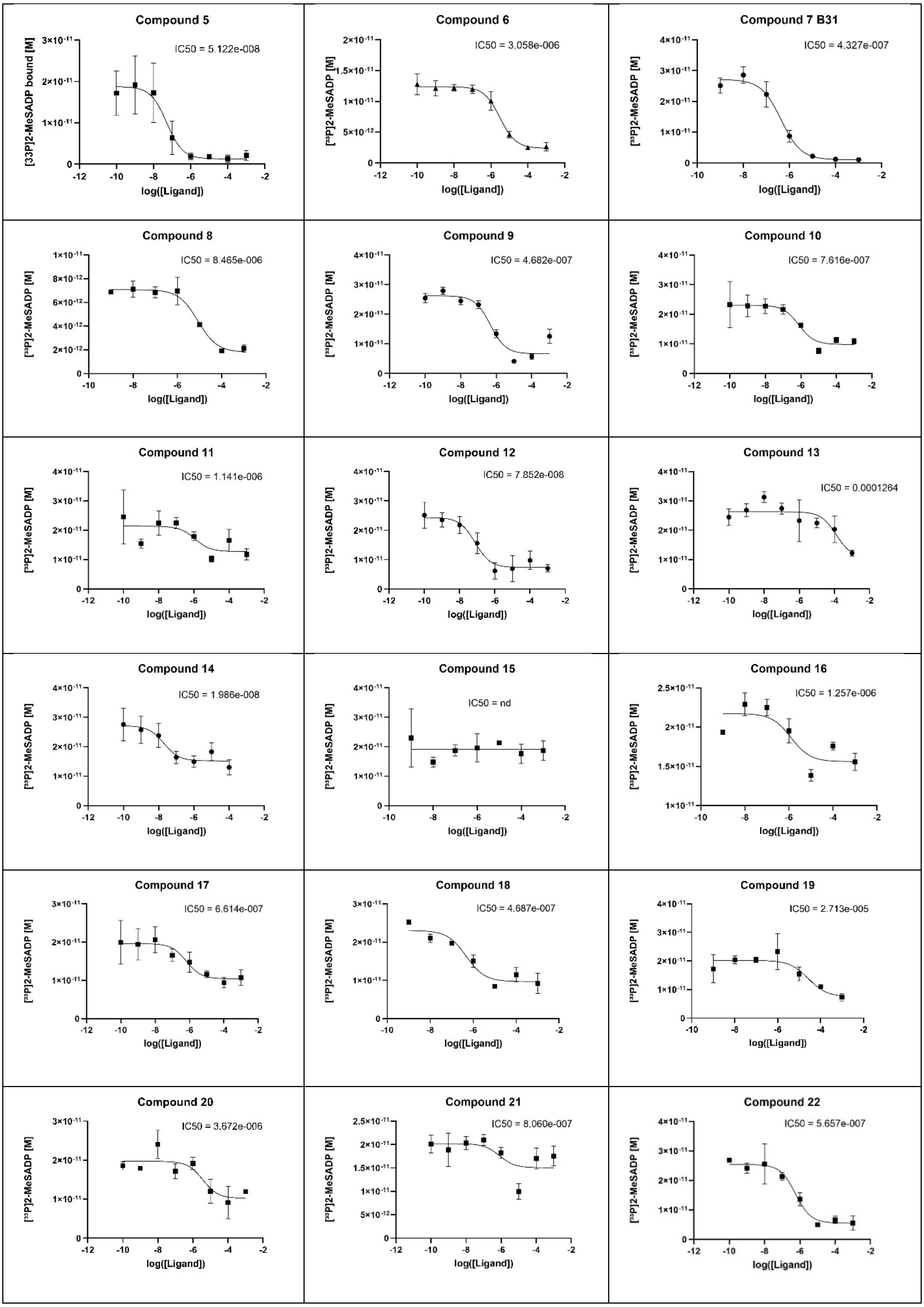

Figure S2. *In vitro* competitive binding curves for compounds **5-22**.

HPLC Radiosynthesis

HPLC Method A: Luna® 5 µm PFP(2) 100 Å, LC column 250 x 10 mm, 800 µl injection, crude reaction solution in EtOH, 0 – 15 min 48% H_2O (0.1% TFA), 52% MeCN (0.1% TFA), 5 mL/min.

HPLC Method B: Luna® 5 µm PFP(2) 100 Å, LC column 250 x 4.6 mm, 100 µl injection, product solution in PBS/EtOH (EtOH < 10%), 0 – 4 min 48% H_2O (0.1% TFA), 52% MeCN (0.1% TFA); 4 – 11 min 40% H_2O (0.1% TFA), 60% MeCN (0.1% TFA), 2 mL/min.

The purity of compound [^{18}F]**12** for *in vitro* and *in vivo* testing was confirmed to be ≥95%.

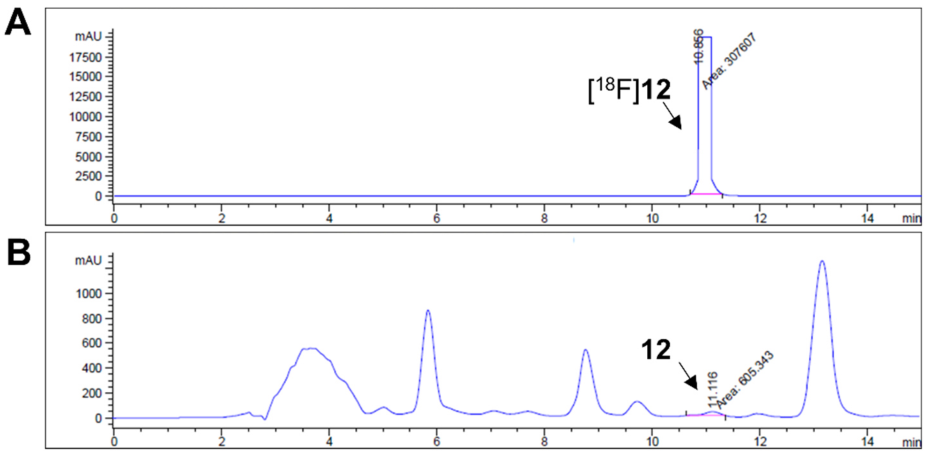

Figure S3. Preparative HPLC chromatogram of the crude reaction mixture of [^{18}F]**12**; (A) radio signal, (B) UV at 210 nm; HPLC method A.

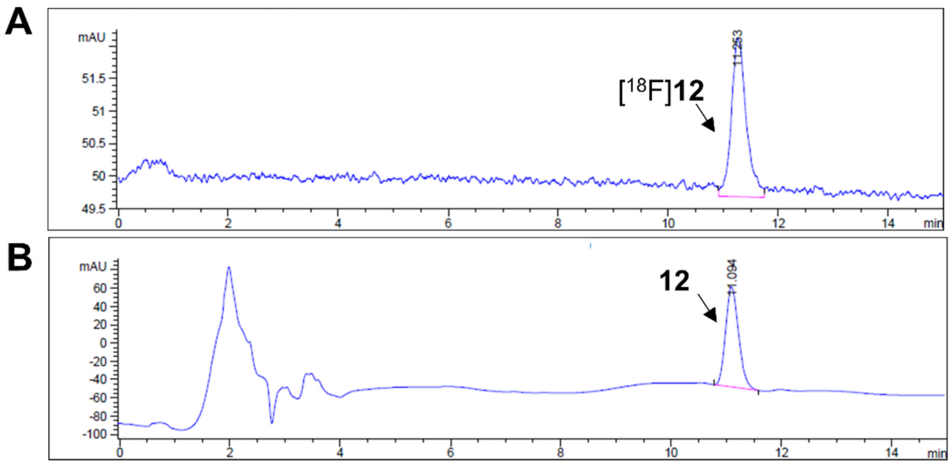

Figure S4. Preparative HPLC chromatogram of the product solution of [^{18}F]**12** with cold reference **12** (1 µg/ml); (A) radio signal, (B) UV at 210 nm; HPLC method A.

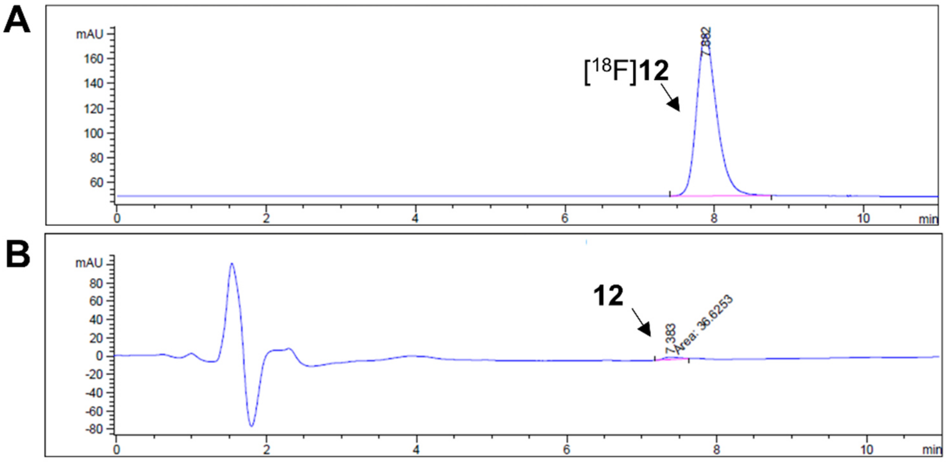

Figure S5. Analytical HPLC chromatogram of the product solution of [^{18}F]**12**; (A) radio signal, (B) UV at 210 nm; HPLC method B.

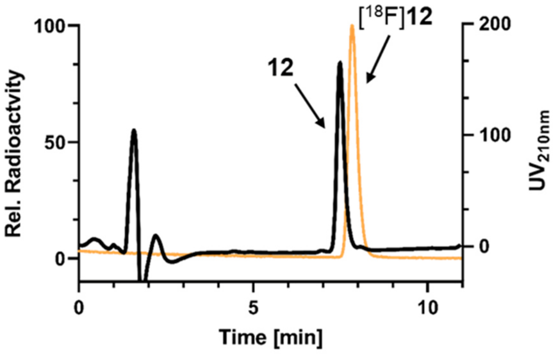

Figure S6. HPLC chromatogram of [^{18}F]**12** (rel. radioactivity, orange) co-injected with reference compound **12** (UV at 210 nm, black)

Stability test

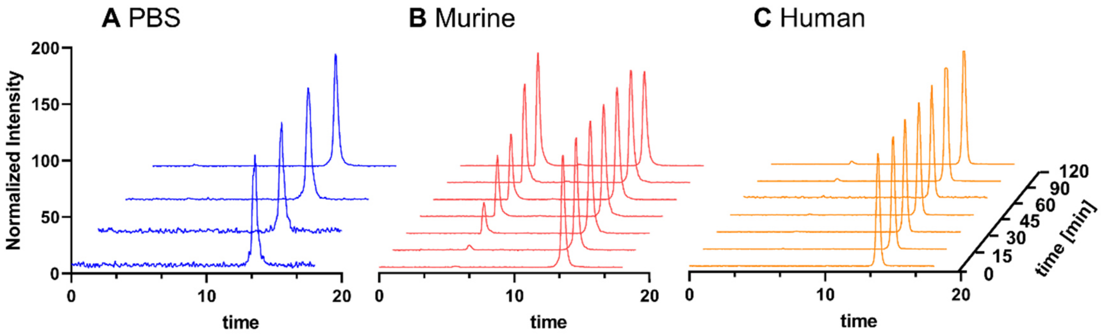

Figure S7. Stability of [^{18}F]**12** in (A) PBS, (B) murine plasma and (C) human plasma measured by radio-HPLC.

Biodistribution data

Table S2. Biodistribution of [^{18}F]**12** in WT mice 60 min p.i., data expressed as decay-corrected mean %ID/g ± SD.

**Entry**	**Organ**	**n**	**%ID/g**
1	Brain	12	0.61 ± 0.21
2	Blood	12	1.24 ± 0.56
3	Plasma	2	1.58 ± 0.78
4	Heart	2	1.30 ± 0.51
5	Lung	2	0.68 ± 0.15
6	Spleen	2	0.77 ± 0.19
7	Pancreas	2	0.79 ± 0.14
8	Kidney	2	5.51 ± 1.08
9	Liver	2	19.62 ± 4.75

PET Imaging

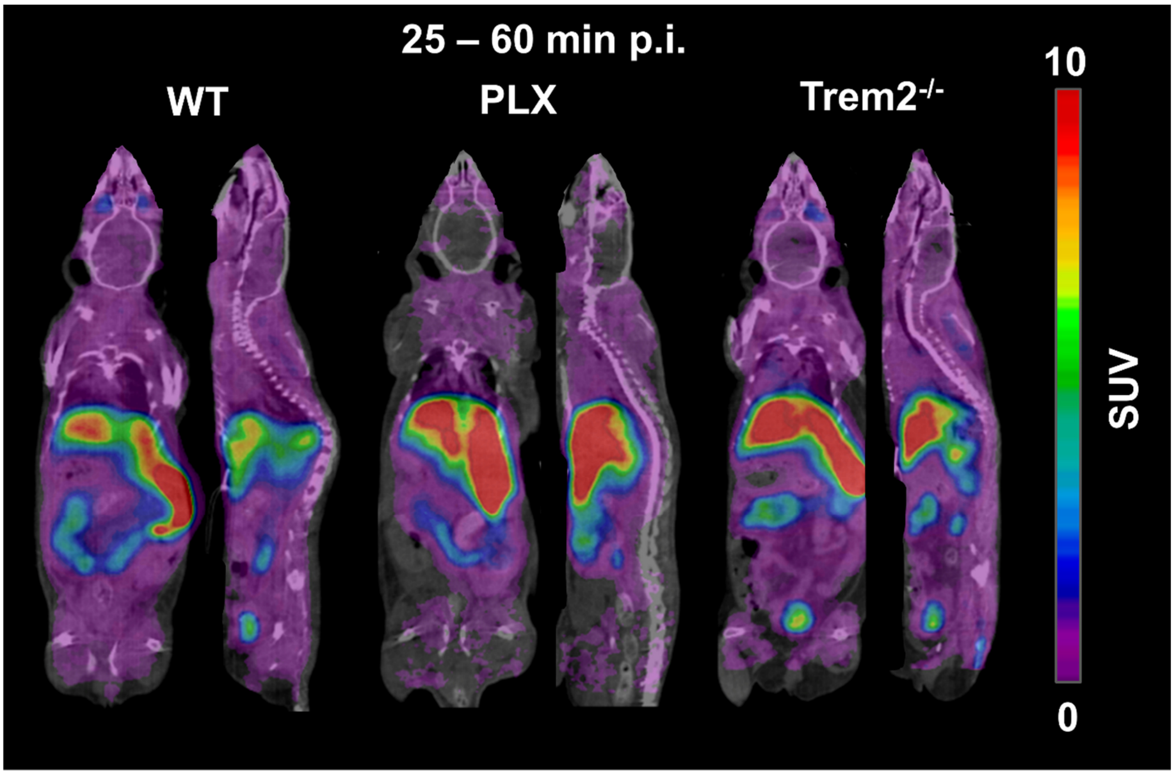

Figure S8. (A) Representative whole-body PET/CT image of WT, PLX and Trem2$^{-/-}$ mice, 25-60 min p.i.

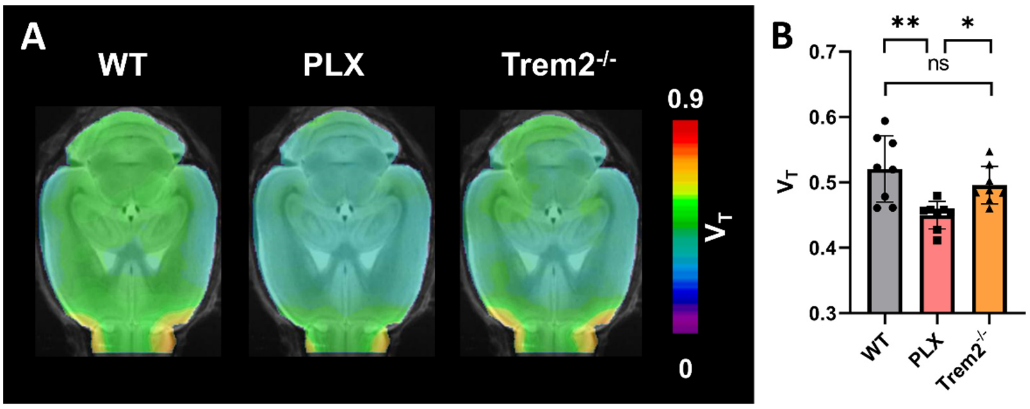

Figure S9. (A) Average V_T image of [^{18}F]**12** in WT, PLX and Trem2$^{-/-}$ mice (n = 8); (B) Tracer quantification of V_T whole brain, in WT, PLX treated and Trem2$^{-/-}$ mice. One-way ANOVA/Tukey's multiple comparisons test (F = 8.024, p = 0.0026), $p > 0.05$ (ns), $p \leq 0.05$ (*), $p \leq 0.01$ (**), mean ± SD.

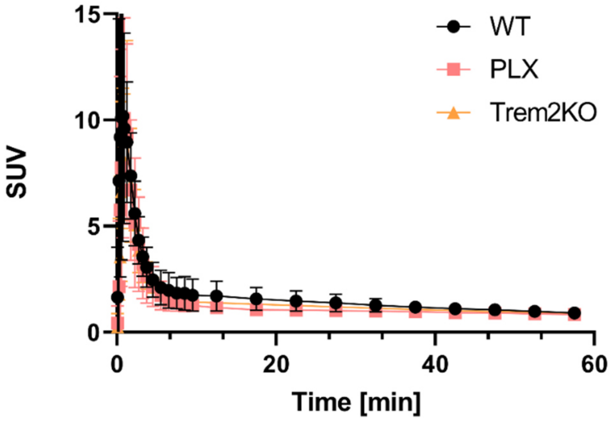

Figure S10. Average ventricle TAC of WT, PLX and Trem2$^{-/-}$ mice.

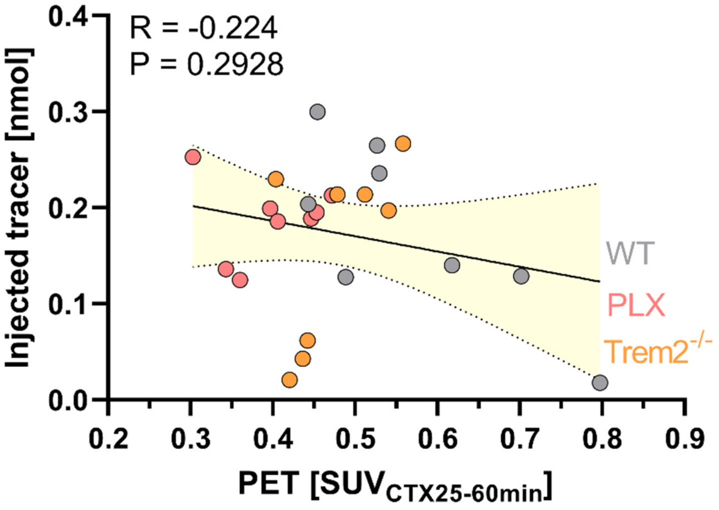

Figure S11. Correlation analysis of injected tracer amount and PET SUV; Linear regression, α = 0.05, 95% CI.

Immunohistochemistry

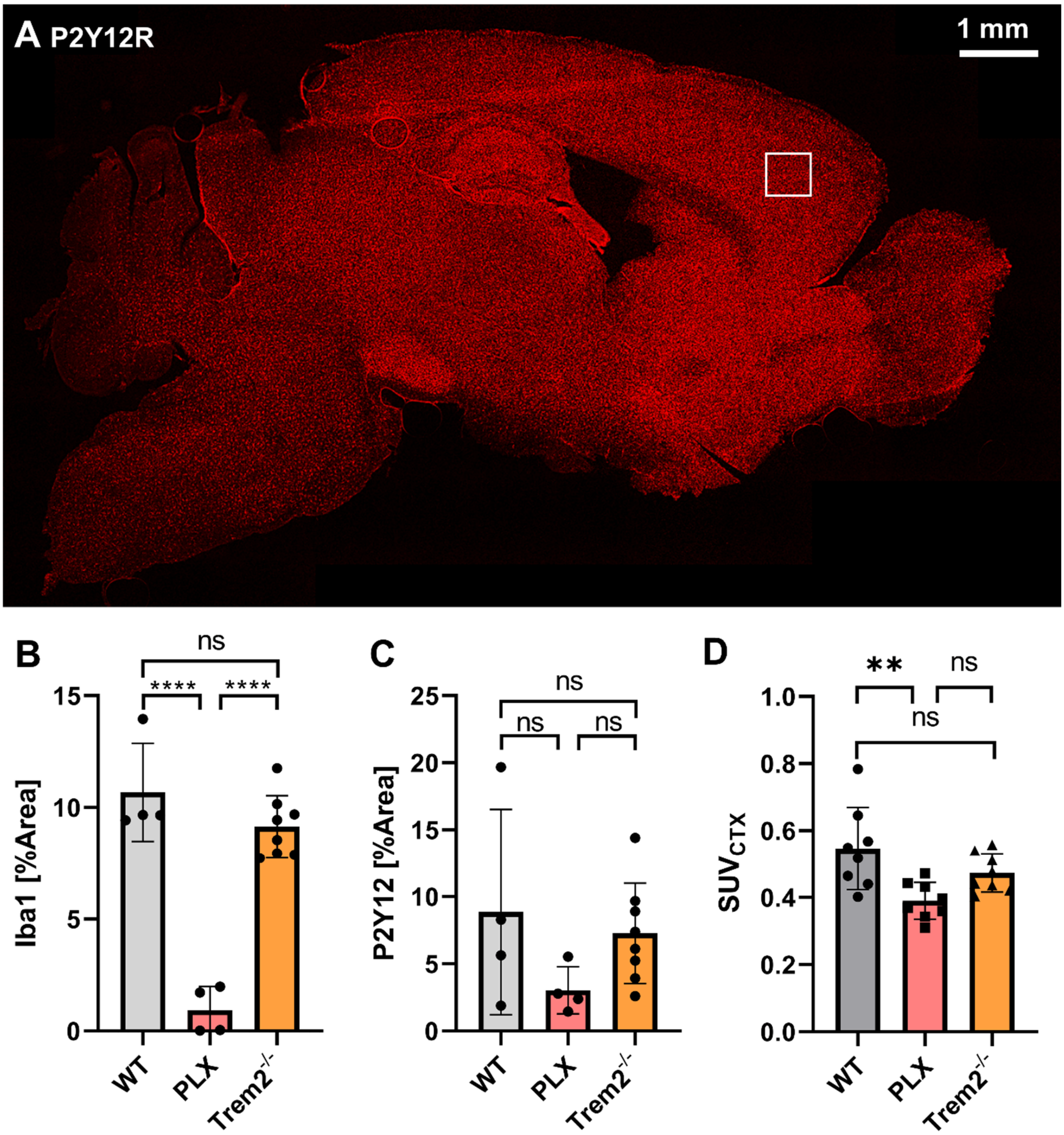

Figure S12. (A) IHC staining of the P2Y12R in a WT mouse; scale bar = 1 mm; the square represents the cortical area shown in the enlarged images in Figure 6A. (B) Iba1 immunofluorescence signal (%Area) in the CTX of WT, PLX and Trem2$^{-/-}$ mice. One-way ANOVA/Tukey's multiple comparisons test ($F = 48.56$, $p < 0.0001$); (C) P2Y12R immunofluorescence signal (%Area) in the CTX section of WT, PLX and Trem2$^{-/-}$ mice. One-way ANOVA/Tukey's multiple comparisons test ($F = 1.724$, $p = 0.2168$); (D) PET tracer quantification in the CTX of WT, PLX and Trem2$^{-/-}$ mice 25-60 min p.i. One-way ANOVA/Tukey's multiple comparisons test ($F = 6.792$, $p = 0.0053$), $p > 0.05$ (ns), $p \leq 0.05$ (*), $p \leq 0.01$ (**), $p \leq 0.001$ (***), $p \leq 0.0001$ (****), mean ± SD.

Metabolite studies

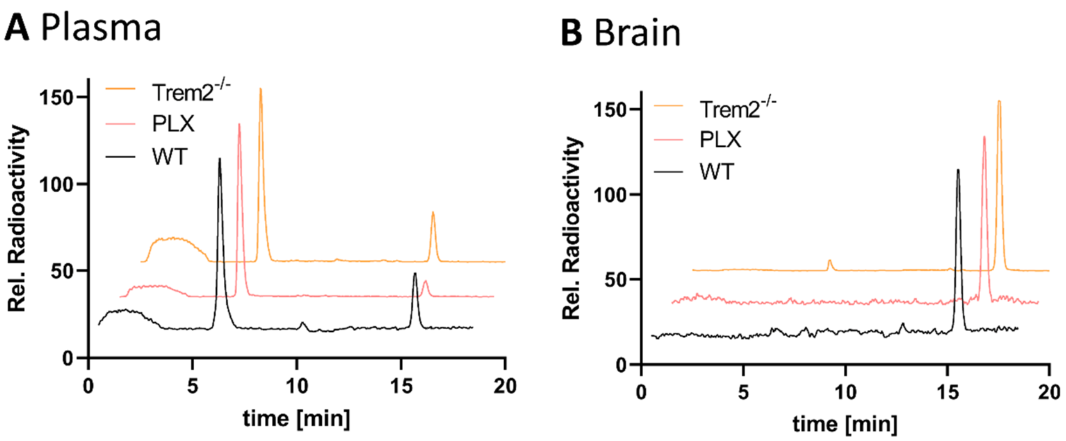

Figure S13. (A) Representative radio-HPLC chromatograms of plasma samples of WT, PLX and Trem2$^{-/-}$ mice 60 min p.i.; (B) Representative radio-HPLC chromatograms of brain samples of WT, PLX and Trem2$^{-/-}$ mice 60 min p.i.

Representative HPLC Spectra for test compounds.

HPLC Method C: Luna 5 µm PFP(2) 100 Å, LC Column 250 x 4.6 mm, 25 µl injection, 1 mM test compound, solution in DMSO, 0 – 4 min 48% H_2O (0.1% TFA), 52% MeCN (0.1% TFA), 2 ml/min; 4 – 11 min 40% H_2O (0.1% TFA), 60% MeCN (0.1% TFA), 2 ml/min.

HPLC Method D: Luna 5 µm PFP(2) 100 Å, LC Column 250 x 4.6 mm, 25 µl injection, 1 mM test compound, solution in DMSO, 0 – 4 min 65% H_2O (0.1% TFA), 35% MeCN (0.1% TFA), 2 ml/min; 4 – 11 min 50% H_2O (0.1% TFA), 50% MeCN (0.1% TFA), 2 ml/min.

The purity of compounds for *in vitro* and *in vivo* testing was confirmed to be ≥95%.

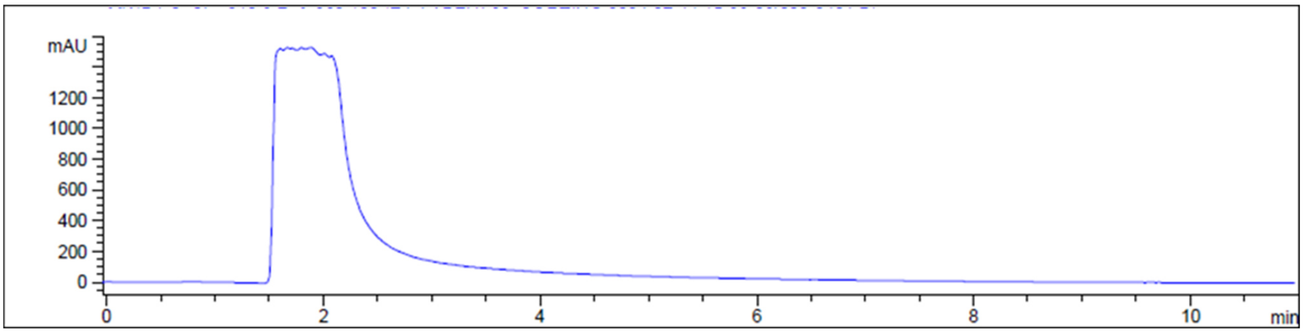

Figure S14. Analytical HPLC chromatogram of DMSO blank (UV 210 nm, HPLC method C).

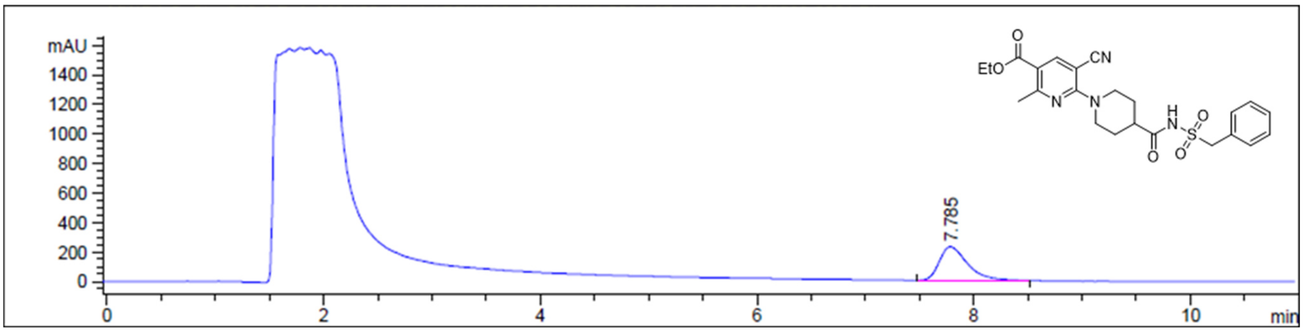

Peak #	RetTime [min]	Type	Width [min]	Area [mAU*s]	Height [mAU]	Area %
1	7.785	BB	0.2813	4176.67480	228.45567	100.0000
Totals :				4176.67480	228.45567	

Figure S15. Analytical HPLC chromatogram of compound **5** AZD1283 (UV 210 nm, HPLC method C).

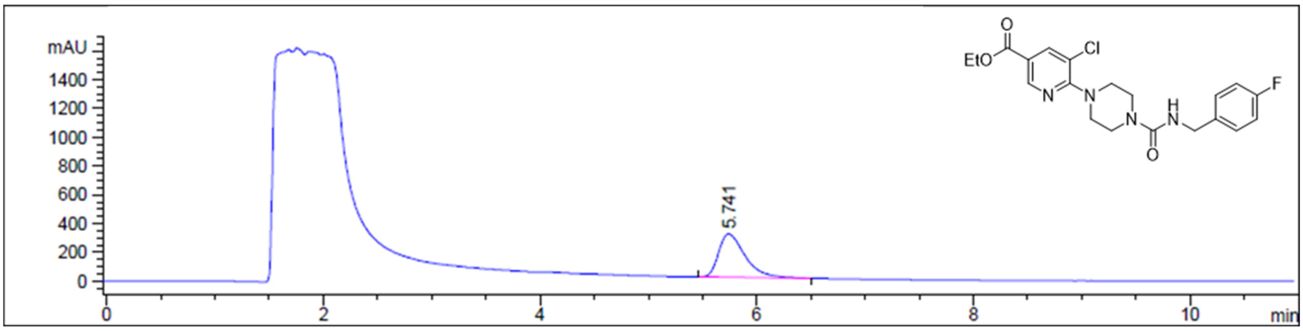

Peak #	RetTime [min]	Type	Width [min]	Area [mAU*s]	Height [mAU]	Area %
1	1.695	BV	0.4778	5.18597e4	1812.74500	100.0000
Totals :				5.18597e4	1812.74500	

Figure S16. Analytical HPLC chromatogram of compound **6** (UV 210 nm, HPLC method C).

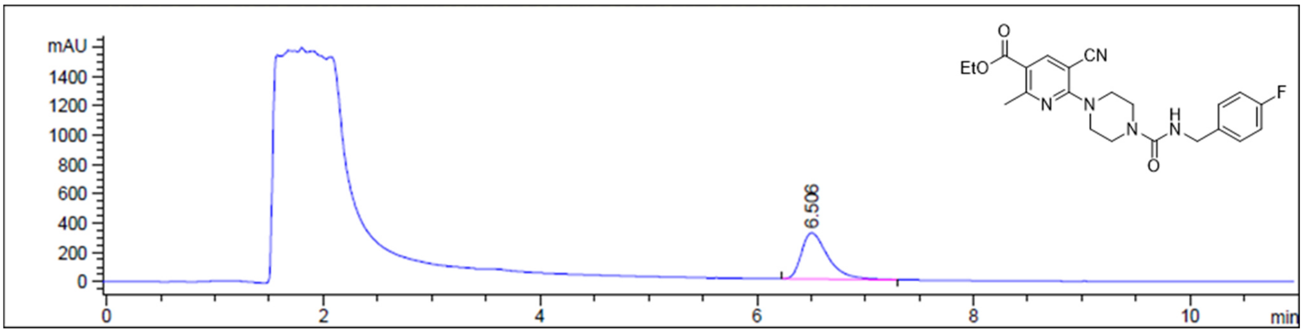

Peak #	RetTime [min]	Type	Width [min]	Area [mAU*s]	Height [mAU]	Area %
1	6.506	BB	0.2531	5402.88330	316.57773	100.0000
Totals :				5402.88330	316.57773	

Figure S17. Analytical HPLC chromatogram of compound **7** (UV 210 nm, HPLC method C).

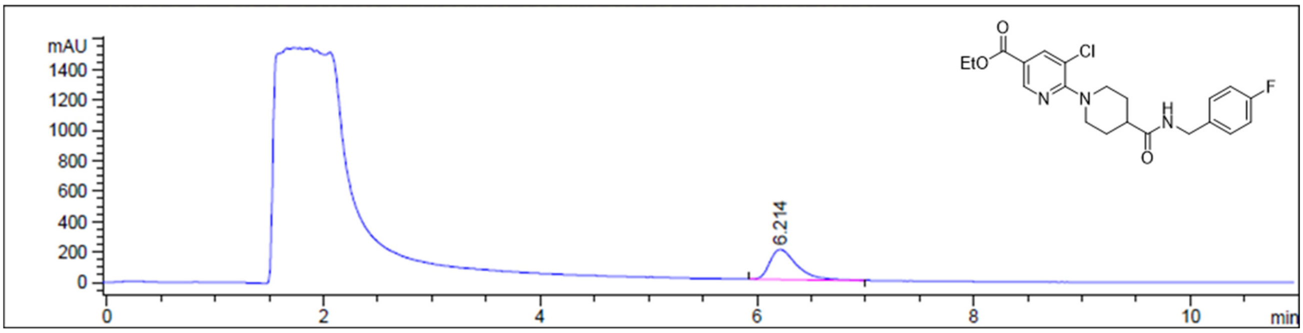

Peak #	RetTime [min]	Type	Width [min]	Area [mAU*s]	Height [mAU]	Area %
1	6.214	BB	0.2590	3443.54883	197.85811	100.0000
Totals :				3443.54883	197.85811	

Figure S18. Analytical HPLC chromatogram of compound **10** (UV 210 nm, HPLC method C).

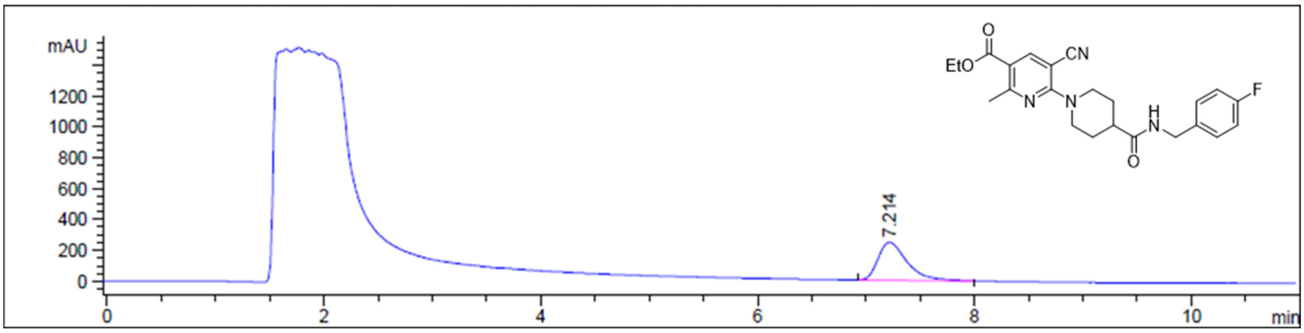

Peak #	RetTime [min]	Type	Width [min]	Area [mAU*s]	Height [mAU]	Area %
1	7.214	BB	0.2794	4621.49854	247.97662	100.0000
Totals :				4621.49854	247.97662	

Figure S19. Analytical HPLC chromatogram of compound **12** (UV 210 nm, HPLC method C).

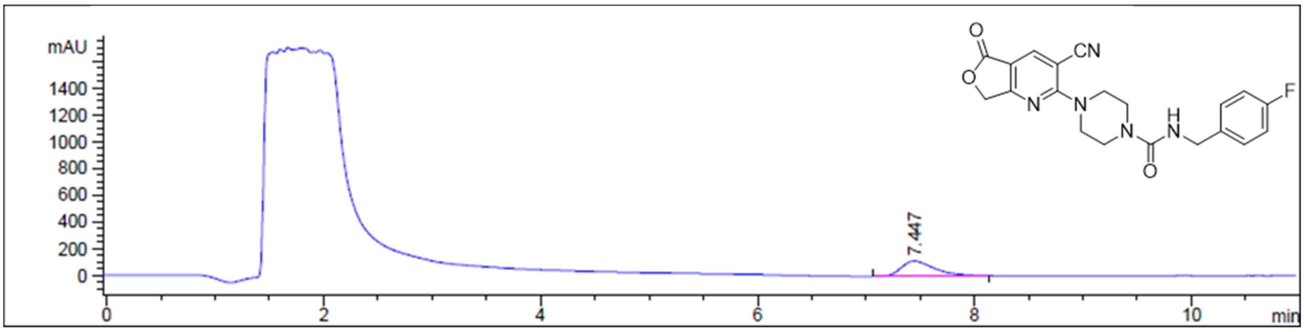

Peak #	RetTime [min]	Type	Width [min]	Area [mAU*s]	Height [mAU]	Area %
1	7.447	BB	0.3217	2460.90088	113.18052	100.0000
Totals :				2460.90088	113.18052	

Figure S20. Analytical HPLC chromatogram of compound **14** (UV 210 nm, HPLC method D).

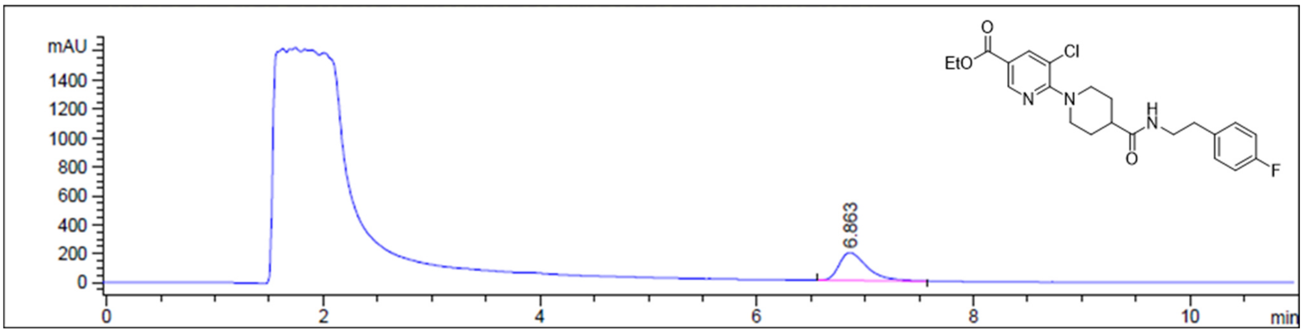

Peak #	RetTime [min]	Type	Width [min]	Area [mAU*s]	Height [mAU]	Area %
1	7.791	BB	0.2716	4540.38086	250.36887	100.0000
Totals :				4540.38086	250.36887	

Figure S21. Analytical HPLC chromatogram of compound **16** (UV 210 nm, HPLC method C).

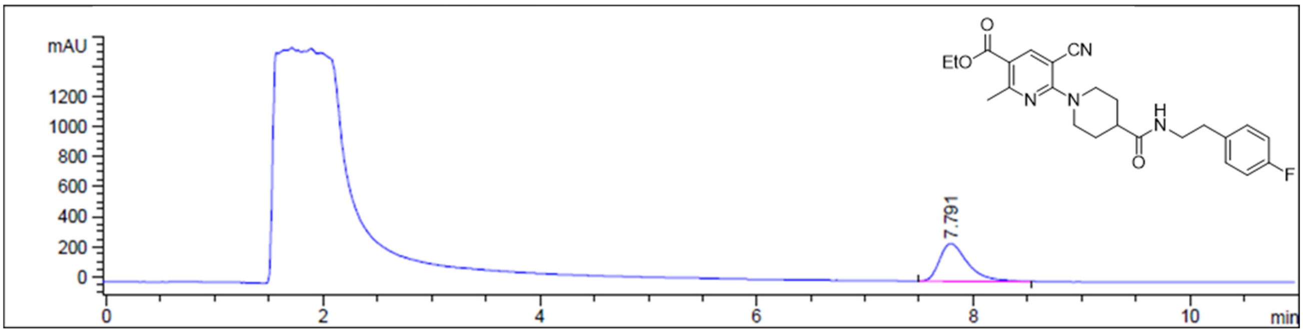

Peak #	RetTime [min]	Type	Width [min]	Area [mAU*s]	Height [mAU]	Area %
1	6.863	BB	0.2700	3486.42261	193.72156	100.0000
Totals :				3486.42261	193.72156	

Figure S22. Analytical HPLC chromatogram of compound **18** (UV 210 nm, HPLC method C).

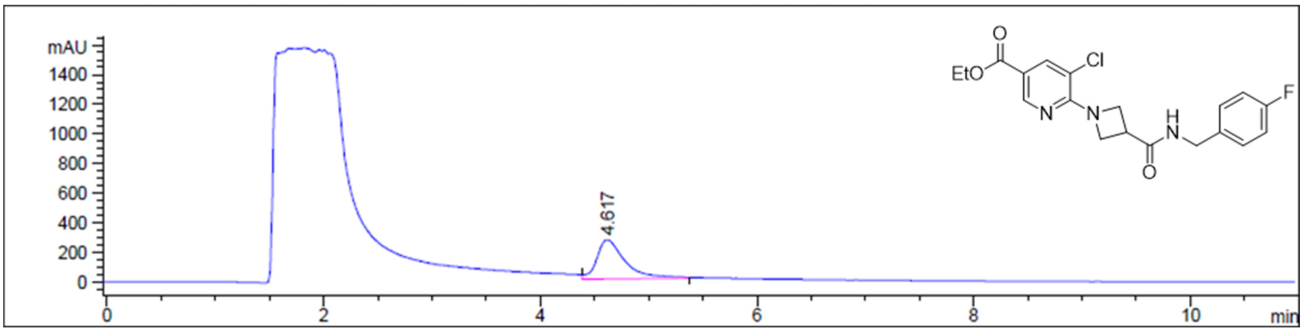

Peak #	RetTime [min]	Type	Width [min]	Area [mAU*s]	Height [mAU]	Area %
1	4.617	BB	0.2664	4829.04590	265.20636	100.0000
Totals :				4829.04590	265.20636	

Figure S23. Analytical HPLC chromatogram of compound **21** (UV 210 nm, HPLC method C).

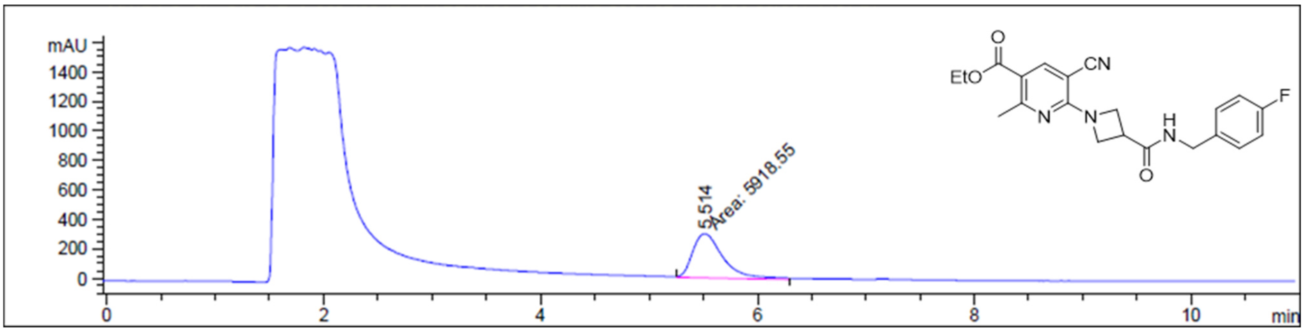

Peak #	RetTime [min]	Type	Width [min]	Area [mAU*s]	Height [mAU]	Area %
1	5.514	MM	0.3275	5918.55078	301.21487	100.0000
Totals :				5918.55078	301.21487	

Figure S24. Analytical HPLC chromatogram of compound **22** (UV 210 nm, HPLC method C).

NMR spectra

*Ethyl 5-chloro-6-(4-((4-fluorobenzyl)carbamoyl)piperazin-1-yl)nicotinate (**6**)*

^{1}H NMR (400 MHz, $CDCl_3$) and **^{13}C NMR** (100 MHz, $CDCl_3$)

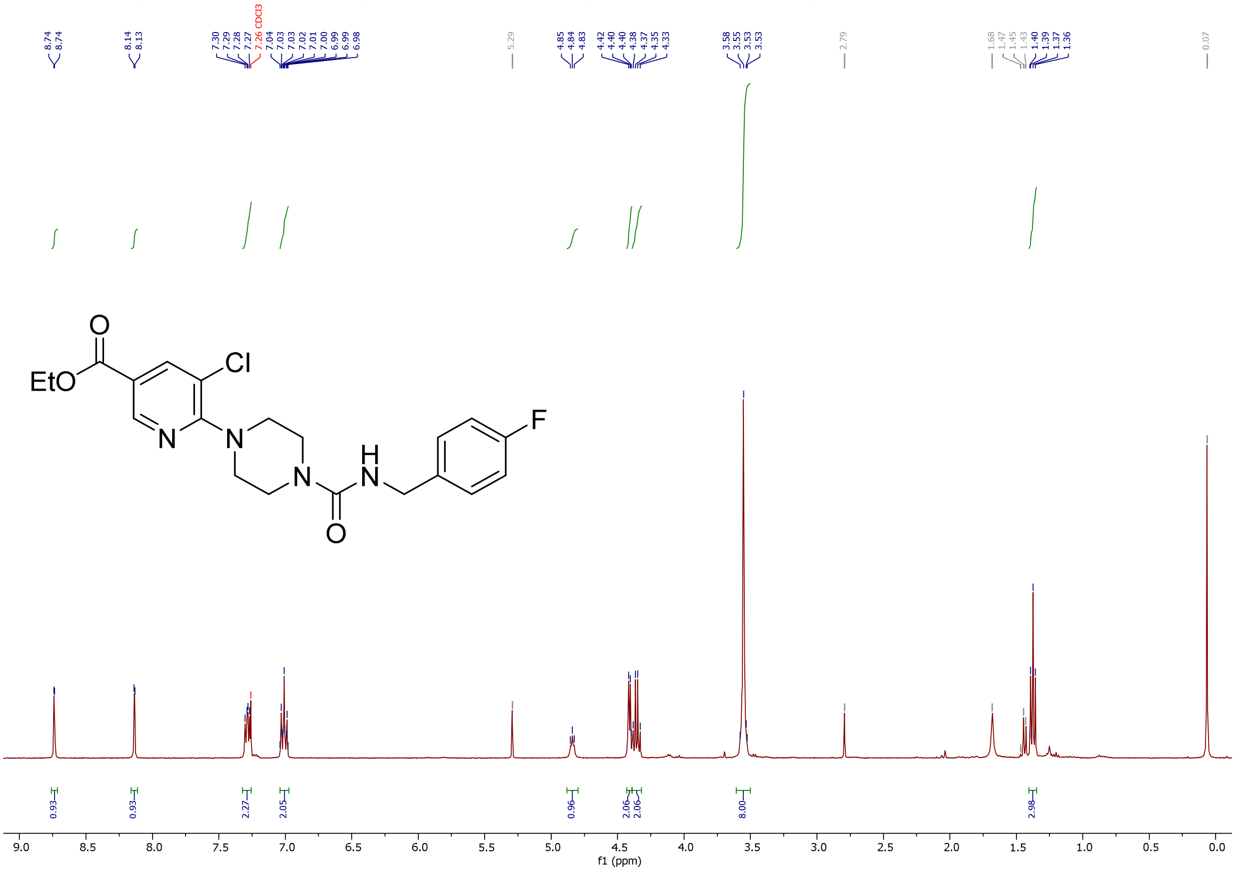

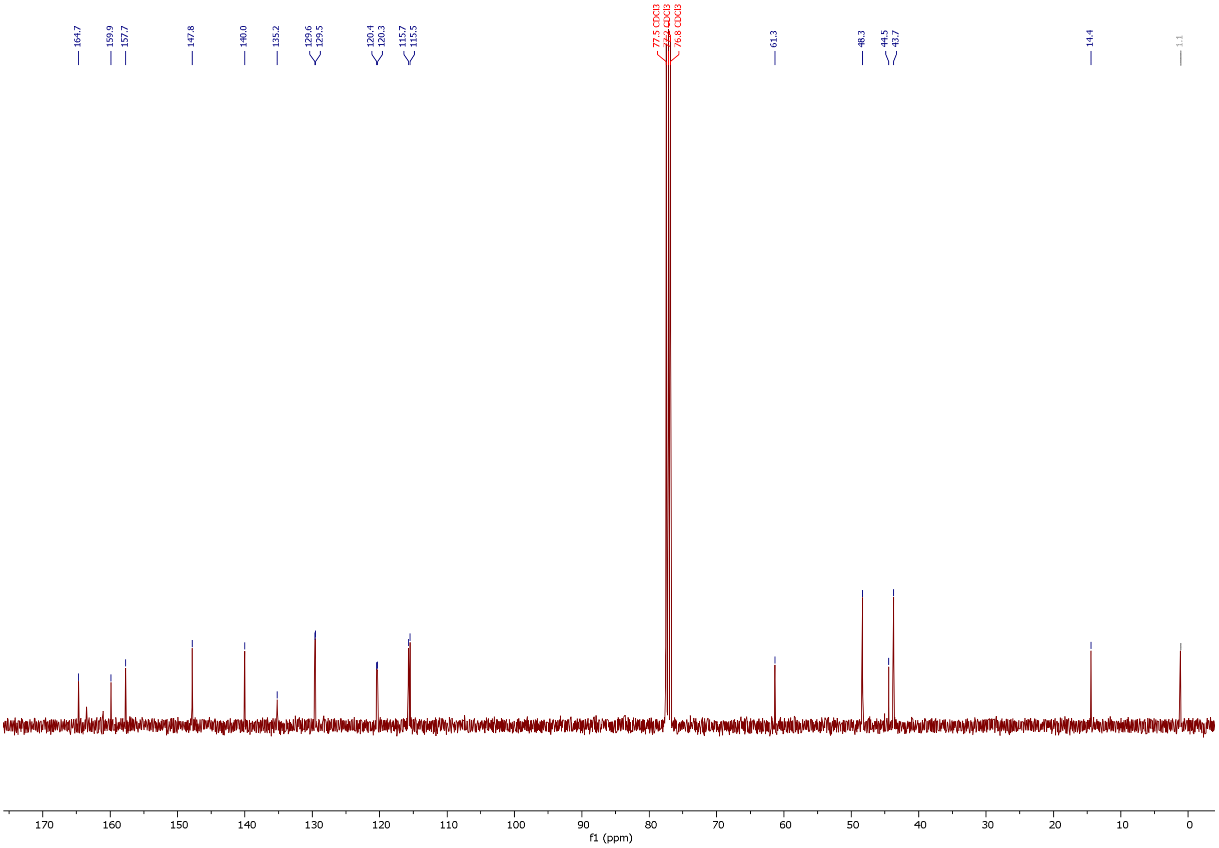

Ethyl 5-cyano-6-(4-((4-fluorobenzyl)carbamoyl)piperazin-1-yl)-2-methylnicotinate (7)

^{1}H NMR (400 MHz, $CDCl_3$) and **^{13}C NMR** (100 MHz, $CDCl_3$)

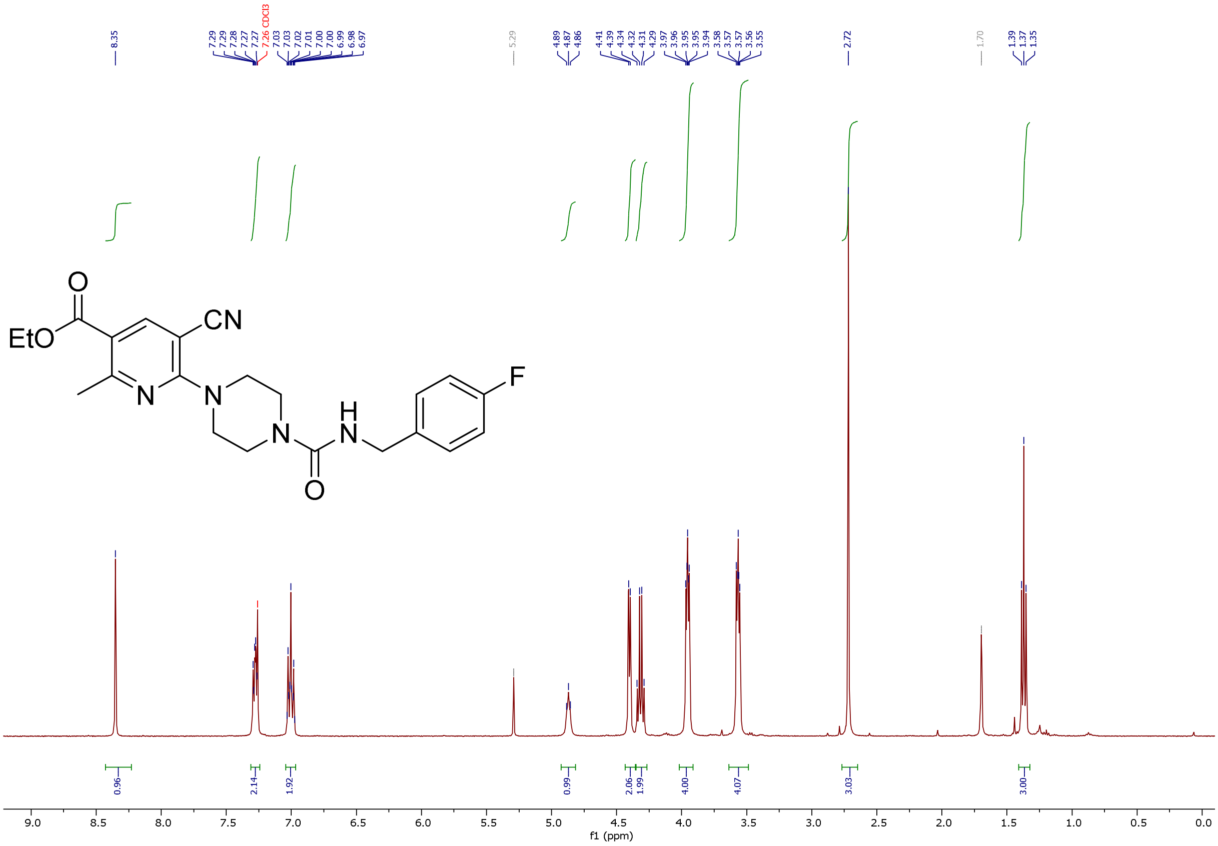

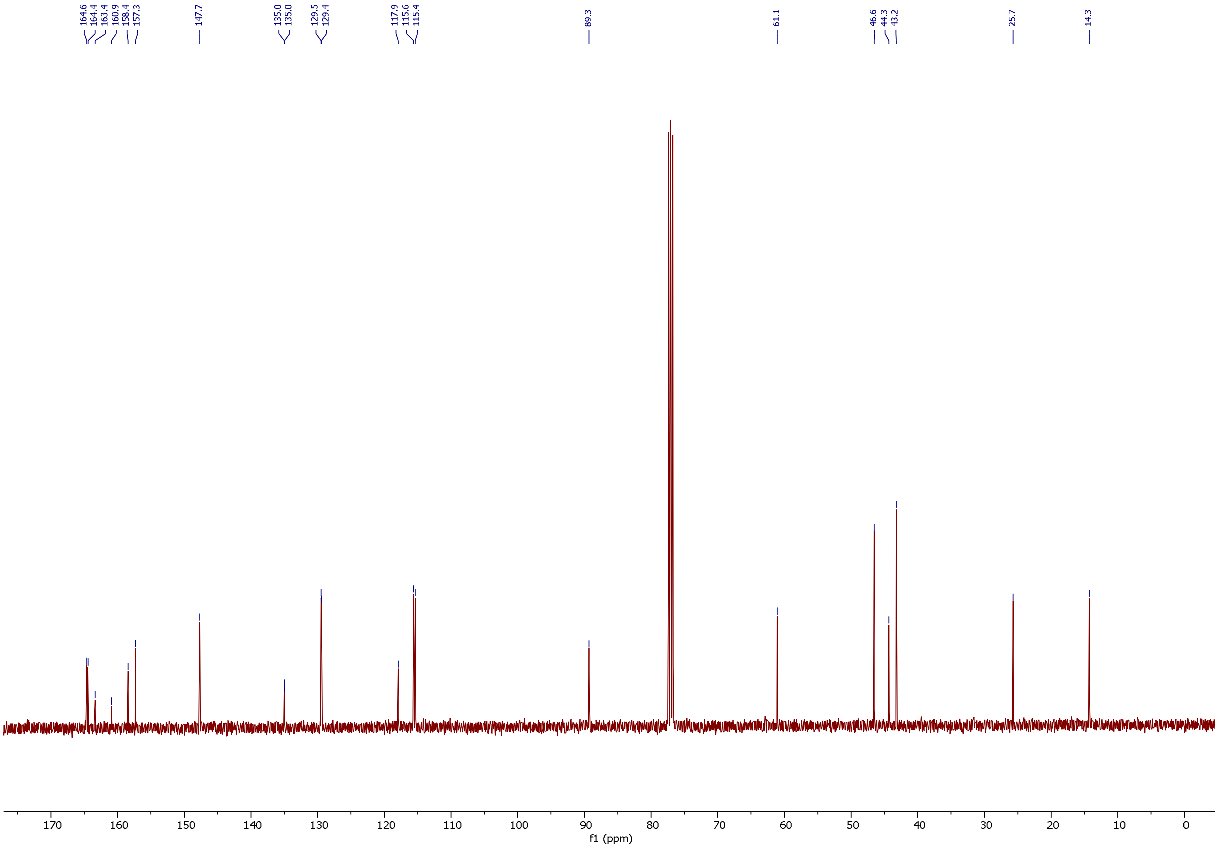

*1-(3-Cyano-5-oxo-5,7-dihydrofuro[3,4-b]pyridin-2-yl)-N-(4-fluorobenzyl)piperidine-4-carboxamide (**8**)*

^{1}H NMR (400 MHz, $CDCl_3$) and **^{13}C NMR** (100 MHz, $CDCl_3$)

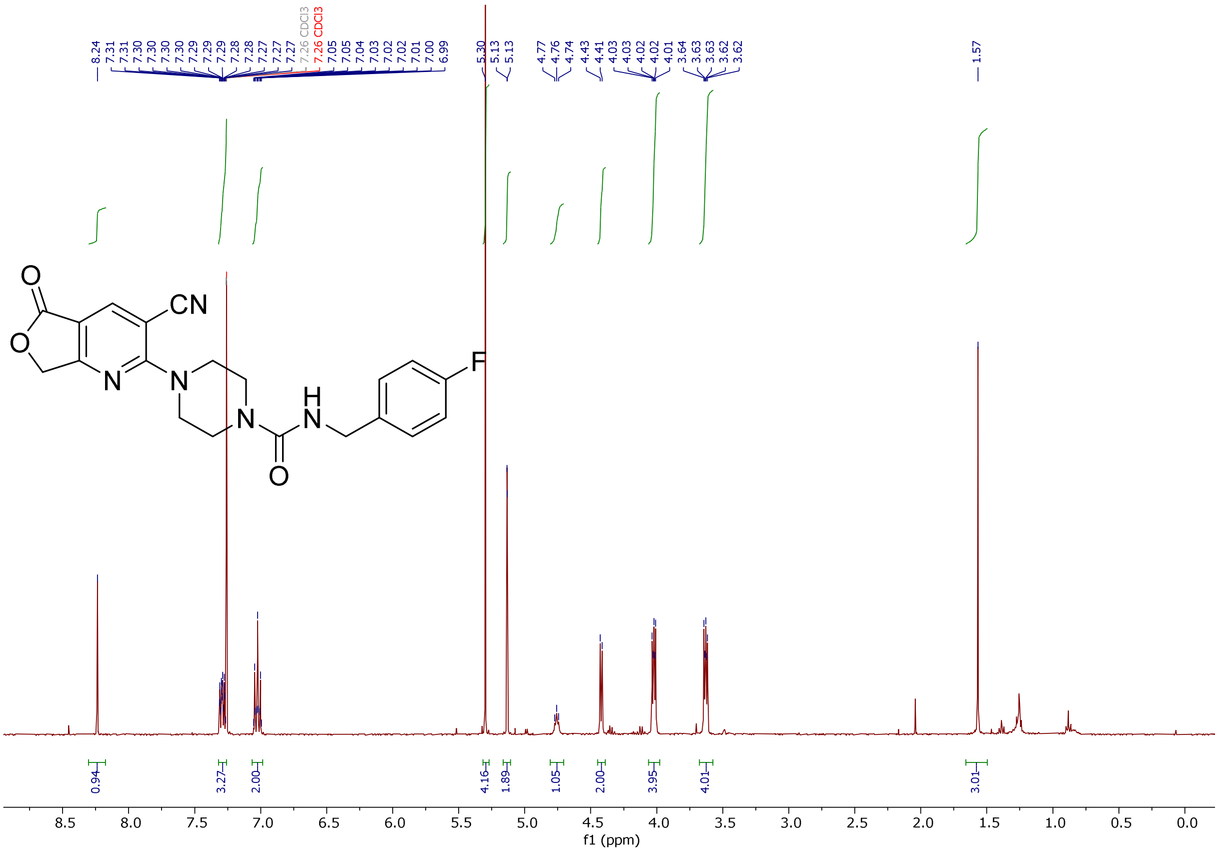

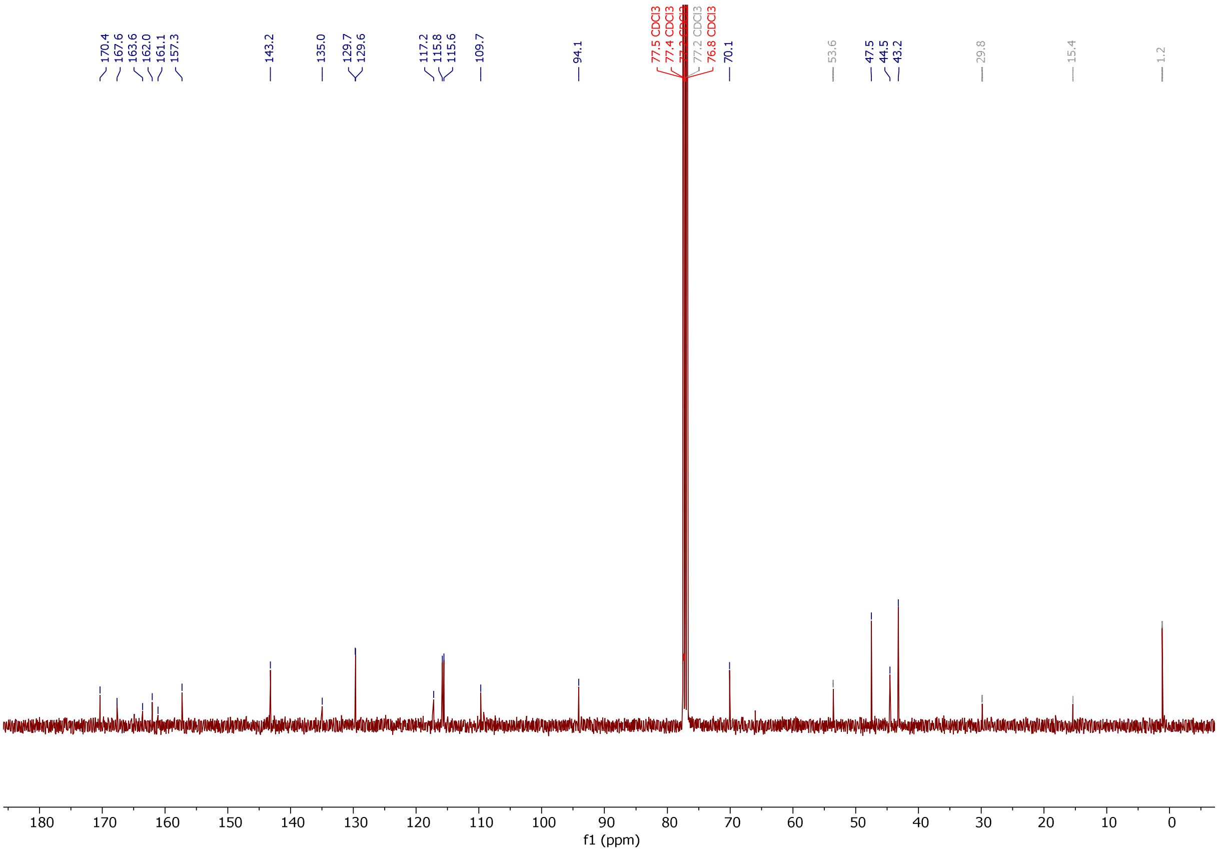

*Ethyl 6-(4-((4-fluorobenzyl)carbamoyl)piperidin-1-yl)-2-methylnicotinate (**9**)*

^{1}H NMR (400 MHz, $CDCl_3$) and **^{13}C NMR** (100 MHz, $CDCl_3$)

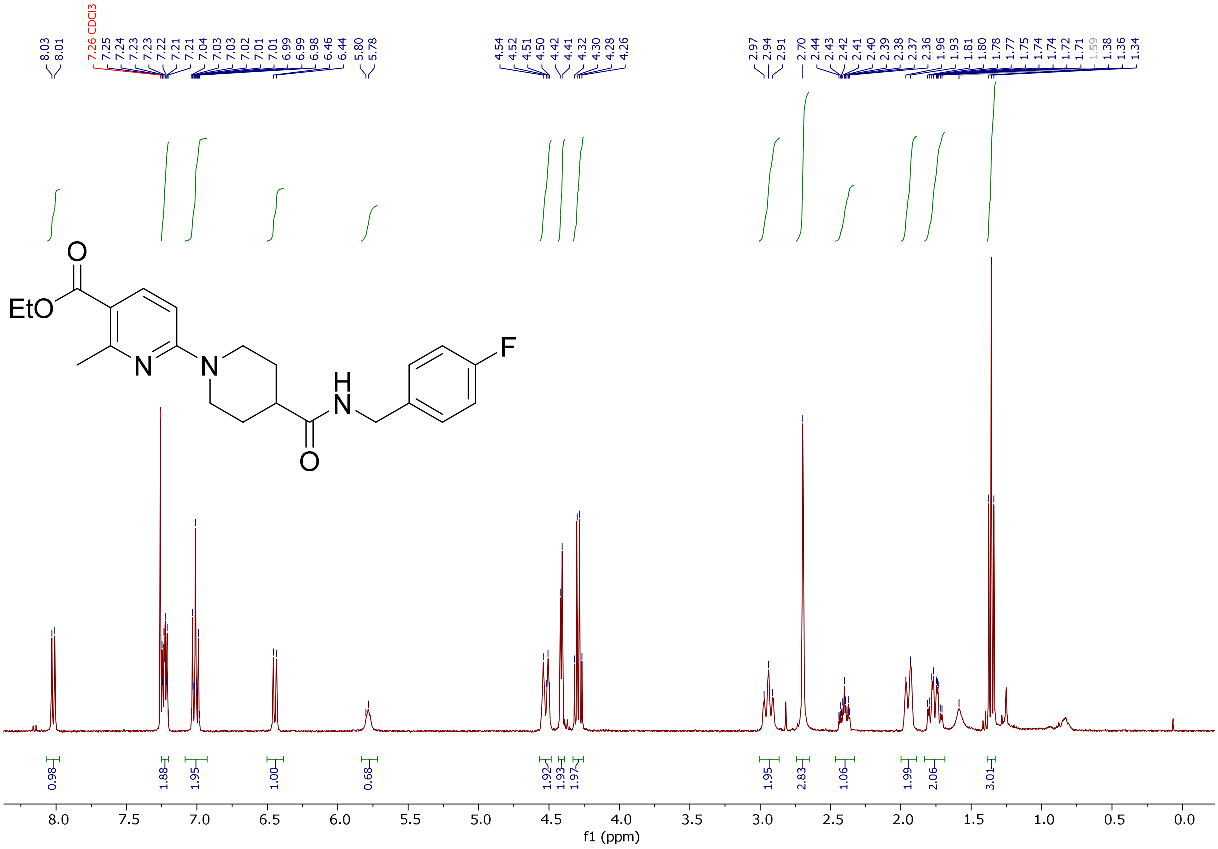

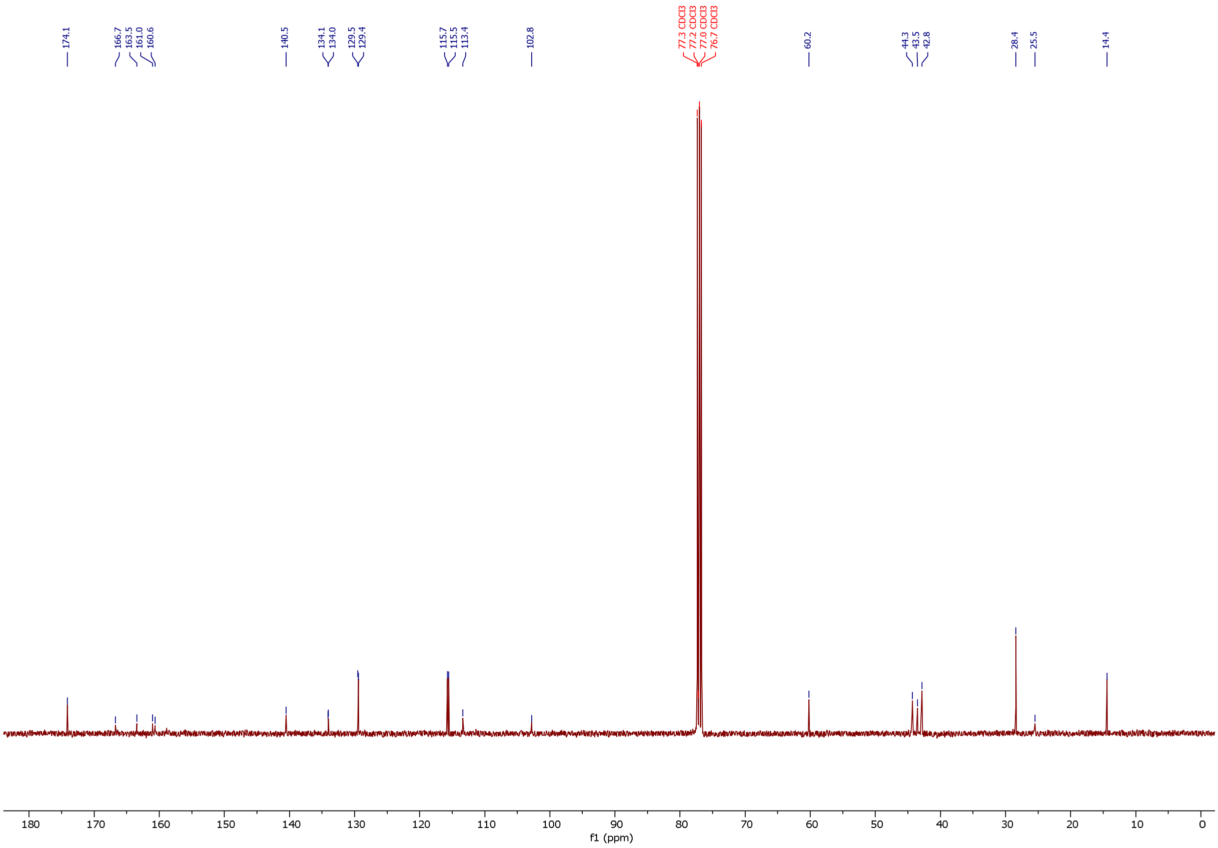

*Ethyl 5-chloro-6-(4-((4-fluorobenzyl)carbamoyl)piperidin-1-yl)nicotinate (**10**)*

^{1}H NMR (400 MHz, $CDCl_3$) and **^{13}C NMR** (100 MHz, $CDCl_3$)

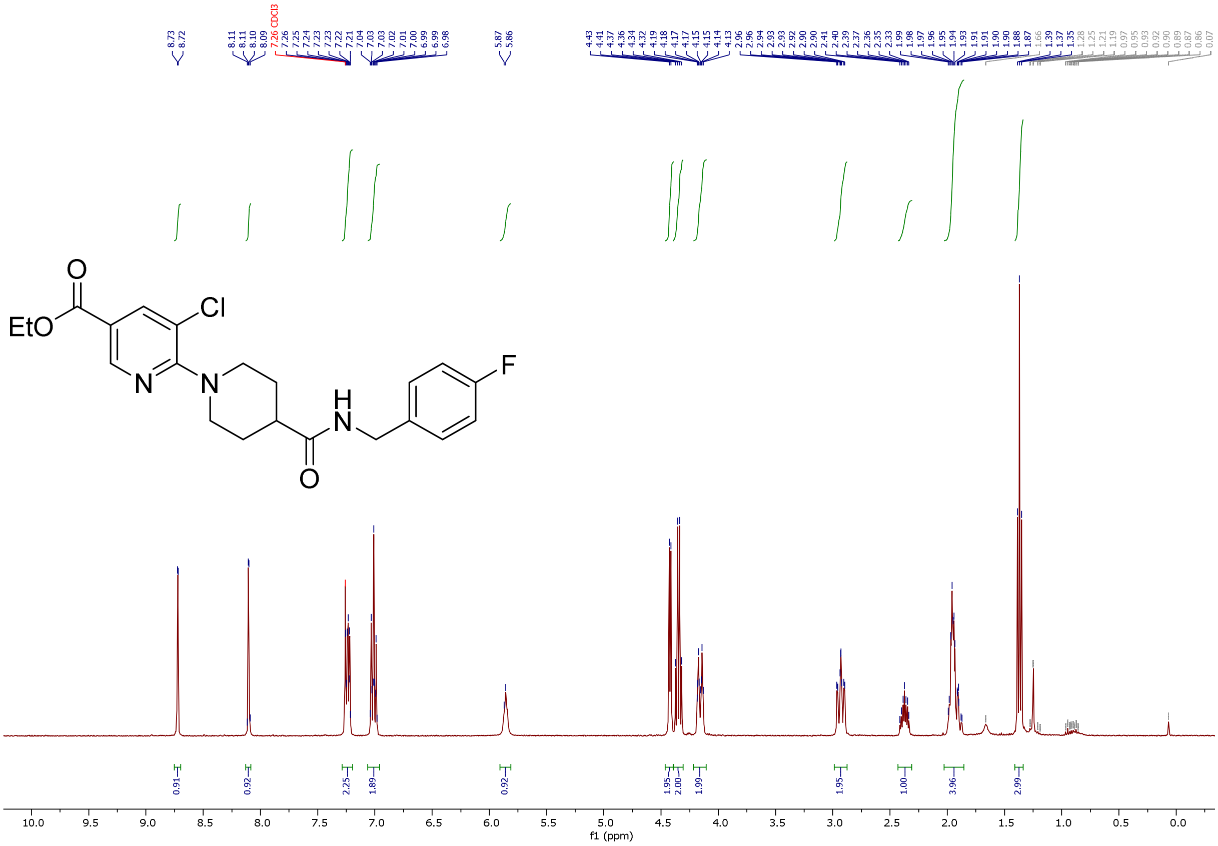

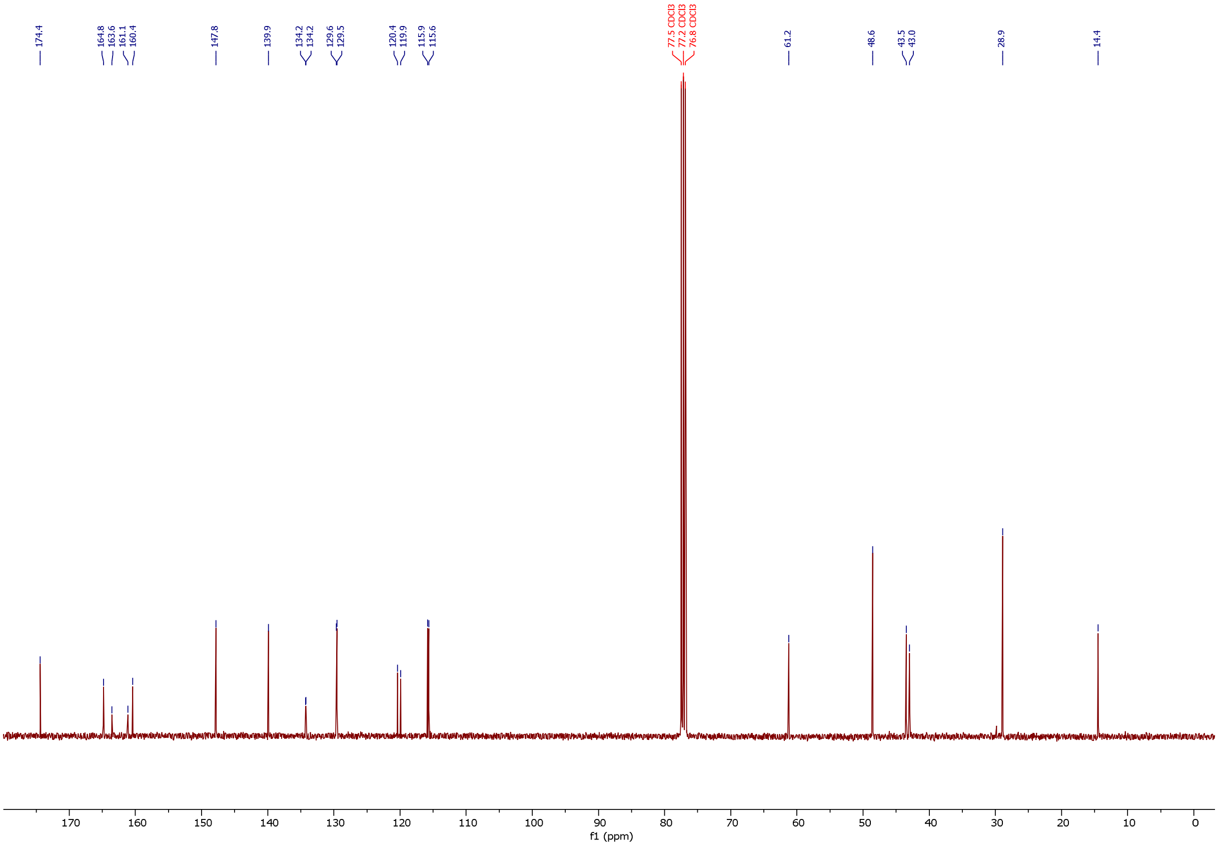

*Ethyl 5-chloro-6-(4-((4-fluorobenzyl)carbamoyl)piperidin-1-yl)-2-methylnicotinate (**11**)*

^{1}H NMR (400 MHz, $CDCl_3$) and **^{13}C NMR** (100 MHz, $CDCl_3$)

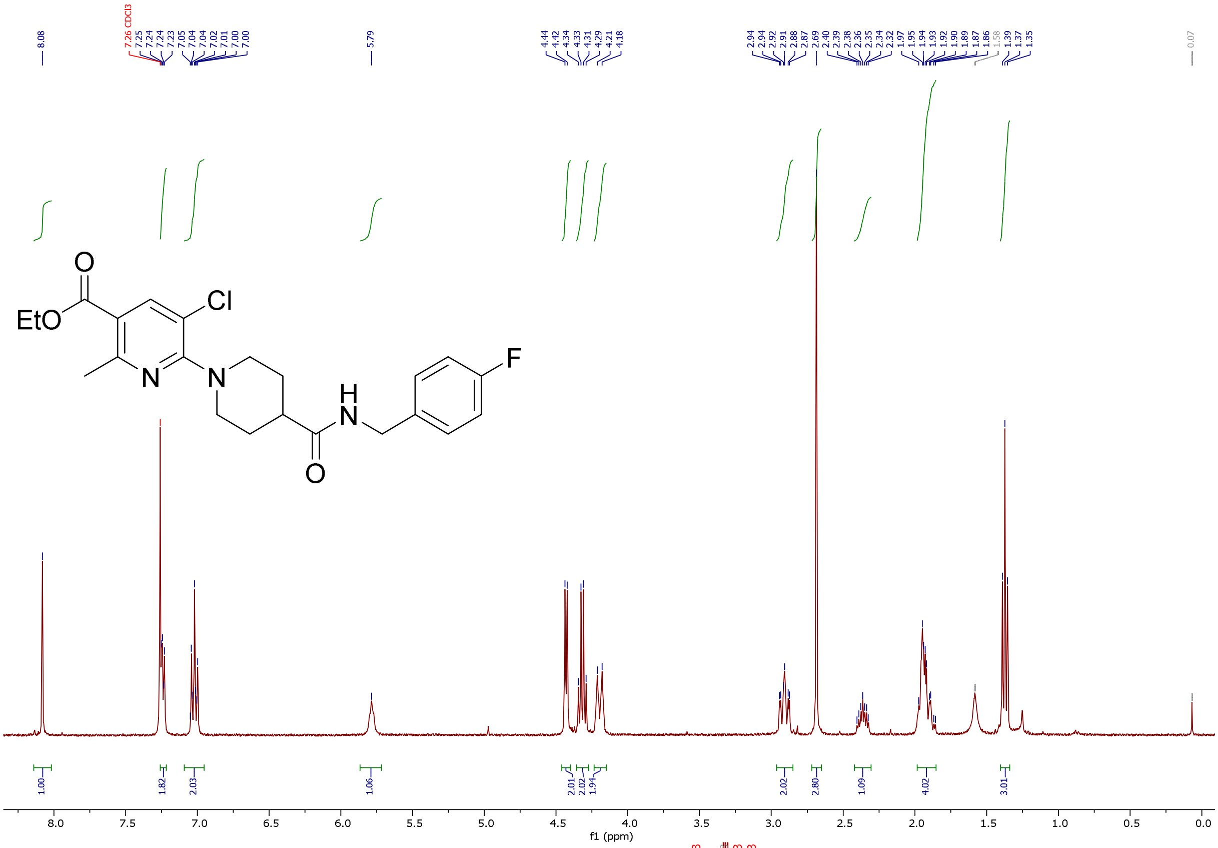

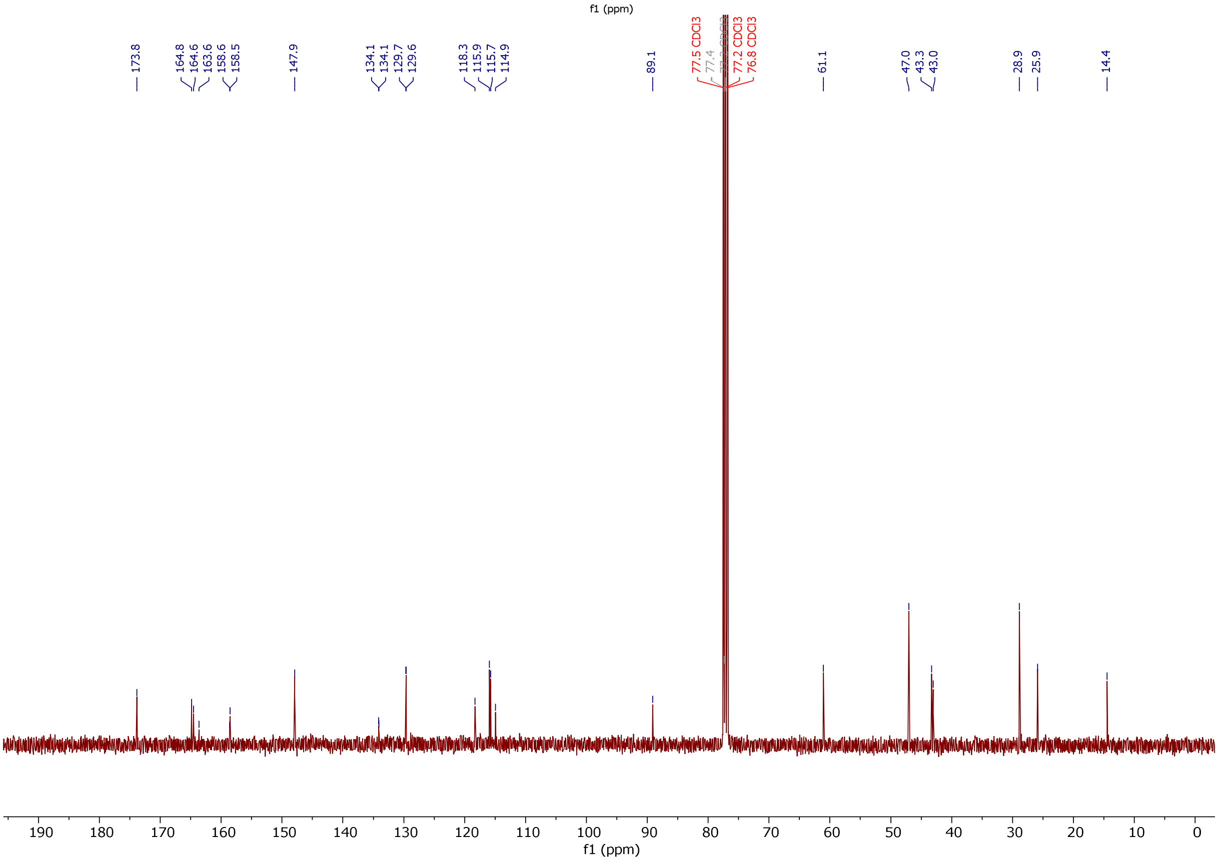

*Ethyl 5-cyano-6-(4-((4-fluorobenzyl)carbamoyl)piperidin-1-yl)-2-methylnicotinate (**12**)*

^{1}H NMR (400 MHz, $CDCl_3$) and **^{13}C NMR** (100 MHz, $CDCl_3$)

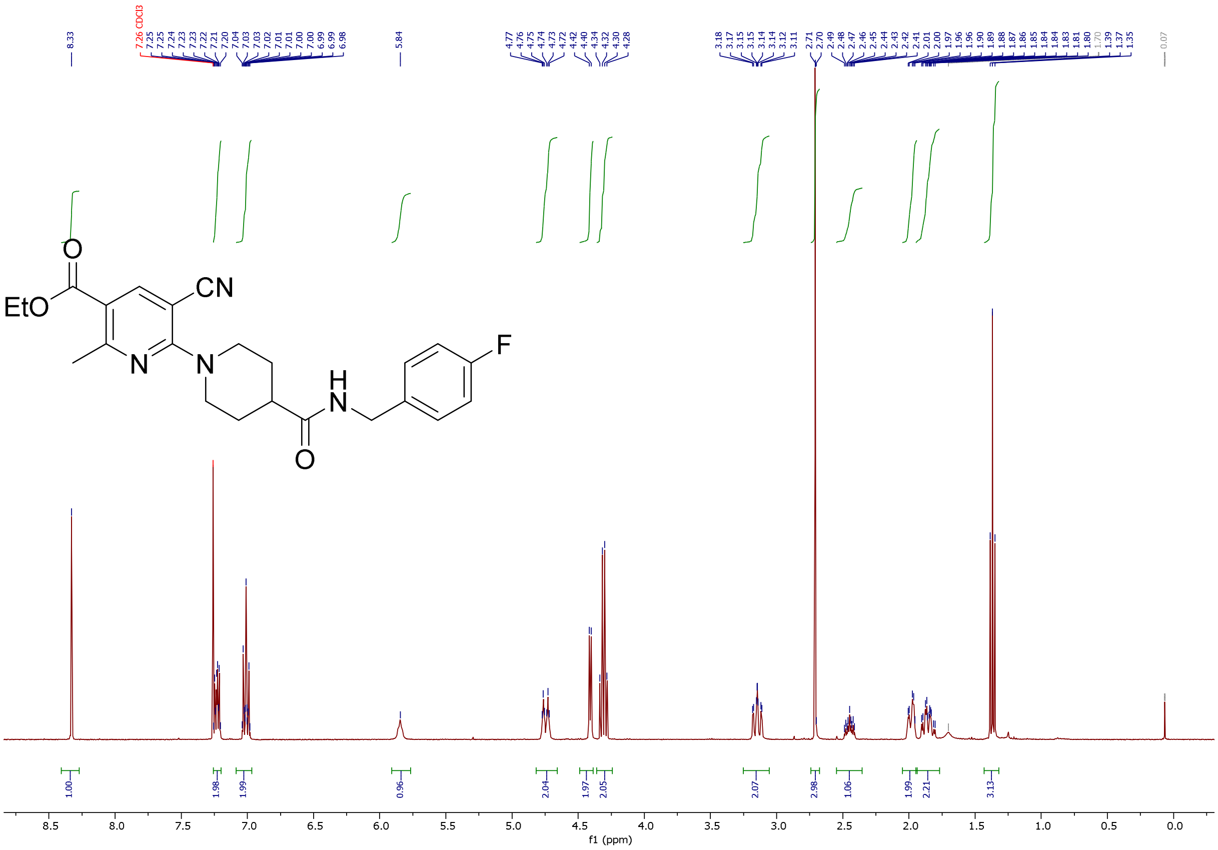

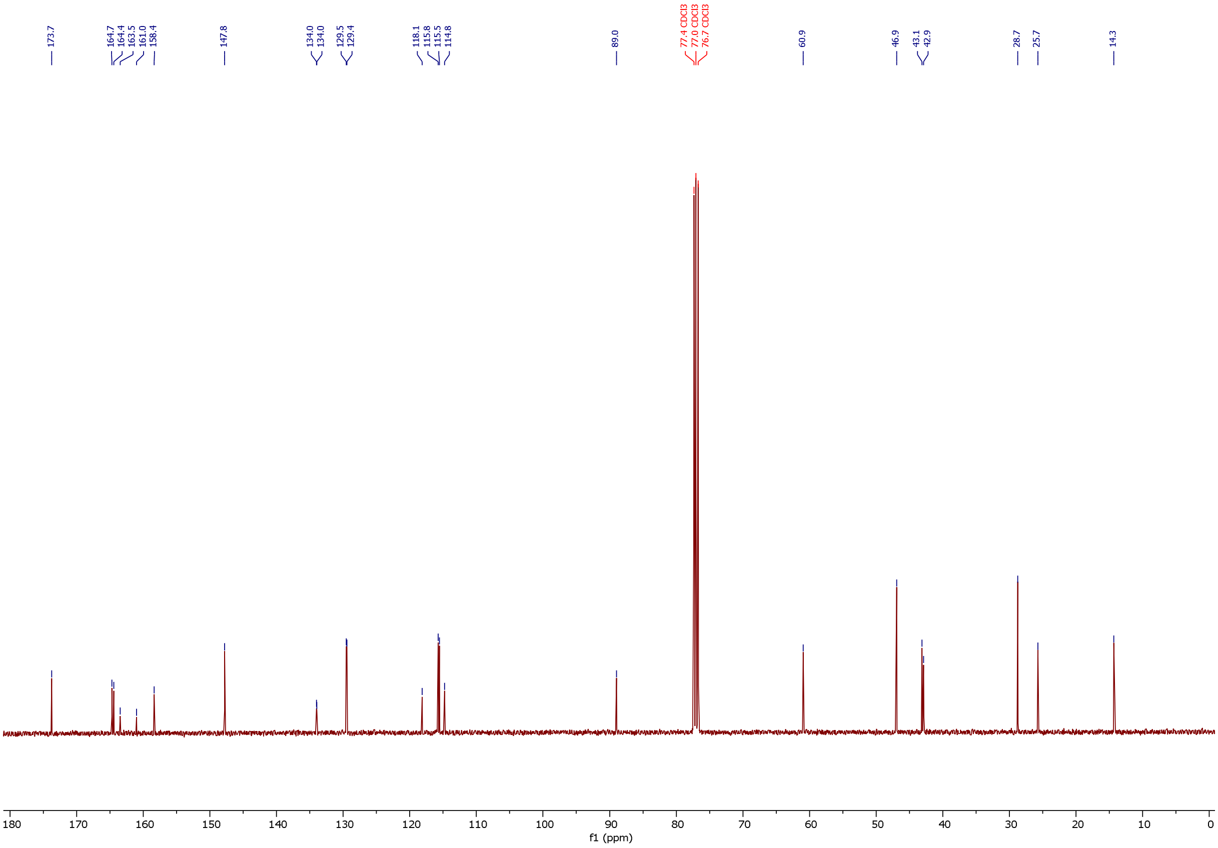

*Ethyl 5-chloro-4-(4-((4-fluorobenzyl)carbamoyl)piperidin-1-yl)-2-propoxybenzoate (**13**)*

^{1}H NMR (400 MHz, $CDCl_3$) and **^{13}C NMR** (100 MHz, $CDCl_3$)

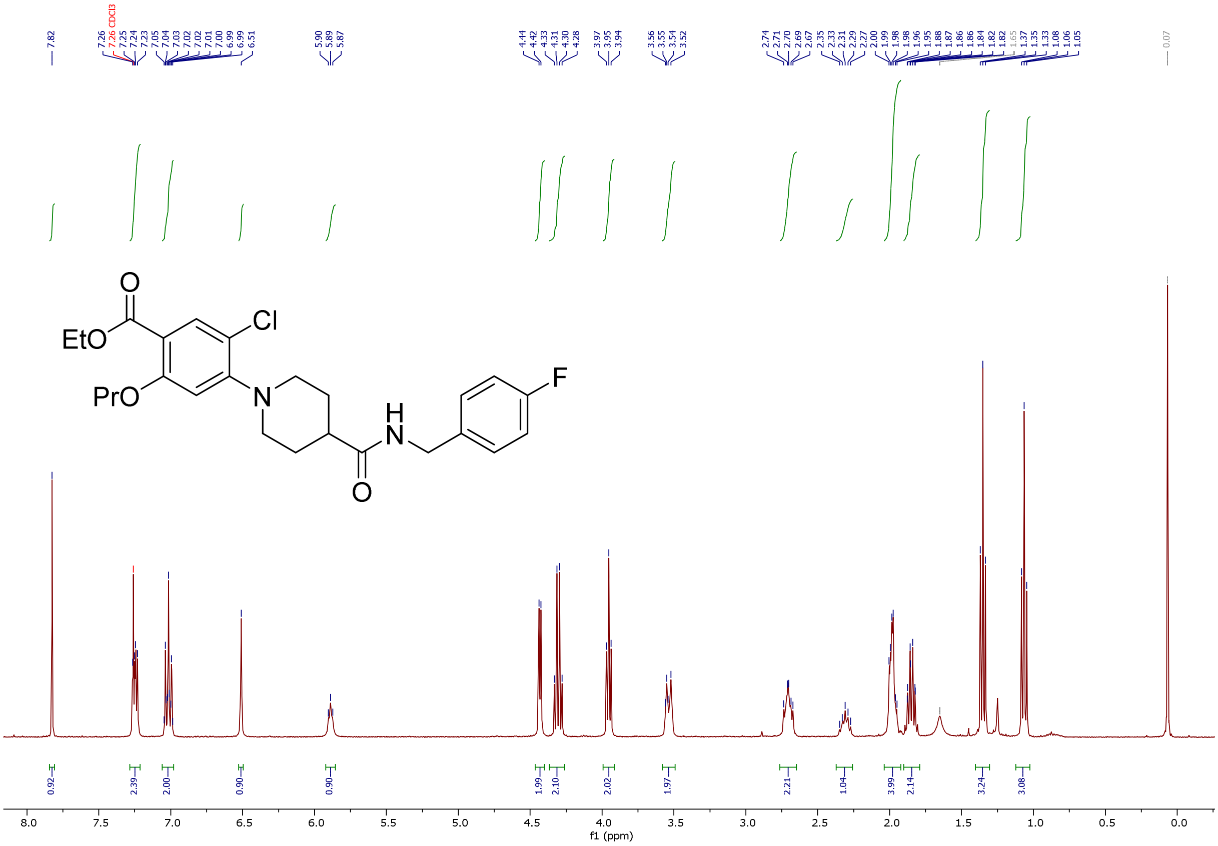

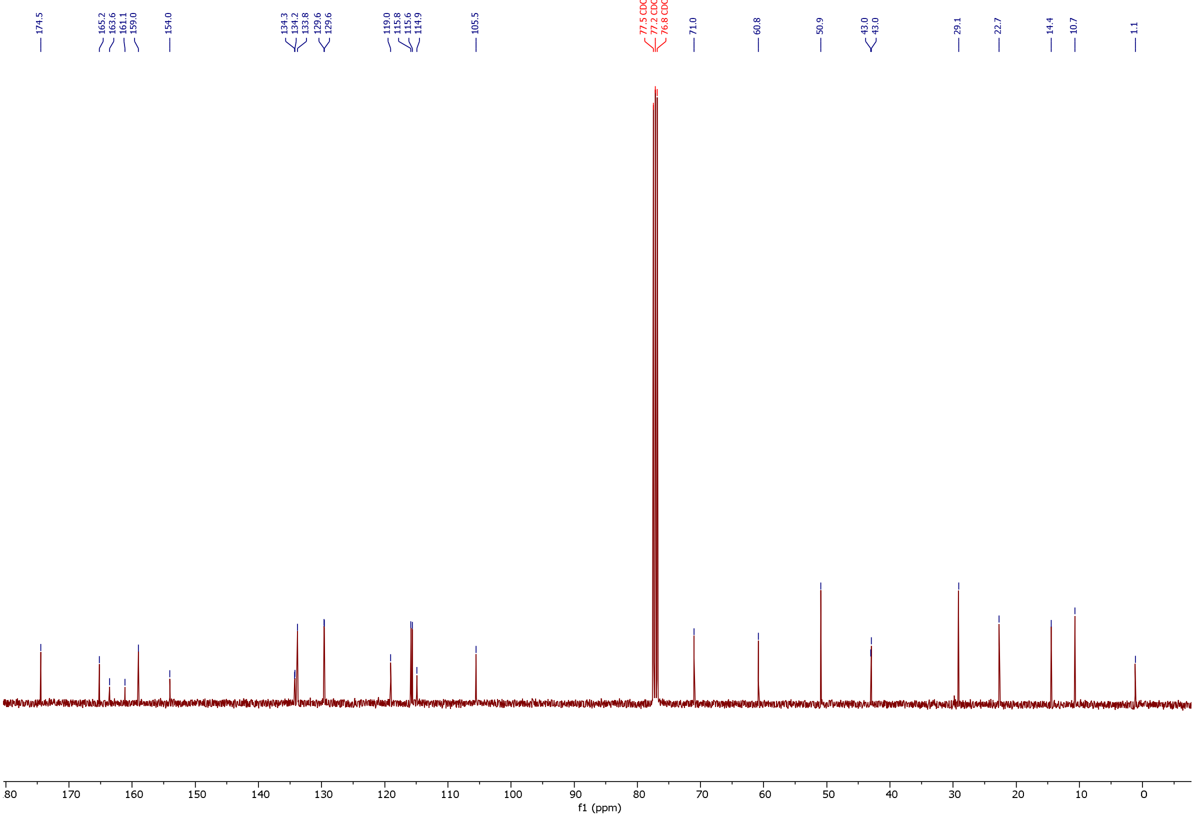

*1-(3-Cyano-5-oxo-5,7-dihydrofuro[3,4-b]pyridin-2-yl)-N-(4-fluorobenzyl)piperidine-4-carboxamide (**14**)*

^{1}H NMR (400 MHz, DMSO-d_6) and **^{13}C NMR** (100 MHz, DMSO-d_6)

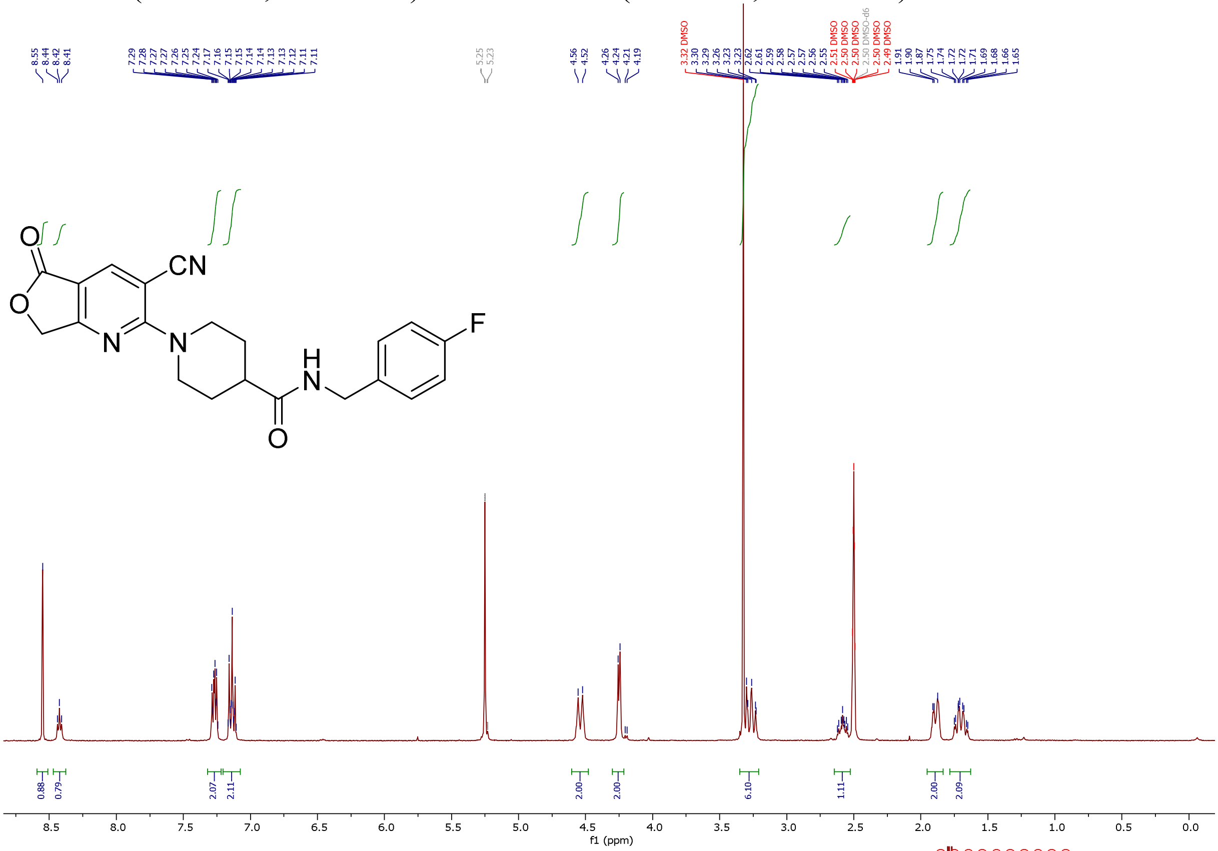

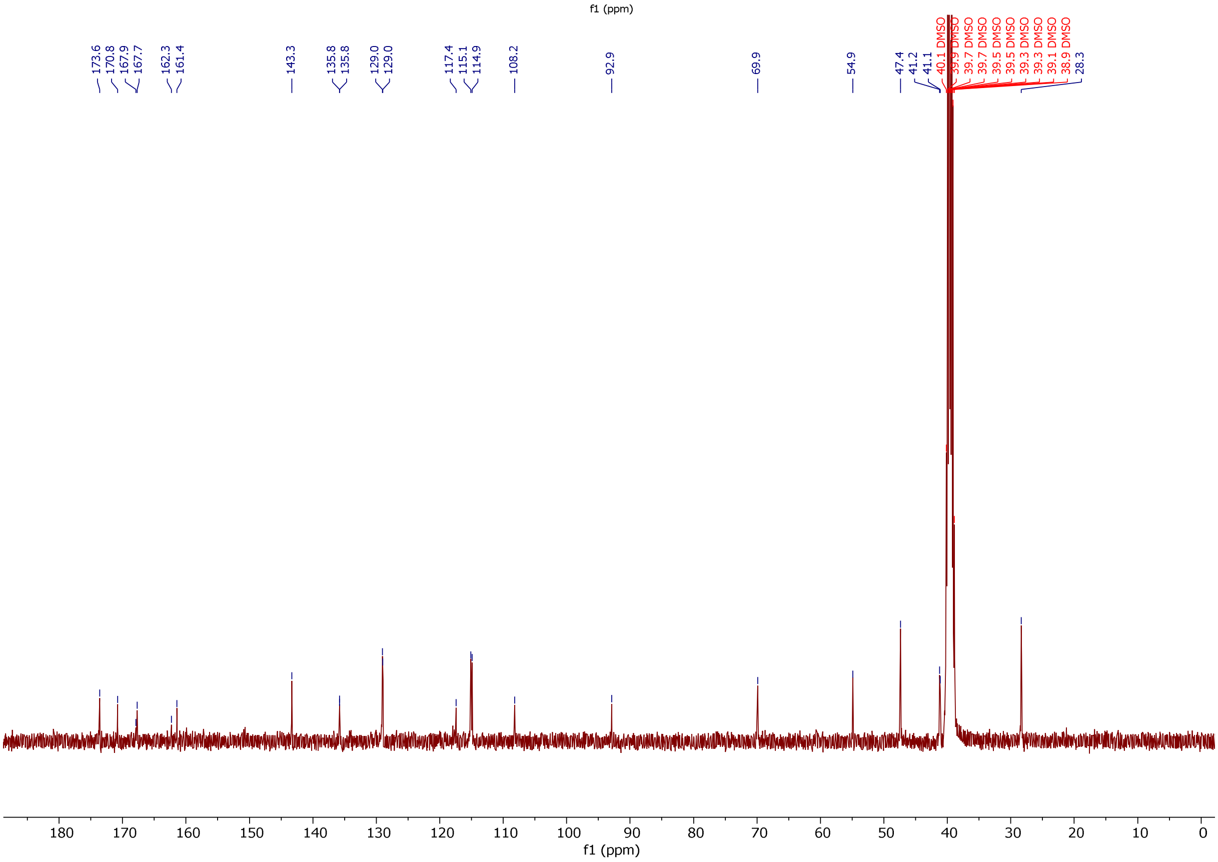

*Ethyl 6-(4-((4-fluorophenethyl)carbamoyl)piperidin-1-yl)-2-methylnicotinate (**15**)*

^{1}H NMR (400 MHz, $CDCl_3$) and **^{13}C NMR** (100 MHz, $CDCl_3$)

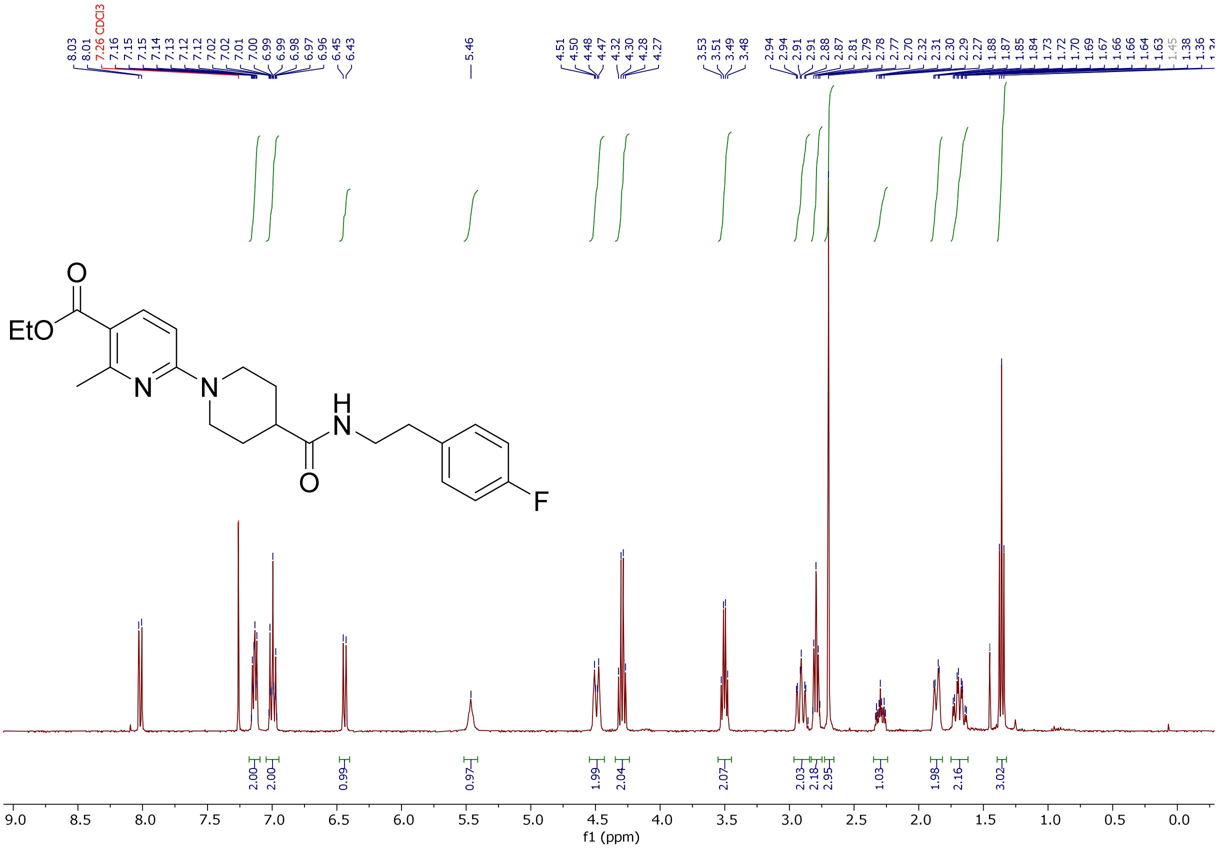

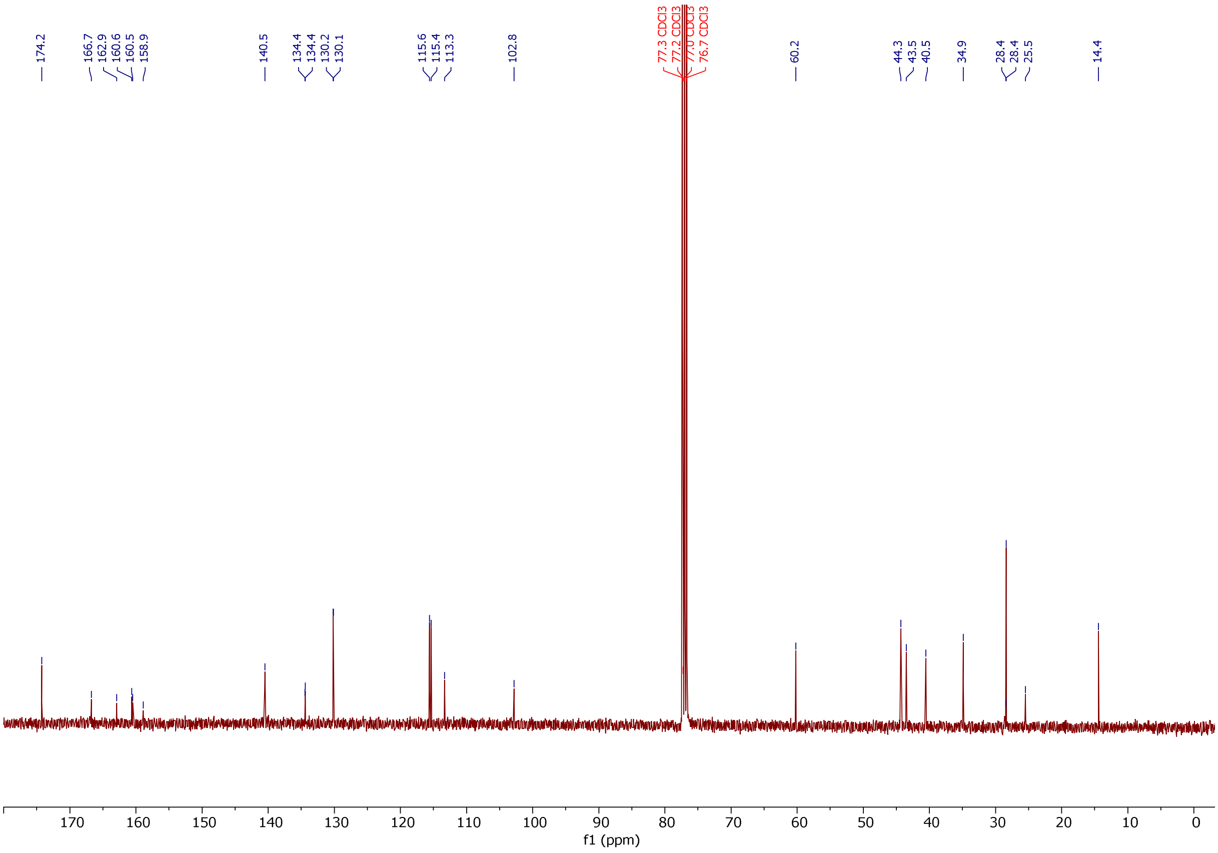

*Ethyl 5-chloro-6-(4-((4-fluorophenethyl)carbamoyl)piperidin-1-yl)nicotinate (**16**)*

^{1}H NMR (400 MHz, $CDCl_3$) and **^{13}C NMR** (100 MHz, $CDCl_3$)

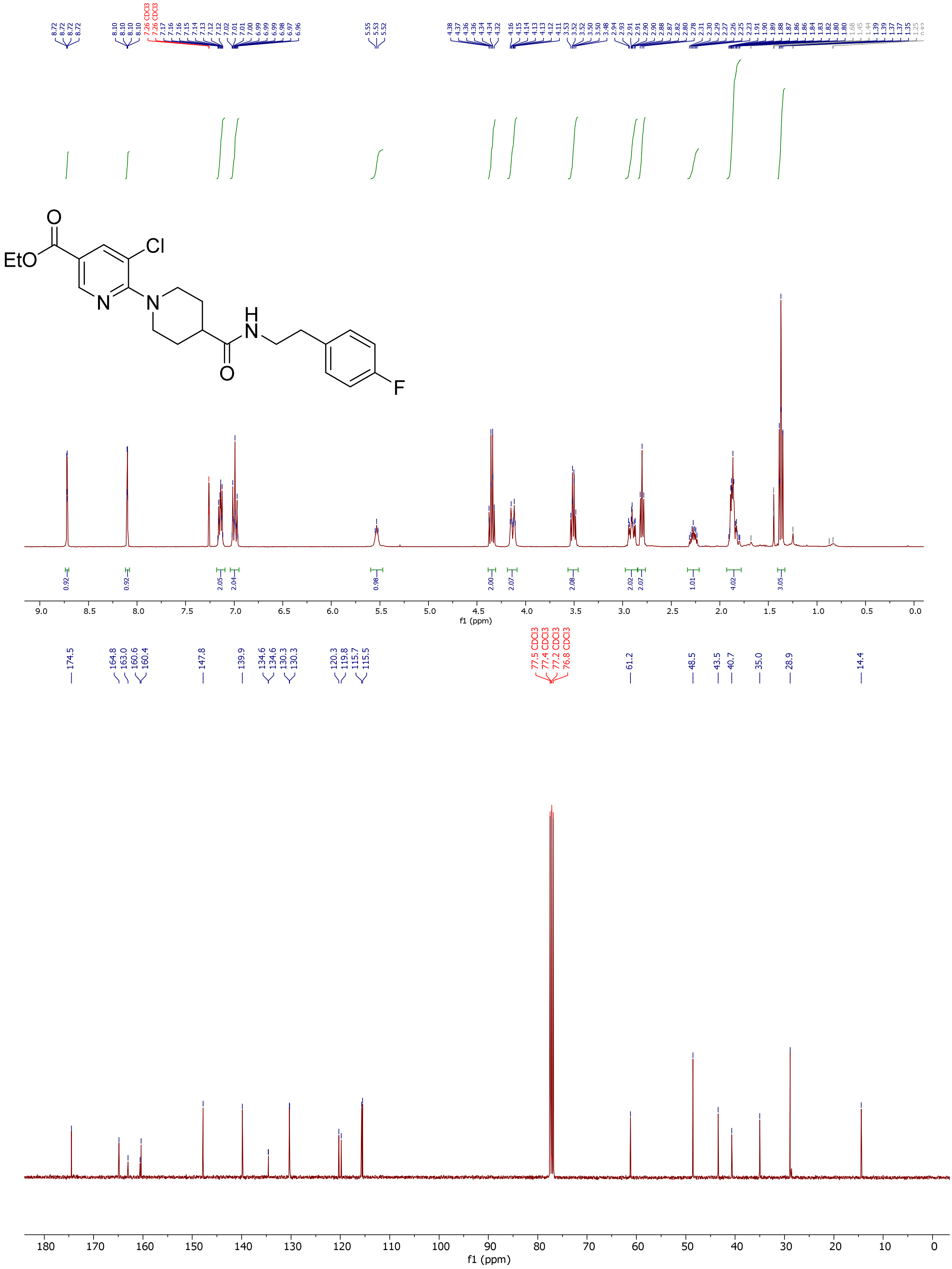

*Ethyl 5-chloro-6-(4-((4-fluorophenethyl)carbamoyl)piperidin-1-yl)-2-methylnicotinate (**17**)*

^{1}H NMR (400 MHz, $CDCl_3$) and **^{13}C NMR** (100 MHz, $CDCl_3$)

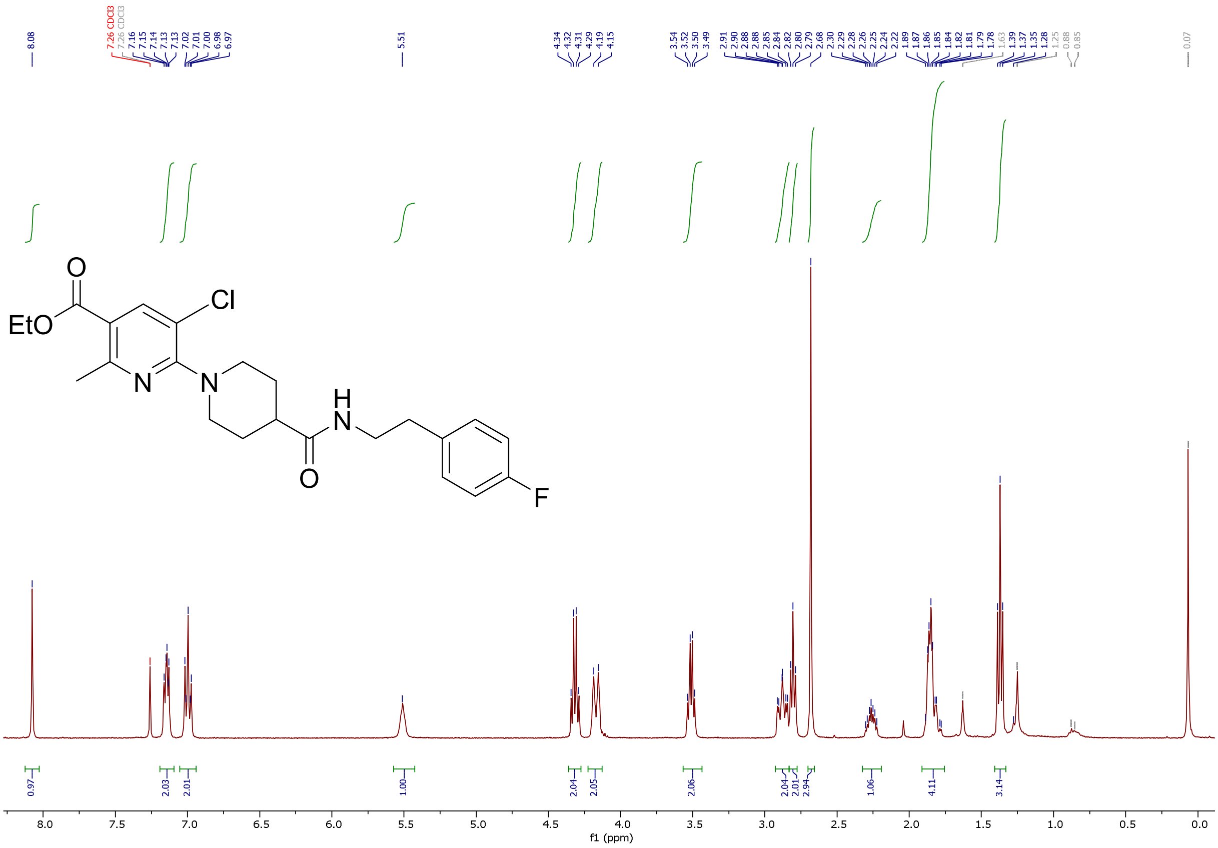

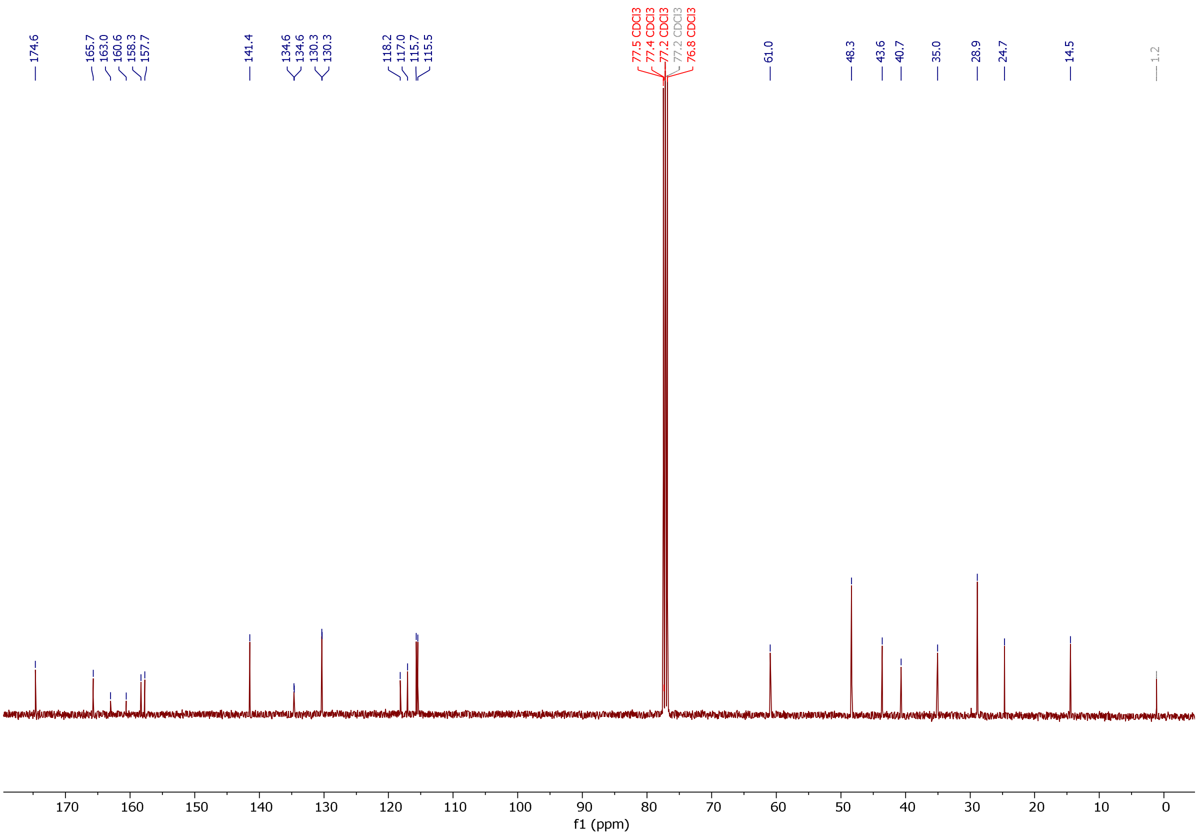

*Ethyl 5-cyano-6-(4-((4-fluorophenethyl)carbamoyl)piperidin-1-yl)-2-methylnicotinate (**18**)*

^{1}H NMR (400 MHz, $CDCl_3$) and **^{13}C NMR** (100 MHz, $CDCl_3$)

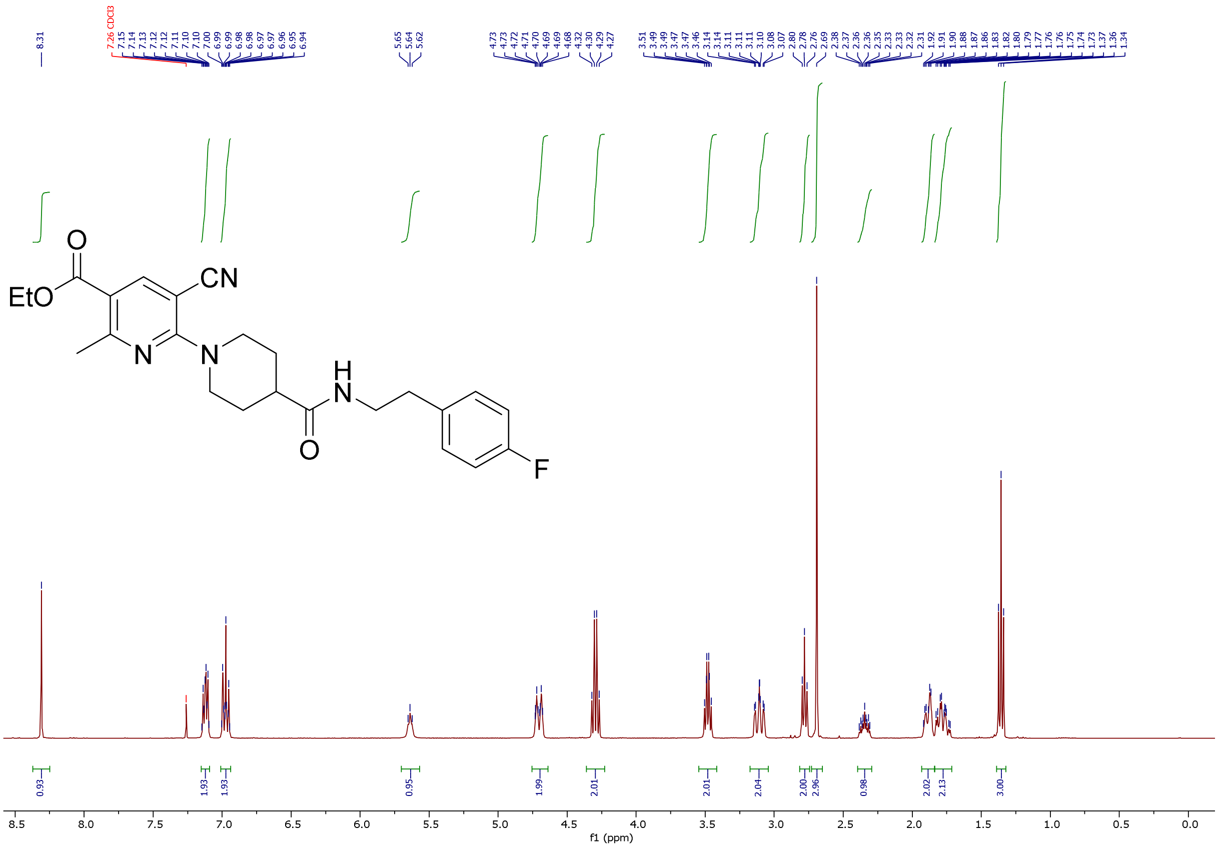

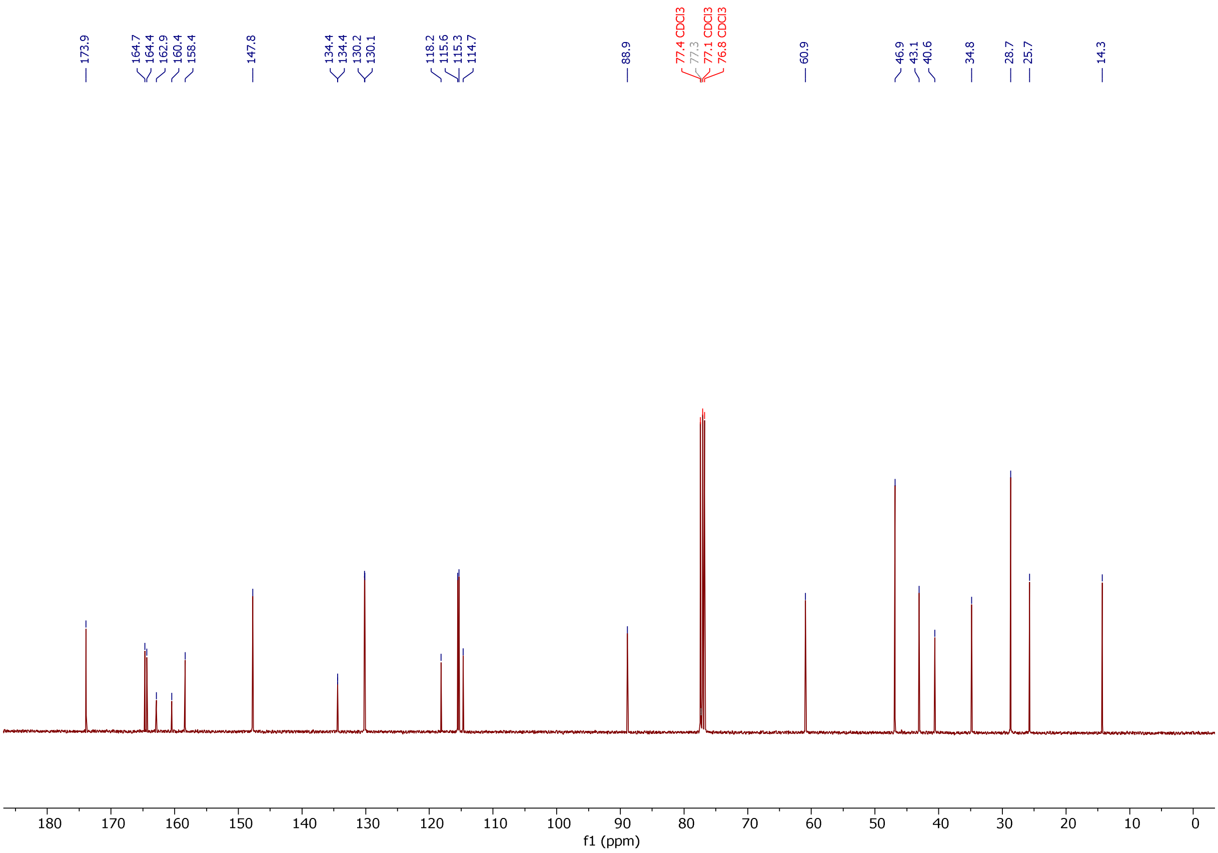

*Ethyl 5-chloro-4-(4-((4-fluorophenethyl)carbamoyl)piperidin-1-yl)-2-propoxybenzoate (**19**)*

^{1}H NMR (400 MHz, $CDCl_3$) and **^{13}C NMR** (100 MHz, $CDCl_3$)

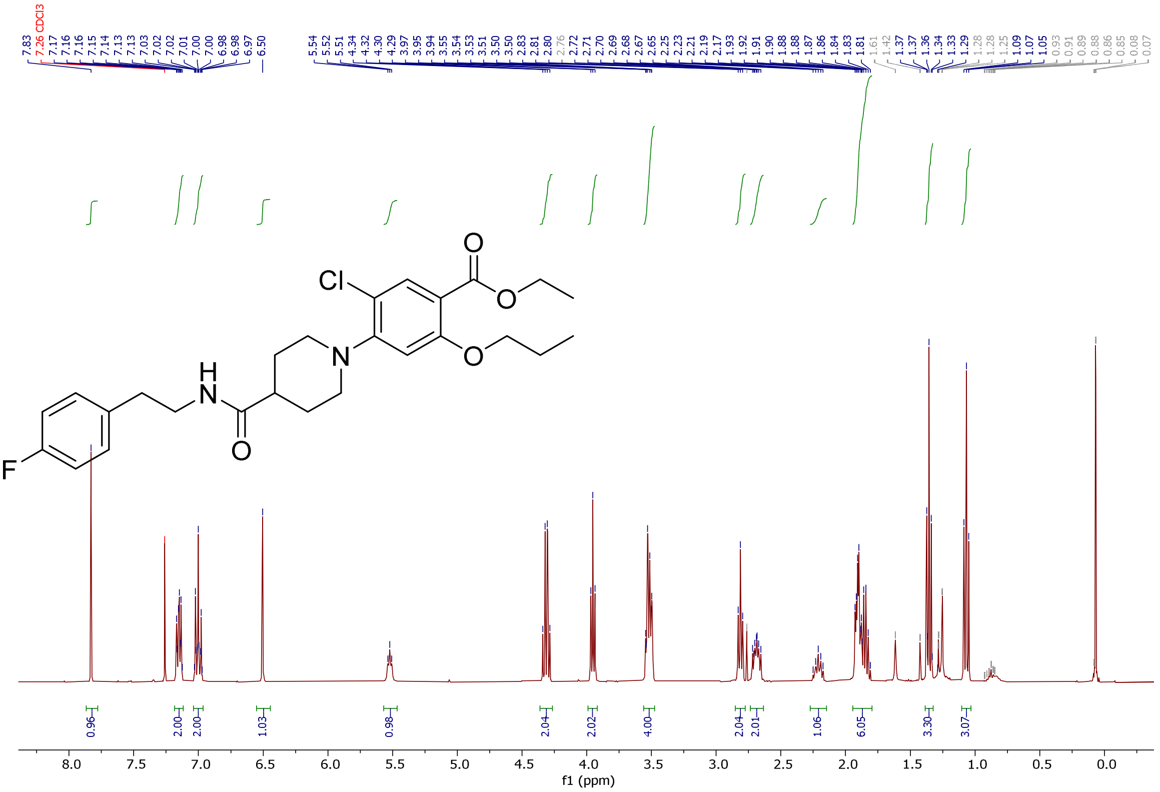

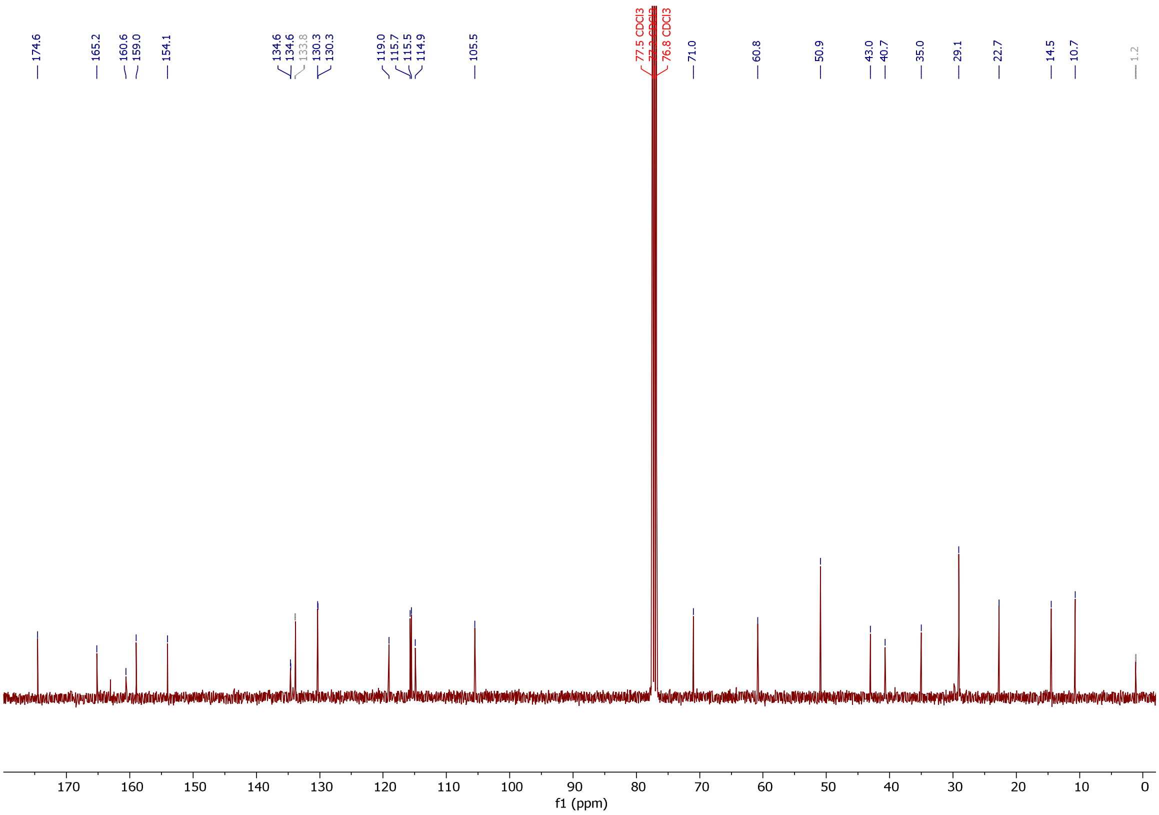

tert-Butyl 4-((4-(4,4,5,5-tetramethyl-1,3,2-dioxaborolan-2-yl)benzyl)carbamoyl)piperidine-1-carboxylate (20)

^{1}H NMR (400 MHz, $CDCl_3$) and **^{13}C NMR** (100 MHz, $CDCl_3$)

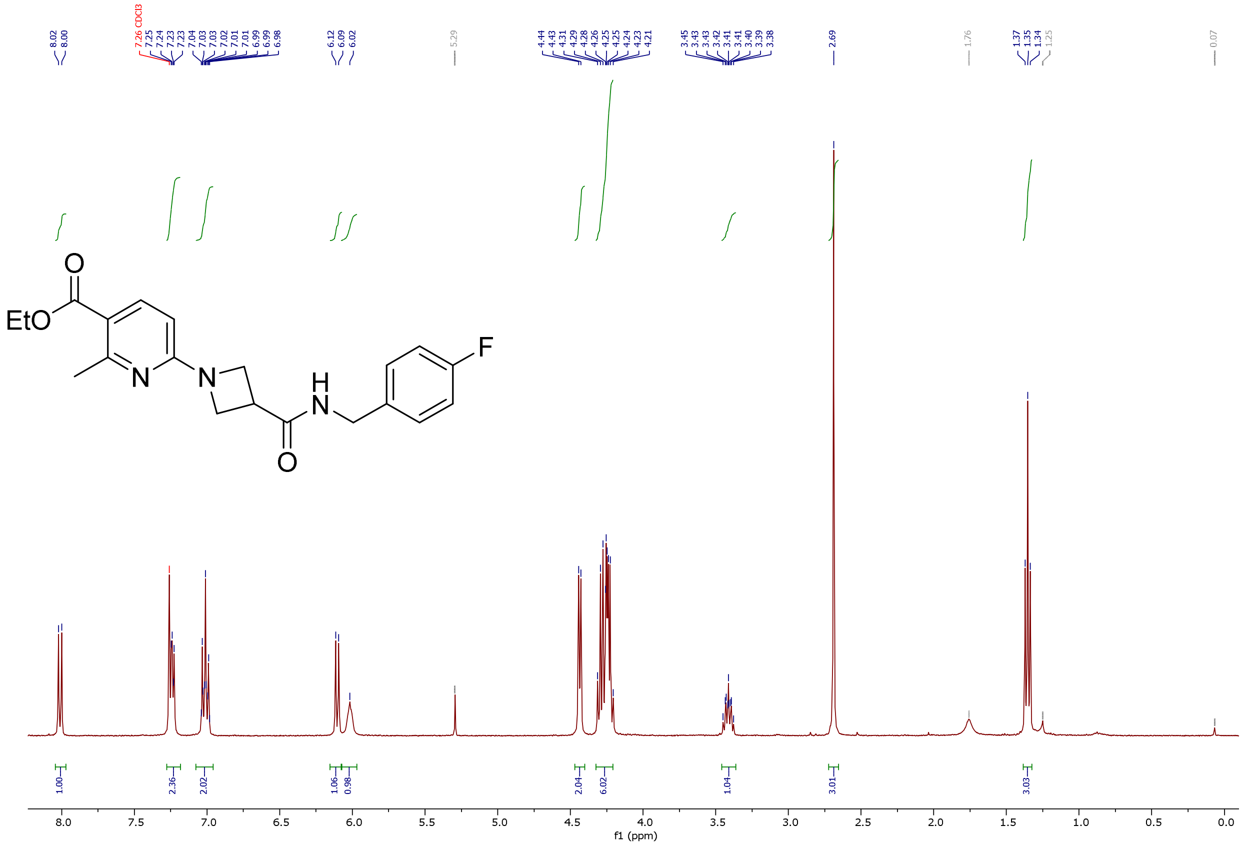

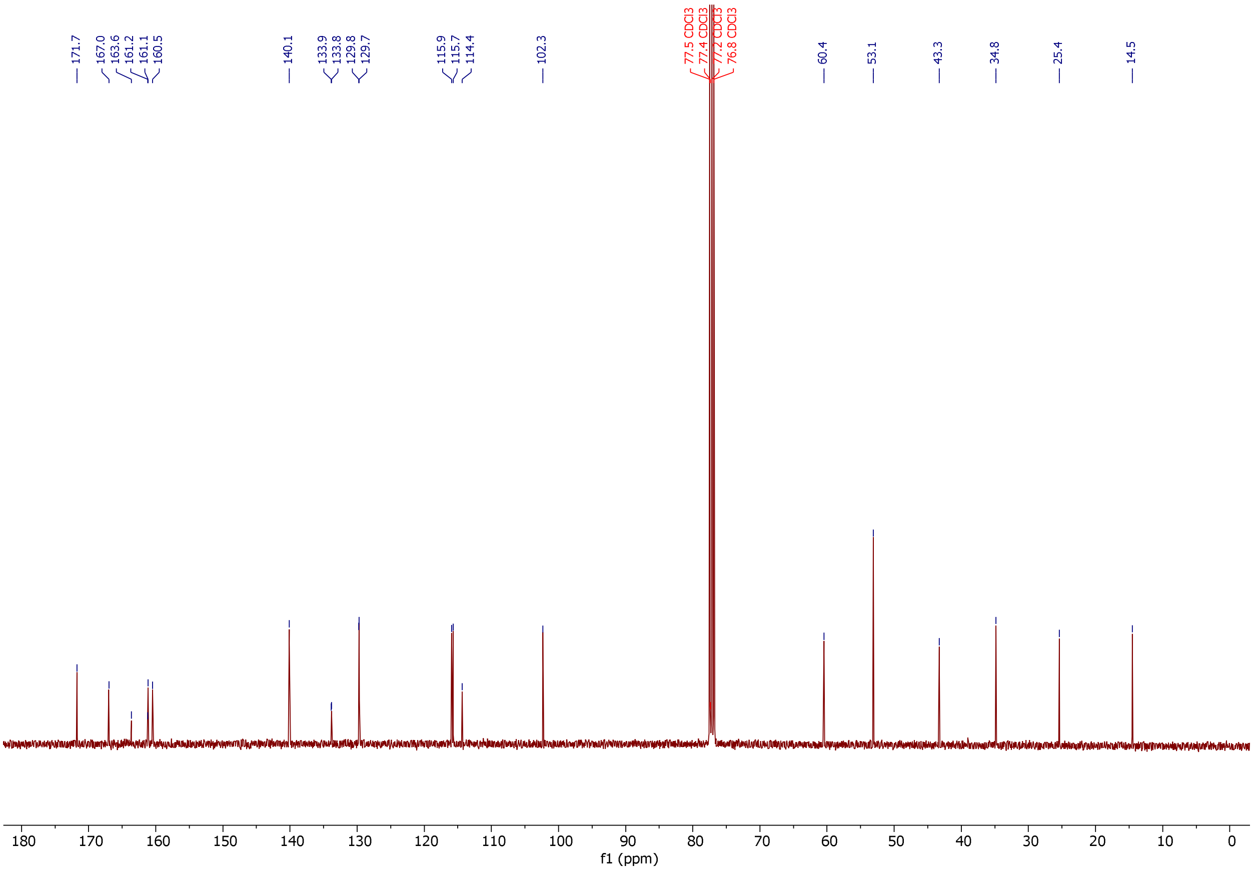

Ethyl 5-chloro-6(3-((4-fluorobenzyl)carbamoyl)azetidine-1-yl)nicotinate (21)

^{1}H NMR (400 MHz, DMSO-d_6) and **^{13}C NMR** (100 MHz, DMSO-d_6)

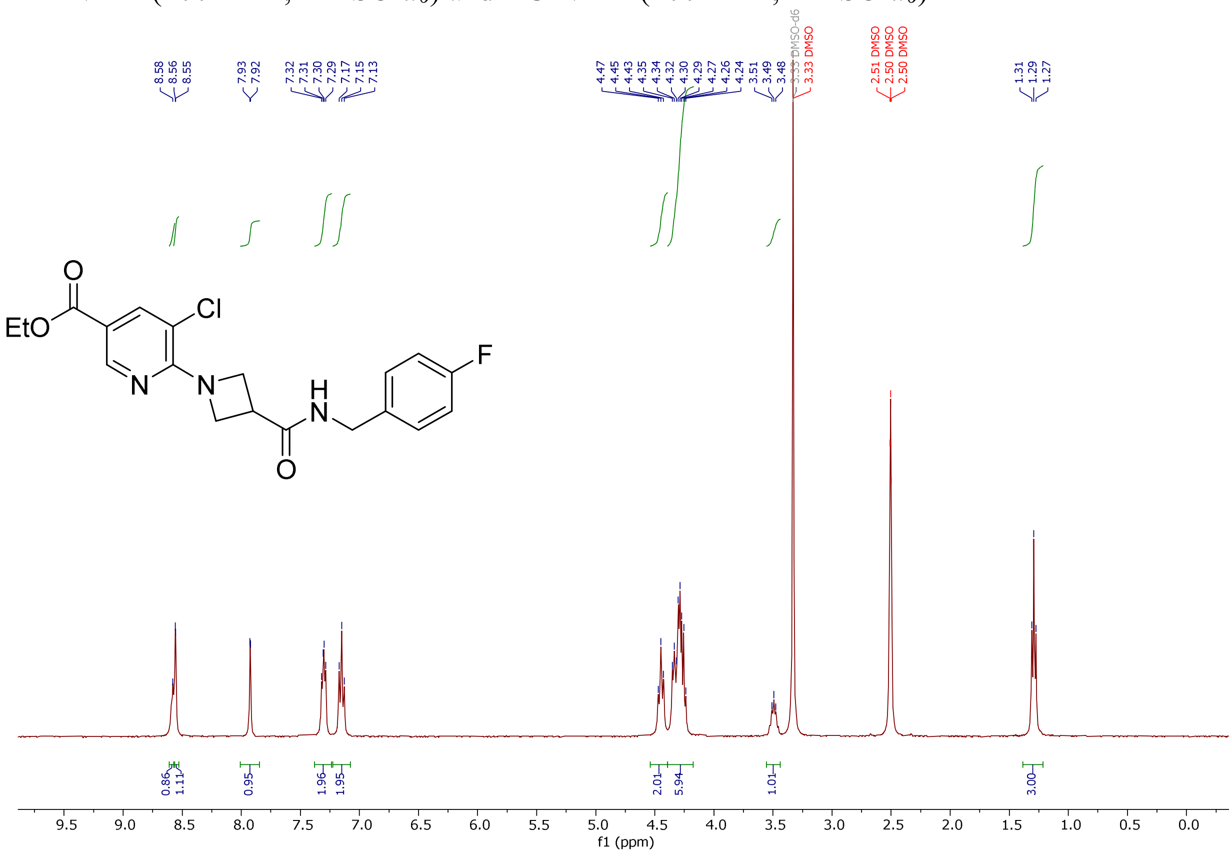

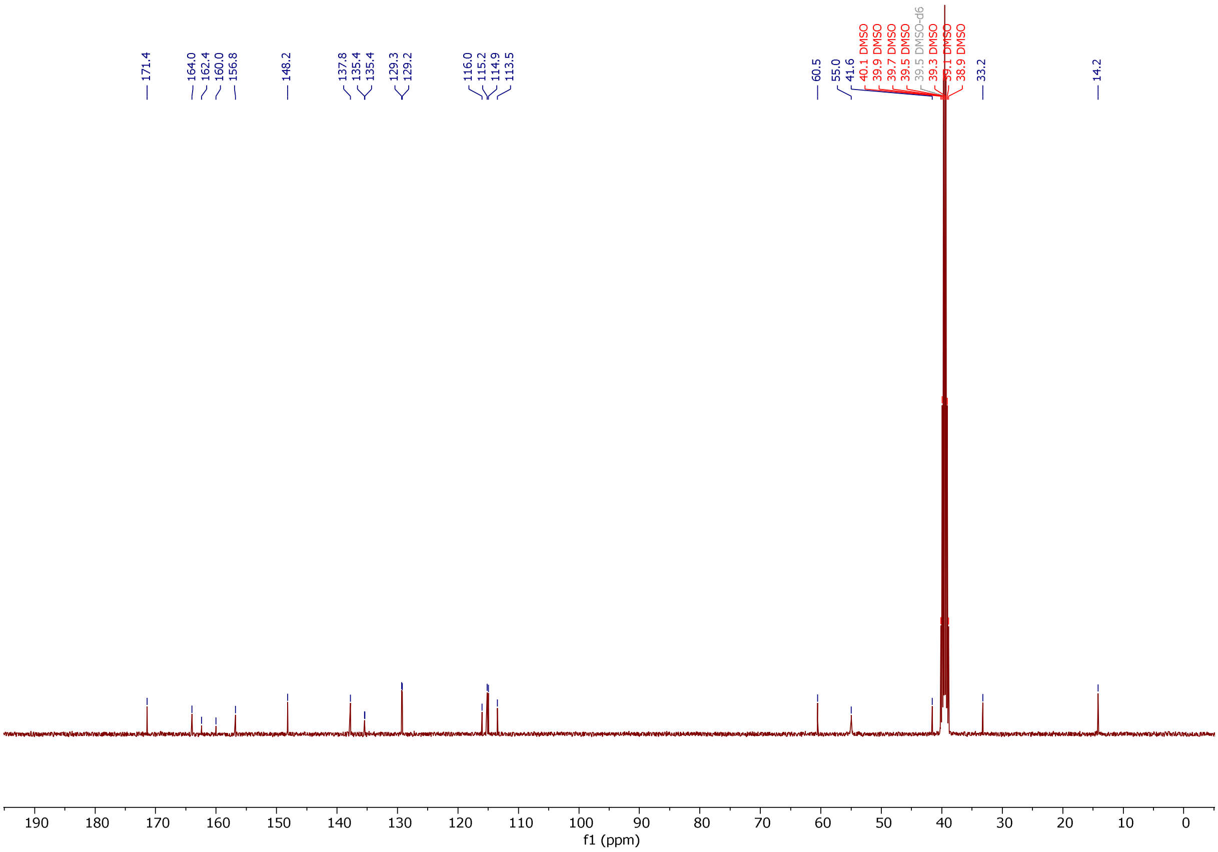

Ethyl 5-cyano-6-(3-((4-fluorobenzyl)carbamoyl)azetidin-1-yl)-2-methylnicotinate (***22***)

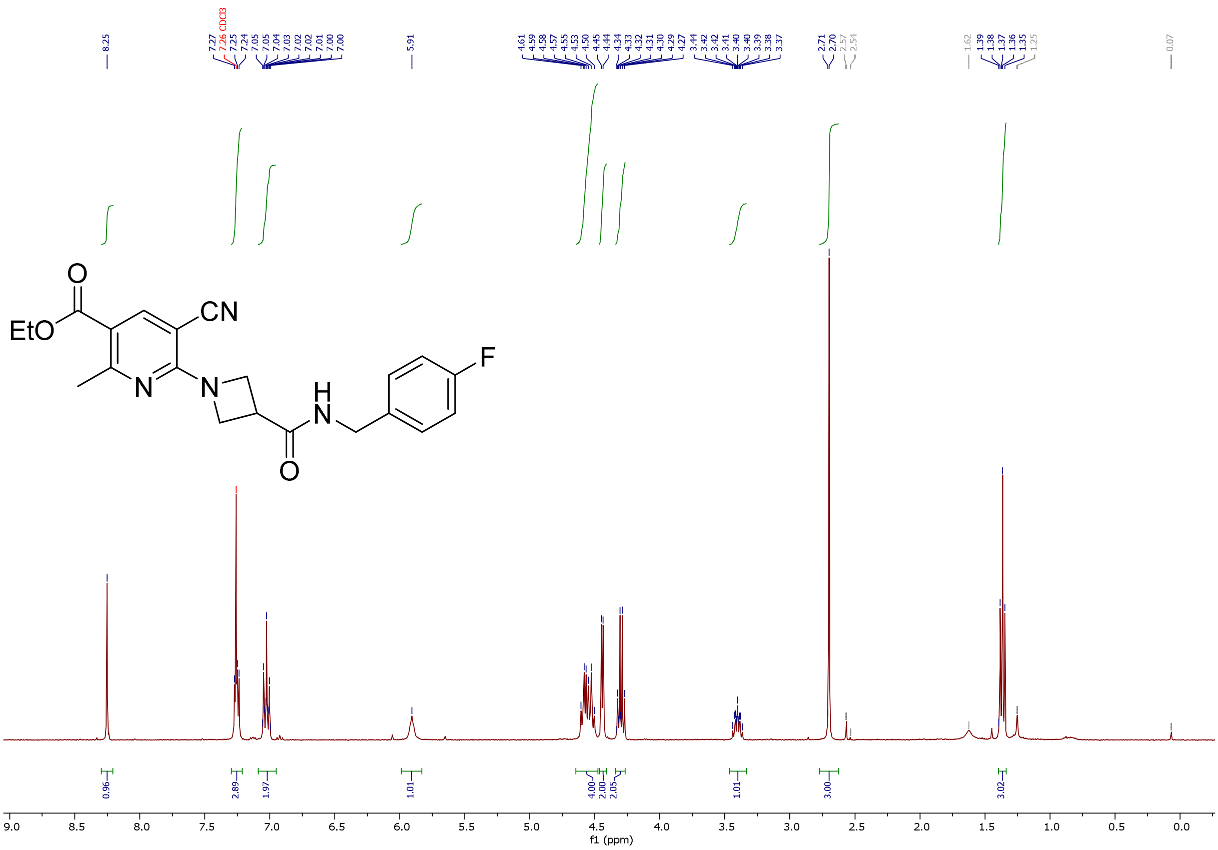

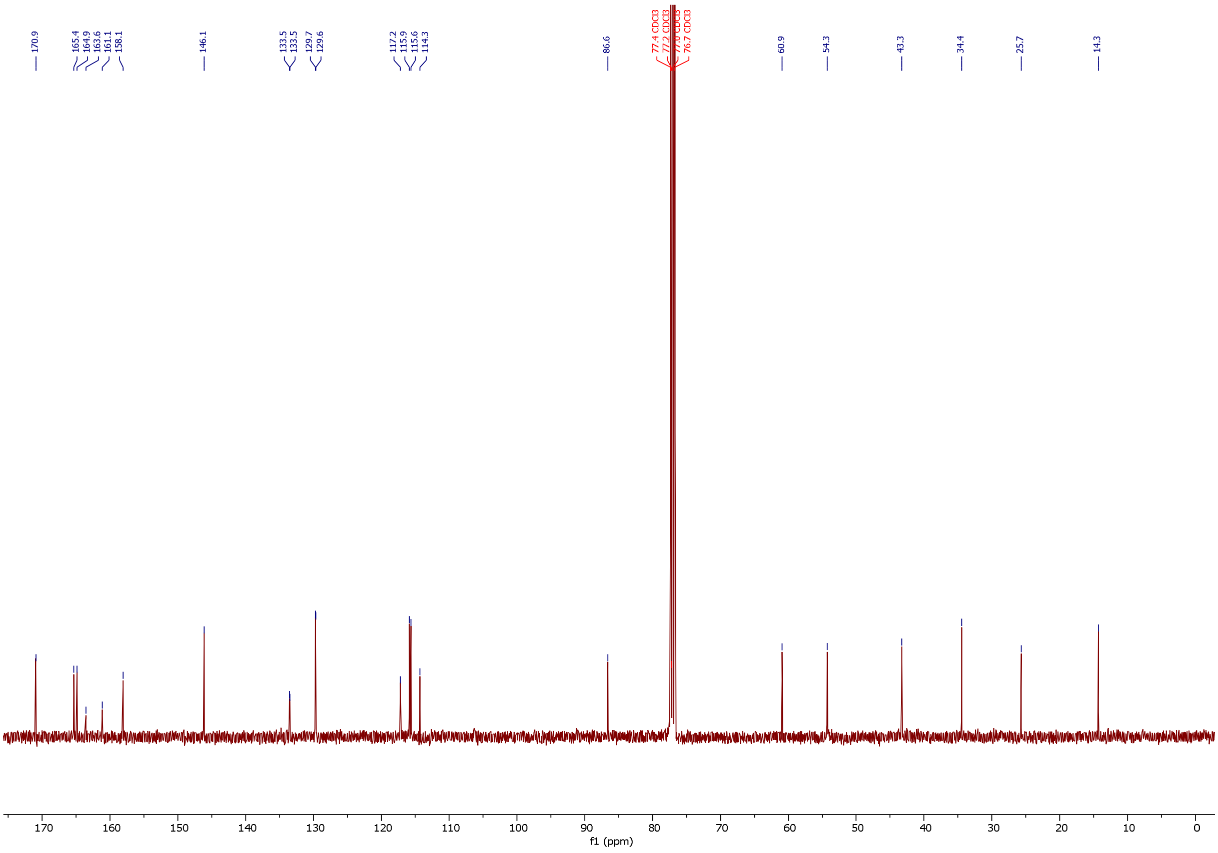

*tert-Butyl piperazine-1-carboxylate (**23**)*

^{1}H NMR (400 MHz, $CDCl_3$) and **^{13}C NMR** (100 MHz, $CDCl_3$)

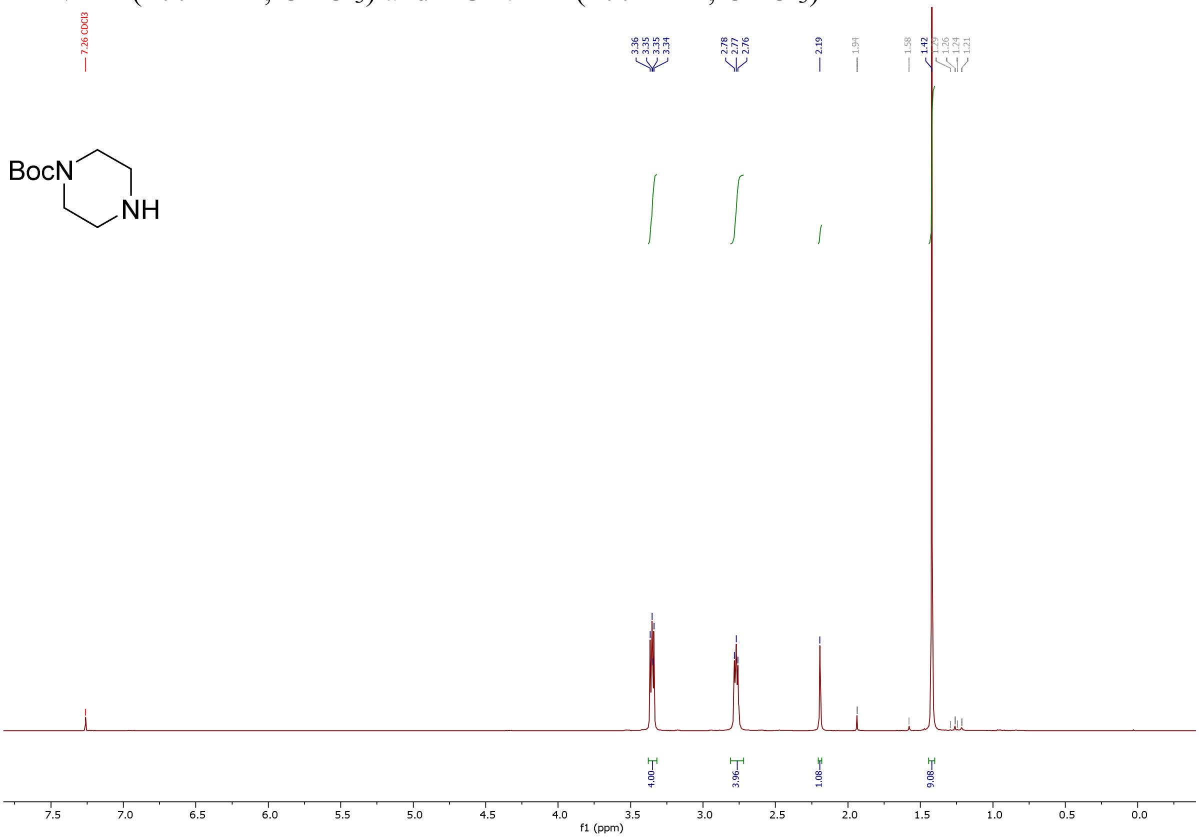

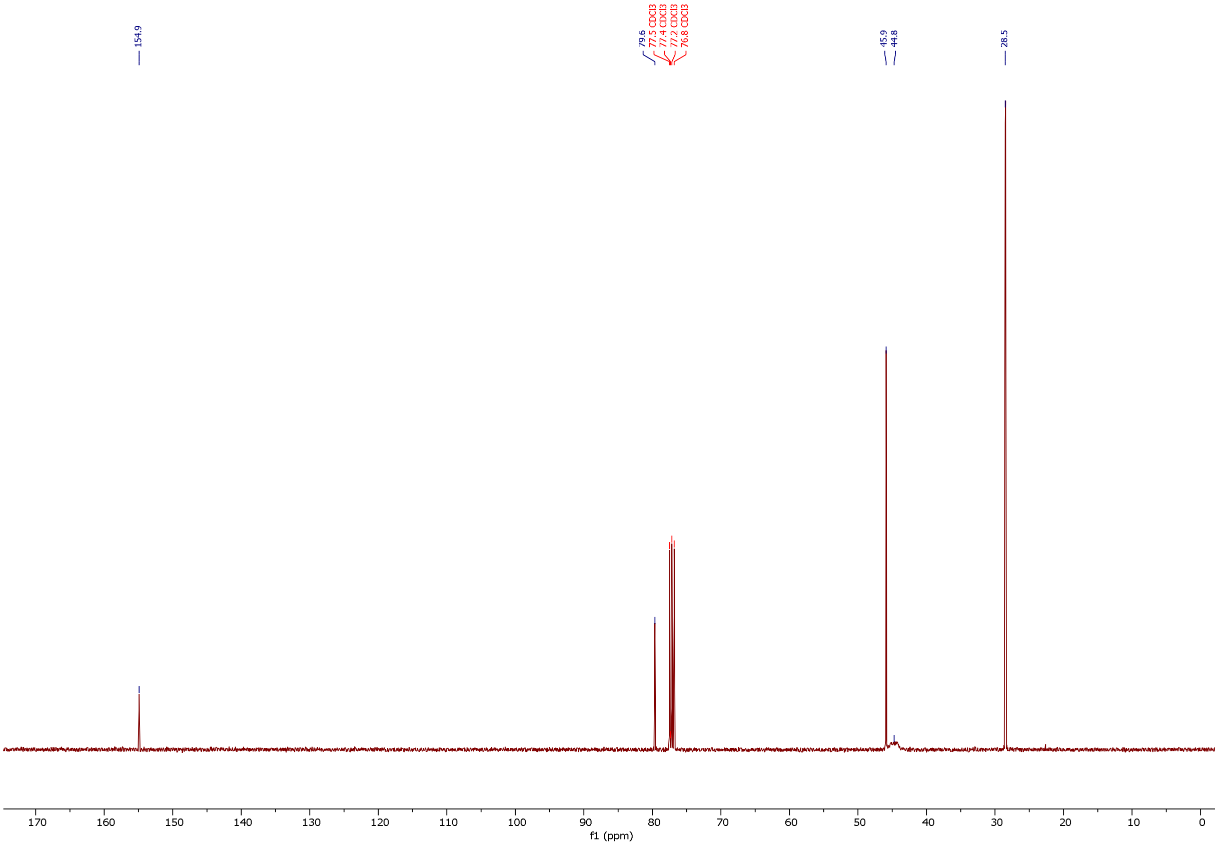

*tert-Butyl 4-((4-fluorobenzyl)carbamoyl)piperazine-1-carboxylate (**24**)*

^{1}H NMR (400 MHz, $CDCl_3$) and **^{13}C NMR** (100 MHz, $CDCl_3$)

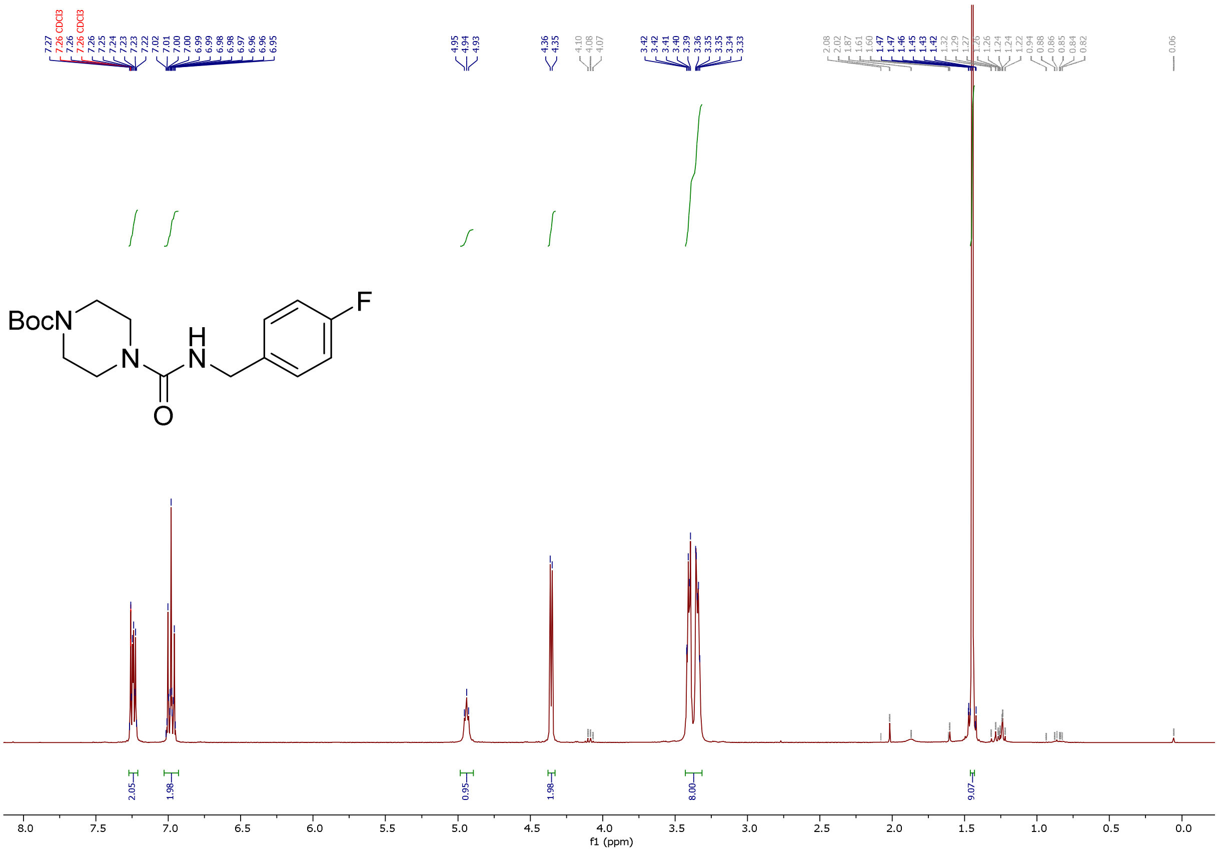

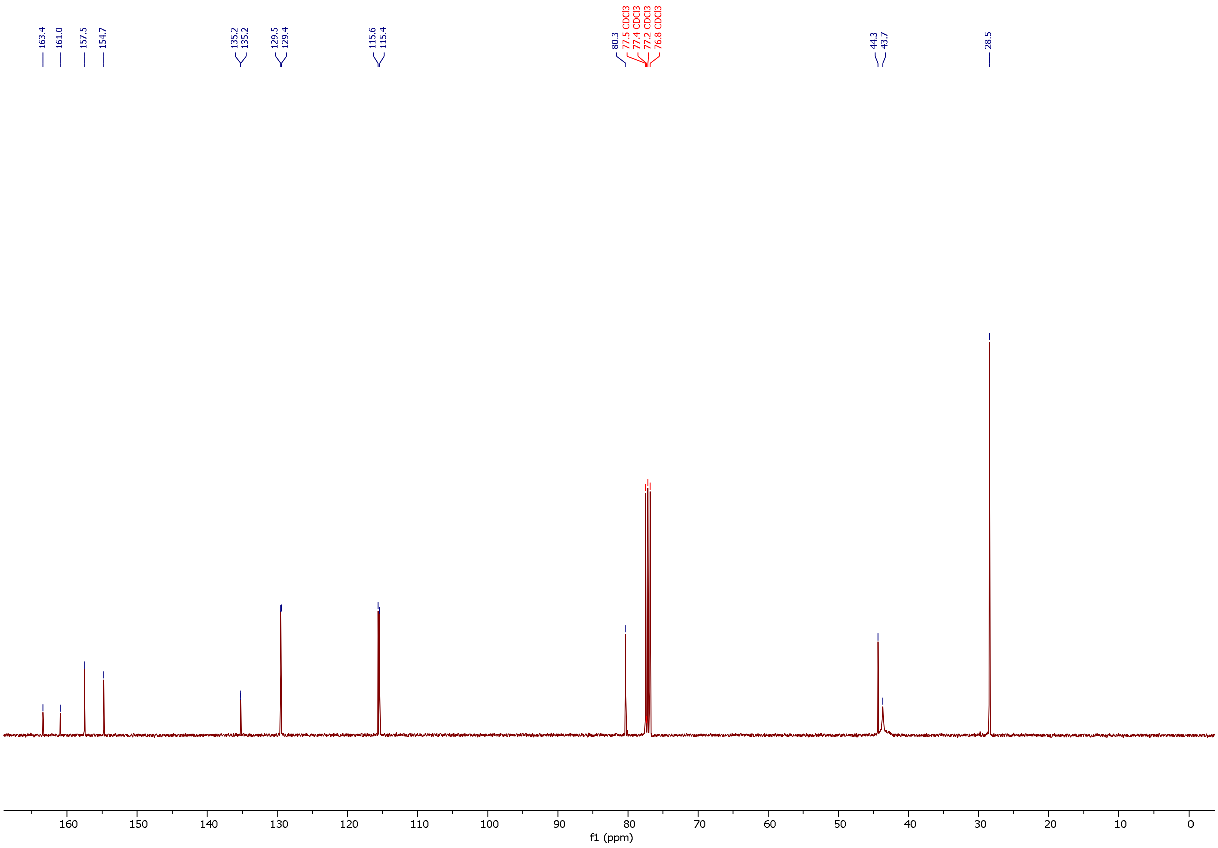

*N-(4-Fluorobenzyl)piperazine-1-carboxamide (**25**)*

^{1}H NMR (400 MHz, D_2O) and **^{13}C NMR** (100 MHz, D_2O)

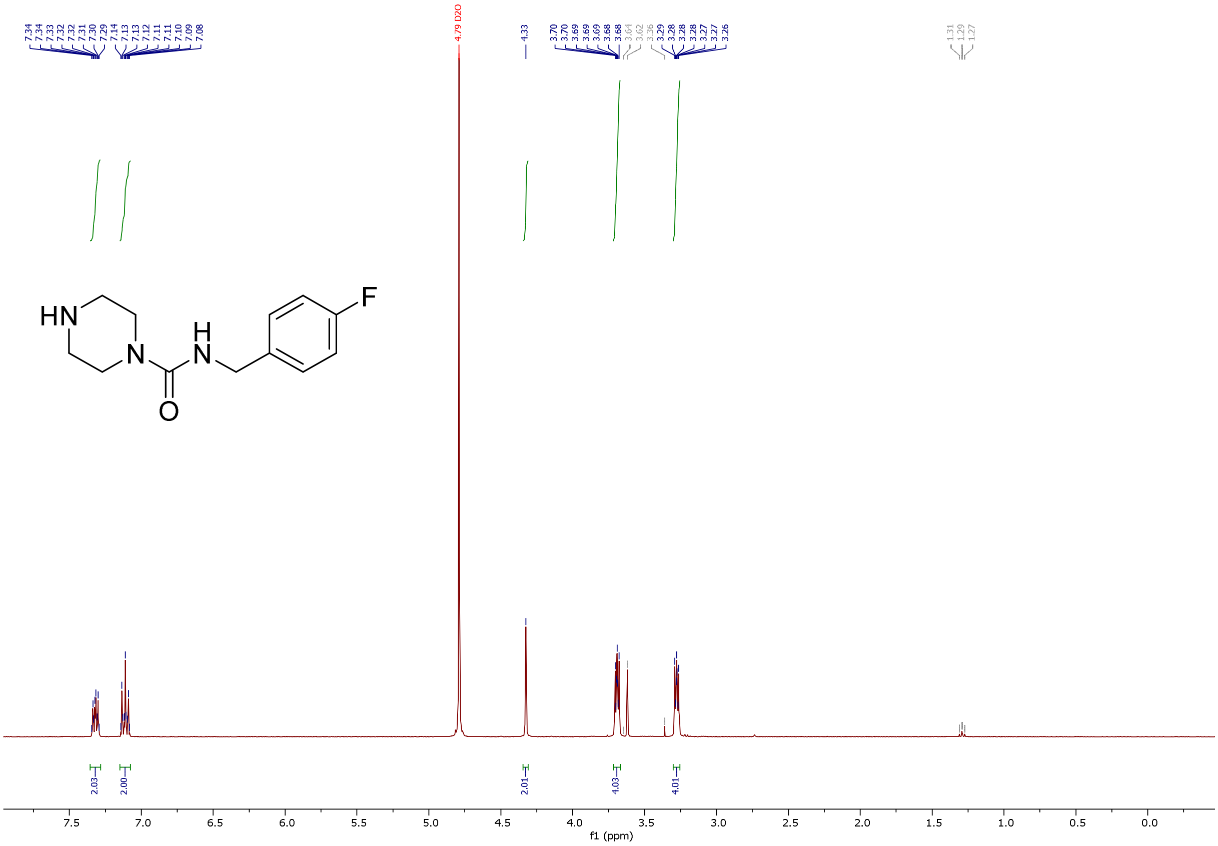

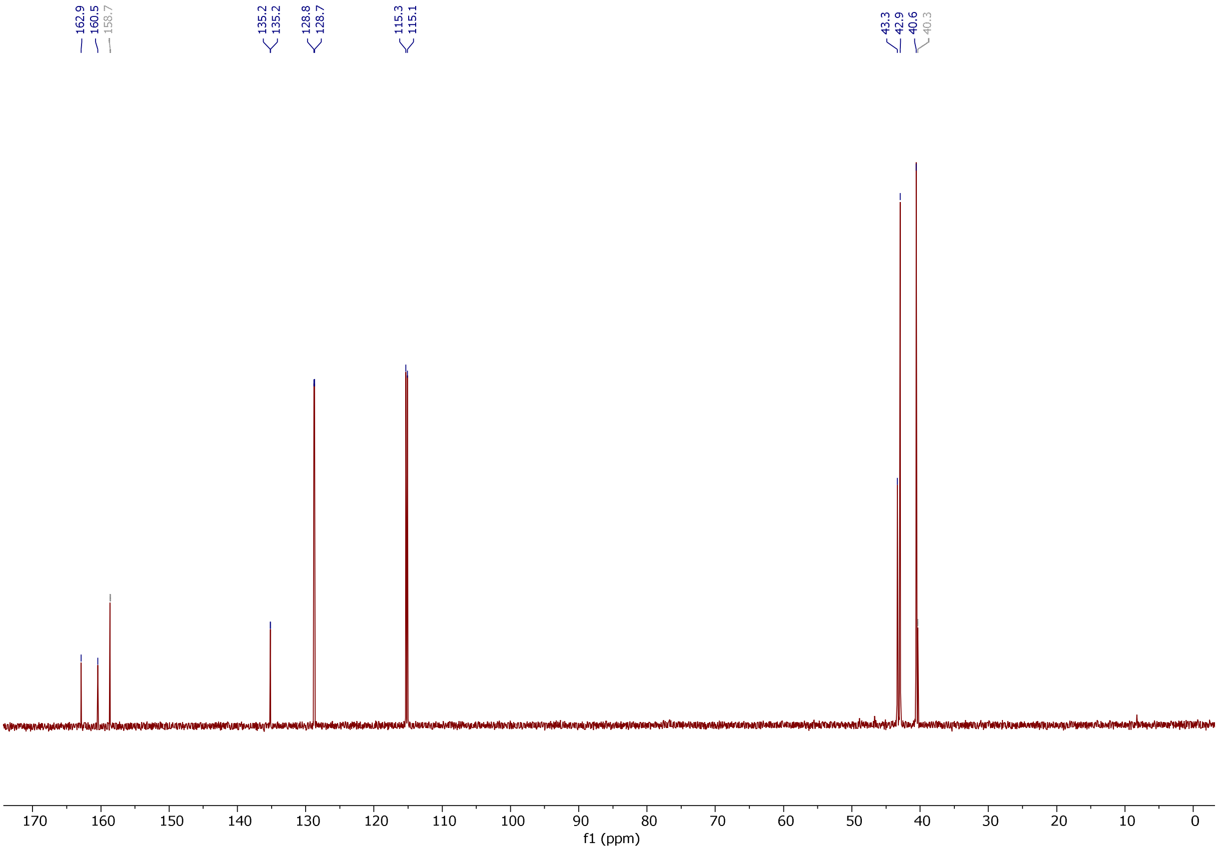

*tert-Butyl 4-((4-fluorobenzyl)carbamoyl)piperidine-1-carboxylate (**28**)*

^{1}H NMR (400 MHz, $CDCl_3$) and **^{13}C NMR** (100 MHz, $CDCl_3$)

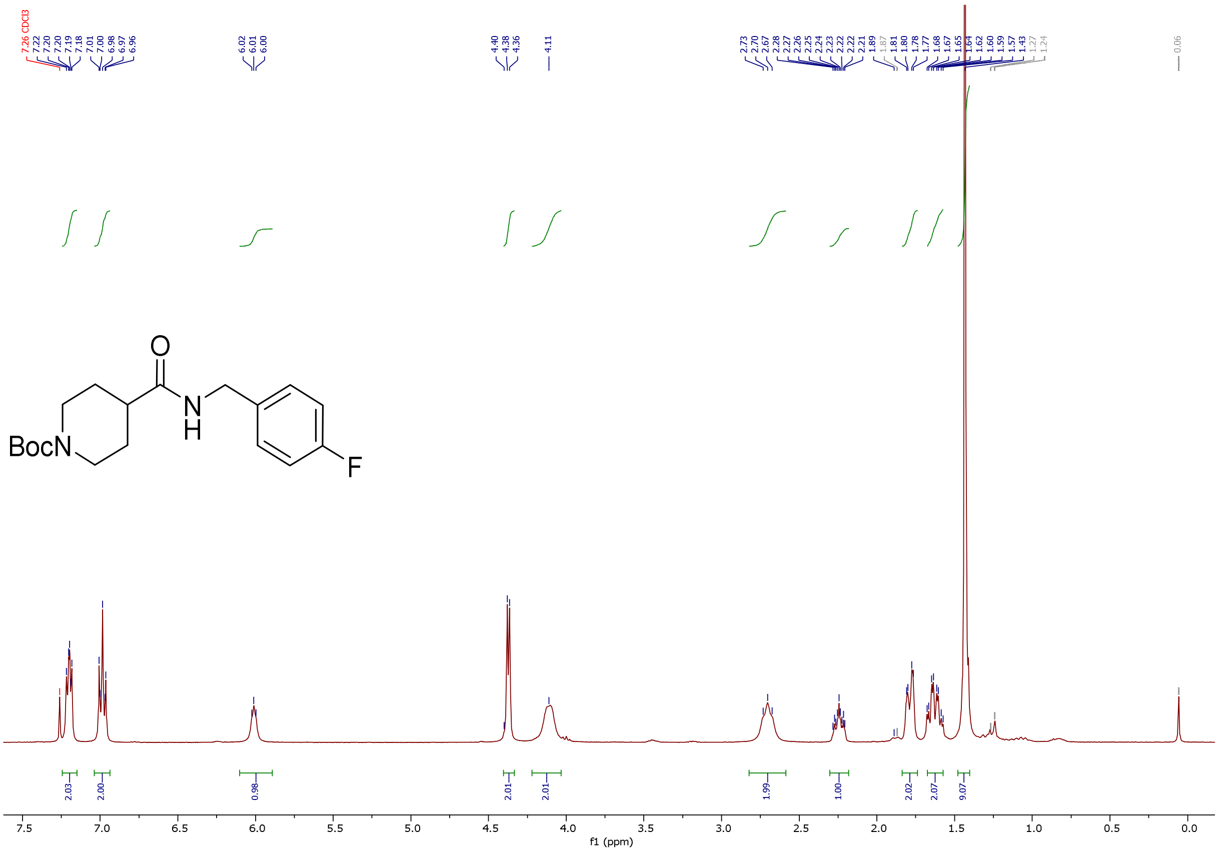

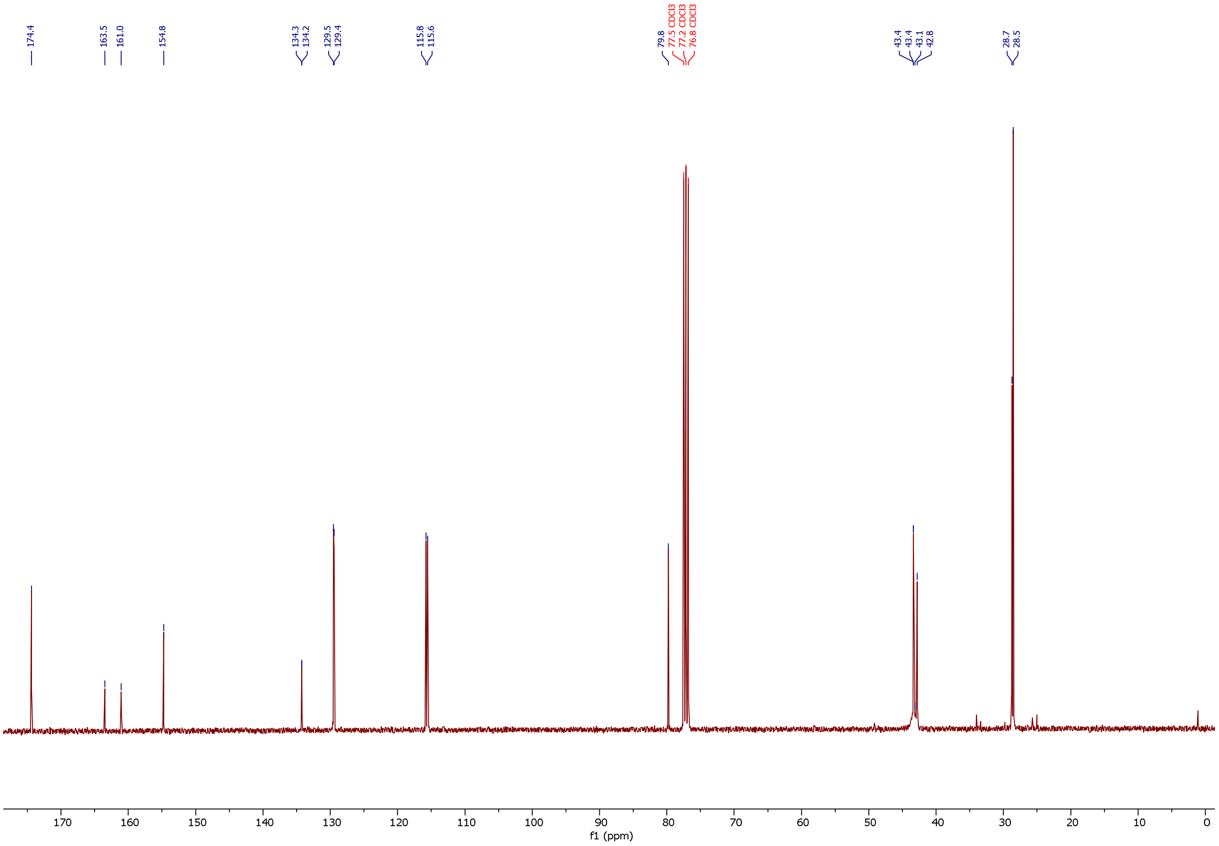

*tert-Butyl 4-((4-fluorophenethyl)carbamoyl)piperidine-1-carboxylate (**29**)*

^{1}H NMR (400 MHz, $CDCl_3$) and **^{13}C NMR** (100 MHz, $CDCl_3$)

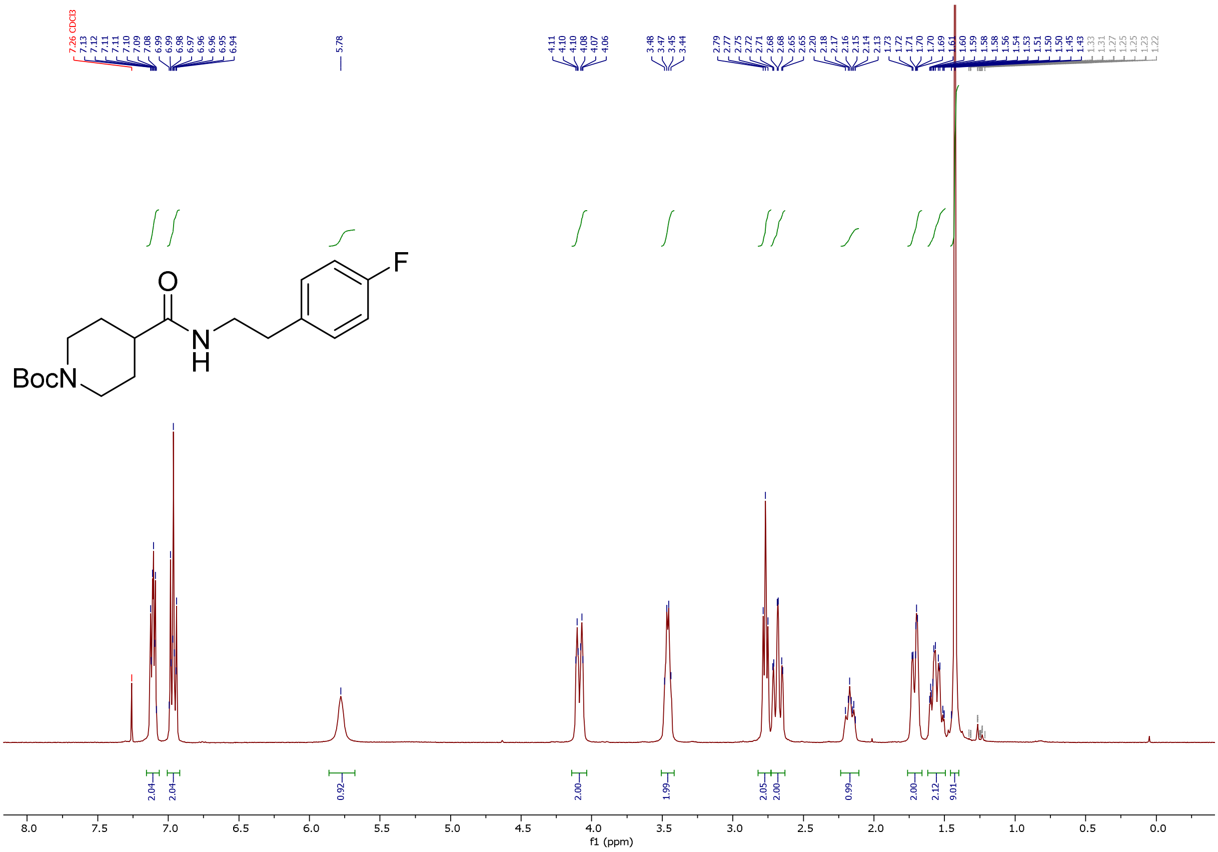

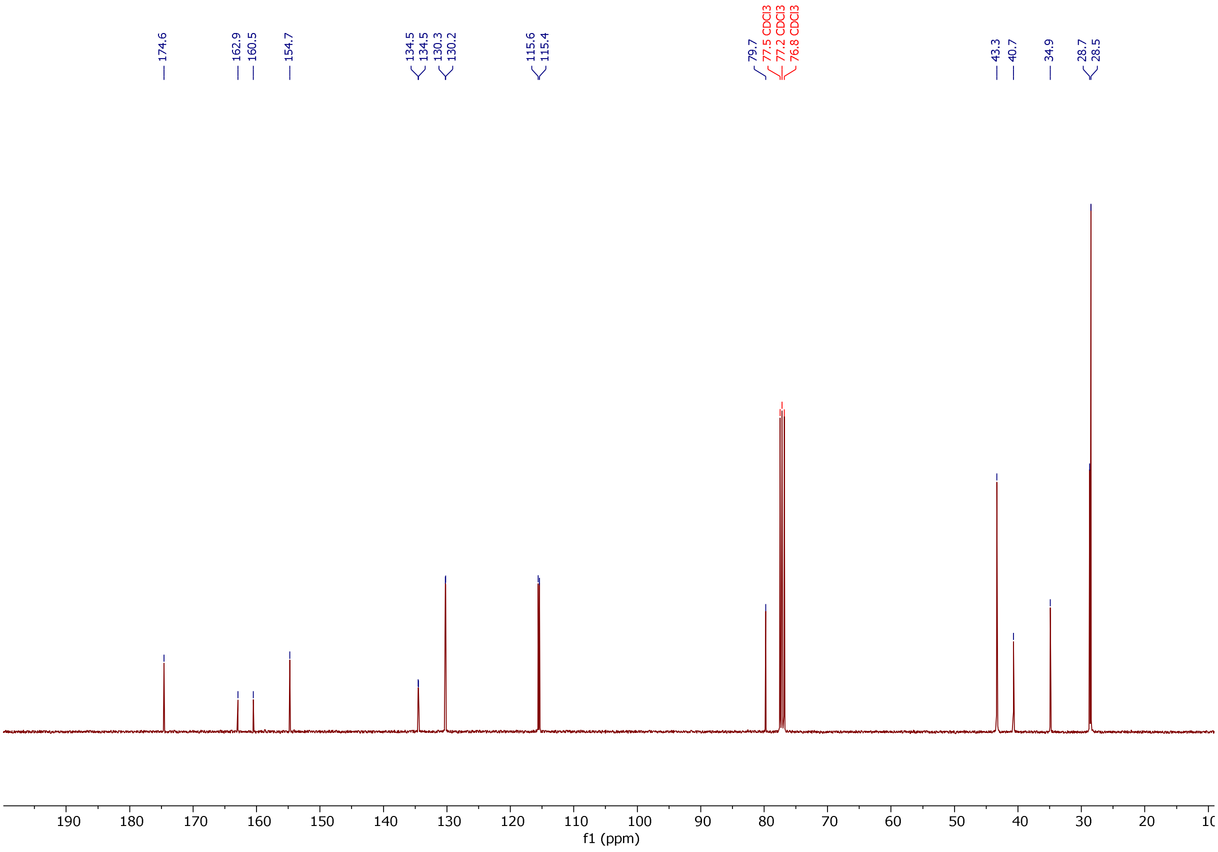

*tert-butyl 3-((4-fluorobenzyl)carbamoyl)azetidine-1-carboxylate (**30**)*

^{1}H NMR (400 MHz, $CDCl_3$) and **^{13}C NMR** (100 MHz, $CDCl_3$)

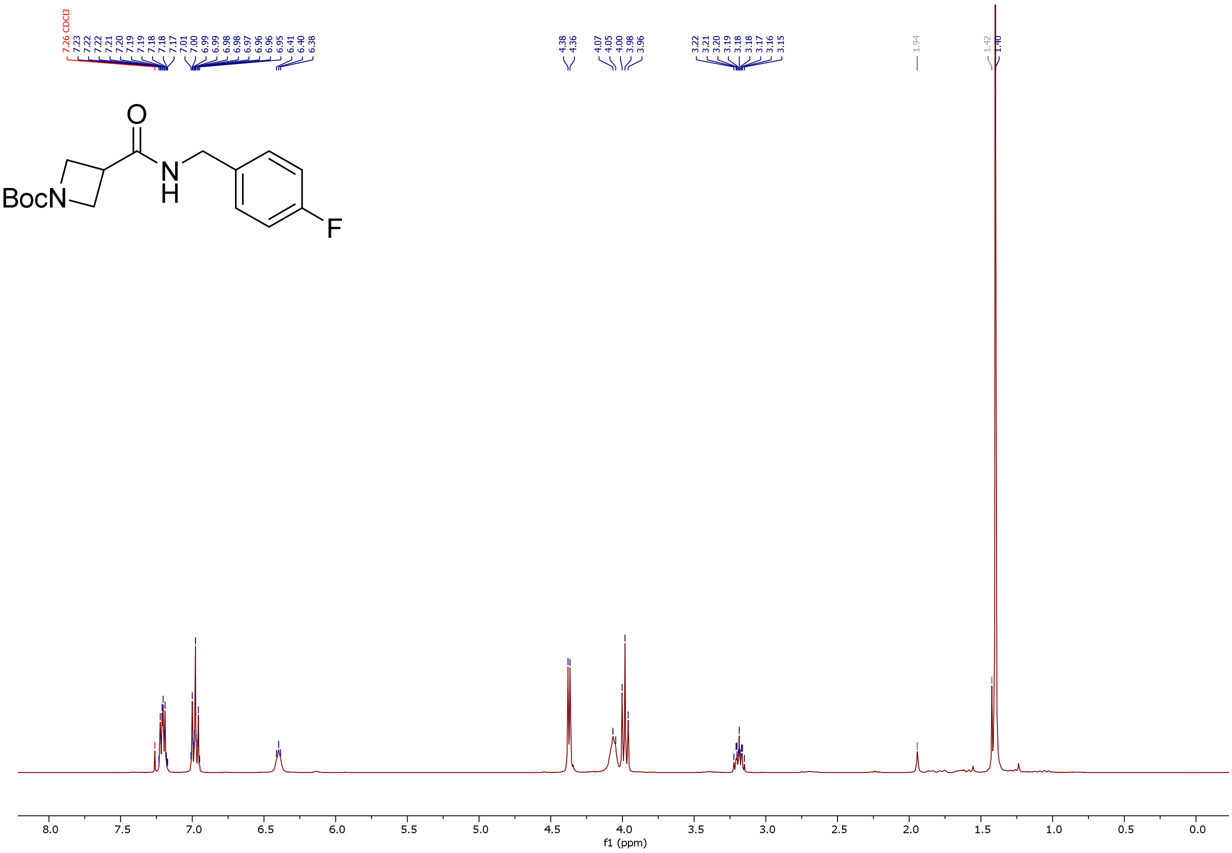

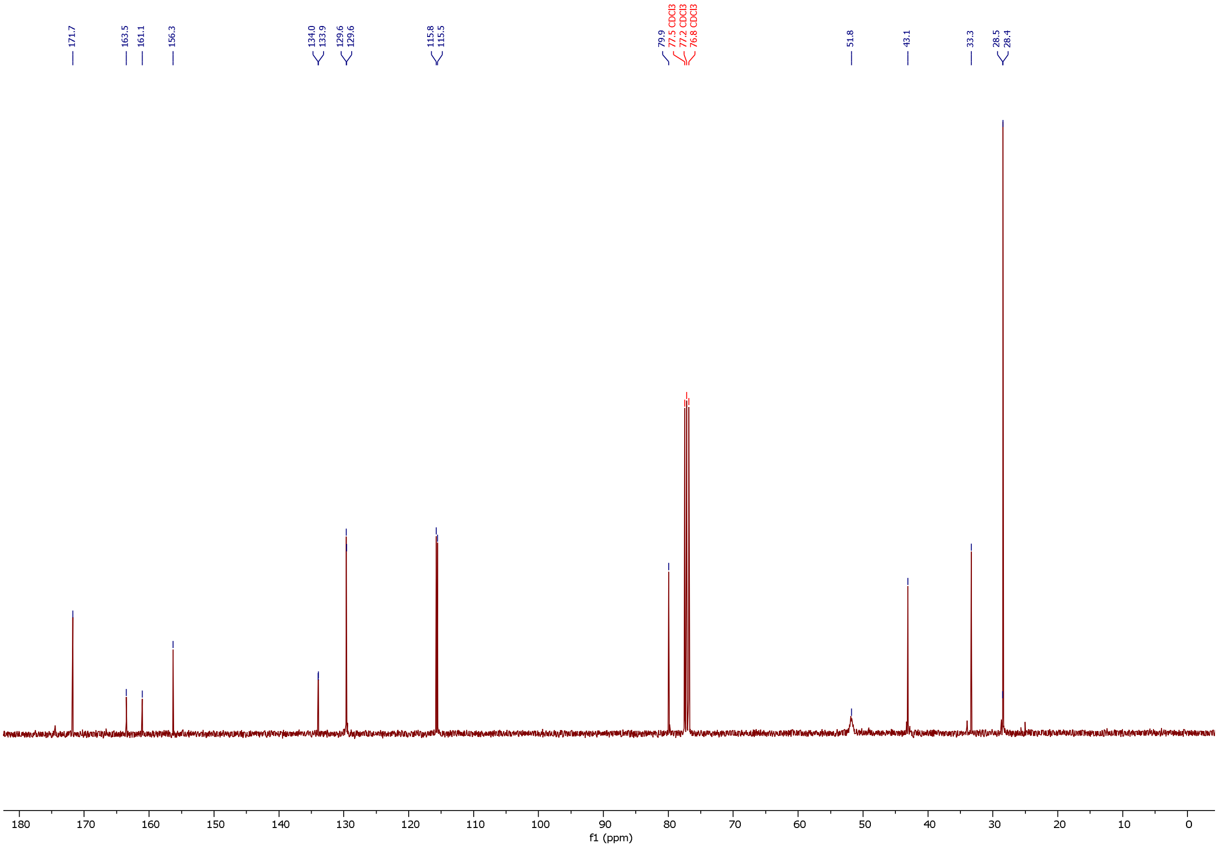

*N-(4-Fluorobenzyl)piperidine-4-carboxamide (**31**)*

^{1}H NMR (400 MHz, D_2O) and **^{13}C NMR** (100 MHz, D_2O)

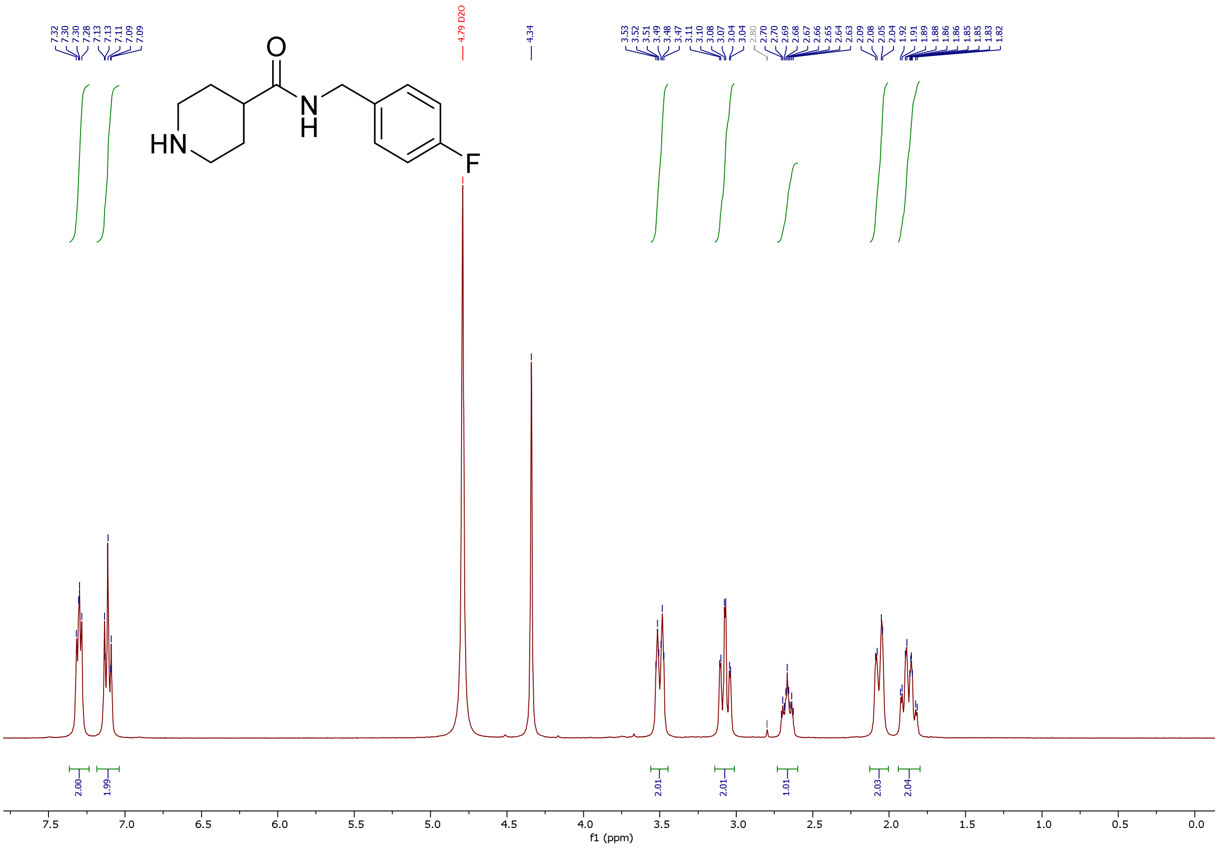

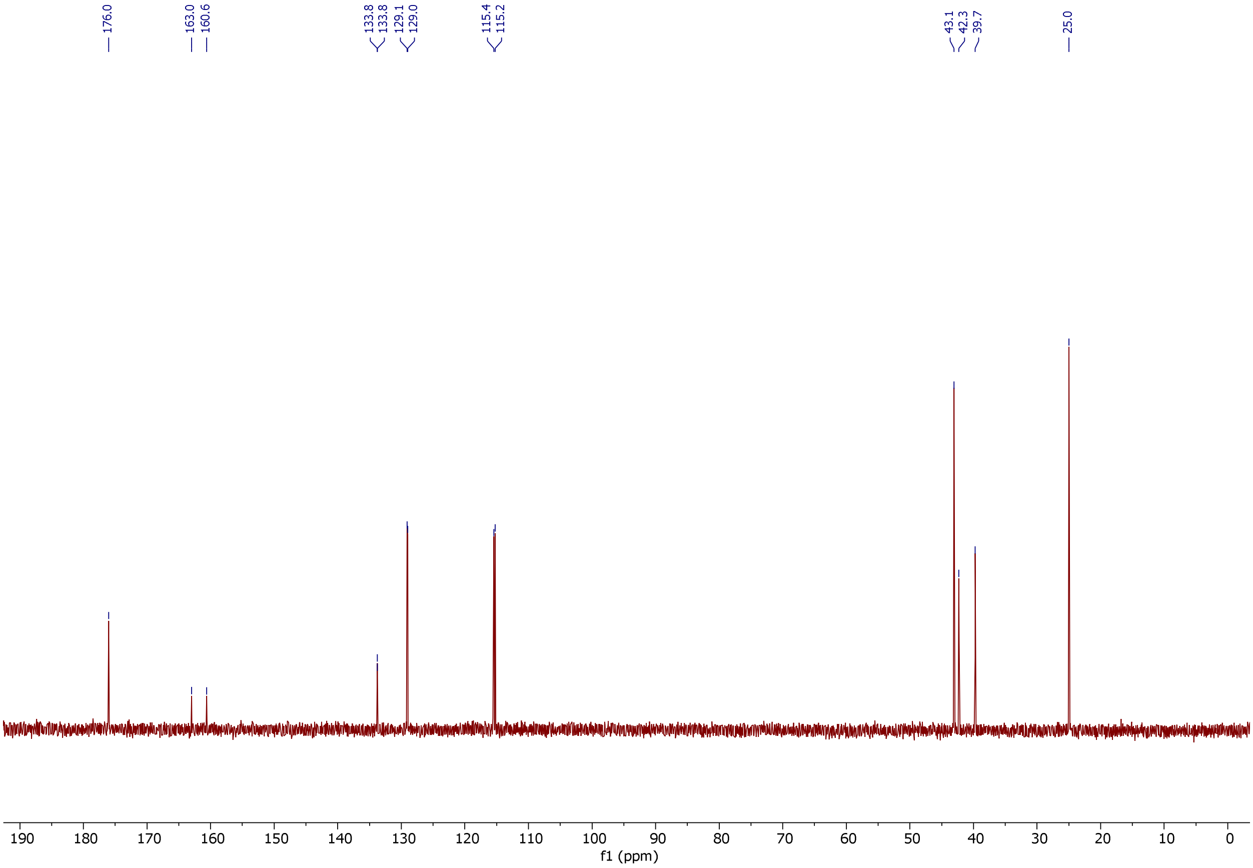

*N-(4-Fluorophenethyl)piperidine-4-carboxamide (**32**)*

^{1}H NMR (400 MHz, MeOD$_4$) and **^{13}C NMR** (100 MHz, MeOD$_4$)

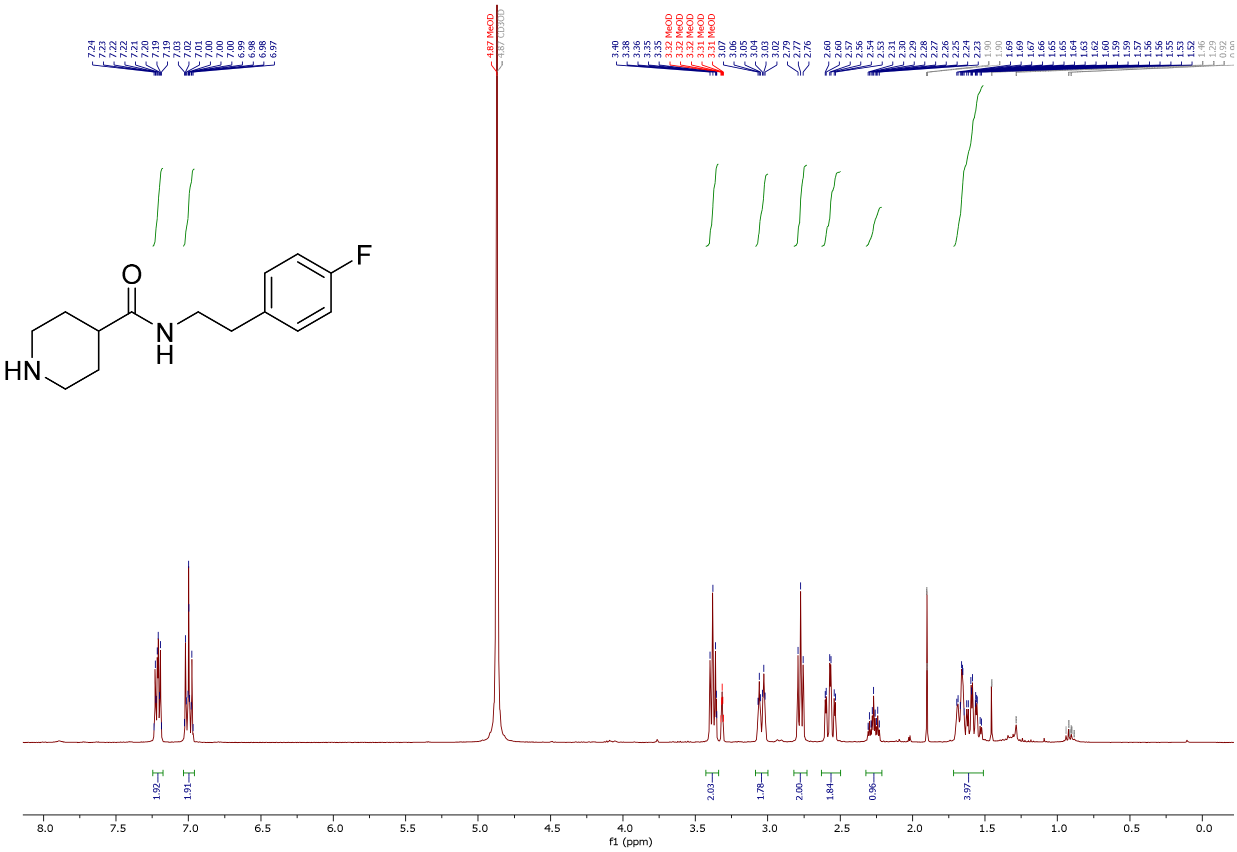

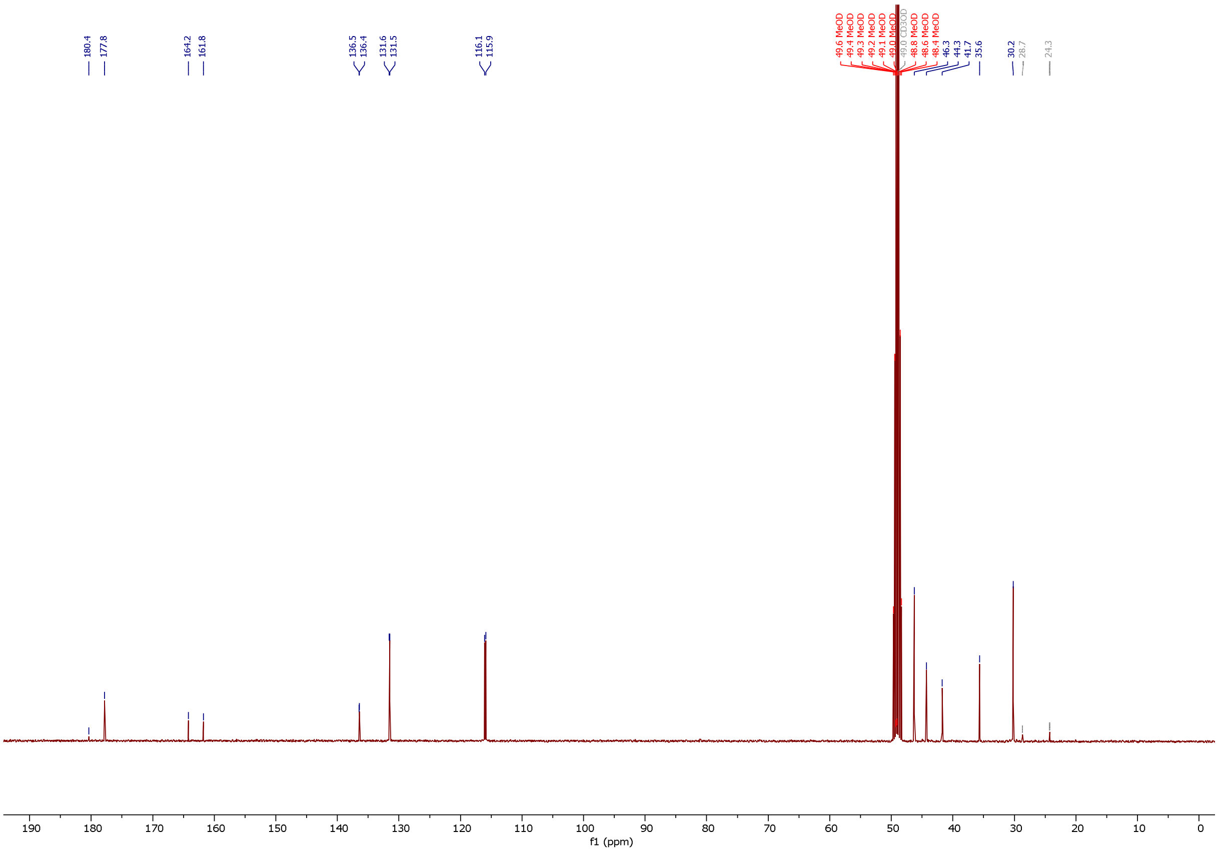

*N-(4-Fluorobenzyl)azetidine-3-carboxamide (**33**)*

¹H NMR (400 MHz, D_2O) and **¹³C NMR** (100 MHz, D_2O)

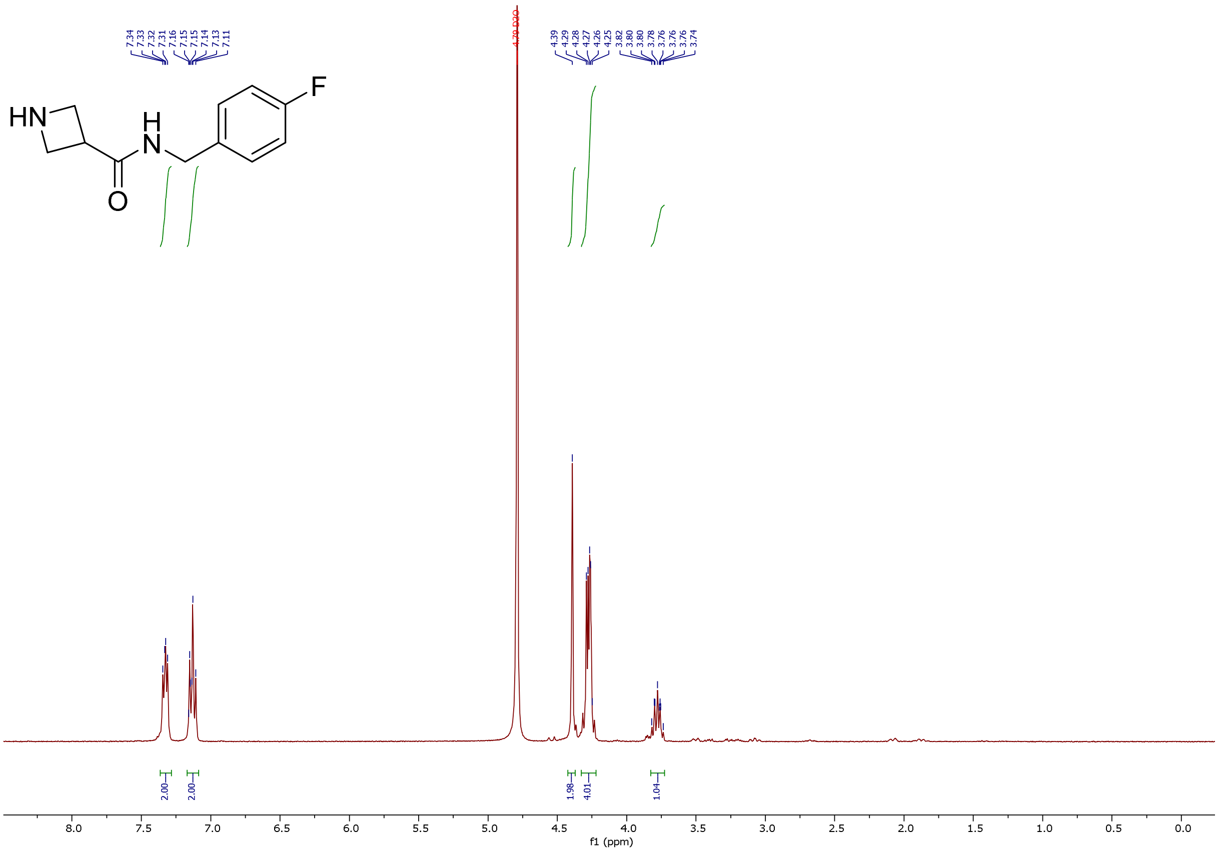

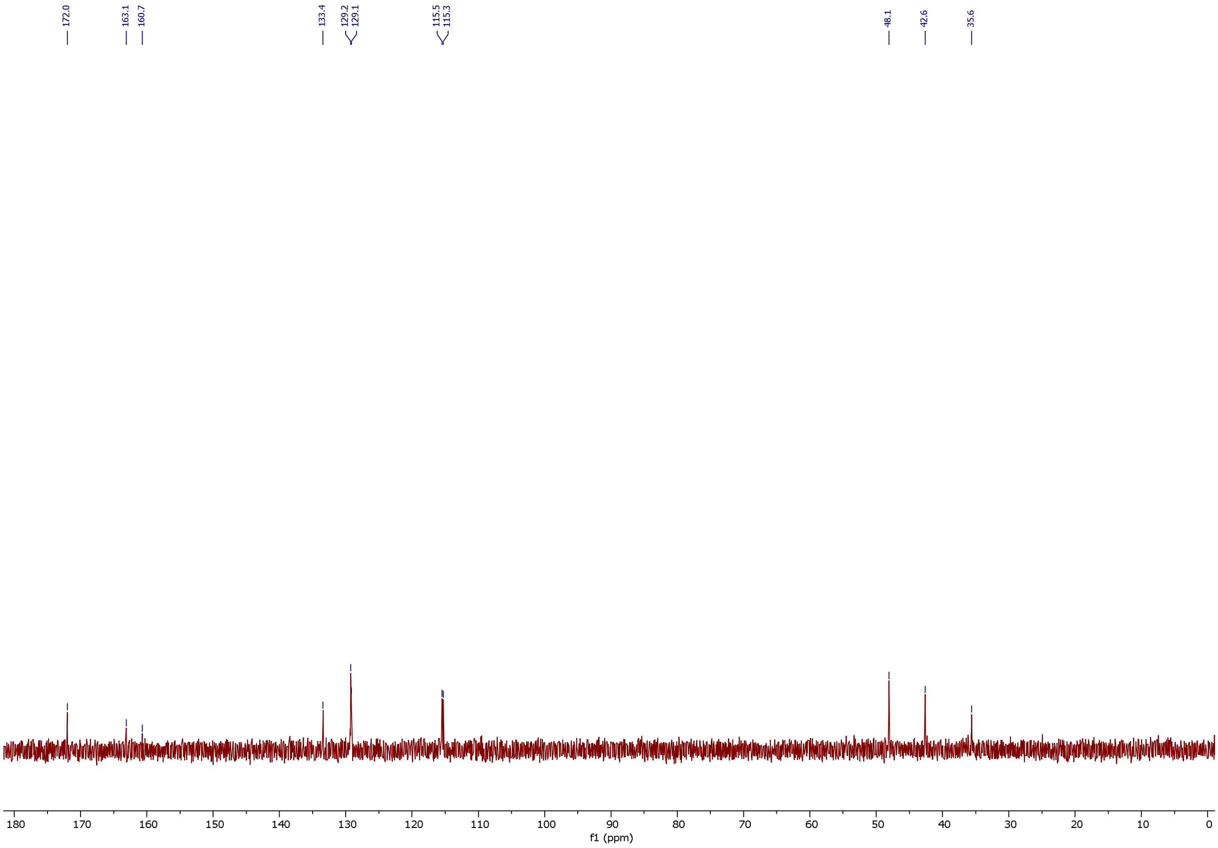

*Ethyl 6-chloro-2-methylnicotinate (**34**)*

^{1}H NMR (400 MHz, $CDCl_3$) and **^{13}C NMR** (100 MHz, $CDCl_3$)

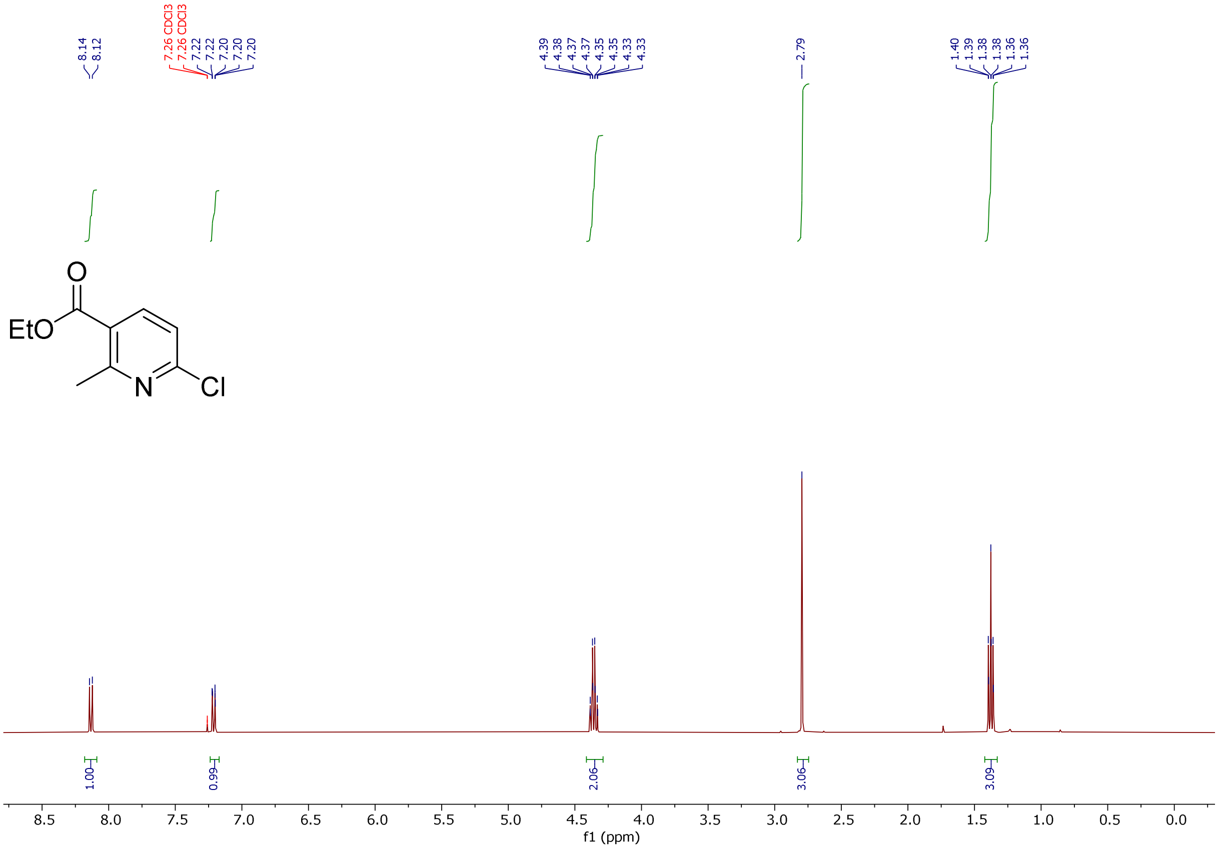

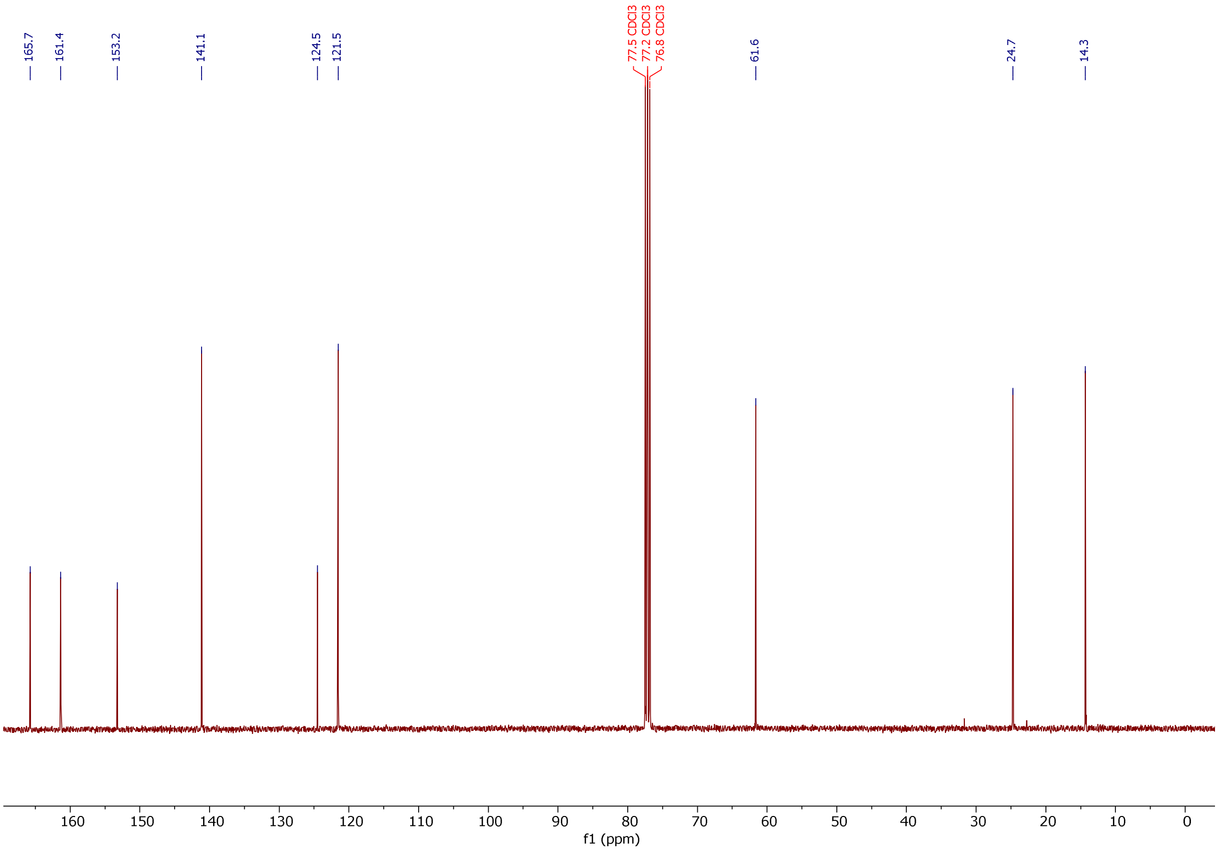

*Ethyl 5,6-dichloronicotinate (**35**)*

^{1}H NMR (400 MHz, $CDCl_3$) and **^{13}C NMR** (101 MHz, $CDCl_3$)

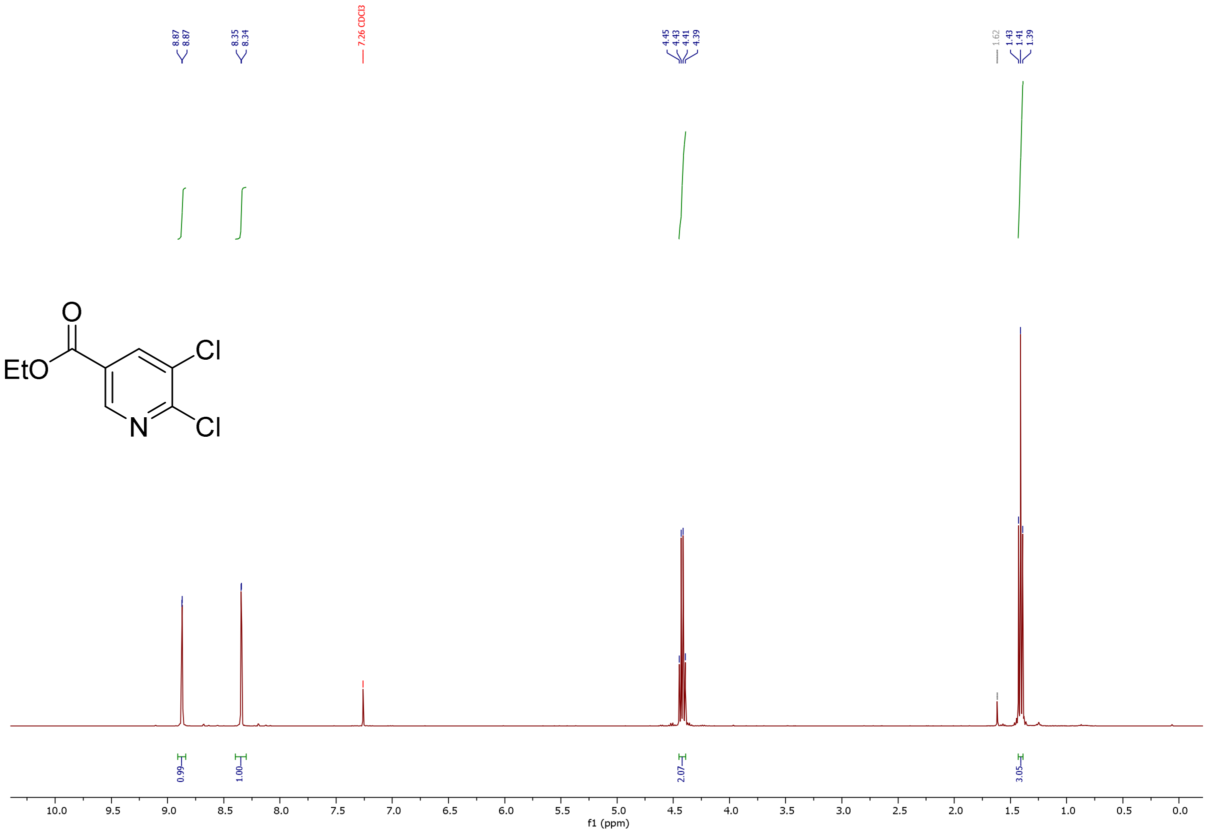

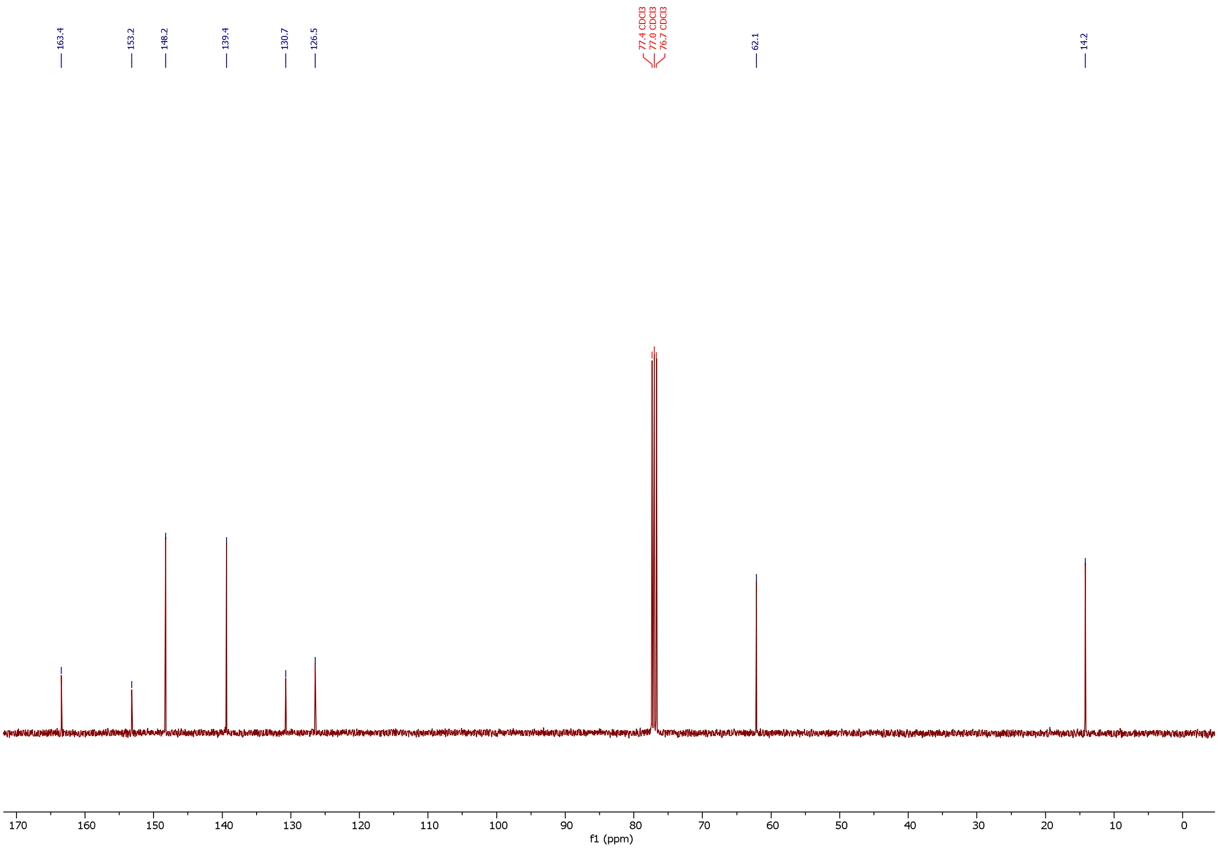

*Ethyl 5,6-dichloro-2-methylnicotinate (**36**)*

^{1}H NMR (400 MHz, $CDCl_3$) and **^{13}C NMR** (100 MHz, $CDCl_3$)

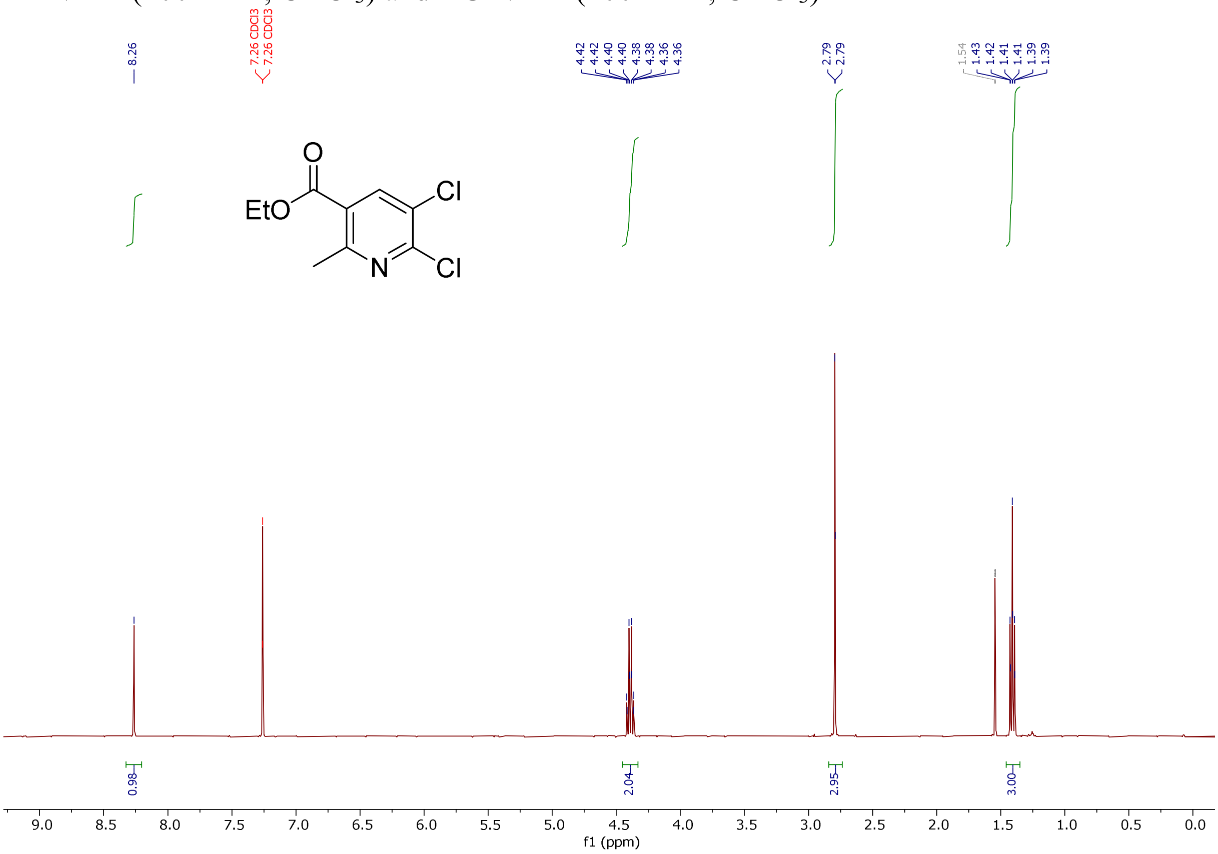

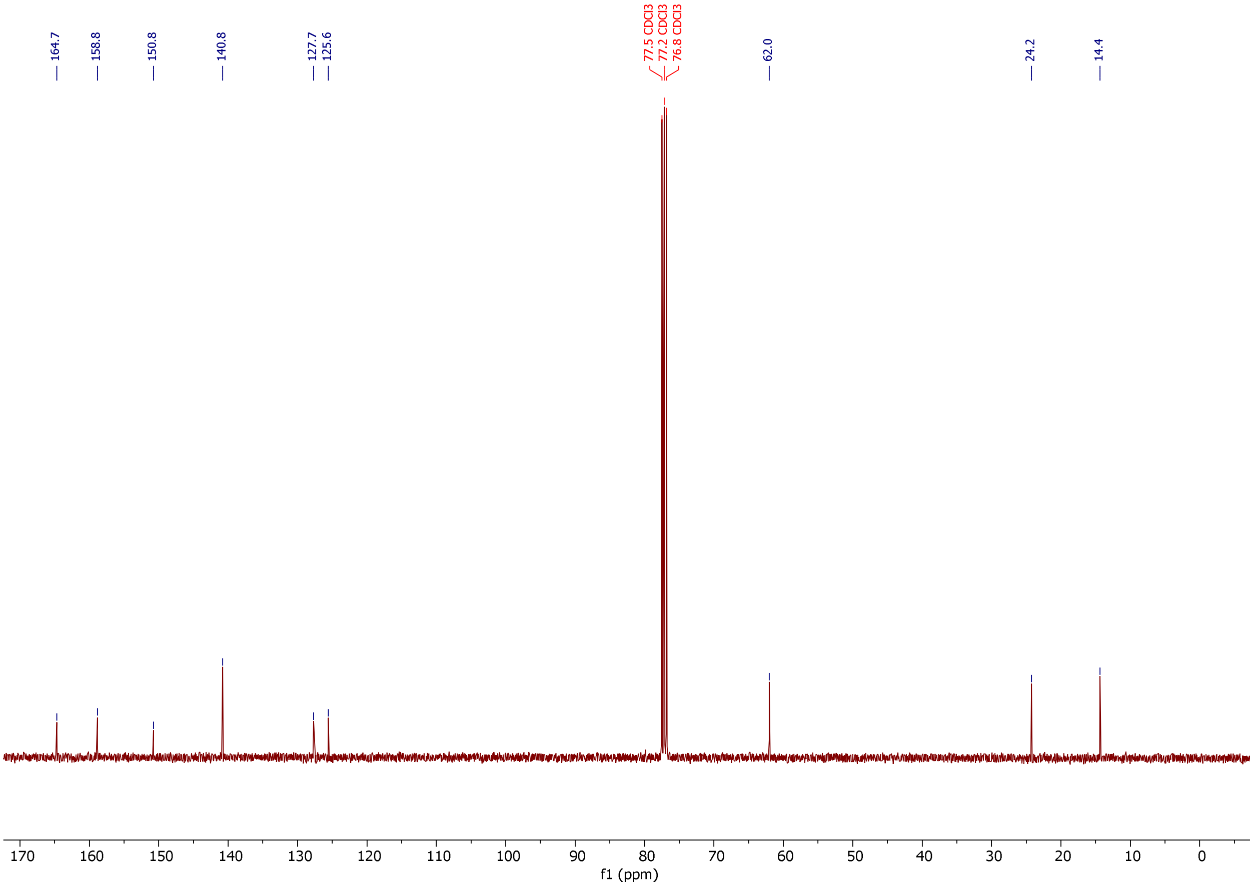

*Ethyl 6-chloro-5-cyano-2-methylnicotinate (**37**)*

^{1}H NMR (400 MHz, $CDCl_3$) and **^{13}C NMR** (100 MHz, $CDCl_3$)

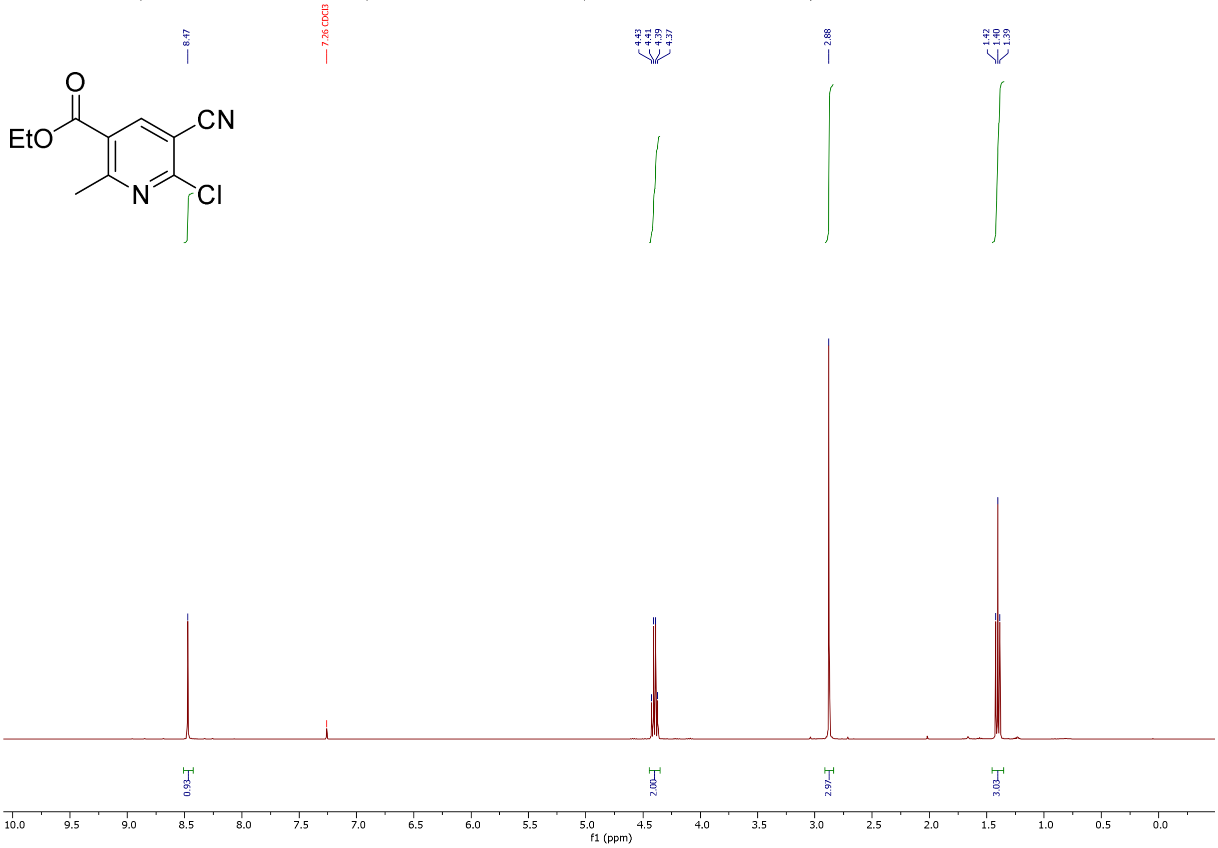

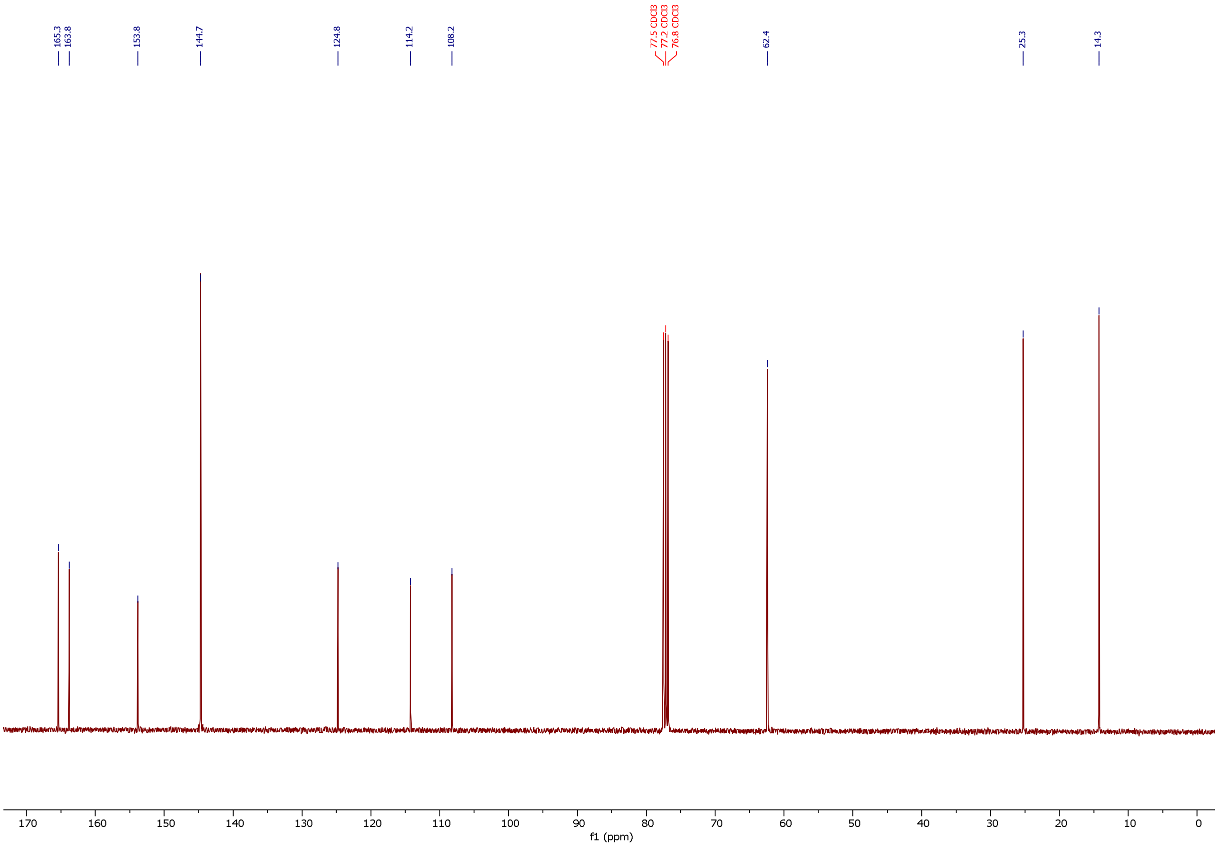

*2-Chloro-5-oxo-5,7-dihydrofuro[3,4-b]pyridine-3-carbonitrile (**38**)*

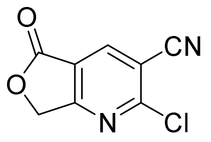

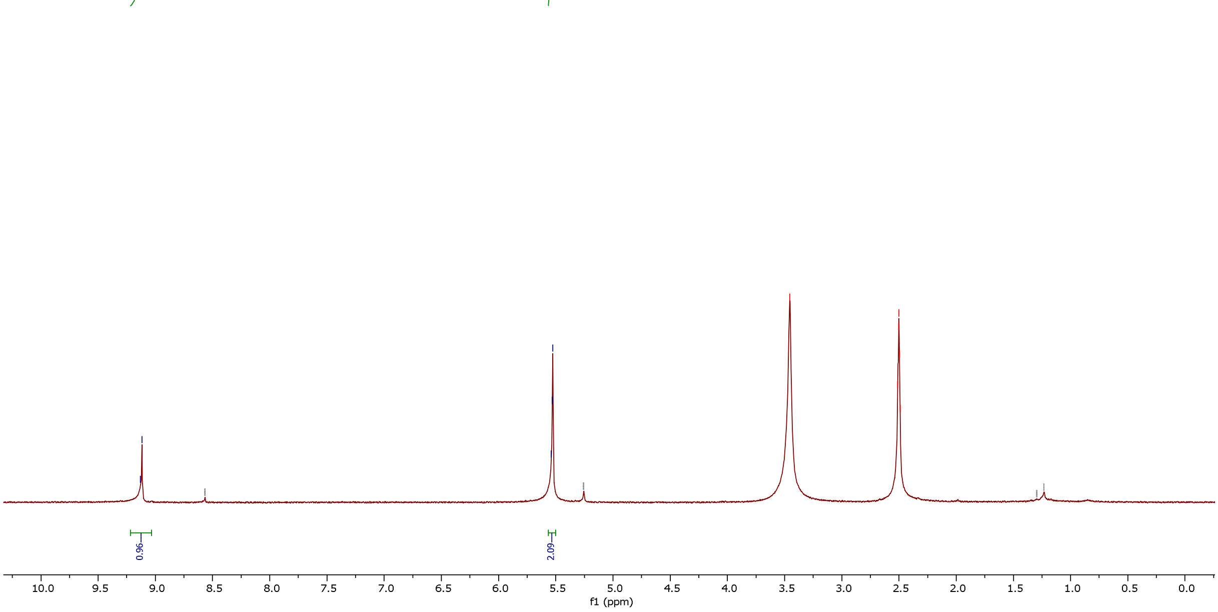

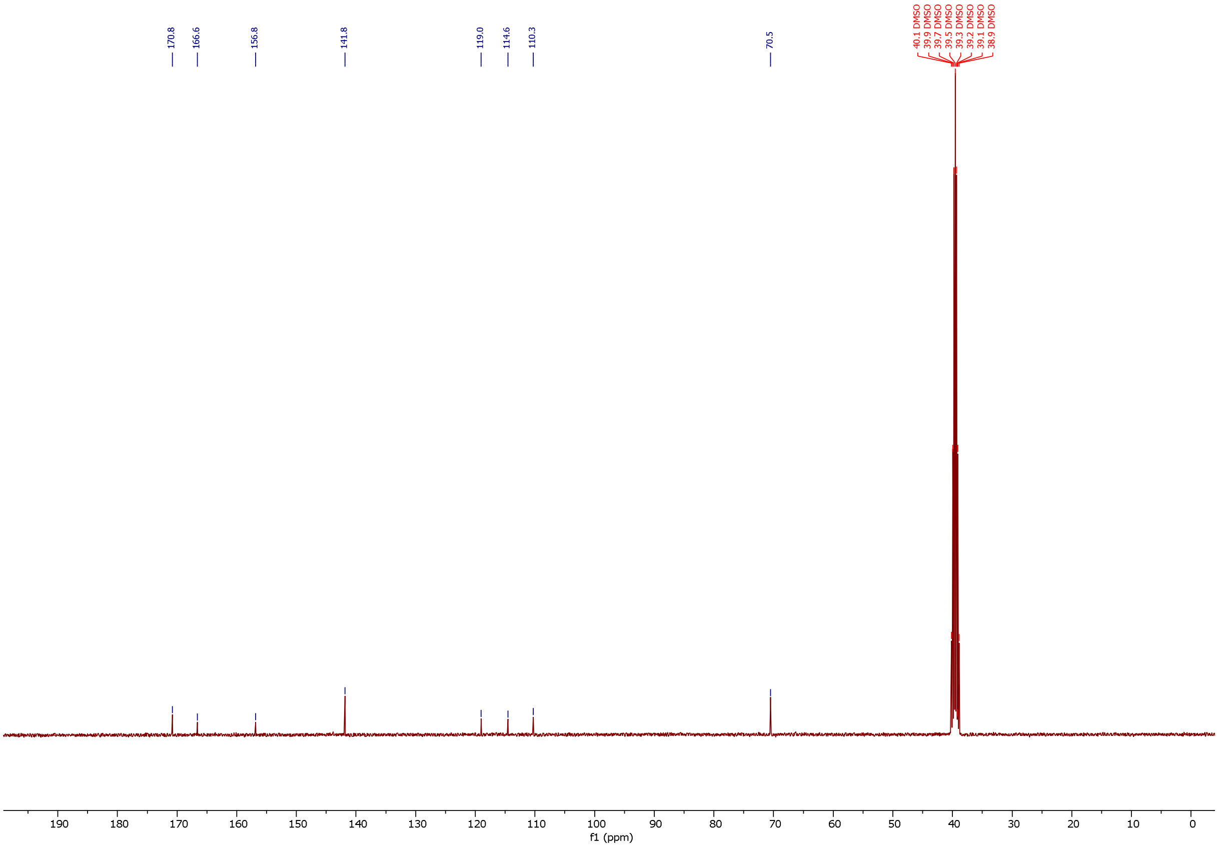

*Ethyl 4-fluoro-2-hydroxybenzoate (**40**)*

^{1}H NMR (400 MHz, $CDCl_3$) and **^{13}C NMR** (100 MHz, $CDCl_3$)

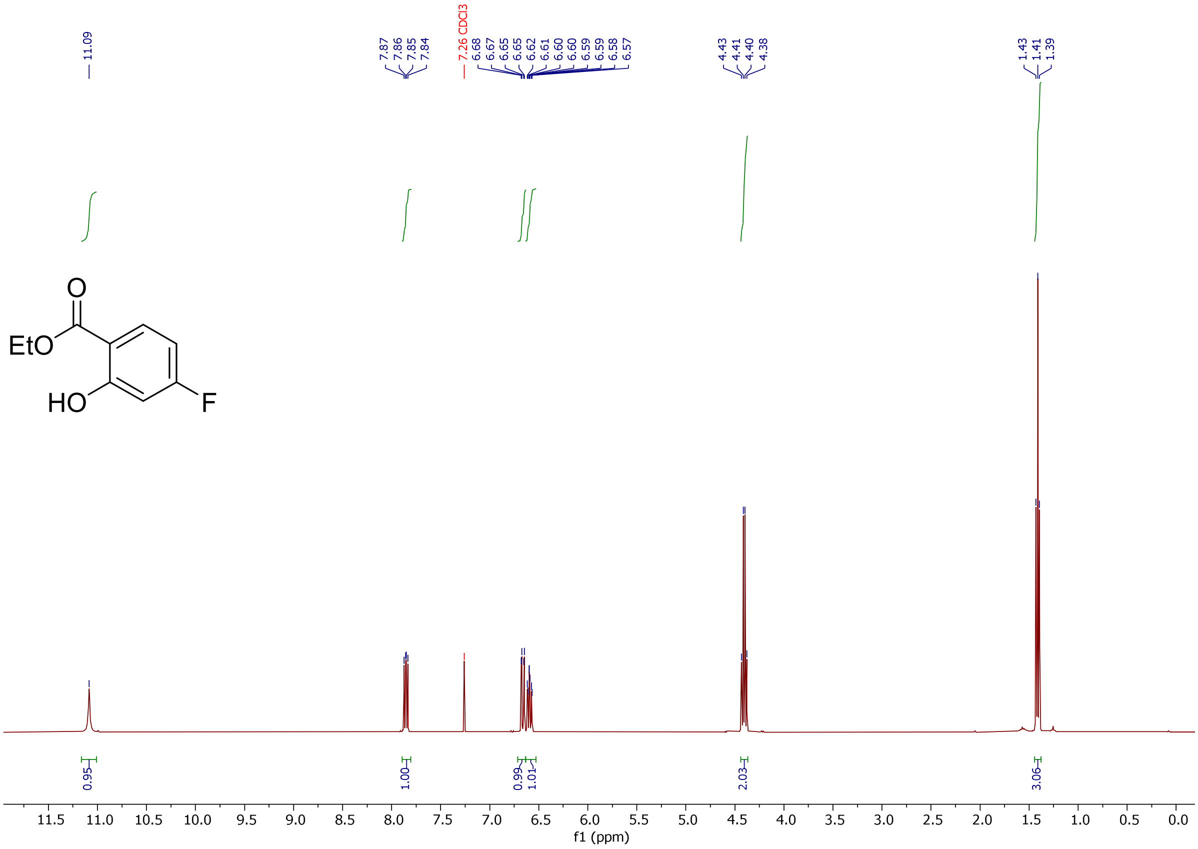

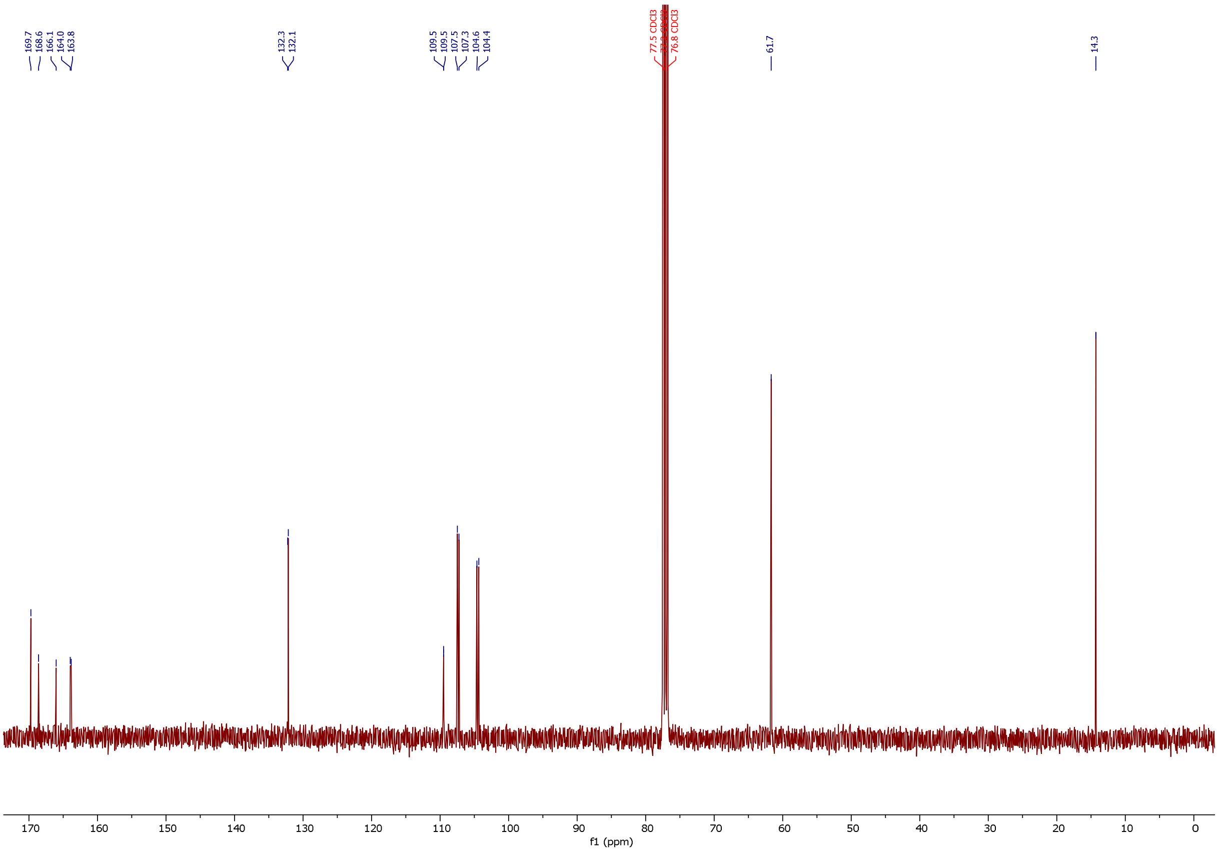

*Ethyl 4-fluoro-2-propoxybenzoate (**41**)*

^{1}H NMR (400 MHz, $CDCl_3$) and **^{13}C NMR** (100 MHz, $CDCl_3$)

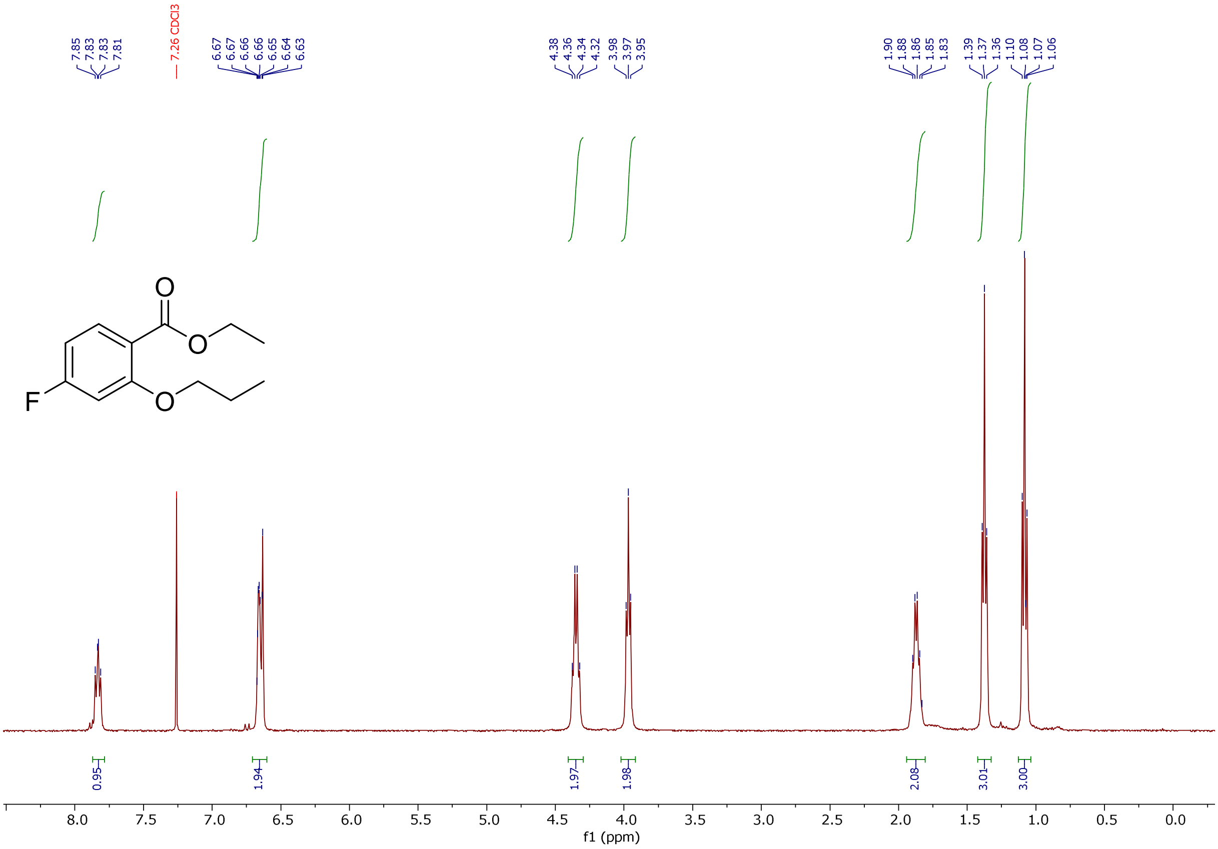

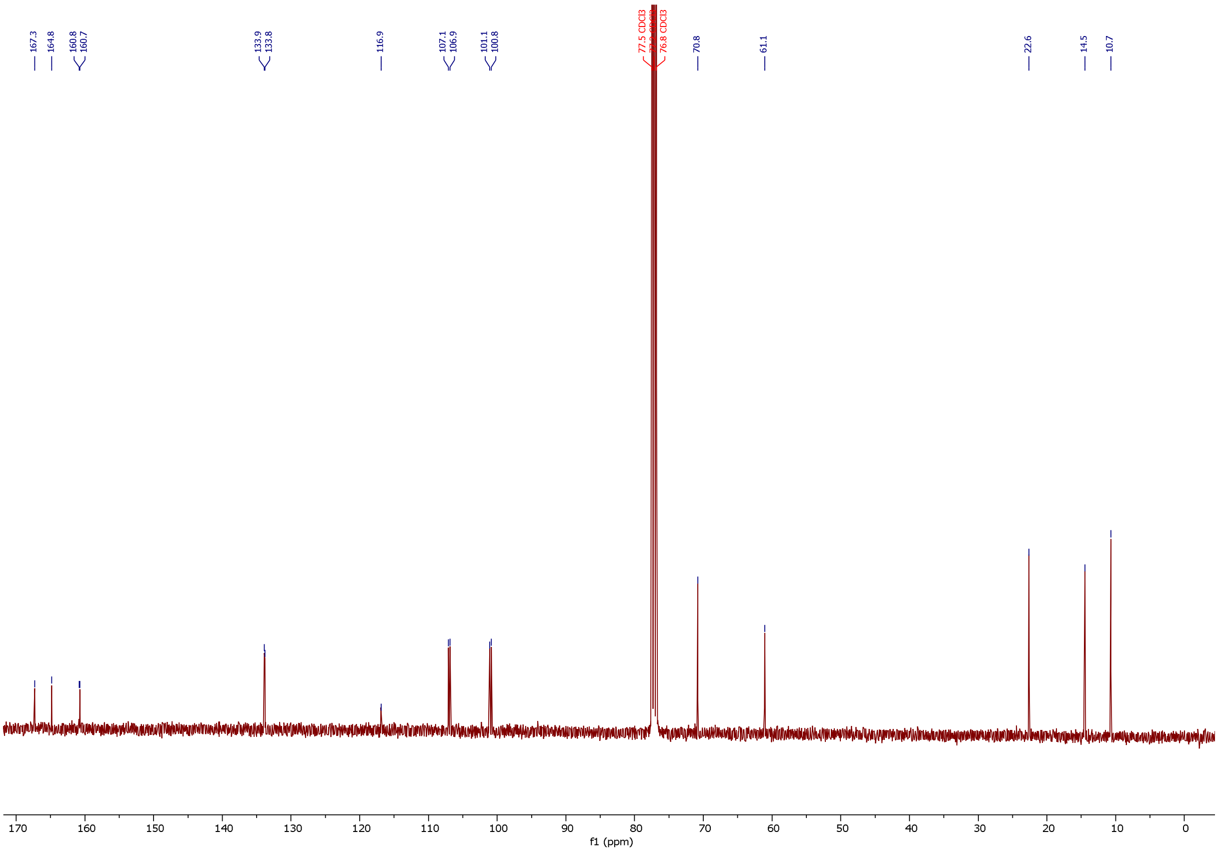

*Ethyl 4-fluoro-2-propoxybenzoate (**42**)*

^{1}H NMR (400 MHz, $CDCl_3$) and **^{13}C NMR** (100 MHz, $CDCl_3$)

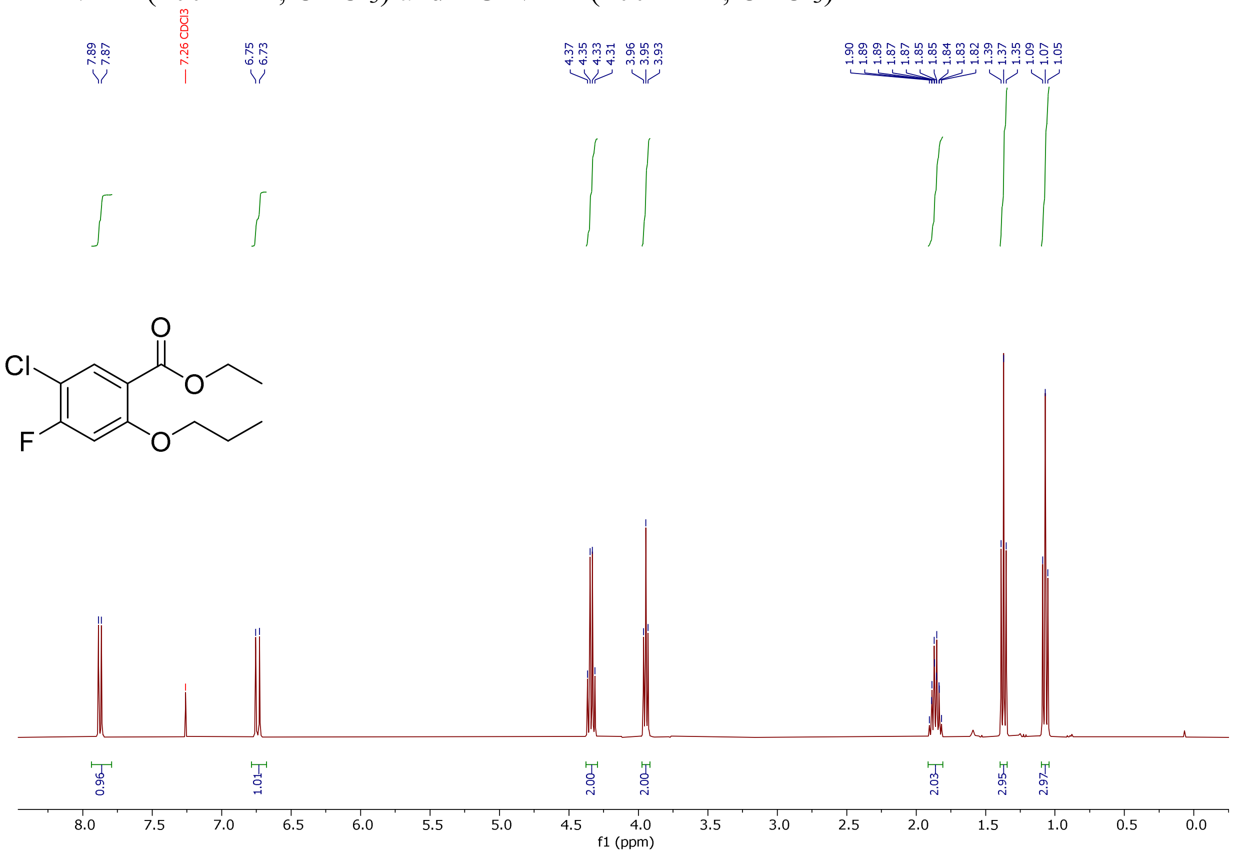

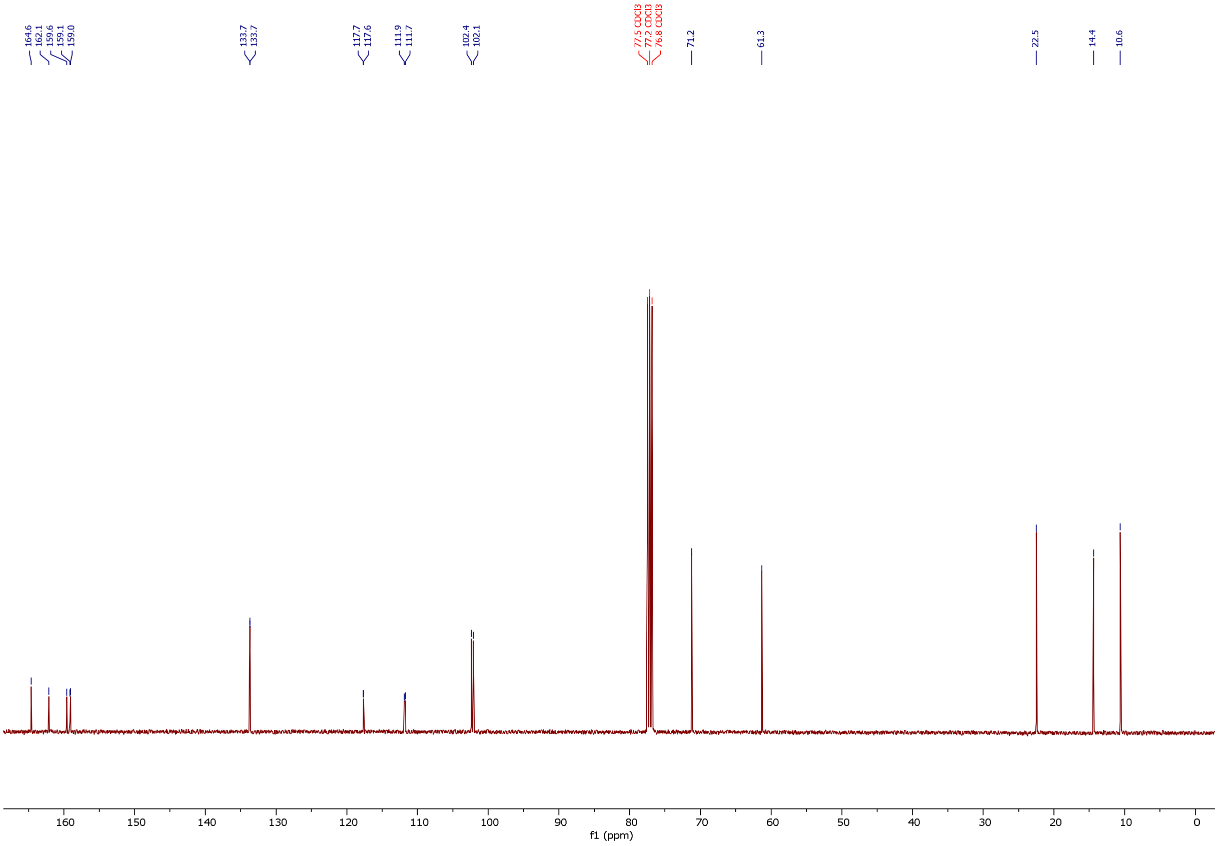

*tert-Butyl 4-((4-(4,4,5,5-tetramethyl-1,3,2-dioxaborolan-2-yl)benzyl)carbamoyl)piperidine-1-carboxylate (**43**)*

^{1}H NMR (400 MHz, $CDCl_3$) and **^{13}C NMR** (100 MHz, $CDCl_3$)

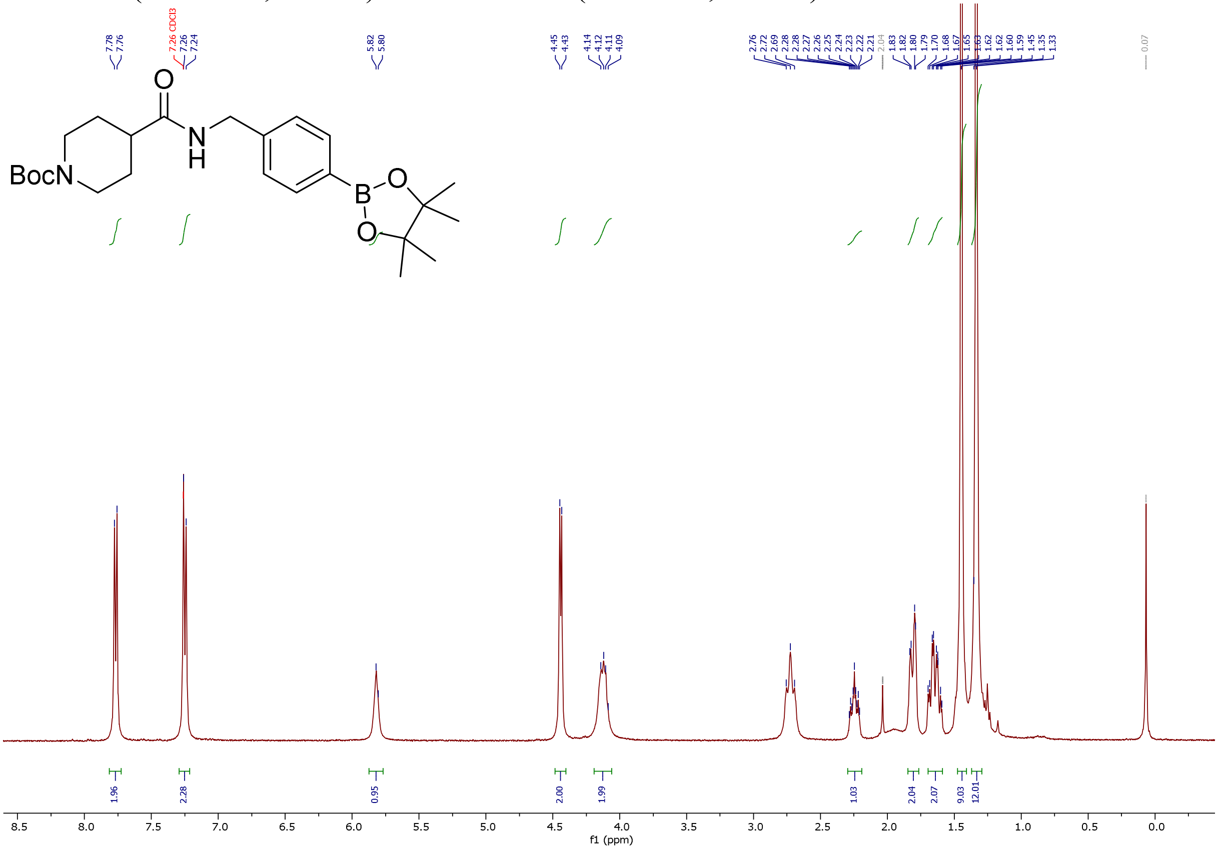

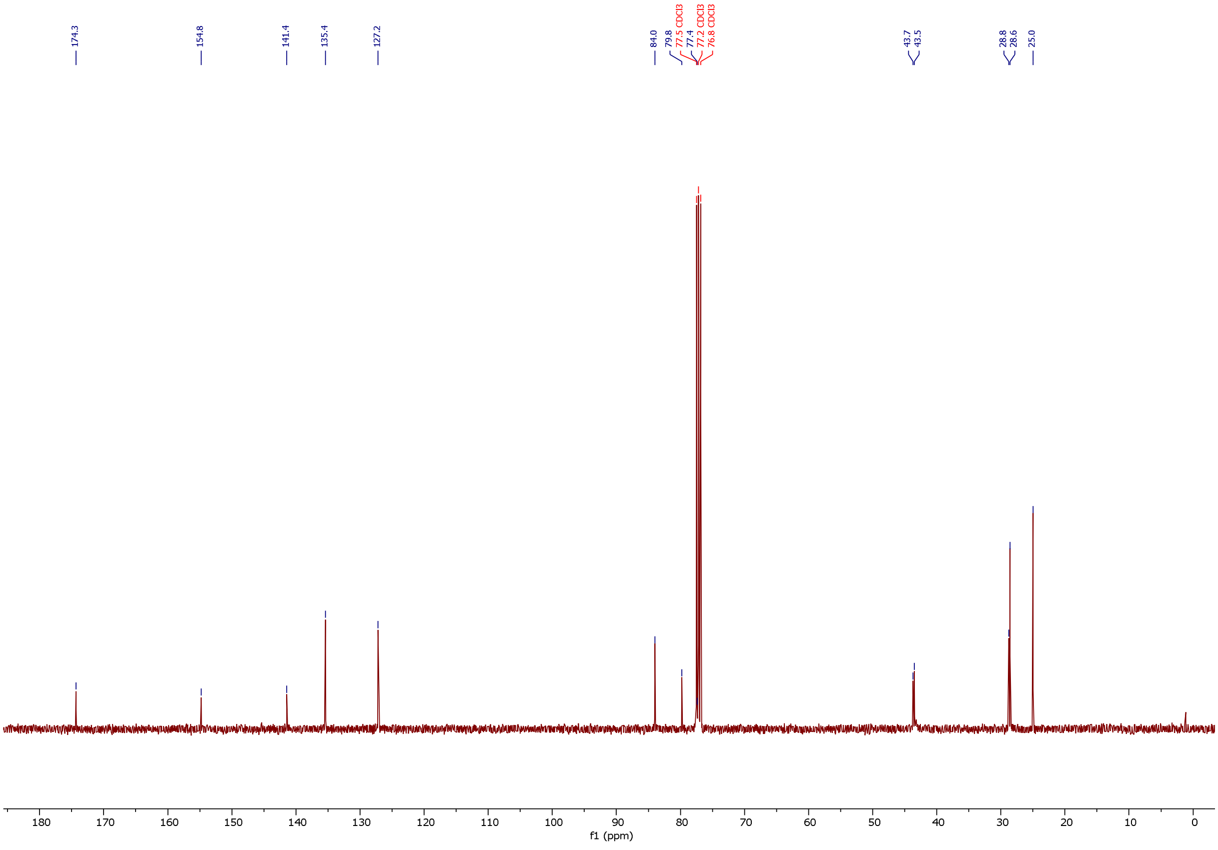

*N-(4-(4,4,5,5-Tetramethyl-1,3,2-dioxaborolan-2-yl)benzyl)piperidine-4-carboxamide (**44**)*

^{1}H NMR (400 MHz, MeOD$_4$) and **^{13}C NMR** (100 MHz, MeOD$_4$)

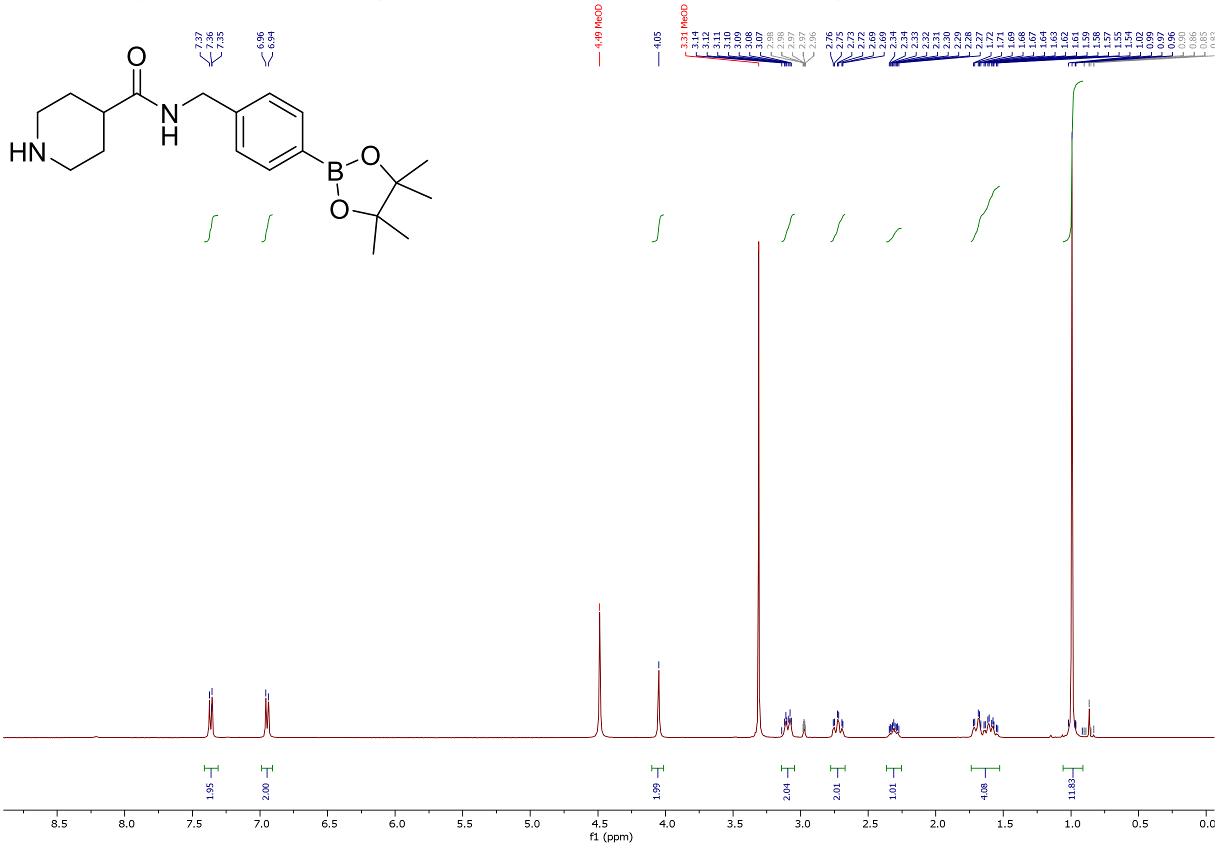

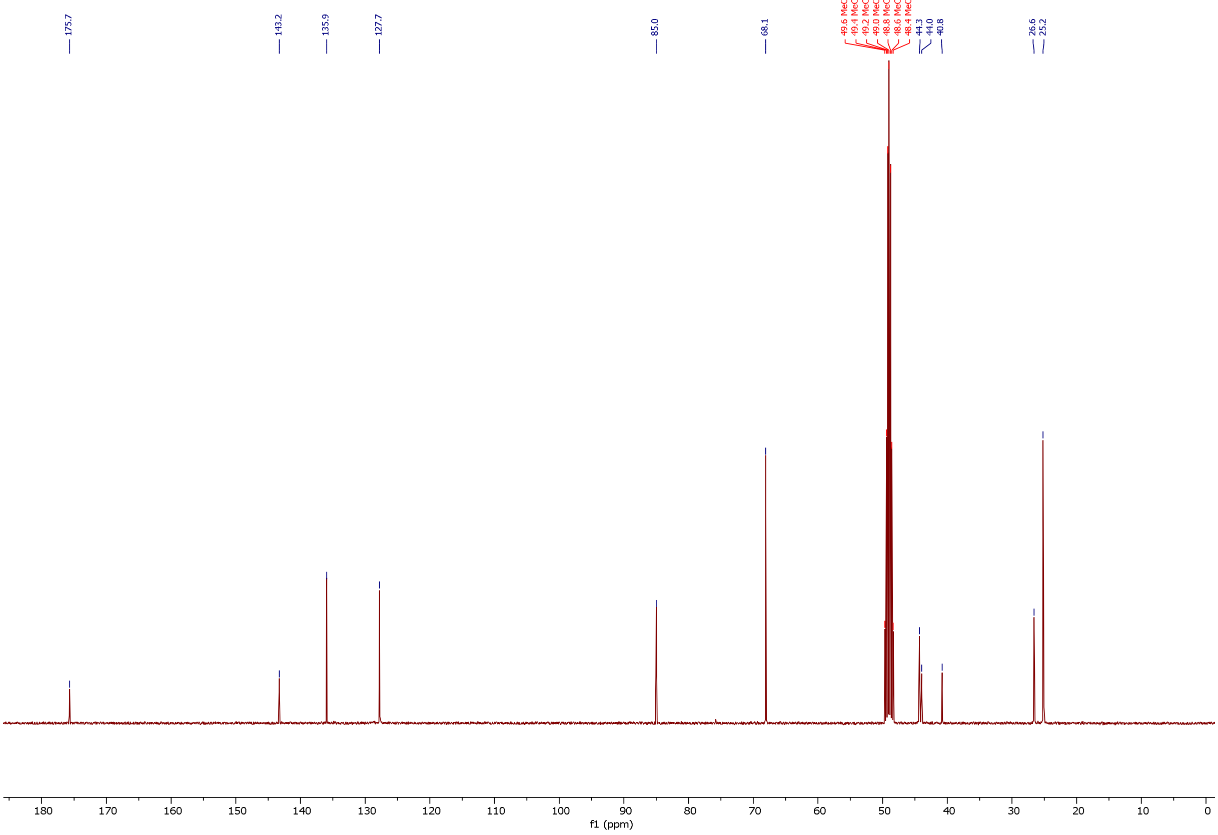

*Ethyl 5-cyano-2-methyl-6-(4-((4-(4,4,5,5-tetramethyl-1,3,2-dioxaborolan-2-yl)benzyl)carbamoyl)piperidin-1-yl)nicotinate (**45**)*

^{1}H NMR (400 MHz, $CDCl_3$) and **^{13}C NMR** (100 MHz, $CDCl_3$)

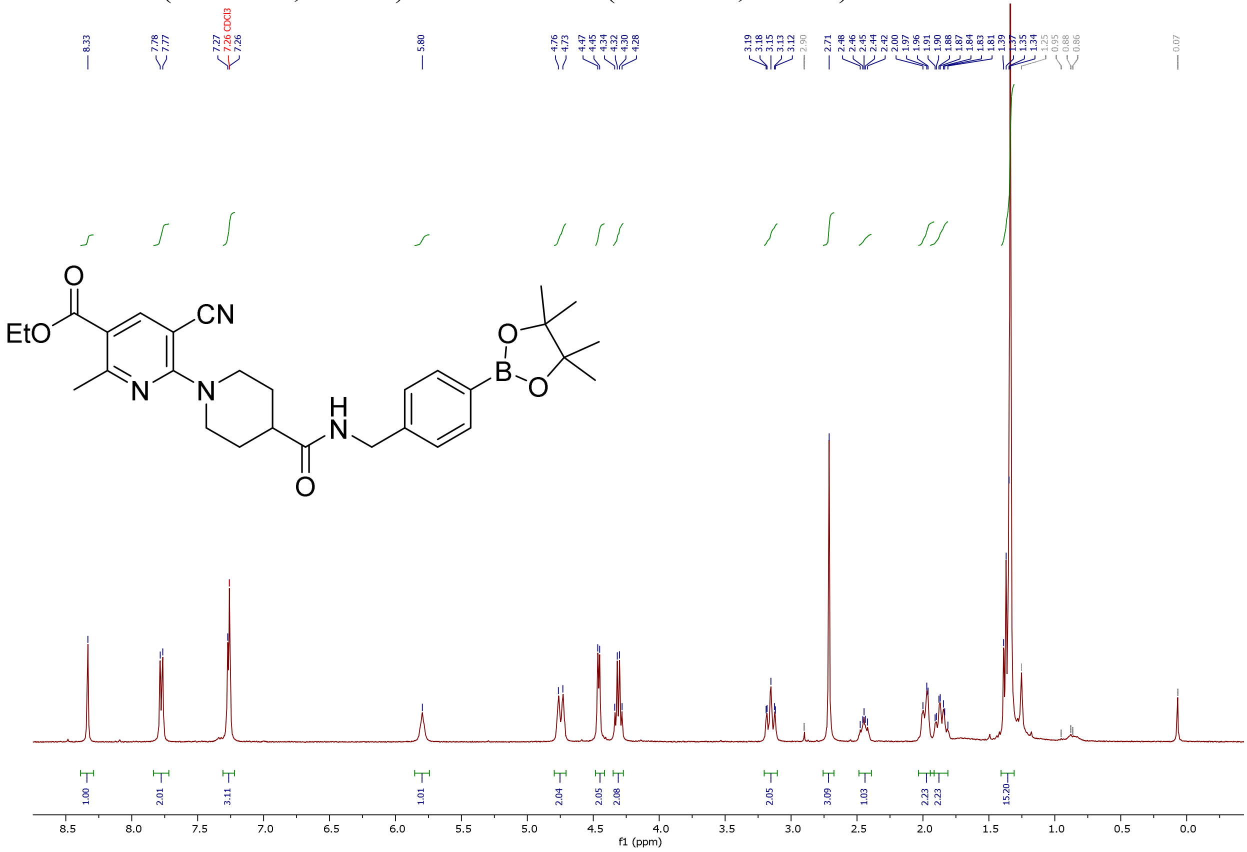

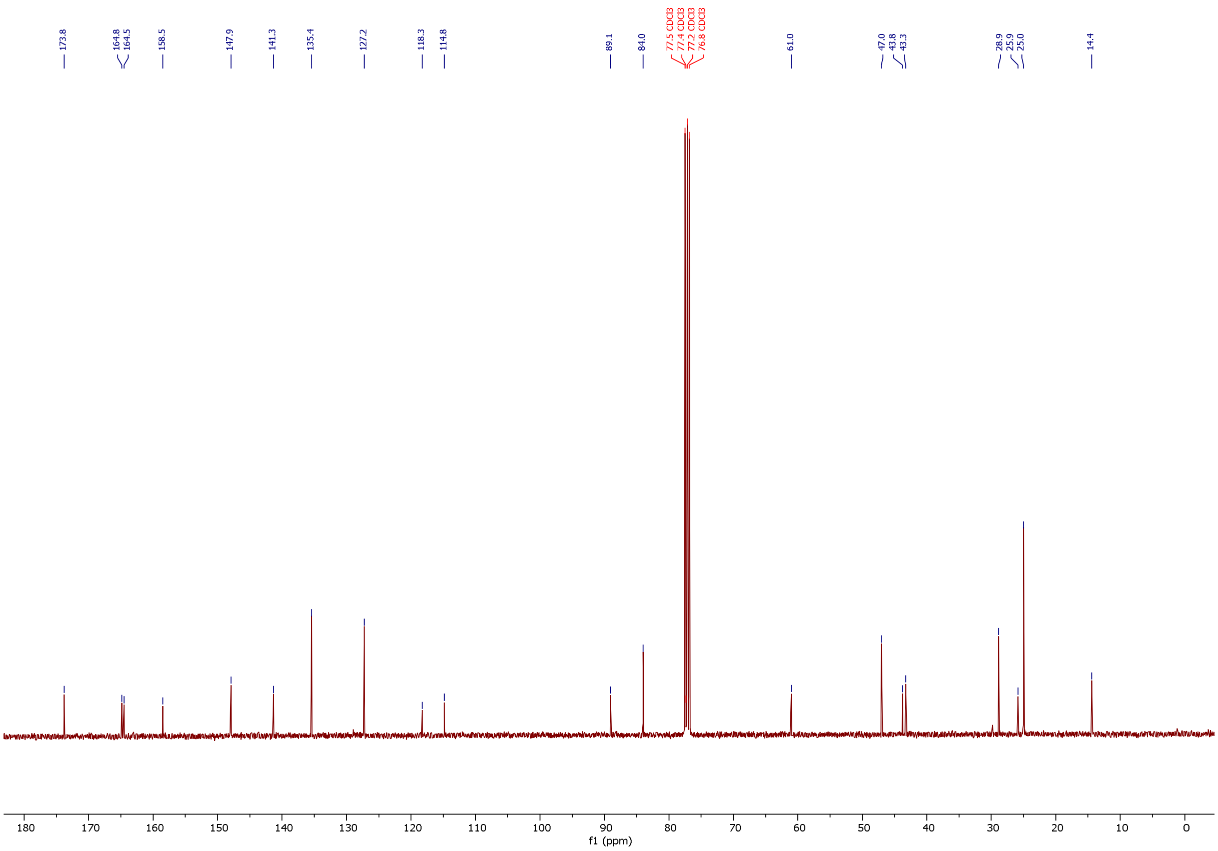

Supplement: Supplementary file 3 [file jm5c00457_si_003.pdf]
